# Lipid lowering therapy in patients with atherosclerotic cardiovascular diseases: Which matters in the real world? Statin intensity or low-density lipoprotein cholesterol level? ‒ Data from a multicenter registry cohort study in Taiwan

**DOI:** 10.1371/journal.pone.0186861

**Published:** 2017-10-26

**Authors:** Yen-Ting Yeh, Wei-Hsian Yin, Wei-Kung Tseng, Fang-Ju Lin, Hung-I Yeh, Jaw-Wen Chen, Yen-Wen Wu, Chau-Chung Wu, on behalf of the Taiwanese Secondary Prevention for Patients with AtheRosCLErotic Disease (T-SPARCLE) Registry Investigators

**Affiliations:** 1 Cardiology Division, Cardiovascular Medical Center, Far Eastern Memorial Hospital, New Taipei City, Taiwan; 2 National Yang-Ming University School of Medicine, Taipei, Taiwan; 3 Division of Cardiology, Heart Center, Cheng-Hsin General Hospital, Taipei, Taiwan; 4 Department of Medical Imaging and Radiological Sciences, I-Shou University, Kaohsiung, Taiwan; 5 Division of Cardiology, Department of Internal Medicine, E-Da Hospital, Kaohsiung, Taiwan; 6 Graduate Institute of Clinical Pharmacy, College of Medicine, National Taiwan University, Taipei, Taiwan; 7 School of Pharmacy, College of Medicine, National Taiwan University, Taipei, Taiwan; 8 Department of Pharmacy, National Taiwan University Hospital, Taipei, Taiwan; 9 Mackay Memorial Hospital, Mackay Medical College, New Taipei City, Taiwan; 10 Department of Medical Research and Education, Taipei Veterans General Hospital, Taipei, Taiwan; 11 Institute of Pharmacology, National Yang-Ming University, Taipei, Taiwan; 12 Cardiology Division, Department of Internal Medicine, National Taiwan University Hospital and National Taiwan University College of Medicine, Taipei, Taiwan; 13 Graduate Institute of Medical Education & Bioethics, College of Medicine, National Taiwan University, Taipei, Taiwan; University of Tampere, FINLAND

## Abstract

**Objective:**

Whether a low-density lipoprotein cholesterol (LDL-C) goal is essential in secondary prevention is still being debated. The aim of our study was to investigate whether achieving particular LDL-C level goals is associated with the reduction in the risk of major adverse cardiac events (MACEs) in patients with atherosclerotic cardiovascular diseases (ASCVD) on statin therapy.

**Methods and results:**

From January 2010 to August 2014, a total of 4099 patients with ASCVD in the Taiwan Secondary Prevention for patients with AtheRosCLErotic disease (T-SPARCLE) registry were analyzed. The risk of a MACE was lower in patients with LDL-C level under control at < 100 mg/dL by statins than in patients with LDL-C level ≥100 mg/dL whether on statin therapy (hazard ratio [HR] 1.66, 95% confidence interval [CI] 1.04‒2.63, *p* = 0.03) or not (HR 2.04, 95% CI 1.06‒3.94, *p* = 0.03). In multivariate Cox model analyses, statin intensity had no significant predictive value, and LDL-C ≥ 100 mg/dL was associated with a slight but not significant trend toward increased risk of MACEs (HR 1.41, 95% CI 0.96‒2.07, *p* = 0.08).

**Conclusions:**

For patients with ASCVD on statin therapy guided by a target-driven strategy, failure to control LDL-C levels to < 100 mg/dL was associated with higher risk of MACEs. Statin intensity alone had no significant impact on the risk of MACEs after multivariate adjustment.

## Introduction

Atherosclerotic cardiovascular disease (ASCVD) is a leading cause of death worldwide, and patients with known ASCVD (especially those with concurrent dyslipidemia) are at high risk for cardiovascular (CV) events [1]. Lipid modification, particularly lowering of low-density lipoprotein cholesterol (LDL-C) with statin therapy, is one of the cornerstones of prevention and treatment for ASCVD. Statins have been shown in multiple large trials and meta-analyses to reduce CV events in patients with ASCVD [2–9]. This is known as secondary prevention.

For a long while, the level at which LDL-C should be controlled has been debated. High-intensity statin therapy has been shown in multiple studies to improve outcomes of patients with ASCVD [10,11]. The 2013 American College of Cardiology/American Heart Association (ACC/AHA) guidelines strongly recommended a fixed-dose strategy of high-intensity statin therapy because most of the studies confirming the efficacy of cholesterol reduction in secondary prevention used a single fixed-dose statin to lower LDL-C levels. And the guidelines made no recommendation for or against specific LDL-C target levels in patients with clinical ASCVD because no randomized controlled study has been conducted to evaluate the titration of statin dosage to achieve specific LDL-C level goals [12]. Since the randomized controlled trials completed to date are unable to answer the question of whether an LDL-C level should be achieved, real-world data from a cohort of patients with ASCVD on statin therapy targeting a specific LDL-C level would be extremely helpful to address this question.

In Taiwan, the National Health Insurance (NHI) covers almost all citizens’ medical expenditures, including expenditures on statin therapy. Based on the NHI revised payment regulation in 2008, the target LDL-C level for secondary prevention is 100 mg/dL and clinicians need to decrease dosage and maintain a minimally-required statin dose once the target level is reached. Therefore, the NHI payment regulation has made patients with ASCVD in Taiwan into basically a cohort on LDL-C goal-directed statin therapy, giving us the opportunity to investigate whether an achieved LDL-C level affects cardiovascular (CV) outcome in patients with ASCVD. Consequently, our study aimed to evaluate whether achieving a specific LDL-C level with statin therapy has an effect on CV outcome in patients with ASCVD.

## Methods

### Study design and setting

The study was conducted using the data from the Taiwan Secondary Prevention for patients with AtheRosCLErotic disease (T-SPARCLE) registry. The T-SPARCLE registry is a multicenter prospective observational registry collecting patients with symptomatic ASCVD at 14 medical sites across Taiwan. The study protocol and previous analysis of the registry have been published previously [13–16]. In this study, we included patients who were treated between January 2010 and August 2014 and had follow-up data as of March 2015.

### Participants

The inclusion criteria were > 18 years old, stable symptomatic atherosclerotic diseases (i.e., coronary atherosclerosis, cerebral vascular disease, or peripheral atherosclerosis), and willingness to follow a National Cholesterol Education Program (NCEP) Therapeutic Lifestyle Change or similar cholesterol-lowering diet. Female patients receiving hormone therapy were included if they were maintained on a stable dose and regimen for at least 8 weeks prior to visit 1 and if they were willing to continue the same regimen throughout the study. Exclusion criteria included serious heart disease, New York Heart Association (NYHA) functional class ≥ III heart failure, life-threatening malignancy, treatment with immunosuppressive agents, unknown type of atherosclerotic vascular disease, taking two statins at enrollment, or any condition or situation which, in the opinion of the investigator, might be not suitable for this registry.

After enrollment, clinical information including medications and laboratory data were collected at every follow-up. Eligible patients who fulfilled the enrollment criteria would be followed every year for a total of 5 years and every 2 years thereafter. The study was approved by the Joint Institutional Review Board, Taiwan, for each participating hospital (JIRB number 09-S-015).

### Main outcomes

The primary endpoint of this study was the occurrence of a first major adverse cardiovascular event (MACE) since enrollment. The MACE is a composite endpoint which includes cardiovascular death, nonfatal stroke (ischemic stroke, hemorrhagic stroke, transient ischemic attack [TIA], or vertebrobasilar insufficiency [VBI]), nonfatal myocardial infarction (MI, including non-ST-elevation MI or ST-elevation MI), or cardiac arrest with resuscitation. The follow-up duration was from enrollment to the occurrence of MACEs or till the last follow-up visit if no MACE occurred.

### Statistical analysis

Patients were stratified into groups by different statin intensity, and baseline characteristics and incidence rates of MACEs were analyzed. Categorical variables are presented as percentage and continuous variables as mean ± standard deviation. Chi-square test was used to compare proportions among groups. Survival analysis was conducted among patients on therapy with different statin intensity, and multivariate Cox proportional hazards (PH) regression modeling was used to identify independent predictors of MACE occurrence while adjusting for the covariates. We further evaluated the risk of MACEs among patients based on distribution of statin use and LDL-C level at enrollment. Hazard ratio (HR) and 95% confidence interval (CI) were estimated. Under missing-at-random (MAR) assumption to handle the missing data, we used multiple imputation (by PROC MI procedure in SAS), which is considered the most effective way of treating missing data and may provide unbiased results with the least influence by the proportion and mechanism of missingness [17]. Multiple imputation acknowledges the uncertainty associated with the imputed values by generating a set of m plausible values (in this study, m = 5) for each unobserved data point, using alternative configurations of covariates. The imputation step resulted in five complete data sets, each with one unique estimate of the missing values. After imputation, we fit Cox proportional hazards model for each complete data set, and then used PROC MIANALYZE procedure in SAS to combine results from the five Cox models (the pooled standard error of the parameter estimate incorporates the uncertainty due to the missing data treatment). Data analyses were performed using the Statistical Analysis System (SAS) version 9.4 (SAS Institute, Cary, NC), and a 2-tailed *p-*value < 0.05 was considered statistically significant.

## Results

**Fig 1:**
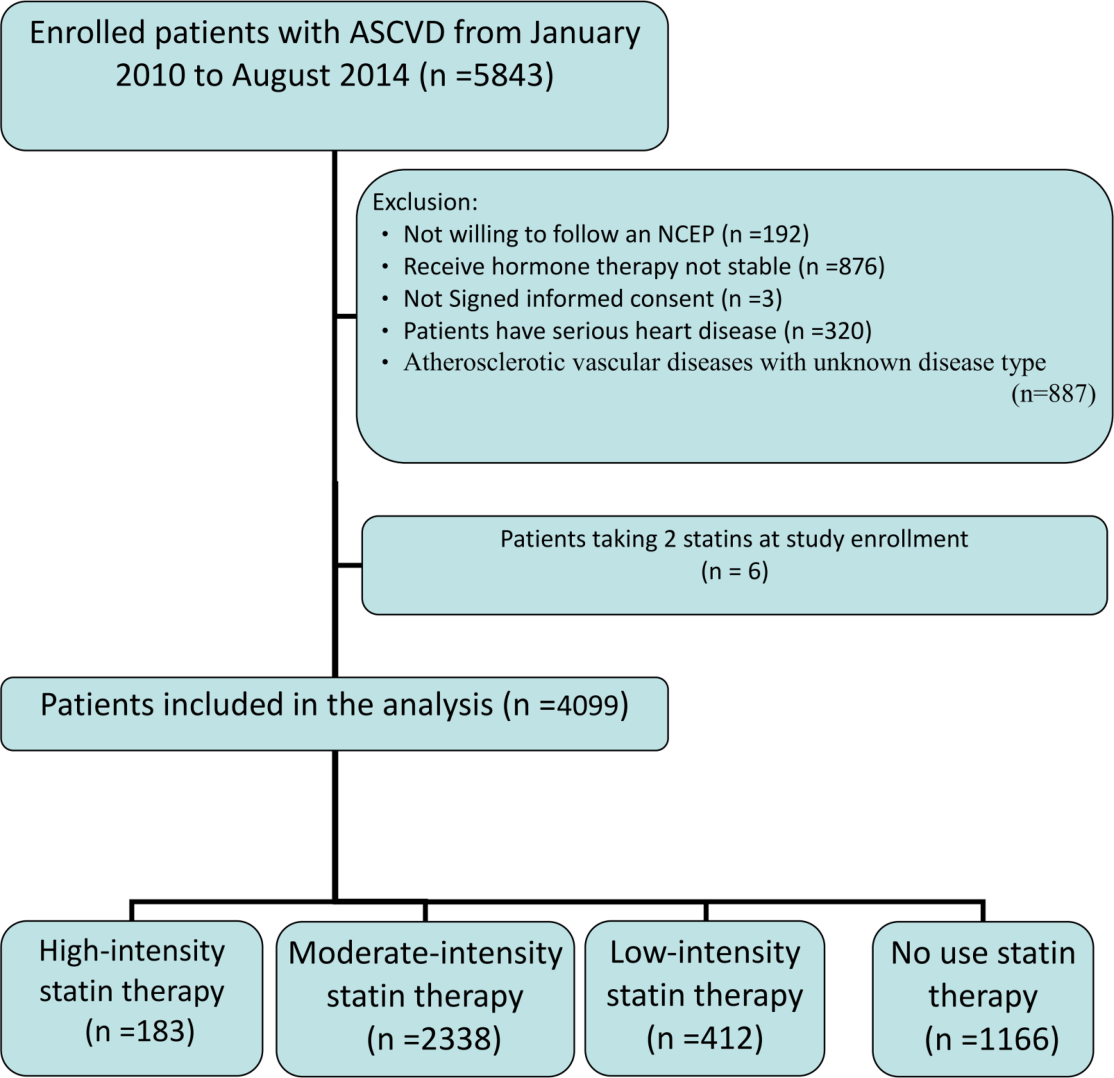
Study flowchart. The study flowchart of T-SPARCLE registry.

From January 2010 to August 2014, a total of 5843 patients met the inclusion criteria, and 4099 of them were included in the final analysis (Fig 1). Of these 4099 patients, the majority (n = 2338, 57%) were on medium-intensity statins at enrollment, 1166 (28%) took no statin at all, and 1781 (43%) failed to achieve the target LDL-C level of < 100 mg/dL. Only a small proportion (n = 183, 4.5%) of the patients took high-intensity statins, reflecting the target-driven nature of the NHI policy on statin therapy. Interestingly, statins were prescribed in only 51% (389 out of 763) of those with prior ischemic stroke or transient ischemic attack. The distribution of patients according to statin intensity and other patient characteristics is presented in Table 1.

**Table 1:** Characteristics and risk factors among patients classified by intensity of statin therapy at enrollment.

	High-intensity statin n = 183		Medium-intensity statin n = 2338		Low-intensity statin n = 412		No statin use n = 1166		*p*-value
Age									
< 75 years	159	(86.9)	1813	(77.5)	292	(70.9)	814	(69.8)	<0.001
≥ 75 years	24	(13.1)	525	(22.5)	120	(29.1)	352	(30.2)	
Gender									
Male	148	(80.9)	1748	(74.8)	318	(77.2)	835	(71.6)	<0.05
Female	35	(19.1)	590	(25.2)	94	(22.8)	331	(28.4)	
Male ≥ 45 years or Female ≥ 55 years	171	(93.4)	2219	(94.9)	407	(98.8)	1096	(94.0)	<0.01
Cigarette smoking history	92	(50.3)	1122	(48.0)	196	(47.6)	468	(40.2)	<0.001
History of hypertension	123	(67.6)	1617	(69.2)	309	(75.0)	894	(76.7)	<0.001
History of heart failure	23	(12.6)	280	(12.0)	45	(10.9)	135	(11.6)	0.91
History of diabetes	70	(42.2)	866	(40.5)	153	(42.3)	389	(35.2)	<0.05
History of coronary artery disease	170	(92.9)	2192	(93.8)	375	(91.0)	855	(73.3)	<0.001
Acute coronary syndrome	155	(84.7)	2051	(87.7)	351	(85.2)	757	(64.9)	<0.001
Ischemic stroke/ transient ischemic attack (TIA)	29	(15.8)	299	(12.8)	61	(14.8)	374	(32.1)	<0.001
Non-ischemic stroke	3	(1.6)	35	(1.5)	9	(2.2)	56	(4.8)	<0.001
Peripheral arterial disease	2	(1.1)	12	(0.5)	2	(0.5)	10	(0.9)	0.41
Chronic kidney disease (eGFR ≤ 60 mL/min)	45	(29.8)	636	(31.5)	99	30.2)	291	(30.5)	0.92
Low HDL-C†	74	(45.4)	923	(44.9)	140	(41.2)	466	(48.9)	0.06
Major risk factors*									
0 RF	4	(2.2)	67	(2.9)	10	(2.4)	33	(2.8)	0.15
1 RF	56	(30.6)	687	(29.4)	126	(30.6)	400	(34.3)	
2 RFs	51	(27.9)	721	(30.8)	104	(25.2)	331	(28.4)	
3 RFs	37	(20.2)	400	(17.1)	78	(18.9)	192	(16.5)	
> 3RFs	35	(19.1)	463	(19.8)	94	(22.8)	210	(18.0)	

Categorical data were all expressed as number (percentage).

eGFR = estimated glomerular filtration rate.

†Low HDL-C was defined as < 40 mg/dL for male and < 50 mg/dL for female.

*Major risk factors included history of smoking, history of hypertension, low HDL-c (<40 mg/dL)

family history of premature coronary heart disease (CHD), age (men≥45 years; women≥55 years)

The median follow-up duration was 2 years, and MACEs occurred in 109 patients. No significant difference in MACE incidence rates was found among statin intensity groups (Table 2). Patients on medium-intensity statins had a higher unadjusted incidence rate of nonfatal myocardial infarction.

**Table 2:** Incidence rate of outcomes among patients classified by intensity of statin therapy.

	High-intensity n = 183	Medium-intensity n = 2338	Low-intensity n = 412	No use n = 1166	*p*-value
MACE (primary outcome)	5.5	15.2	6.9	14.4	0.10
Cardiovascular death	0	2.7	1.4	5.1	0.11
Non-fatal stroke	2.7	4.7	2.8	6.3	0.52
Non-fatal MI	2.7	6.7	2.8	2.1	0.04
Cardiac arrest	0	1.1	0	0.8	0.53

Per 1000 person-years.

CV = cardiovascular; MACE = major adverse cardiovascular event; MI = myocardial infarction

To evaluate the effect of achieving the target LDL-C level on MACE occurrence, we performed multivariate Cox PH regression analysis based on statin use status and LDL-C level reached at baseline (Table 3). Compared to those achieving the LDL-C target (< 100 mg/dL) with statins, patients with LDL-C level ≥ 100 mg/dL had more occurrences of MACEs no matter their statin use status (HR 1.66, 95% CI 1.04‒2.63, *p* = 0.03 for those on statins; HR 2.04, 95% CI 1.06‒3.94, *p* = 0.03 for those not on statins). On the other hand, for patients who achieved the LDL-C target level of < 100 mg/dL, there was no association between statin intensity and risk of MACEs (Table 4). The multivariate Cox PH model identified history of diabetes mellitus (DM), congestive heart failure, cigarette smoking, and chronic kidney disease as independent predictors of MACEs (Table 5). Statin intensity had no significant predictive value, and LDL-C ≥ 100 mg/dL was associated with a trend toward increased risk of MACEs, although the increase was not statistically significant (HR 1.41, 95% CI 0.96‒2.07, *p* = 0.08). After addition of an interaction term of statin use and LDL-C level, the multivariate-adjusted Cox model showed no significant interaction (P > 0.05).

**Table 3:** Multivariate Cox regression model for MACE by joint distribution of statin use status and LDL-C level.

Category	n	Hazard ratio†	95% CI	*p*-value
Under statin LDL-C < 100 mg/dL	1747	1.00	(as reference)	
Not under statin & LDL < 100 mg/dL	571	1.42	0.77–2.63	0.26
Under statin & LDL ≥ 100 mg/dL	1186	1.66	1.04–2.63	0.03
Not under statin & LDL ≥ 100 mg/dL	595	2.04	1.06–3.94	0.03

†Adjusted for age, gender, body mass index (BMI) level, cigarette smoking history, fibrate use, history of hypertension, heart failure, diabetes, myocardial infarction, ischemic stroke or transient ischemic attack, previous coronary or lower extremity arterial disease (LEAD) intervention and levels of estimated glomerular filtration rate (eGFR) at baseline.

**Table 4:** Multivariate Cox regression model for MACE by statin intensity in patients achieving the target of LDL-C < 100 mg/dL.

Category	n	Hazard ratio†	95% CI	*p*-value
Under moderate-intensity statin	1418	1.00	(as reference)	
Under high-intensity statin	99	0.78	0.18–3.35	0.74
Under low-intensity statin	230	0.43	0.10–1.79	0.24
Not under statin	571	1.28	0.68–2.41	0.44

† Adjusted for age, gender, body mass index (BMI) level, cigarette smoking history, fibrate use, history of hypertension, heart failure, diabetes, myocardial infarction, ischemic stroke or transient ischemic attack, previous coronary or lower extremity arterial disease (LEAD) intervention and levels of estimated glomerular filtration rate (eGFR) at baseline.

**Table 5:** Multivariate Cox regression model for predicting MACE.

Parameter	β	Hazard ratio	95% CI	*p*-value
Age	0.01	1.01	0.99–1.03	0.28
Male (vs. female)	-0.42	0.65	0.40–1.08	0.10
BMI (vs. BMI ≥ 27.5)				
BMI < 23	0.20	1.22	0.70–2.14	0.48
23 ≤ BMI < 27.5	0.04	1.04	0.66–1.63	0.87
Cigarette smoking history	0.50	1.65	1.04–2.63	0.03
History of hypertension	0.25	1.28	0.80–2.05	0.30
History of diabetes	0.48	1.62	1.09–2.39	0.02
History of heart failure	0.81	2.24	1.44–3.51	<0.001
History of myocardial infarction	0.61	1.84	0.93–3.66	0.08
Previous coronary/LEAD intervention	0.01	1.01	0.66–1.56	0.95
History of ischemic stroke/ transient ischemic attack	-0.04	0.96	0.53–1.72	0.89
Chronic kidney disease (vs. eGFR > 60 ml/min)				
30 < eGFR ≤ 60 ml/min	0.46	1.59	1.03–2.45	0.04
eGFR ≤ 30 ml/min	1.11	3.04	1.64–5.61	<0.001
LDL-C level (vs. < 100 mg/dL)				
LDL-C ≥ 100 mg/dL	0.34	1.41	0.96–2.07	0.08
Statin therapy intensity (vs. moderate-intensity)				
No statin use	0.09	1.09	0.69–1.72	0.70
Low-intensity	-0.81	0.44	0.18–1.11	0.08
High-intensity	-0.99	0.37	0.09–1.52	0.17
Fibrate use	-0.24	0.79	0.31–2.00	0.62

BMI = body mass index; eGFR = estimated glomerular filtration rate; LEAD = lower extremity arterial disease.

## Discussion

### Principal findings

In this multicenter registry cohort study, we found that failure to achieve an LDL-C target level of < 100 mg/dL, irrespective of statin use, was associated with increased risk of MACEs in patients with ASCVD, and no specific statin intensity had a significant impact on CV outcome. Such findings highlight the importance of keeping LDL-C at goal levels instead of fixing statin intensity without having any specific LDL-C target level to reach.

### Comparison with other studies

Multiple randomized controlled trials (RCTs) have demonstrated that (for patients with ASCVD) the risk of CV events is reduced more by fixed high-intensity statin treatment than fixed lower-dose statin treatment. This finding has resulted in the recommendation of fixed high-intensity statin strategy by the 2013 ACC/AHA guidelines. In these RCTs, high-intensity statin treatment and lower-intensity statin treatment achieved a mean LDL-C level of 67–79 and 97–102 mg/dL, respectively, and these levels differed by 23–30 mg/dL between the 2 groups [12]. In a meta-analysis of 26 RCTs and involving nearly 170,000 patients [18], each reduction in LDL-C level of 1 mmol/L (38.7 mg/dL) resulted in an approximately 22% relative reduction in risk of major vascular events, and there was no variation in the relative reduction of CV risk among these trials after adjusting for LDL-C reduction. This indicates that the reduction in CV risk is due primarily to LDL-C lowering. This conclusion is compatible with the one drawn in this real-world observational study.

In this study, the study population was a cohort of patients with ASCVD on treatment to lower LDL-C to target levels of < 100 mg/dL according to the National Health Insurance (NHI) regulations in Taiwan. Given the nature of this target-driven strategy, the mean achieved LDL-C levels did not differ much (range 97.6–102.7 mg/dL) among statin intensity groups. Since the reduction in CV risk is mainly driven by LDL-C lowering (relative risk reduction of 22% per 1 mmol/L [38.7 mg/dL] LDL-C reduction) according to the meta-analysis mentioned previously [18], it is therefore not surprising that statin intensity was not associated with the risk of MACEs in our study.

Some studies have demonstrated the additional benefit of combining a statin (e.g., simvastatin) with ezetimibe. IMPROVE-IT, a double-blind RCT, demonstrated that combination therapy further lowered the risk of CV events in patients having prior acute coronary syndrome [19]. A nationwide population study using the National Health Insurance Research Database in Taiwan (NHIRD-TW) showed an independent association between the combination therapy and a lower risk of MACEs in patients with DM [20]. In the T-SPARCLE registry, however, only a small proportion (< 5%) of the study population received other lipid-lowering agents in addition to statins, so we could not evaluate the effect of combination therapy on the risk of MACEs in this study.

### Public health impact

The incidence and mortality of acute myocardial infarction (AMI) have continuously declined in developed western countries [21–23]. Improvements in thrombolysis, primary percutaneous coronary interventions (PCI), coronary artery bypass grafting (CABG), and concurrent antiplatelet and antithrombotic therapies, have contributed much to the reduction or control of established CV risk factors, such as hypertension, DM, and dyslipidemia. However, data from the NHIRD-TW showed the decrease in the incidence of ST-elevation myocardial infarction in Taiwan has been only modest compared to that in western countries [24]. Suboptimal control of CV risk factors might partly explain this difference. Based on data obtained from the T-SPARCLE registry, which includes subjects from 14 medical sites across Taiwan, 28% of patients with ASCVD took no statin drug and 43% failed to achieve the LDL-C goal of < 100 mg/dL. This implied that there is still substantial room for improvement in CV risk and outcome.

## Limitations

Our study has several limitations. First, this is an observational cohort study. To directly compare a target-driven strategy to an intensity-driven strategy, we may need an RCT which randomizes the enrolled patients to groups based on each strategy. Second, our multivariate Cox PH model revealed a borderline association between LDL-C ≥ 100 mg/dL and increased risk of MACEs, and our study might be underpowered to detect such an association during a short follow-up period. Third, the relatively low patient numbers in the high-intensity and low-intensity groups might have increased the possibility of type II error. Fourth, data of statin therapy and LDL-C level were all collected at enrollment, and we didn’t have data of therapy discontinuation or modification during the follow-up period, which might have an impact on the outcomes. Fifth, the LDL-C target level in our study cohort was 100 mg/dL not 70 mg/dL, which has been a widely accepted target for patients with ASCVD and high CV risk in western countries. In Caucasians, because the benefit of LDL-C reduction is the same within the range 77–135 mg/dL,^12^ the more optimal treatment target would likely be 70 mg/dL rather than 100 mg/dL. However, there is always an argument that the Asian (compared to the Causcasian) population has less need to use a high-intensity statin or to achieve such a low LDL-C level for secondary prevention. For example, in the Secondary Prevention Cohort Study of the Japan Lipid Intervention Trial (J-LIT), it was found that regulating the serum LDL-C concentration to <120 mg/dL is a reasonable therapeutic strategy to reduce coronary heart disease progression in Japanese patients with prior history under low-dose statin treatment [25]. This issue can be further investigated only if the Taiwanese NHI payment regulation revises the LDL-C target level to < 70 mg/dL for secondary prevention in the future.

## Conclusions

In the real world, for patients with ASCVD on statin therapy guided by a target-driven strategy, failure to control LDL-C levels to < 100 mg/dL was associated with higher risk of MACEs. Statin intensity alone had no significant impact on the risk of MACEs after multivariate adjustment.

## Supporting information

S1 File Supporting tables and figures. Supporting tables and figures of T-SPARCLE registry.
(PDF)

S2 File Statistical data. Statistical data of T-SPARCLE registry.
(PDF)
