# Supplementary material for: Lipid lowering therapy in patients with atherosclerotic cardiovascular diseases: Which matters in the real world? Statin intensity or low-density lipoprotein cholesterol level? ‒ Data from a multicenter registry cohort study in Taiwan
Source: PLoS One. 2017 Oct 26;12(10):e0186861. doi: 10.1371/journal.pone.0186861 (PMC5658082; doi:10.1371/journal.pone.0186861)
Supplement: S1 File — Supporting tables and figures of T-SPARCLE registry. (PDF) [file pone.0186861.s001.pdf]

Table S1. Percentages of censored cases.

Summary of the Number of Censored and Uncensored Values					
Stratum	statin_intensity_HML	Total	Failed	Censored	Percent Censored
1	High-intensity statin dose	183	2	181	98.91
2	Low-intensity statin dose	412	5	407	98.79
3	Medium-intensity statin dose	2338	68	2270	97.09
4	No use statin dose	1166	34	1132	97.08
Total		4099	109	3990	97.34

Figure S1. The Kaplan-Meier plot.

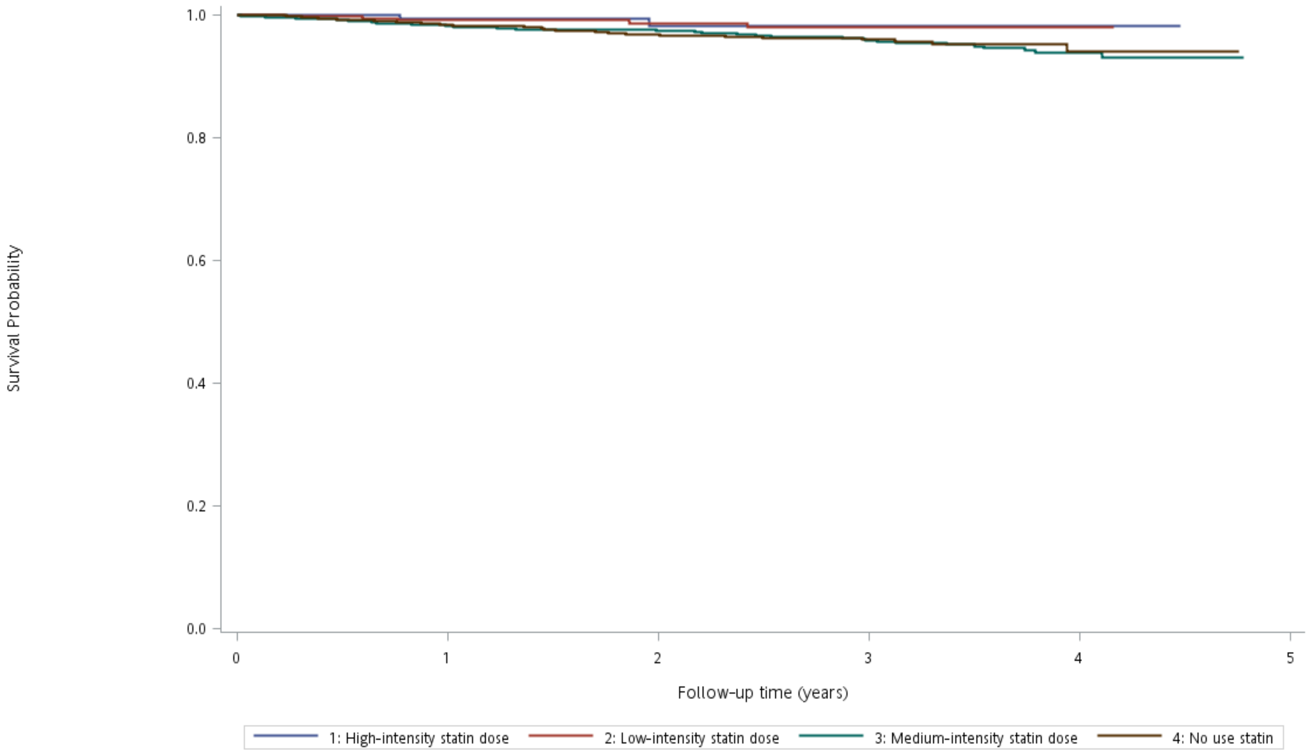

Number of subjects at risk

	0	1	2	3	4	5
1: High-intensity statin dose	183	115	86	51	16	0
2: Low-intensity statin dose	412	270	160	42	7	0
3: Medium-intensity statin dose	2338	1491	1016	507	170	0
4: No use statin	1166	881	516	289	76	0

Table S2. Event numbers of outcomes among patients classified by statin dose intensity

	High-intensity statin dose n=183	Medium-intensity statin dose n=2338	low-intensity statin dose n=412	No use statin dose n=1166	*p* value
Primary outcome*	2	68	5	34	0.20
CV death*	0	12	1	12	0.14
Non-fatal stroke*	1	21	2	15	0.46
Non-fatal MI*	1	30	2	5	0.14
Cardiac arrest*	0	5	0	2	1.0
