# Supplementary material for: Lipid lowering therapy in patients with atherosclerotic cardiovascular diseases: Which matters in the real world? Statin intensity or low-density lipoprotein cholesterol level? ‒ Data from a multicenter registry cohort study in Taiwan
Source: PLoS One. 2017 Oct 26;12(10):e0186861. doi: 10.1371/journal.pone.0186861 (PMC5658082; doi:10.1371/journal.pone.0186861)
Supplement: S2 File — Statistical data of T-SPARCLE registry. (PDF) [file pone.0186861.s002.pdf]

The SAS System

The FREQ Procedure

Table of age_75cutoff by statin_intensity_HML					
age_75cutoff	statin_intensity_HML				
Frequency<br>Percent<br>Row Pct<br>Col Pct	No use statin dose	High-intensity statin dose	Medium-intensity statin dose	Low-intensity statin dose	Total
age<75 year	814<br>19.86<br>26.45<br>69.81	159<br>3.88<br>5.17<br>86.89	1813<br>44.23<br>58.90<br>77.54	292<br>7.12<br>9.49<br>70.87	3078<br>75.09
age>=75 year	352<br>8.59<br>34.48<br>30.19	24<br>0.59<br>2.35<br>13.11	525<br>12.81<br>51.42<br>22.46	120<br>2.93<br>11.75<br>29.13	1021<br>24.91
Total	1166<br>28.45	183<br>4.46	2338<br>57.04	412<br>10.05	4099<br>100.00

Statistics for Table of age_75cutoff by statin_intensity_HML

Statistic	DF	Value	Prob
Chi-Square	3	42.4314	<.0001
Likelihood Ratio Chi-Square	3	43.7327	<.0001
Mantel-Haenszel Chi-Square	1	9.0600	0.0026
Phi Coefficient		0.1017	
Contingency Coefficient		0.1012	
Cramer's V		0.1017	

Fisher's Exact Test	
Table Probability (P)	<.0001
Pr <= P	<.0001

Sample Size = 4099

The SAS System

The FREQ Procedure

Table of Sex by statin_intensity_HML					
Sex(Sex)	statin_intensity_HML				
Frequency<br>Percent<br>Row Pct<br>Col Pct	No use statin dose	High-intensity statin dose	Medium-intensity statin dose	Low-intensity statin dose	Total
Female	331<br>8.08<br>31.52<br>28.39	35<br>0.85<br>3.33<br>19.13	590<br>14.39<br>56.19<br>25.24	94<br>2.29<br>8.95<br>22.82	1050<br>25.62
Male	835<br>20.37<br>27.39<br>71.61	148<br>3.61<br>4.85<br>80.87	1748<br>42.64<br>57.33<br>74.76	318<br>7.76<br>10.43<br>77.18	3049<br>74.38
Total	1166<br>28.45	183<br>4.46	2338<br>57.04	412<br>10.05	4099<br>100.00

Statistics for Table of Sex by statin_intensity_HML

Statistic	DF	Value	Prob
Chi-Square	3	10.6203	0.0140
Likelihood Ratio Chi-Square	3	10.8231	0.0127
Mantel-Haenszel Chi-Square	1	5.1890	0.0227
Phi Coefficient		0.0509	
Contingency Coefficient		0.0508	
Cramer's V		0.0509	

Fisher's Exact Test	
Table Probability (P)	<.0001
Pr <= P	0.0140

Sample Size = 4099

The SAS System

The FREQ Procedure

Table of sex_age by statin_intensity_HML					
sex_age	statin_intensity_HML				
Frequency<br>Percent<br>Row Pct<br>Col Pct	No use statin dose	High-intensity statin dose	Medium-intensity statin dose	Low-intensity statin dose	Total
.	70<br>.<br>.<br>.	12<br>.<br>.<br>.	119<br>.<br>.<br>.	5<br>.<br>.<br>.	.<br>.
Male >= 45 years	801<br>20.58<br>27.41<br>73.08	137<br>3.52<br>4.69<br>80.12	1668<br>42.85<br>57.08<br>75.17	316<br>8.12<br>10.81<br>77.64	2922<br>75.06
Female < 55 years	295<br>7.58<br>30.38<br>26.92	34<br>0.87<br>3.50<br>19.88	551<br>14.15<br>56.75<br>24.83	91<br>2.34<br>9.37<br>22.36	971<br>24.94
Total	1096<br>28.15	171<br>4.39	2219<br>57.00	407<br>10.45	3893<br>100.00
Frequency Missing = 206					

Statistics for Table of sex_age by statin_intensity_HML

Statistic	DF	Value	Prob
Chi-Square	3	6.0845	0.1076
Likelihood Ratio Chi-Square	3	6.2014	0.1022
Mantel-Haenszel Chi-Square	1	2.7334	0.0983
Phi Coefficient		0.0395	
Contingency Coefficient		0.0395	
Cramer's V		0.0395	

Fisher's Exact Test	
Table Probability (P)	<.0001
Pr <= P	0.1103

Effective Sample Size = 3893
Frequency Missing = 206

The SAS System

The FREQ Procedure

Table of Smoking by statin_intensity_HML					
Smoking(Smoking)	statin_intensity_HML				
Frequency<br>Percent<br>Row Pct<br>Col Pct	No use statin dose	High-intensity statin dose	Medium-intensity statin dose	Low-intensity statin dose	Total
.	1<br>.<br>.<br>.	0<br>.<br>.<br>.	3<br>.<br>.<br>.	0<br>.<br>.<br>.	.<br>.
No smoking history	697<br>17.02<br>31.44<br>59.83	91<br>2.22<br>4.10<br>49.73	1213<br>29.62<br>54.71<br>51.95	216<br>5.27<br>9.74<br>52.43	2217<br>54.14
Smoking history	468<br>11.43<br>24.92<br>40.17	92<br>2.25<br>4.90<br>50.27	1122<br>27.40<br>59.74<br>48.05	196<br>4.79<br>10.44<br>47.57	1878<br>45.86
Total	1165<br>28.45	183<br>4.47	2335<br>57.02	412<br>10.06	4095<br>100.00
Frequency Missing = 4					

Statistics for Table of Smoking by statin_intensity_HML

Statistic	DF	Value	Prob
Chi-Square	3	21.6210	<.0001
Likelihood Ratio Chi-Square	3	21.7364	<.0001
Mantel-Haenszel Chi-Square	1	16.6785	<.0001
Phi Coefficient		0.0727	
Contingency Coefficient		0.0725	
Cramer's V		0.0727	

Fisher's Exact Test	
Table Probability (P)	<.0001
Pr <= P	<.0001

Effective Sample Size = 4095
Frequency Missing = 4

The SAS System

The FREQ Procedure

Table of Hx_HTN by statin_intensity_HML					
Hx_HTN(Hx_HTN)	statin_intensity_HML				
Frequency<br>Percent<br>Row Pct<br>Col Pct	No use statin dose	High-intensity statin dose	Medium-intensity statin dose	Low-intensity statin dose	Total
.	0<br>.<br>.<br>.	1<br>.<br>.<br>.	2<br>.<br>.<br>.	0<br>.<br>.<br>.	.<br>.
No HTN history	272<br>6.64<br>23.59<br>23.33	59<br>1.44<br>5.12<br>32.42	719<br>17.55<br>62.36<br>30.78	103<br>2.51<br>8.93<br>25.00	1153<br>28.15
HTN history	894<br>21.83<br>30.38<br>76.67	123<br>3.00<br>4.18<br>67.58	1617<br>39.48<br>54.94<br>69.22	309<br>7.54<br>10.50<br>75.00	2943<br>71.85
Total	1166<br>28.47	182<br>4.44	2336<br>57.03	412<br>10.06	4096<br>100.00
Frequency Missing = 3					

Statistics for Table of Hx_HTN by statin_intensity_HML

Statistic	DF	Value	Prob
Chi-Square	3	25.0502	<.0001
Likelihood Ratio Chi-Square	3	25.4314	<.0001
Mantel-Haenszel Chi-Square	1	9.9457	0.0016
Phi Coefficient		0.0782	
Contingency Coefficient		0.0780	
Cramer's V		0.0782	

Fisher's Exact Test	
Table Probability (P)	<.0001
Pr <= P	<.0001

Effective Sample Size = 4096
Frequency Missing = 3

The SAS System

The FREQ Procedure

Table of Hx_HF by statin_intensity_HML					
Hx_HF(Hx_HF)	statin_intensity_HML				
Frequency<br>Percent<br>Row Pct<br>Col Pct	No use statin dose	High-intensity statin dose	Medium-intensity statin dose	Low-intensity statin dose	Total
.	5<br>.<br>.<br>.	0<br>.<br>.<br>.	4<br>.<br>.<br>.	0<br>.<br>.<br>.	.<br>.
No HF history	1026<br>25.09<br>28.44<br>88.37	160<br>3.91<br>4.44<br>87.43	2054<br>50.22<br>56.94<br>88.00	367<br>8.97<br>10.17<br>89.08	3607<br>88.19
HF history	135<br>3.30<br>27.95<br>11.63	23<br>0.56<br>4.76<br>12.57	280<br>6.85<br>57.97<br>12.00	45<br>1.10<br>9.32<br>10.92	483<br>11.81
Total	1161<br>28.39	183<br>4.47	2334<br>57.07	412<br>10.07	4090<br>100.00
Frequency Missing = 9					

Statistics for Table of Hx_HF by statin_intensity_HML

Statistic	DF	Value	Prob
Chi-Square	3	0.5277	0.9128
Likelihood Ratio Chi-Square	3	0.5327	0.9116
Mantel-Haenszel Chi-Square	1	0.0016	0.9682
Phi Coefficient		0.0114	
Contingency Coefficient		0.0114	
Cramer's V		0.0114	

Fisher's Exact Test	
Table Probability (P)	0.0002
Pr <= P	0.9126

Effective Sample Size = 4090
Frequency Missing = 9

The SAS System

The FREQ Procedure

Table of new_DM by statin_intensity_HML					
new_DM	statin_intensity_HML				
Frequency<br>Percent<br>Row Pct<br>Col Pct	No use statin dose	High-intensity statin dose	Medium-intensity statin dose	Low-intensity statin dose	Total
.	60<br>.<br>.<br>.	17<br>.<br>.<br>.	201<br>.<br>.<br>.	50<br>.<br>.<br>.	.<br>.
No DM history	717<br>19.01<br>31.27<br>64.83	96<br>2.55<br>4.19<br>57.83	1271<br>33.70<br>55.43<br>59.48	209<br>5.54<br>9.11<br>57.73	2293<br>60.81
DM history	389<br>10.32<br>26.32<br>35.17	70<br>1.86<br>4.74<br>42.17	866<br>22.96<br>58.59<br>40.52	153<br>4.06<br>10.35<br>42.27	1478<br>39.19
Total	1106<br>29.33	166<br>4.40	2137<br>56.67	362<br>9.60	3771<br>100.00
Frequency Missing = 328					

Statistics for Table of new_DM by statin_intensity_HML

Statistic	DF	Value	Prob
Chi-Square	3	11.1433	0.0110
Likelihood Ratio Chi-Square	3	11.2218	0.0106
Mantel-Haenszel Chi-Square	1	9.8024	0.0017
Phi Coefficient		0.0544	
Contingency Coefficient		0.0543	
Cramer's V		0.0544	

Fisher's Exact Test	
Table Probability (P)	<.0001
Pr <= P	0.0104

Effective Sample Size = 3771
Frequency Missing = 328

The SAS System

The FREQ Procedure

Table of IC2a_CAD_asmissing by statin_intensity_HML					
IC2a_CAD_asmissing	statin_intensity_HML				
Frequency<br>Percent<br>Row Pct<br>Col Pct	No use statin dose	High-intensity statin dose	Medium-intensity statin dose	Low-intensity statin dose	Total
No CAD history	311<br>7.59<br>61.34<br>26.67	13<br>0.32<br>2.56<br>7.10	146<br>3.56<br>28.80<br>6.24	37<br>0.90<br>7.30<br>8.98	507<br>12.37
CAD history	855<br>20.86<br>23.80<br>73.33	170<br>4.15<br>4.73<br>92.90	2192<br>53.48<br>61.02<br>93.76	375<br>9.15<br>10.44<br>91.02	3592<br>87.63
Total	1166<br>28.45	183<br>4.46	2338<br>57.04	412<br>10.05	4099<br>100.00

Statistics for Table of IC2a_CAD_asmissing by statin_intensity_HML

Statistic	DF	Value	Prob
Chi-Square	3	310.0333	<.0001
Likelihood Ratio Chi-Square	3	280.0163	<.0001
Mantel-Haenszel Chi-Square	1	251.9160	<.0001
Phi Coefficient		0.2750	
Contingency Coefficient		0.2652	
Cramer's V		0.2750	

Fisher's Exact Test	
Table Probability (P)	<.0001
Pr <= P	<.0001

Sample Size = 4099

The SAS System

The FREQ Procedure

Table of ACS_asmissing by statin_intensity_HML					
ACS_asmissing	statin_intensity_HML				
Frequency<br>Percent<br>Row Pct<br>Col Pct	No use statin dose	High-intensity statin dose	Medium-intensity statin dose	Low-intensity statin dose	Total
No ACS history	409<br>9.98<br>52.10<br>35.08	28<br>0.68<br>3.57<br>15.30	287<br>7.00<br>36.56<br>12.28	61<br>1.49<br>7.77<br>14.81	785<br>19.15
ACS history	757<br>18.47<br>22.84<br>64.92	155<br>3.78<br>4.68<br>84.70	2051<br>50.04<br>61.89<br>87.72	351<br>8.56<br>10.59<br>85.19	3314<br>80.85
Total	1166<br>28.45	183<br>4.46	2338<br>57.04	412<br>10.05	4099<br>100.00

Statistics for Table of ACS_asmissing by statin_intensity_HML

Statistic	DF	Value	Prob
Chi-Square	3	269.1689	<.0001
Likelihood Ratio Chi-Square	3	249.6213	<.0001
Mantel-Haenszel Chi-Square	1	225.7099	<.0001
Phi Coefficient		0.2563	
Contingency Coefficient		0.2482	
Cramer's V		0.2563	

Fisher's Exact Test	
Table Probability (P)	<.0001
Pr <= P	<.0001

Sample Size = 4099

The SAS System

The FREQ Procedure

Table of IschTIA_asmissing by statin_intensity_HML					
IschTIA_asmissing	statin_intensity_HML				
Frequency<br>Percent<br>Row Pct<br>Col Pct	No use statin dose	High-intensity statin dose	Medium-intensity statin dose	Low-intensity statin dose	Total
No Ischemic/TIA history	792<br>19.32<br>23.74<br>67.92	154<br>3.76<br>4.62<br>84.15	2039<br>49.74<br>61.12<br>87.21	351<br>8.56<br>10.52<br>85.19	3336<br>81.39
Ischemic/TIA history	374<br>9.12<br>49.02<br>32.08	29<br>0.71<br>3.80<br>15.85	299<br>7.29<br>39.19<br>12.79	61<br>1.49<br>7.99<br>14.81	763<br>18.61
Total	1166<br>28.45	183<br>4.46	2338<br>57.04	412<br>10.05	4099<br>100.00

Statistics for Table of IschTIA_asmissing by statin_intensity_HML

Statistic	DF	Value	Prob
Chi-Square	3	196.7118	<.0001
Likelihood Ratio Chi-Square	3	183.2430	<.0001
Mantel-Haenszel Chi-Square	1	166.2868	<.0001
Phi Coefficient		0.2191	
Contingency Coefficient		0.2140	
Cramer's V		0.2191	

Fisher's Exact Test	
Table Probability (P)	<.0001
Pr <= P	<.0001

Sample Size = 4099

The SAS System

The FREQ Procedure

Table of IC2a_CVA_HemoStroke_asmissing by statin_intensity_HML					
IC2a_CVA_HemoStroke_asmissing	statin_intensity_HML				
Frequency<br>Percent<br>Row Pct<br>Col Pct	No use statin dose	High-intensity statin dose	Medium-intensity statin dose	Low-intensity statin dose	Total
No Hemorrhagic history	1110<br>27.08<br>27.78<br>95.20	180<br>4.39<br>4.50<br>98.36	2303<br>56.18<br>57.63<br>98.50	403<br>9.83<br>10.09<br>97.82	3996<br>97.49
Hemorrhagic history	56<br>1.37<br>54.37<br>4.80	3<br>0.07<br>2.91<br>1.64	35<br>0.85<br>33.98<br>1.50	9<br>0.22<br>8.74<br>2.18	103<br>2.51
Total	1166<br>28.45	183<br>4.46	2338<br>57.04	412<br>10.05	4099<br>100.00

Statistics for Table of IC2a_CVA_HemoStroke_asmissing by statin_intensity_HML

Statistic	DF	Value	Prob
Chi-Square	3	35.5591	<.0001
Likelihood Ratio Chi-Square	3	32.1112	<.0001
Mantel-Haenszel Chi-Square	1	27.5870	<.0001
Phi Coefficient		0.0931	
Contingency Coefficient		0.0927	
Cramer's V		0.0931	

Fisher's Exact Test	
Table Probability (P)	<.0001
Pr <= P	<.0001

Sample Size = 4099

The SAS System

The FREQ Procedure

Table of IC2a_PAD_asmissing by statin_intensity_HML					
IC2a_PAD_asmissing	statin_intensity_HML				
Frequency<br>Percent<br>Row Pct<br>Col Pct	No use statin dose	High-intensity statin dose	Medium-intensity statin dose	Low-intensity statin dose	Total
No PAD history	1156<br>28.20<br>28.38<br>99.14	181<br>4.42<br>4.44<br>98.91	2326<br>56.75<br>57.11<br>99.49	410<br>10.00<br>10.07<br>99.51	4073<br>99.37
PAD history	10<br>0.24<br>38.46<br>0.86	2<br>0.05<br>7.69<br>1.09	12<br>0.29<br>46.15<br>0.51	2<br>0.05<br>7.69<br>0.49	26<br>0.63
Total	1166<br>28.45	183<br>4.46	2338<br>57.04	412<br>10.05	4099<br>100.00

Statistics for Table of IC2a_PAD_asmissing by statin_intensity_HML

Statistic	DF	Value	Prob
Chi-Square	3	2.2217	0.5277
Likelihood Ratio Chi-Square	3	2.0717	0.5577
Mantel-Haenszel Chi-Square	1	1.6835	0.1945
Phi Coefficient		0.0233	
Contingency Coefficient		0.0233	
Cramer's V		0.0233	
WARNING: 25% of the cells have expected counts less than 5. Chi-Square may not be a valid test.			

Fisher's Exact Test	
Table Probability (P)	0.0048
Pr <= P	0.4118

Sample Size = 4099

The SAS System

The FREQ Procedure

Table of CKD_Binary by statin_intensity_HML					
CKD_Binary	statin_intensity_HML				
Frequency<br>Percent<br>Row Pct<br>Col Pct	No use statin dose	High-intensity statin dose	Medium-intensity statin dose	Low-intensity statin dose	Total
.	212<br>.<br>.<br>.	32<br>.<br>.<br>.	319<br>.<br>.<br>.	84<br>.<br>.<br>.	.<br>.
eGFR >60	663<br>19.21<br>27.85<br>69.50	106<br>3.07<br>4.45<br>70.20	1383<br>40.06<br>58.08<br>68.50	229<br>6.63<br>9.62<br>69.82	2381<br>68.97
eGFR <=60	291<br>8.43<br>27.17<br>30.50	45<br>1.30<br>4.20<br>29.80	636<br>18.42<br>59.38<br>31.50	99<br>2.87<br>9.24<br>30.18	1071<br>31.03
Total	954<br>27.64	151<br>4.37	2019<br>58.49	328<br>9.50	3452<br>100.00
Frequency Missing = 647					

Statistics for Table of CKD_Binary by statin_intensity_HML

Statistic	DF	Value	Prob
Chi-Square	3	0.5493	0.9079
Likelihood Ratio Chi-Square	3	0.5504	0.9077
Mantel-Haenszel Chi-Square	1	0.1117	0.7382
Phi Coefficient		0.0126	
Contingency Coefficient		0.0126	
Cramer's V		0.0126	

Fisher's Exact Test	
Table Probability (P)	<.0001
Pr <= P	0.9157

Effective Sample Size = 3452
Frequency Missing = 647

WARNING: 16% of the data are missing.

The SAS System

The FREQ Procedure

Table of HDL_Binary by statin_intensity_HML					
HDL_Binary	statin_intensity_HML				
Frequency<br>Percent<br>Row Pct<br>Col Pct	No use statin dose	High-intensity statin dose	Medium-intensity statin dose	Low-intensity statin dose	Total
.	213<br>.<br>.<br>.	20<br>.<br>.<br>.	282<br>.<br>.<br>.	72<br>.<br>.<br>.	.<br>.
Low HDL-c	466<br>13.27<br>29.07<br>48.90	74<br>2.11<br>4.62<br>45.40	923<br>26.28<br>57.58<br>44.89	140<br>3.99<br>8.73<br>41.18	1603<br>45.64
High HDL-c	487<br>13.87<br>25.51<br>51.10	89<br>2.53<br>4.66<br>54.60	1133<br>32.26<br>59.35<br>55.11	200<br>5.69<br>10.48<br>58.82	1909<br>54.36
Total	953<br>27.14	163<br>4.64	2056<br>58.54	340<br>9.68	3512<br>100.00
Frequency Missing = 587					

Statistics for Table of HDL_Binary by statin_intensity_HML

Statistic	DF	Value	Prob
Chi-Square	3	7.2743	0.0637
Likelihood Ratio Chi-Square	3	7.2803	0.0635
Mantel-Haenszel Chi-Square	1	6.8657	0.0088
Phi Coefficient		0.0455	
Contingency Coefficient		0.0455	
Cramer's V		0.0455	

Fisher's Exact Test	
Table Probability (P)	<.0001
Pr <= P	0.0638

Effective Sample Size = 3512
Frequency Missing = 587

WARNING: 14% of the data are missing.

The SAS System

The FREQ Procedure

Table of RF_Level by statin_intensity_HML					
RF_Level	statin_intensity_HML				
Frequency<br>Percent<br>Row Pct<br>Col Pct	No use statin dose	High-intensity statin dose	Medium-intensity statin dose	Low-intensity statin dose	Total
0 Risk factor	33<br>0.81<br>28.95<br>2.83	4<br>0.10<br>3.51<br>2.19	67<br>1.63<br>58.77<br>2.87	10<br>0.24<br>8.77<br>2.43	114<br>2.78
1 Risk factor	400<br>9.76<br>31.52<br>34.31	56<br>1.37<br>4.41<br>30.60	687<br>16.76<br>54.14<br>29.38	126<br>3.07<br>9.93<br>30.58	1269<br>30.96
2 Risk factor	331<br>8.08<br>27.42<br>28.39	51<br>1.24<br>4.23<br>27.87	721<br>17.59<br>59.73<br>30.84	104<br>2.54<br>8.62<br>25.24	1207<br>29.45
3 Risk factor	192<br>4.68<br>27.16<br>16.47	37<br>0.90<br>5.23<br>20.22	400<br>9.76<br>56.58<br>17.11	78<br>1.90<br>11.03<br>18.93	707<br>17.25
>3 Risk factor	210<br>5.12<br>26.18<br>18.01	35<br>0.85<br>4.36<br>19.13	463<br>11.30<br>57.73<br>19.80	94<br>2.29<br>11.72<br>22.82	802<br>19.57
Total	1166<br>28.45	183<br>4.46	2338<br>57.04	412<br>10.05	4099<br>100.00

Statistics for Table of RF_Level by statin_intensity_HML

Statistic	DF	Value	Prob
Chi-Square	12	17.0194	0.1489
Likelihood Ratio Chi-Square	12	16.9074	0.1531
Mantel-Haenszel Chi-Square	1	7.3472	0.0067
Phi Coefficient		0.0644	
Contingency Coefficient		0.0643	
Cramer's V		0.0372	

Sample Size = 4099

phregparms_mianalyze_mi_4099

Obs	statin_intensity_HML	IR_Prim	IR_Prim_CVdeath	IR_Stroke	IR_MI	IR_Cardi
1	No use statin dose	14.4	5.1	6.3	2.1	0.8
2	High-intensity statin dose	5.5	0.0	2.7	2.7	0.0
3	Medium-intensity statin dose	15.2	2.7	4.7	6.7	1.1
4	Low-intensity statin dose	6.9	1.4	2.8	2.8	0.0

phregparms_mianalyze_mi_4099

The GENMOD Procedure

Imputation Number=1

Model Information		
Data Set	WORK.OUT_LIPID_MI_IMPUTATION_1	
Distribution	Poisson	
Link Function	Log	
Dependent Variable	Prim_Outc	Prim_Outc
Offset Variable	logPrim_YRS	

Number of Observations Read	4099
Number of Observations Used	4099

Class Level Information		
Class	Levels	Values
statin_intensity_HML	4	High-intensity statin dose Low-intensity statin dose Medium-intensity statin dose No use statin dose

Criteria For Assessing Goodness Of Fit			
Criterion	DF	Value	Value/DF
Deviance	4095	870.0446	0.2125
Scaled Deviance	4095	870.0446	0.2125
Pearson Chi-Square	4095	10243.8674	2.5016
Scaled Pearson X2	4095	10243.8674	2.5016
Log Likelihood		-544.0223	
Full Log Likelihood		-544.0223	
AIC (smaller is better)		1096.0446	
AICC (smaller is better)		1096.0544	
BIC (smaller is better)		1121.3186	

Algorithm converged.

phregparms_mianalyze_mi_4099

The GENMOD Procedure

Imputation Number=1

Analysis Of Maximum Likelihood Parameter Estimates								
Parameter		DF	Estimate	Standard Error	Wald 95% Confidence Limits		Wald Chi-Square	Pr > ChiSq
Intercept		1	-4.2225	0.1715	-4.5586	-3.8863	606.19	<.0001
statin_intensity_HML	High-intensity statin dose	1	-0.9680	0.7276	-2.3941	0.4580	1.77	0.1834
statin_intensity_HML	Low-intensity statin dose	1	-0.7531	0.4790	-1.6918	0.1857	2.47	0.1159
statin_intensity_HML	Medium-intensity statin dose	1	0.0445	0.2100	-0.3671	0.4562	0.04	0.8321
statin_intensity_HML	No use statin dose	0	0.0000	0.0000	0.0000	0.0000	.	.
Scale		0	1.0000	0.0000	1.0000	1.0000		

Note: The scale parameter was held fixed.

LR Statistics For Type 3 Analysis			
Source	DF	Chi-Square	Pr > ChiSq
statin_intensity_HML	3	6.19	0.1029

phregparms_mianalyze_mi_4099

The GENMOD Procedure

Imputation Number=1

Model Information	
Data Set	WORK.OUT_LIPID_MI_IMPUTATION_1
Distribution	Poisson
Link Function	Log
Dependent Variable	Prim_CVdeath
Offset Variable	logPrim_YRS

Number of Observations Read	4099
Number of Observations Used	4099

Class Level Information		
Class	Levels	Values
statin_intensity_HML	4	High-intensity statin dose Low-intensity statin dose Medium-intensity statin dose No use statin dose

Criteria For Assessing Goodness Of Fit			
Criterion	DF	Value	Value/DF
Deviance	4095	293.4050	0.0716
Scaled Deviance	4095	293.4050	0.0716
Pearson Chi-Square	4095	18223.4350	4.4502
Scaled Pearson X2	4095	18223.4350	4.4502
Log Likelihood		-171.7025	
Full Log Likelihood		-171.7025	
AIC (smaller is better)		351.4050	
AICC (smaller is better)		351.4148	
BIC (smaller is better)		376.6790	

Algorithm converged.

phregparms_mianalyze_mi_4099

The GENMOD Procedure

Imputation Number=1

Analysis Of Maximum Likelihood Parameter Estimates								
Parameter		DF	Estimate	Standard Error	Wald 95% Confidence Limits		Wald Chi-Square	Pr > ChiSq
Intercept		1	-5.2639	0.2887	-5.8297	-4.6981	332.51	<.0001
statin_intensity_HML	High-intensity statin dose	1	-22.8281	66449.82	-130262	130216.4	0.00	0.9997
statin_intensity_HML	Low-intensity statin dose	1	-1.3211	1.0408	-3.3611	0.7189	1.61	0.2044
statin_intensity_HML	Medium-intensity statin dose	1	-0.6486	0.4082	-1.4488	0.1515	2.52	0.1121
statin_intensity_HML	No use statin dose	0	0.0000	0.0000	0.0000	0.0000	.	.
Scale		0	1.0000	0.0000	1.0000	1.0000		

Note: The scale parameter was held fixed.

LR Statistics For Type 3 Analysis			
Source	DF	Chi-Square	Pr > ChiSq
statin_intensity_HML	3	5.99	0.1122

phregparms_mianalyze_mi_4099

The GENMOD Procedure

Imputation Number=1

Model Information	
Data Set	WORK.OUT_LIPID_MI_IMPUTATION_1
Distribution	Poisson
Link Function	Log
Dependent Variable	Prim_NonfatalStroke
Offset Variable	logPrim_YRS

Number of Observations Read	4099
Number of Observations Used	4099

Class Level Information		
Class	Levels	Values
statin_intensity_HML	4	High-intensity statin dose Low-intensity statin dose Medium-intensity statin dose No use statin dose

Criteria For Assessing Goodness Of Fit			
Criterion	DF	Value	Value/DF
Deviance	4095	380.1807	0.0928
Scaled Deviance	4095	380.1807	0.0928
Pearson Chi-Square	4095	7432.2171	1.8149
Scaled Pearson X2	4095	7432.2171	1.8149
Log Likelihood		-229.0903	
Full Log Likelihood		-229.0903	
AIC (smaller is better)		466.1807	
AICC (smaller is better)		466.1905	
BIC (smaller is better)		491.4547	

Algorithm converged.

phregparms_mianalyze_mi_4099

The GENMOD Procedure

Imputation Number=1

Analysis Of Maximum Likelihood Parameter Estimates								
Parameter		DF	Estimate	Standard Error	Wald 95% Confidence Limits		Wald Chi-Square	Pr > ChiSq
Intercept		1	-5.0408	0.2582	-5.5468	-4.5347	381.14	<.0001
statin_intensity_HML	High-intensity statin dose	1	-0.8429	1.0328	-2.8671	1.1814	0.67	0.4144
statin_intensity_HML	Low-intensity statin dose	1	-0.8511	0.7528	-2.3265	0.6243	1.28	0.2582
statin_intensity_HML	Medium-intensity statin dose	1	-0.3121	0.3381	-0.9747	0.3504	0.85	0.3558
statin_intensity_HML	No use statin dose	0	0.0000	0.0000	0.0000	0.0000	.	.
Scale		0	1.0000	0.0000	1.0000	1.0000		

Note: The scale parameter was held fixed.

LR Statistics For Type 3 Analysis			
Source	DF	Chi-Square	Pr > ChiSq
statin_intensity_HML	3	2.27	0.5192

phregparms_mianalyze_mi_4099

The GENMOD Procedure

Imputation Number=1

Model Information	
Data Set	WORK.OUT_LIPID_MI_IMPUTATION_1
Distribution	Poisson
Link Function	Log
Dependent Variable	Prim_NonfatalMI
Offset Variable	logPrim_YRS

Number of Observations Read	4099
Number of Observations Used	4099

Class Level Information		
Class	Levels	Values
statin_intensity_HML	4	High-intensity statin dose Low-intensity statin dose Medium-intensity statin dose No use statin dose

Criteria For Assessing Goodness Of Fit			
Criterion	DF	Value	Value/DF
Deviance	4095	365.9033	0.0894
Scaled Deviance	4095	365.9033	0.0894
Pearson Chi-Square	4095	5992.7496	1.4634
Scaled Pearson X2	4095	5992.7496	1.4634
Log Likelihood		-220.9517	
Full Log Likelihood		-220.9517	
AIC (smaller is better)		449.9033	
AICC (smaller is better)		449.9131	
BIC (smaller is better)		475.1773	

Algorithm converged.

phregparms_mianalyze_mi_4099

The GENMOD Procedure

Imputation Number=1

Analysis Of Maximum Likelihood Parameter Estimates								
Parameter		DF	Estimate	Standard Error	Wald 95% Confidence Limits		Wald Chi-Square	Pr > ChiSq
Intercept		1	-6.1394	0.4472	-7.0159	-5.2629	188.46	<.0001
statin_intensity_HML	High-intensity statin dose	1	0.2557	1.0954	-1.8913	2.4028	0.05	0.8154
statin_intensity_HML	Low-intensity statin dose	1	0.2475	0.8367	-1.3923	1.8874	0.09	0.7673
statin_intensity_HML	Medium-intensity statin dose	1	1.1431	0.4830	0.1964	2.0899	5.60	0.0180
statin_intensity_HML	No use statin dose	0	0.0000	0.0000	0.0000	0.0000	.	.
Scale		0	1.0000	0.0000	1.0000	1.0000		

Note: The scale parameter was held fixed.

LR Statistics For Type 3 Analysis			
Source	DF	Chi-Square	Pr > ChiSq
statin_intensity_HML	3	8.51	0.0366

phregparms_mianalyze_mi_4099

The GENMOD Procedure

Imputation Number=1

Model Information	
Data Set	WORK.OUT_LIPID_MI_IMPUTATION_1
Distribution	Poisson
Link Function	Log
Dependent Variable	Prim_CardiacArrestRESUS
Offset Variable	logPrim_YRS

Number of Observations Read	4099
Number of Observations Used	4099

Class Level Information		
Class	Levels	Values
statin_intensity_HML	4	High-intensity statin dose Low-intensity statin dose Medium-intensity statin dose No use statin dose

Criteria For Assessing Goodness Of Fit			
Criterion	DF	Value	Value/DF
Deviance	4095	90.1028	0.0220
Scaled Deviance	4095	90.1028	0.0220
Pearson Chi-Square	4095	7240.9992	1.7683
Scaled Pearson X2	4095	7240.9992	1.7683
Log Likelihood		-52.0514	
Full Log Likelihood		-52.0514	
AIC (smaller is better)		112.1028	
AICC (smaller is better)		112.1125	
BIC (smaller is better)		137.3768	

Algorithm converged.

phregparms_mianalyze_mi_4099

The GENMOD Procedure

Imputation Number=1

Analysis Of Maximum Likelihood Parameter Estimates								
Parameter		DF	Estimate	Standard Error	Wald 95% Confidence Limits		Wald Chi-Square	Pr > ChiSq
Intercept		1	-7.0557	0.7071	-8.4416	-5.6698	99.57	<.0001
statin_intensity_HML	High-intensity statin dose	1	-23.0364	180629.3	-354050	354004.0	0.00	0.9999
statin_intensity_HML	Low-intensity statin dose	1	-22.9957	124642.3	-244317	244271.4	0.00	0.9999
statin_intensity_HML	Medium-intensity statin dose	1	0.2677	0.8367	-1.3721	1.9075	0.10	0.7490
statin_intensity_HML	No use statin dose	0	0.0000	0.0000	0.0000	0.0000	.	.
Scale		0	1.0000	0.0000	1.0000	1.0000		

Note: The scale parameter was held fixed.

LR Statistics For Type 3 Analysis			
Source	DF	Chi-Square	Pr > ChiSq
statin_intensity_HML	3	2.19	0.5342

derive.out_lipid_mi_4099

The PHREG Procedure

Imputation Number=1

Model Information		
Data Set	WORK.OUT_LIPID_MI_4099	
Dependent Variable	Prim_Outc_Time	
Censoring Variable	Prim_Outc	Prim_Outc
Censoring Value(s)	0	
Ties Handling	BRESLOW	

Number of Observations Read	4099
Number of Observations Used	2315

Summary of the Number of Event and Censored Values			
Total	Event	Censored	Percent Censored
2315	56	2259	97.58

Convergence Status
Convergence criterion (GCONV=1E-8) satisfied.

Model Fit Statistics		
Criterion	Without Covariates	With Covariates
-2 LOG L	796.026	763.237
AIC	796.026	793.237
SBC	796.026	823.617

Testing Global Null Hypothesis: BETA=0			
Test	Chi-Square	DF	Pr > ChiSq
Likelihood Ratio	32.7899	15	0.0050
Score	40.0039	15	0.0005
Wald	36.5820	15	0.0015

derive.out_lipid_mi_4099

The PHREG Procedure

Imputation Number=1

Analysis of Maximum Likelihood Estimates									
Parameter	DF	Parameter Estimate	Standard Error	Chi-Square	Pr > ChiSq	Hazard Ratio	95% Hazard Ratio Confidence Limits		Label
Age	1	0.01470	0.01352	1.1811	0.2771	1.015	0.988	1.042	
Male (vs. female)	1	-0.46065	0.34581	1.7744	0.1828	0.631	0.320	1.243	Male (vs. female)
History of HTN	1	0.20624	0.33440	0.3804	0.5374	1.229	0.638	2.367	History of HTN
History of DM	1	0.25093	0.27915	0.8081	0.3687	1.285	0.744	2.221	
History of HF	1	0.93284	0.29955	9.6979	0.0018	2.542	1.413	4.572	History of HF
History of MI	1	0.64772	0.47104	1.8909	0.1691	1.911	0.759	4.811	
Previous coronary/LEAD intervention	1	0.17152	0.31212	0.3020	0.5826	1.187	0.644	2.189	
History of ischemic stroke/ TIA	1	0.40944	0.37662	1.1819	0.2770	1.506	0.720	3.151	
Cigarette smoking history	1	0.32034	0.32438	0.9752	0.3234	1.378	0.729	2.602	Cigarette smoking history
fibrate_use	1	-0.08726	0.61433	0.0202	0.8870	0.916	0.275	3.055	
BMI_WHOAsia_D1	1	0.15307	0.37997	0.1623	0.6870	1.165	0.553	2.454	
BMI_WHOAsia_D2	1	-0.14065	0.31631	0.1977	0.6566	0.869	0.467	1.615	
CKD_refgt60_D2	1	0.44338	0.30494	2.1142	0.1459	1.558	0.857	2.832	
CKD_refgt60_D3	1	1.05352	0.43623	5.8324	0.0157	2.868	1.220	6.743	
Not under statin & LDL < 100 mg/dL	1	0.32490	0.31325	1.0758	0.2996	1.384	0.749	2.557	

derive.out_lipid_mi_4099

The PHREG Procedure

Imputation Number=2

Model Information		
Data Set	WORK.OUT_LIPID_MI_4099	
Dependent Variable	Prim_Outc_Time	
Censoring Variable	Prim_Outc	Prim_Outc
Censoring Value(s)	0	
Ties Handling	BRESLOW	

Number of Observations Read	4099
Number of Observations Used	2321

Summary of the Number of Event and Censored Values			
Total	Event	Censored	Percent Censored
2321	56	2265	97.59

Convergence Status
Convergence criterion (GCONV=1E-8) satisfied.

Model Fit Statistics		
Criterion	Without Covariates	With Covariates
-2 LOG L	795.549	763.052
AIC	795.549	793.052
SBC	795.549	823.432

Testing Global Null Hypothesis: BETA=0			
Test	Chi-Square	DF	Pr > ChiSq
Likelihood Ratio	32.4965	15	0.0055
Score	39.9533	15	0.0005
Wald	36.6344	15	0.0014

derive.out_lipid_mi_4099

The PHREG Procedure

Imputation Number=2

Analysis of Maximum Likelihood Estimates									
Parameter	DF	Parameter Estimate	Standard Error	Chi-Square	Pr > ChiSq	Hazard Ratio	95% Hazard Ratio Confidence Limits		Label
Age	1	0.01585	0.01351	1.3760	0.2408	1.016	0.989	1.043	
Male (vs. female)	1	-0.48291	0.34482	1.9614	0.1614	0.617	0.314	1.213	Male (vs. female)
History of HTN	1	0.20496	0.33509	0.3741	0.5408	1.227	0.636	2.367	History of HTN
History of DM	1	0.22827	0.27990	0.6651	0.4148	1.256	0.726	2.175	
History of HF	1	0.95447	0.29961	10.1484	0.0014	2.597	1.444	4.673	History of HF
History of MI	1	0.64356	0.47353	1.8471	0.1741	1.903	0.752	4.815	
Previous coronary/LEAD intervention	1	0.18032	0.31133	0.3355	0.5624	1.198	0.651	2.205	
History of ischemic stroke/ TIA	1	0.36884	0.37707	0.9568	0.3280	1.446	0.691	3.028	
Cigarette smoking history	1	0.33990	0.32282	1.1086	0.2924	1.405	0.746	2.645	Cigarette smoking history
fibrate_use	1	-0.08202	0.61380	0.0179	0.8937	0.921	0.277	3.068	
BMI_WHOAsia_D1	1	-0.07575	0.38400	0.0389	0.8436	0.927	0.437	1.968	
BMI_WHOAsia_D2	1	-0.18381	0.31066	0.3501	0.5541	0.832	0.453	1.530	
CKD_refgt60_D2	1	0.43616	0.30469	2.0492	0.1523	1.547	0.851	2.810	
CKD_refgt60_D3	1	0.98914	0.44043	5.0438	0.0247	2.689	1.134	6.375	
Not under statin & LDL < 100 mg/dL	1	0.36608	0.31349	1.3637	0.2429	1.442	0.780	2.666	

derive.out_lipid_mi_4099

The PHREG Procedure

Imputation Number=3

Model Information		
Data Set	WORK.OUT_LIPID_MI_4099	
Dependent Variable	Prim_Outc_Time	
Censoring Variable	Prim_Outc	Prim_Outc
Censoring Value(s)	0	
Ties Handling	BRESLOW	

Number of Observations Read	4099
Number of Observations Used	2327

Summary of the Number of Event and Censored Values			
Total	Event	Censored	Percent Censored
2327	56	2271	97.59

Convergence Status
Convergence criterion (GCONV=1E-8) satisfied.

Model Fit Statistics		
Criterion	Without Covariates	With Covariates
-2 LOG L	795.795	764.178
AIC	795.795	794.178
SBC	795.795	824.558

Testing Global Null Hypothesis: BETA=0			
Test	Chi-Square	DF	Pr > ChiSq
Likelihood Ratio	31.6171	15	0.0073
Score	39.2965	15	0.0006
Wald	35.8722	15	0.0018

derive.out_lipid_mi_4099

The PHREG Procedure

Imputation Number=3

Analysis of Maximum Likelihood Estimates									
Parameter	DF	Parameter Estimate	Standard Error	Chi-Square	Pr > ChiSq	Hazard Ratio	95% Hazard Ratio Confidence Limits		Label
Age	1	0.01535	0.01351	1.2920	0.2557	1.015	0.989	1.043	
Male (vs. female)	1	-0.45897	0.34503	1.7695	0.1834	0.632	0.321	1.243	Male (vs. female)
History of HTN	1	0.18531	0.33494	0.3061	0.5801	1.204	0.624	2.320	History of HTN
History of DM	1	0.22030	0.28020	0.6181	0.4317	1.246	0.720	2.159	
History of HF	1	0.92103	0.30054	9.3916	0.0022	2.512	1.394	4.527	History of HF
History of MI	1	0.65787	0.47280	1.9360	0.1641	1.931	0.764	4.877	
Previous coronary/LEAD intervention	1	0.16095	0.30851	0.2722	0.6019	1.175	0.642	2.150	
History of ischemic stroke/ TIA	1	0.39896	0.37643	1.1233	0.2892	1.490	0.713	3.117	
Cigarette smoking history	1	0.35603	0.32310	1.2142	0.2705	1.428	0.758	2.689	Cigarette smoking history
fibrate_use	1	-0.02823	0.61440	0.0021	0.9633	0.972	0.292	3.241	
BMI_WHOAsia_D1	1	0.01796	0.38802	0.0021	0.9631	1.018	0.476	2.178	
BMI_WHOAsia_D2	1	-0.10360	0.31187	0.1103	0.7398	0.902	0.489	1.661	
CKD_refgt60_D2	1	0.41005	0.30712	1.7826	0.1818	1.507	0.825	2.751	
CKD_refgt60_D3	1	1.04378	0.44301	5.5512	0.0185	2.840	1.192	6.767	
Not under statin & LDL < 100 mg/dL	1	0.33294	0.31248	1.1353	0.2866	1.395	0.756	2.574	

derive.out_lipid_mi_4099

The PHREG Procedure

Imputation Number=4

Model Information		
Data Set	WORK.OUT_LIPID_MI_4099	
Dependent Variable	Prim_Outc_Time	
Censoring Variable	Prim_Outc	Prim_Outc
Censoring Value(s)	0	
Ties Handling	BRESLOW	

Number of Observations Read	4099
Number of Observations Used	2325

Summary of the Number of Event and Censored Values			
Total	Event	Censored	Percent Censored
2325	56	2269	97.59

Convergence Status
Convergence criterion (GCONV=1E-8) satisfied.

Model Fit Statistics		
Criterion	Without Covariates	With Covariates
-2 LOG L	796.263	764.097
AIC	796.263	794.097
SBC	796.263	824.478

Testing Global Null Hypothesis: BETA=0			
Test	Chi-Square	DF	Pr > ChiSq
Likelihood Ratio	32.1653	15	0.0061
Score	39.5006	15	0.0005
Wald	36.0845	15	0.0017

derive.out_lipid_mi_4099

The PHREG Procedure

Imputation Number=4

Analysis of Maximum Likelihood Estimates									
Parameter	DF	Parameter Estimate	Standard Error	Chi-Square	Pr > ChiSq	Hazard Ratio	95% Hazard Ratio Confidence Limits		Label
Age	1	0.01535	0.01357	1.2788	0.2581	1.015	0.989	1.043	
Male (vs. female)	1	-0.44651	0.34683	1.6575	0.1979	0.640	0.324	1.263	Male (vs. female)
History of HTN	1	0.21812	0.33553	0.4226	0.5156	1.244	0.644	2.401	History of HTN
History of DM	1	0.22466	0.28036	0.6421	0.4229	1.252	0.723	2.169	
History of HF	1	0.88732	0.30039	8.7254	0.0031	2.429	1.348	4.376	History of HF
History of MI	1	0.62068	0.47464	1.7100	0.1910	1.860	0.734	4.716	
Previous coronary/LEAD intervention	1	0.17176	0.31101	0.3050	0.5808	1.187	0.645	2.184	
History of ischemic stroke/ TIA	1	0.36150	0.37603	0.9243	0.3364	1.435	0.687	3.000	
Cigarette smoking history	1	0.32120	0.32331	0.9870	0.3205	1.379	0.732	2.598	Cigarette smoking history
fibrate_use	1	-0.06441	0.61223	0.0111	0.9162	0.938	0.282	3.113	
BMI_WHOAsia_D1	1	0.11845	0.38080	0.0968	0.7558	1.126	0.534	2.375	
BMI_WHOAsia_D2	1	-0.15027	0.31542	0.2270	0.6338	0.860	0.464	1.597	
CKD_refgt60_D2	1	0.45039	0.30479	2.1836	0.1395	1.569	0.863	2.851	
CKD_refgt60_D3	1	1.03529	0.44141	5.5009	0.0190	2.816	1.185	6.689	
Not under statin & LDL < 100 mg/dL	1	0.36937	0.31424	1.3816	0.2398	1.447	0.782	2.679	

derive.out_lipid_mi_4099

The PHREG Procedure

Imputation Number=5

Model Information		
Data Set	WORK.OUT_LIPID_MI_4099	
Dependent Variable	Prim_Outc_Time	
Censoring Variable	Prim_Outc	Prim_Outc
Censoring Value(s)	0	
Ties Handling	BRESLOW	

Number of Observations Read	4099
Number of Observations Used	2301

Summary of the Number of Event and Censored Values			
Total	Event	Censored	Percent Censored
2301	56	2245	97.57

Convergence Status
Convergence criterion (GCONV=1E-8) satisfied.

Model Fit Statistics		
Criterion	Without Covariates	With Covariates
-2 LOG L	794.700	762.990
AIC	794.700	792.990
SBC	794.700	823.370

Testing Global Null Hypothesis: BETA=0			
Test	Chi-Square	DF	Pr > ChiSq
Likelihood Ratio	31.7104	15	0.0070
Score	38.4968	15	0.0008
Wald	35.4599	15	0.0021

derive.out_lipid_mi_4099

The PHREG Procedure

Imputation Number=5

Analysis of Maximum Likelihood Estimates									
Parameter	DF	Parameter Estimate	Standard Error	Chi-Square	Pr > ChiSq	Hazard Ratio	95% Hazard Ratio Confidence Limits		Label
Age	1	0.01691	0.01342	1.5882	0.2076	1.017	0.991	1.044	
Male (vs. female)	1	-0.45072	0.34482	1.7086	0.1912	0.637	0.324	1.252	Male (vs. female)
History of HTN	1	0.18929	0.33473	0.3198	0.5717	1.208	0.627	2.329	History of HTN
History of DM	1	0.24821	0.27876	0.7928	0.3732	1.282	0.742	2.213	
History of HF	1	0.89535	0.30003	8.9053	0.0028	2.448	1.360	4.408	History of HF
History of MI	1	0.65950	0.47127	1.9583	0.1617	1.934	0.768	4.870	
Previous coronary/LEAD intervention	1	0.12866	0.30777	0.1748	0.6759	1.137	0.622	2.079	
History of ischemic stroke/ TIA	1	0.39461	0.37509	1.1068	0.2928	1.484	0.711	3.095	
Cigarette smoking history	1	0.34658	0.32159	1.1614	0.2812	1.414	0.753	2.656	Cigarette smoking history
fibrate_use	1	-0.04178	0.61237	0.0047	0.9456	0.959	0.289	3.185	
BMI_WHOAsia_D1	1	0.06449	0.38926	0.0274	0.8684	1.067	0.497	2.287	
BMI_WHOAsia_D2	1	-0.09632	0.31265	0.0949	0.7580	0.908	0.492	1.676	
CKD_refgt60_D2	1	0.44905	0.30369	2.1864	0.1392	1.567	0.864	2.841	
CKD_refgt60_D3	1	0.96724	0.44021	4.8278	0.0280	2.631	1.110	6.234	
Not under statin & LDL < 100 mg/dL	1	0.36438	0.31230	1.3613	0.2433	1.440	0.781	2.655	

phregparms_lipid_mi_4099

The MIANALYZE Procedure

Model Information	
PARMS Data Set	WORK.PHREGPARMS_LIPID_MI_4099
Number of Imputations	5

Variance Information							
Parameter	Variance			DF	Relative Increase in Variance	Fraction Missing Information	Relative Efficiency
	Between	Within	Total				
age	0.000000681	0.000182	0.000183	201109	0.004480	0.004470	0.999107
MALE (VS. FEMALE)	0.000199	0.119343	0.119581	1.01E6	0.001997	0.001995	0.999601
History of HTN	0.000180	0.112183	0.112399	1.09E6	0.001924	0.001922	0.999616
History of DM	0.000199	0.078217	0.078456	432569	0.003050	0.003046	0.999391
History of HF	0.000753	0.090016	0.090920	40469	0.010042	0.009991	0.998006
History of MI	0.000243	0.223408	0.223700	2.35E6	0.001306	0.001305	0.999739
Previous coronary/LEAD intervention	0.000408	0.096194	0.096683	155904	0.005091	0.005078	0.998985
History of ischemic stroke/ TIA	0.000421	0.141561	0.142066	316318	0.003569	0.003562	0.999288
Cigarette smoking history	0.000247	0.104356	0.104653	497380	0.002844	0.002840	0.999432
fibrate_use	0.000646	0.376294	0.377069	945038	0.002062	0.002059	0.999588
BMI_WHOAsia_D1	0.008050	0.147784	0.157444	1062.5	0.065368	0.063119	0.987533
BMI_WHOAsia_D2	0.001283	0.098213	0.099752	16802	0.015671	0.015546	0.996900
CKD_refgt60_D2	0.000272	0.093054	0.093381	326840	0.003511	0.003504	0.999300
CKD_refgt60_D3	0.001409	0.193834	0.195525	53515	0.008721	0.008683	0.998266
Not under statin & LDL < 100 mg/dL	0.000437	0.098064	0.098588	141158	0.005352	0.005337	0.998934

Parameter Estimates							
Parameter	Estimate	Std Error	95% Confidence Limits		DF	Minimum	Maximum
age	0.015631	0.013536	-0.01090	0.042162	201109	0.014695	0.016913
MALE (VS. FEMALE)	-0.459952	0.345806	-1.13772	0.217815	1.01E6	-0.482911	-0.446512
History of HTN	0.200785	0.335259	-0.45631	0.857881	1.09E6	0.185315	0.218121

phregparms_lipid_mi_4099

The MIANALYZE Procedure

Parameter Estimates							
Parameter	Estimate	Std Error	95% Confidence Limits		DF	Minimum	Maximum
History of DM	0.234473	0.280100	-0.31451	0.783460	432569	0.220295	0.250931
History of HF	0.918203	0.301529	0.32720	1.509208	40469	0.887321	0.954466
History of MI	0.645867	0.472969	-0.28114	1.572870	2.35E6	0.620684	0.659498
Previous coronary/LEAD intervention	0.162642	0.310940	-0.44679	0.772077	155904	0.128658	0.180324
History of ischemic stroke/ TIA	0.386670	0.376917	-0.35208	1.125417	316318	0.361505	0.409441
Cigarette smoking history	0.336808	0.323501	-0.29724	0.970860	497380	0.320335	0.356026
fibrate_use	-0.060743	0.614060	-1.26428	1.142794	945038	-0.087263	-0.028234
BMI_WHOAsia_D1	0.055643	0.396792	-0.72294	0.834228	1062.5	-0.075754	0.153075
BMI_WHOAsia_D2	-0.134929	0.315836	-0.75400	0.484142	16802	-0.183813	-0.096317
CKD_refgt60_D2	0.437806	0.305582	-0.16113	1.036739	326840	0.410050	0.450390
CKD_refgt60_D3	1.017793	0.442182	0.15111	1.884473	53515	0.967235	1.053515
Not under statin & LDL < 100 mg/dL	0.351535	0.313988	-0.26388	0.966945	141158	0.324904	0.369366

Parameter Estimates			
Parameter	Theta0	t for H0: Parameter=Theta0	Pr > |t|
age	0	1.15	0.2482
MALE (VS. FEMALE)	0	-1.33	0.1835
History of HTN	0	0.60	0.5492
History of DM	0	0.84	0.4025
History of HF	0	3.05	0.0023
History of MI	0	1.37	0.1721
Previous coronary/LEAD intervention	0	0.52	0.6009
History of ischemic stroke/ TIA	0	1.03	0.3050
Cigarette smoking history	0	1.04	0.2978
fibrate_use	0	-0.10	0.9212

phregparms_lipid_mi_4099

The MIANALYZE Procedure

Parameter Estimates			
Parameter	Theta0	t for H0: Parameter=Theta0	Pr > |t|
BMI_WHOAsia_D1	0	0.14	0.8885
BMI_WHOAsia_D2	0	-0.43	0.6692
CKD_refgt60_D2	0	1.43	0.1519
CKD_refgt60_D3	0	2.30	0.0214
Not under statin & LDL < 100 mg/dL	0	1.12	0.2629

phregparms_mianalyze_mi_4099

Obs	Parm	Estimate	EST_exp	LCLMean_exp	UCLMean_exp	Probt
1	age	0.015631	1.01575	0.98916	1.04306	0.2482
2	MALE (VS. FEMALE)	-0.459952	0.63131	0.32055	1.24336	0.1835
3	History of HTN	0.200785	1.22236	0.63362	2.35816	0.5492
4	History of DM	0.234473	1.26424	0.73014	2.18903	0.4025
5	History of HF	0.918203	2.50479	1.38708	4.52315	0.0023
6	History of MI	0.645867	1.90764	0.75493	4.82046	0.1721
7	Previous coronary/LEAD intervention	0.162642	1.17662	0.63968	2.16426	0.6009
8	History of ischemic stroke/ TIA	0.386670	1.47207	0.70323	3.08150	0.3050
9	Cigarette smoking history	0.336808	1.40047	0.74286	2.64021	0.2978
10	fibrate_use	-0.060743	0.94107	0.28244	3.13552	0.9212
11	BMI_WHOAsia_D1	0.055643	1.05722	0.48532	2.30303	0.8885
12	BMI_WHOAsia_D2	-0.134929	0.87378	0.47048	1.62278	0.6692
13	CKD_refgt60_D2	0.437806	1.54930	0.85118	2.82001	0.1519
14	CKD_refgt60_D3	1.017793	2.76708	1.16313	6.58289	0.0214
15	Not under statin & LDL < 100 mg/dL	0.351535	1.42125	0.76807	2.62990	0.2629

derive.out_lipid_mi_4099

The PHREG Procedure

Imputation Number=1

Model Information		
Data Set	WORK.OUT_LIPID_MI_4099	
Dependent Variable	Prim_Outc_Time	
Censoring Variable	Prim_Outc	Prim_Outc
Censoring Value(s)	0	
Ties Handling	BRESLOW	

Number of Observations Read	4099
Number of Observations Used	2321

Summary of the Number of Event and Censored Values			
Total	Event	Censored	Percent Censored
2321	54	2267	97.67

Convergence Status
Convergence criterion (GCONV=1E-8) satisfied.

Model Fit Statistics		
Criterion	Without Covariates	With Covariates
-2 LOG L	773.954	732.347
AIC	773.954	762.347
SBC	773.954	792.182

Testing Global Null Hypothesis: BETA=0			
Test	Chi-Square	DF	Pr > ChiSq
Likelihood Ratio	41.6068	15	0.0003
Score	44.2322	15	0.0001
Wald	41.2909	15	0.0003

derive.out_lipid_mi_4099

The PHREG Procedure

Imputation Number=1

Analysis of Maximum Likelihood Estimates									
Parameter	DF	Parameter Estimate	Standard Error	Chi-Square	Pr > ChiSq	Hazard Ratio	95% Hazard Ratio Confidence Limits		Label
Age	1	0.00199	0.01365	0.0213	0.8840	1.002	0.976	1.029	
Male (vs. female)	1	-0.62623	0.37210	2.8324	0.0924	0.535	0.258	1.109	Male (vs. female)
History of HTN	1	0.59880	0.37419	2.5608	0.1095	1.820	0.874	3.789	History of HTN
History of DM	1	0.70329	0.28844	5.9450	0.0148	2.020	1.148	3.556	
History of HF	1	0.76213	0.32681	5.4384	0.0197	2.143	1.129	4.066	History of HF
History of MI	1	0.86998	0.55023	2.4999	0.1139	2.387	0.812	7.018	
Previous coronary/LEAD intervention	1	0.39103	0.33558	1.3578	0.2439	1.479	0.766	2.854	
History of ischemic stroke/ TIA	1	-0.24419	0.46078	0.2809	0.5961	0.783	0.317	1.933	
Cigarette smoking history	1	0.58432	0.34620	2.8487	0.0914	1.794	0.910	3.536	Cigarette smoking history
fibrate_use	1	-0.55441	0.73820	0.5640	0.4526	0.574	0.135	2.441	
BMI_WHOAsia_D1	1	0.28590	0.37910	0.5687	0.4508	1.331	0.633	2.798	
BMI_WHOAsia_D2	1	-0.14120	0.31994	0.1948	0.6590	0.868	0.464	1.626	
CKD_refgt60_D2	1	0.64068	0.30720	4.3496	0.0370	1.898	1.039	3.465	
CKD_refgt60_D3	1	0.71574	0.50464	2.0117	0.1561	2.046	0.761	5.500	
Not under statin & LDL ≥ 100 mg/dL	1	0.72458	0.33668	4.6317	0.0314	2.064	1.067	3.993	

derive.out_lipid_mi_4099

The PHREG Procedure

Imputation Number=2

Model Information		
Data Set	WORK.OUT_LIPID_MI_4099	
Dependent Variable	Prim_Outc_Time	
Censoring Variable	Prim_Outc	Prim_Outc
Censoring Value(s)	0	
Ties Handling	BRESLOW	

Number of Observations Read	4099
Number of Observations Used	2339

Summary of the Number of Event and Censored Values			
Total	Event	Censored	Percent Censored
2339	54	2285	97.69

Convergence Status
Convergence criterion (GCONV=1E-8) satisfied.

Model Fit Statistics		
Criterion	Without Covariates	With Covariates
-2 LOG L	775.189	733.834
AIC	775.189	763.834
SBC	775.189	793.669

Testing Global Null Hypothesis: BETA=0			
Test	Chi-Square	DF	Pr > ChiSq
Likelihood Ratio	41.3546	15	0.0003
Score	44.6657	15	<.0001
Wald	41.0690	15	0.0003

derive.out_lipid_mi_4099

The PHREG Procedure

Imputation Number=2

Analysis of Maximum Likelihood Estimates									
Parameter	DF	Parameter Estimate	Standard Error	Chi-Square	Pr > ChiSq	Hazard Ratio	95% Hazard Ratio Confidence Limits		Label
Age	1	0.0001725	0.01340	0.0002	0.9897	1.000	0.974	1.027	
Male (vs. female)	1	-0.60810	0.37672	2.6056	0.1065	0.544	0.260	1.139	Male (vs. female)
History of HTN	1	0.58653	0.37232	2.4817	0.1152	1.798	0.867	3.729	History of HTN
History of DM	1	0.69824	0.28890	5.8414	0.0157	2.010	1.141	3.541	
History of HF	1	0.75487	0.32725	5.3209	0.0211	2.127	1.120	4.040	History of HF
History of MI	1	0.82863	0.54945	2.2743	0.1315	2.290	0.780	6.723	
Previous coronary/LEAD intervention	1	0.35773	0.33325	1.1523	0.2831	1.430	0.744	2.748	
History of ischemic stroke/ TIA	1	-0.24224	0.45612	0.2821	0.5954	0.785	0.321	1.919	
Cigarette smoking history	1	0.56090	0.35097	2.5541	0.1100	1.752	0.881	3.486	Cigarette smoking history
fibrate_use	1	-0.63112	0.73779	0.7317	0.3923	0.532	0.125	2.259	
BMI_WHOAsia_D1	1	0.33559	0.37722	0.7915	0.3737	1.399	0.668	2.930	
BMI_WHOAsia_D2	1	-0.08861	0.31893	0.0772	0.7811	0.915	0.490	1.710	
CKD_refgt60_D2	1	0.69698	0.30434	5.2446	0.0220	2.008	1.106	3.645	
CKD_refgt60_D3	1	0.69119	0.50660	1.8615	0.1724	1.996	0.740	5.388	
Not under statin & LDL ≥ 100 mg/dL	1	0.68350	0.33291	4.2153	0.0401	1.981	1.031	3.804	

derive.out_lipid_mi_4099

The PHREG Procedure

Imputation Number=3

Model Information		
Data Set	WORK.OUT_LIPID_MI_4099	
Dependent Variable	Prim_Outc_Time	
Censoring Variable	Prim_Outc	Prim_Outc
Censoring Value(s)	0	
Ties Handling	BRESLOW	

Number of Observations Read	4099
Number of Observations Used	2333

Summary of the Number of Event and Censored Values			
Total	Event	Censored	Percent Censored
2333	54	2279	97.69

Convergence Status
Convergence criterion (GCONV=1E-8) satisfied.

Model Fit Statistics		
Criterion	Without Covariates	With Covariates
-2 LOG L	774.402	732.841
AIC	774.402	762.841
SBC	774.402	792.676

Testing Global Null Hypothesis: BETA=0			
Test	Chi-Square	DF	Pr > ChiSq
Likelihood Ratio	41.5610	15	0.0003
Score	44.3316	15	<.0001
Wald	41.1627	15	0.0003

derive.out_lipid_mi_4099

The PHREG Procedure

Imputation Number=3

Analysis of Maximum Likelihood Estimates									
Parameter	DF	Parameter Estimate	Standard Error	Chi-Square	Pr > ChiSq	Hazard Ratio	95% Hazard Ratio Confidence Limits		Label
Age	1	0.00206	0.01375	0.0225	0.8808	1.002	0.975	1.029	
Male (vs. female)	1	-0.63743	0.37217	2.9335	0.0868	0.529	0.255	1.096	Male (vs. female)
History of HTN	1	0.57479	0.37293	2.3755	0.1233	1.777	0.855	3.690	History of HTN
History of DM	1	0.69319	0.28834	5.7796	0.0162	2.000	1.137	3.520	
History of HF	1	0.73370	0.32689	5.0378	0.0248	2.083	1.097	3.953	History of HF
History of MI	1	0.85604	0.54897	2.4316	0.1189	2.354	0.803	6.903	
Previous coronary/LEAD intervention	1	0.40003	0.33463	1.4290	0.2319	1.492	0.774	2.875	
History of ischemic stroke/ TIA	1	-0.24257	0.45861	0.2798	0.5969	0.785	0.319	1.928	
Cigarette smoking history	1	0.60036	0.34566	3.0167	0.0824	1.823	0.926	3.589	Cigarette smoking history
fibrate_use	1	-0.56464	0.73881	0.5841	0.4447	0.569	0.134	2.419	
BMI_WHOAsia_D1	1	0.29054	0.37936	0.5865	0.4438	1.337	0.636	2.813	
BMI_WHOAsia_D2	1	-0.20403	0.31870	0.4099	0.5220	0.815	0.437	1.523	
CKD_refgt60_D2	1	0.64157	0.30778	4.3451	0.0371	1.899	1.039	3.472	
CKD_refgt60_D3	1	0.65337	0.51001	1.6412	0.2002	1.922	0.707	5.222	
Not under statin & LDL ≥ 100 mg/dL	1	0.73071	0.33458	4.7697	0.0290	2.077	1.078	4.001	

derive.out_lipid_mi_4099

The PHREG Procedure

Imputation Number=4

Model Information		
Data Set	WORK.OUT_LIPID_MI_4099	
Dependent Variable	Prim_Outc_Time	
Censoring Variable	Prim_Outc	Prim_Outc
Censoring Value(s)	0	
Ties Handling	BRESLOW	

Number of Observations Read	4099
Number of Observations Used	2363

Summary of the Number of Event and Censored Values			
Total	Event	Censored	Percent Censored
2363	54	2309	97.71

Convergence Status
Convergence criterion (GCONV=1E-8) satisfied.

Model Fit Statistics		
Criterion	Without Covariates	With Covariates
-2 LOG L	775.847	733.887
AIC	775.847	763.887
SBC	775.847	793.722

Testing Global Null Hypothesis: BETA=0			
Test	Chi-Square	DF	Pr > ChiSq
Likelihood Ratio	41.9599	15	0.0002
Score	45.0926	15	<.0001
Wald	41.8052	15	0.0002

derive.out_lipid_mi_4099

The PHREG Procedure

Imputation Number=4

Analysis of Maximum Likelihood Estimates									
Parameter	DF	Parameter Estimate	Standard Error	Chi-Square	Pr > ChiSq	Hazard Ratio	95% Hazard Ratio Confidence Limits		Label
Age	1	0.00169	0.01351	0.0156	0.9007	1.002	0.976	1.029	
Male (vs. female)	1	-0.59612	0.37066	2.5865	0.1078	0.551	0.266	1.139	Male (vs. female)
History of HTN	1	0.64979	0.37225	3.0471	0.0809	1.915	0.923	3.972	History of HTN
History of DM	1	0.67838	0.28861	5.5248	0.0187	1.971	1.119	3.470	
History of HF	1	0.76158	0.32740	5.4108	0.0200	2.142	1.127	4.069	History of HF
History of MI	1	0.81338	0.54663	2.2141	0.1368	2.256	0.773	6.585	
Previous coronary/LEAD intervention	1	0.38000	0.33416	1.2932	0.2555	1.462	0.760	2.815	
History of ischemic stroke/ TIA	1	-0.29409	0.45471	0.4183	0.5178	0.745	0.306	1.817	
Cigarette smoking history	1	0.58927	0.34512	2.9154	0.0877	1.803	0.917	3.546	Cigarette smoking history
fibrate_use	1	-0.59197	0.74212	0.6363	0.4251	0.553	0.129	2.369	
BMI_WHOAsia_D1	1	0.33099	0.37921	0.7618	0.3828	1.392	0.662	2.928	
BMI_WHOAsia_D2	1	-0.13808	0.31914	0.1872	0.6652	0.871	0.466	1.628	
CKD_refgt60_D2	1	0.65634	0.30569	4.6098	0.0318	1.928	1.059	3.510	
CKD_refgt60_D3	1	0.75224	0.50946	2.1802	0.1398	2.122	0.782	5.759	
Not under statin & LDL ≥ 100 mg/dL	1	0.71673	0.33567	4.5591	0.0327	2.048	1.061	3.954	

derive.out_lipid_mi_4099

The PHREG Procedure

Imputation Number=5

Model Information		
Data Set	WORK.OUT_LIPID_MI_4099	
Dependent Variable	Prim_Outc_Time	
Censoring Variable	Prim_Outc	Prim_Outc
Censoring Value(s)	0	
Ties Handling	BRESLOW	

Number of Observations Read	4099
Number of Observations Used	2351

Summary of the Number of Event and Censored Values			
Total	Event	Censored	Percent Censored
2351	54	2297	97.70

Convergence Status
Convergence criterion (GCONV=1E-8) satisfied.

Model Fit Statistics		
Criterion	Without Covariates	With Covariates
-2 LOG L	775.530	733.121
AIC	775.530	763.121
SBC	775.530	792.956

Testing Global Null Hypothesis: BETA=0			
Test	Chi-Square	DF	Pr > ChiSq
Likelihood Ratio	42.4087	15	0.0002
Score	45.3723	15	<.0001
Wald	42.0188	15	0.0002

derive.out_lipid_mi_4099

The PHREG Procedure

Imputation Number=5

Analysis of Maximum Likelihood Estimates									
Parameter	DF	Parameter Estimate	Standard Error	Chi-Square	Pr > ChiSq	Hazard Ratio	95% Hazard Ratio Confidence Limits		Label
Age	1	0.00197	0.01365	0.0208	0.8854	1.002	0.976	1.029	
Male (vs. female)	1	-0.57434	0.37196	2.3843	0.1226	0.563	0.272	1.167	Male (vs. female)
History of HTN	1	0.59186	0.37276	2.5210	0.1123	1.807	0.870	3.753	History of HTN
History of DM	1	0.75366	0.28763	6.8656	0.0088	2.125	1.209	3.734	
History of HF	1	0.77929	0.32679	5.6868	0.0171	2.180	1.149	4.136	History of HF
History of MI	1	0.86568	0.54614	2.5125	0.1129	2.377	0.815	6.932	
Previous coronary/LEAD intervention	1	0.37315	0.33512	1.2398	0.2655	1.452	0.753	2.801	
History of ischemic stroke/ TIA	1	-0.32047	0.45593	0.4941	0.4821	0.726	0.297	1.774	
Cigarette smoking history	1	0.56669	0.34643	2.6759	0.1019	1.762	0.894	3.475	Cigarette smoking history
fibrate_use	1	-0.63194	0.74110	0.7271	0.3938	0.532	0.124	2.272	
BMI_WHOAsia_D1	1	0.33137	0.37855	0.7663	0.3814	1.393	0.663	2.925	
BMI_WHOAsia_D2	1	-0.14456	0.31986	0.2043	0.6513	0.865	0.462	1.620	
CKD_refgt60_D2	1	0.63764	0.30632	4.3330	0.0374	1.892	1.038	3.449	
CKD_refgt60_D3	1	0.64737	0.50584	1.6379	0.2006	1.911	0.709	5.149	
Not under statin & LDL ≥ 100 mg/dL	1	0.71362	0.33702	4.4834	0.0342	2.041	1.054	3.952	

phregparms_lipid_mi_4099

The MIANALYZE Procedure

Model Information	
PARMS Data Set	WORK.PHREGPARMS_LIPID_MI_4099
Number of Imputations	5

Variance Information							
Parameter	Variance						
	Between	Within	Total	DF	Relative Increase in Variance	Fraction Missing Information	Relative Efficiency
age	0.000000636	0.000185	0.000186	236338	0.004131	0.004122	0.999176
MALE (VS. FEMALE)	0.000618	0.138926	0.139667	141910	0.005337	0.005323	0.998937
History of HTN	0.000841	0.139048	0.140057	77072	0.007256	0.007230	0.998556
History of DM	0.000816	0.083167	0.084146	29536	0.011774	0.011704	0.997665
History of HF	0.000271	0.106946	0.107271	436264	0.003037	0.003033	0.999394
History of MI	0.000607	0.300619	0.301347	685575	0.002421	0.002418	0.999517
Previous coronary/LEAD intervention	0.000266	0.111924	0.112244	493683	0.002855	0.002851	0.999430
History of ischemic stroke/ TIA	0.001327	0.209065	0.210657	69994	0.007617	0.007588	0.998485
Cigarette smoking history	0.000265	0.120325	0.120644	574769	0.002645	0.002642	0.999472
fibrate_use	0.001312	0.547018	0.548592	485789	0.002878	0.002874	0.999426
BMI_WHOAsia_D1	0.000598	0.143406	0.144124	161212	0.005006	0.004994	0.999002
BMI_WHOAsia_D2	0.001678	0.101961	0.103974	10663	0.019750	0.019552	0.996105
CKD_refgt60_D2	0.000613	0.093801	0.094536	66168	0.007836	0.007805	0.998441
CKD_refgt60_D3	0.001919	0.257365	0.259668	50838	0.008950	0.008909	0.998221
Not under statin & LDL ≥ 100 mg/dL	0.000332	0.112477	0.112875	320693	0.003544	0.003538	0.999293

phregparms_lipid_mi_4099

The MIANALYZE Procedure

Parameter Estimates							
Parameter	Estimate	Std Error	95% Confidence Limits		DF	Minimum	Maximum
age	0.001576	0.013620	-0.02512	0.028272	236338	0.000172	0.002062
MALE (VS. FEMALE)	-0.608443	0.373721	-1.34093	0.124043	141910	-0.637429	-0.574339
History of HTN	0.600355	0.374242	-0.13316	1.333868	77072	0.574789	0.649793
History of DM	0.705355	0.290079	0.13679	1.273923	29536	0.678382	0.753664
History of HF	0.758313	0.327523	0.11638	1.400248	436264	0.733703	0.779288
History of MI	0.846741	0.548951	-0.22918	1.922666	685575	0.813379	0.869979
Previous coronary/LEAD intervention	0.380388	0.335028	-0.27626	1.037033	493683	0.357730	0.400027
History of ischemic stroke/ TIA	-0.268712	0.458974	-1.16830	0.630876	69994	-0.320472	-0.242237
Cigarette smoking history	0.580307	0.347338	-0.10046	1.261078	574769	0.560897	0.600362
fibrate_use	-0.594816	0.740670	-2.04651	0.856875	485789	-0.631941	-0.554409
BMI_WHOAsia_D1	0.314878	0.379636	-0.42920	1.058956	161212	0.285898	0.335588
BMI_WHOAsia_D2	-0.143298	0.322450	-0.77536	0.488765	10663	-0.204034	-0.088612
CKD_refgt60_D2	0.654641	0.307467	0.05200	1.257277	66168	0.637640	0.696977
CKD_refgt60_D3	0.691983	0.509577	-0.30679	1.690759	50838	0.647370	0.752244
Not under statin & LDL ≥ 100 mg/dL	0.713827	0.335969	0.05534	1.372317	320693	0.683500	0.730708

phregparms_lipid_mi_4099

The MIANALYZE Procedure

Parameter Estimates			
Parameter	Theta0	t for H0: Parameter=Theta0	Pr > |t|
age	0	0.12	0.9079
MALE (VS. FEMALE)	0	-1.63	0.1035
History of HTN	0	1.60	0.1087
History of DM	0	2.43	0.0150
History of HF	0	2.32	0.0206
History of MI	0	1.54	0.1230
Previous coronary/LEAD intervention	0	1.14	0.2562
History of ischemic stroke/ TIA	0	-0.59	0.5582
Cigarette smoking history	0	1.67	0.0948
fibrate_use	0	-0.80	0.4219
BMI_WHOAsia_D1	0	0.83	0.4069
BMI_WHOAsia_D2	0	-0.44	0.6568
CKD_refgt60_D2	0	2.13	0.0332
CKD_refgt60_D3	0	1.36	0.1745
Not under statin & LDL ≥ 100 mg/dL	0	2.12	0.0336

phregparms_mianalyze_mi_4099

Obs	Parm	Estimate	EST_exp	LCLMean_exp	UCLMean_exp	Probt
1	age	0.001576	1.00158	0.97519	1.02867	0.9079
2	MALE (VS. FEMALE)	-0.608443	0.54420	0.26160	1.13206	0.1035
3	History of HTN	0.600355	1.82277	0.87533	3.79570	0.1087
4	History of DM	0.705355	2.02457	1.14658	3.57485	0.0150
5	History of HF	0.758313	2.13467	1.12342	4.05621	0.0206
6	History of MI	0.846741	2.33203	0.79518	6.83917	0.1230
7	Previous coronary/LEAD intervention	0.380388	1.46285	0.75862	2.82083	0.2562
8	History of ischemic stroke/ TIA	-0.268712	0.76436	0.31089	1.87926	0.5582
9	Cigarette smoking history	0.580307	1.78659	0.90442	3.52923	0.0948
10	fibrate_use	-0.594816	0.55166	0.12919	2.35579	0.4219
11	BMI_WHOAsia_D1	0.314878	1.37009	0.65103	2.88336	0.4069
12	BMI_WHOAsia_D2	-0.143298	0.86650	0.46054	1.63030	0.6568
13	CKD_refgt60_D2	0.654641	1.92445	1.05338	3.51583	0.0332
14	CKD_refgt60_D3	0.691983	1.99767	0.73580	5.42359	0.1745
15	Not under statin & LDL ≥ 100 mg/dL	0.713827	2.04179	1.05690	3.94448	0.0336

derive.out_lipid_mi_4099

The PHREG Procedure

Imputation Number=1

Model Information		
Data Set	WORK.OUT_LIPID_MI_4099	
Dependent Variable	Prim_Outc_Time	
Censoring Variable	Prim_Outc	Prim_Outc
Censoring Value(s)	0	
Ties Handling	BRESLOW	

Number of Observations Read	4099
Number of Observations Used	2933

Summary of the Number of Event and Censored Values			
Total	Event	Censored	Percent Censored
2933	75	2858	97.44

Convergence Status
Convergence criterion (GCONV=1E-8) satisfied.

Model Fit Statistics		
Criterion	Without Covariates	With Covariates
-2 LOG L	1108.947	1054.701
AIC	1108.947	1084.701
SBC	1108.947	1119.463

Testing Global Null Hypothesis: BETA=0			
Test	Chi-Square	DF	Pr > ChiSq
Likelihood Ratio	54.2461	15	<.0001
Score	61.0124	15	<.0001
Wald	53.6492	15	<.0001

derive.out_lipid_mi_4099

The PHREG Procedure

Imputation Number=1

Analysis of Maximum Likelihood Estimates									
Parameter	DF	Parameter Estimate	Standard Error	Chi-Square	Pr > ChiSq	Hazard Ratio	95% Hazard Ratio Confidence Limits		Label
Age	1	0.01648	0.01173	1.9735	0.1601	1.017	0.994	1.040	
Male (vs. female)	1	-0.34918	0.32575	1.1490	0.2837	0.705	0.372	1.335	Male (vs. female)
History of HTN	1	0.25005	0.28624	0.7631	0.3823	1.284	0.733	2.250	History of HTN
History of DM	1	0.56148	0.24181	5.3917	0.0202	1.753	1.091	2.816	
History of HF	1	0.78016	0.28064	7.7278	0.0054	2.182	1.259	3.782	History of HF
History of MI	1	0.47159	0.47556	0.9834	0.3214	1.603	0.631	4.070	
Previous coronary/LEAD intervention	1	0.11794	0.27133	0.1889	0.6638	1.125	0.661	1.915	
History of ischemic stroke/ TIA	1	-0.14063	0.37400	0.1414	0.7069	0.869	0.417	1.808	
Cigarette smoking history	1	0.63618	0.29570	4.6287	0.0314	1.889	1.058	3.373	Cigarette smoking history
fibrate_use	1	-13.19972	521.02596	0.0006	0.9798	0.000	0.000	.	
BMI_WHOAsia_D1	1	0.28759	0.32637	0.7764	0.3782	1.333	0.703	2.528	
BMI_WHOAsia_D2	1	-0.10562	0.27472	0.1478	0.7006	0.900	0.525	1.542	
CKD_refgt60_D2	1	0.63449	0.26336	5.8041	0.0160	1.886	1.126	3.160	
CKD_refgt60_D3	1	1.08498	0.37030	8.5849	0.0034	2.959	1.432	6.115	
Under statin & LDL ≥ 100 mg/dL	1	0.47926	0.23454	4.1757	0.0410	1.615	1.020	2.557	

derive.out_lipid_mi_4099

The PHREG Procedure

Imputation Number=2

Model Information		
Data Set	WORK.OUT_LIPID_MI_4099	
Dependent Variable	Prim_Outc_Time	
Censoring Variable	Prim_Outc	Prim_Outc
Censoring Value(s)	0	
Ties Handling	BRESLOW	

Number of Observations Read	4099
Number of Observations Used	2933

Summary of the Number of Event and Censored Values			
Total	Event	Censored	Percent Censored
2933	75	2858	97.44

Convergence Status
Convergence criterion (GCONV=1E-8) satisfied.

Model Fit Statistics		
Criterion	Without Covariates	With Covariates
-2 LOG L	1108.947	1053.927
AIC	1108.947	1083.927
SBC	1108.947	1118.690

Testing Global Null Hypothesis: BETA=0			
Test	Chi-Square	DF	Pr > ChiSq
Likelihood Ratio	55.0198	15	<.0001
Score	61.2088	15	<.0001
Wald	53.5942	15	<.0001

derive.out_lipid_mi_4099

The PHREG Procedure

Imputation Number=2

Analysis of Maximum Likelihood Estimates									
Parameter	DF	Parameter Estimate	Standard Error	Chi-Square	Pr > ChiSq	Hazard Ratio	95% Hazard Ratio Confidence Limits		Label
Age	1	0.01812	0.01177	2.3707	0.1236	1.018	0.995	1.042	
Male (vs. female)	1	-0.35860	0.32446	1.2215	0.2691	0.699	0.370	1.320	Male (vs. female)
History of HTN	1	0.24970	0.28549	0.7649	0.3818	1.284	0.734	2.246	History of HTN
History of DM	1	0.56447	0.24072	5.4985	0.0190	1.759	1.097	2.819	
History of HF	1	0.75636	0.28100	7.2452	0.0071	2.131	1.228	3.695	History of HF
History of MI	1	0.51608	0.47721	1.1695	0.2795	1.675	0.658	4.269	
Previous coronary/LEAD intervention	1	0.11317	0.27127	0.1741	0.6765	1.120	0.658	1.906	
History of ischemic stroke/ TIA	1	-0.09596	0.37571	0.0652	0.7984	0.908	0.435	1.897	
Cigarette smoking history	1	0.64063	0.29516	4.7108	0.0300	1.898	1.064	3.384	Cigarette smoking history
fibrate_use	1	-13.20449	510.09338	0.0007	0.9793	0.000	0.000	.	
BMI_WHOAsia_D1	1	0.22518	0.32372	0.4839	0.4867	1.253	0.664	2.362	
BMI_WHOAsia_D2	1	-0.16398	0.27312	0.3605	0.5482	0.849	0.497	1.450	
CKD_refgt60_D2	1	0.64209	0.26169	6.0205	0.0141	1.900	1.138	3.174	
CKD_refgt60_D3	1	1.08620	0.36534	8.8396	0.0029	2.963	1.448	6.063	
Under statin & LDL ≥ 100 mg/dL	1	0.50695	0.23489	4.6581	0.0309	1.660	1.048	2.631	

derive.out_lipid_mi_4099

The PHREG Procedure

Imputation Number=3

Model Information		
Data Set	WORK.OUT_LIPID_MI_4099	
Dependent Variable	Prim_Outc_Time	
Censoring Variable	Prim_Outc	Prim_Outc
Censoring Value(s)	0	
Ties Handling	BRESLOW	

Number of Observations Read	4099
Number of Observations Used	2933

Summary of the Number of Event and Censored Values			
Total	Event	Censored	Percent Censored
2933	75	2858	97.44

Convergence Status
Convergence criterion (GCONV=1E-8) satisfied.

Model Fit Statistics		
Criterion	Without Covariates	With Covariates
-2 LOG L	1108.947	1052.670
AIC	1108.947	1082.670
SBC	1108.947	1117.432

Testing Global Null Hypothesis: BETA=0			
Test	Chi-Square	DF	Pr > ChiSq
Likelihood Ratio	56.2769	15	<.0001
Score	63.5284	15	<.0001
Wald	55.5549	15	<.0001

derive.out_lipid_mi_4099

The PHREG Procedure

Imputation Number=3

Analysis of Maximum Likelihood Estimates									
Parameter	DF	Parameter Estimate	Standard Error	Chi-Square	Pr > ChiSq	Hazard Ratio	95% Hazard Ratio Confidence Limits		Label
Age	1	0.01693	0.01190	2.0256	0.1547	1.017	0.994	1.041	
Male (vs. female)	1	-0.35440	0.32544	1.1859	0.2762	0.702	0.371	1.328	Male (vs. female)
History of HTN	1	0.24021	0.28553	0.7077	0.4002	1.272	0.727	2.225	History of HTN
History of DM	1	0.56815	0.24134	5.5419	0.0186	1.765	1.100	2.833	
History of HF	1	0.76891	0.28048	7.5152	0.0061	2.157	1.245	3.738	History of HF
History of MI	1	0.45612	0.47570	0.9194	0.3376	1.578	0.621	4.009	
Previous coronary/LEAD intervention	1	0.13030	0.27154	0.2303	0.6313	1.139	0.669	1.940	
History of ischemic stroke/ TIA	1	-0.14339	0.37385	0.1471	0.7013	0.866	0.416	1.803	
Cigarette smoking history	1	0.65510	0.29671	4.8747	0.0273	1.925	1.076	3.444	Cigarette smoking history
fibrate_use	1	-13.09142	495.72822	0.0007	0.9789	0.000	0.000	.	
BMI_WHOAsia_D1	1	0.30246	0.32028	0.8919	0.3450	1.353	0.722	2.535	
BMI_WHOAsia_D2	1	-0.18921	0.27493	0.4737	0.4913	0.828	0.483	1.419	
CKD_refgt60_D2	1	0.62889	0.26497	5.6330	0.0176	1.876	1.116	3.153	
CKD_refgt60_D3	1	1.15526	0.36927	9.7873	0.0018	3.175	1.540	6.547	
Under statin & LDL ≥ 100 mg/dL	1	0.50666	0.23472	4.6595	0.0309	1.660	1.048	2.629	

derive.out_lipid_mi_4099

The PHREG Procedure

Imputation Number=4

Model Information		
Data Set	WORK.OUT_LIPID_MI_4099	
Dependent Variable	Prim_Outc_Time	
Censoring Variable	Prim_Outc	Prim_Outc
Censoring Value(s)	0	
Ties Handling	BRESLOW	

Number of Observations Read	4099
Number of Observations Used	2933

Summary of the Number of Event and Censored Values			
Total	Event	Censored	Percent Censored
2933	75	2858	97.44

Convergence Status
Convergence criterion (GCONV=1E-8) satisfied.

Model Fit Statistics		
Criterion	Without Covariates	With Covariates
-2 LOG L	1108.947	1052.293
AIC	1108.947	1082.293
SBC	1108.947	1117.055

Testing Global Null Hypothesis: BETA=0			
Test	Chi-Square	DF	Pr > ChiSq
Likelihood Ratio	56.6544	15	<.0001
Score	64.2225	15	<.0001
Wald	55.9993	15	<.0001

derive.out_lipid_mi_4099

The PHREG Procedure

Imputation Number=4

Analysis of Maximum Likelihood Estimates									
Parameter	DF	Parameter Estimate	Standard Error	Chi-Square	Pr > ChiSq	Hazard Ratio	95% Hazard Ratio Confidence Limits		Label
Age	1	0.01702	0.01182	2.0749	0.1497	1.017	0.994	1.041	
Male (vs. female)	1	-0.37076	0.32522	1.2996	0.2543	0.690	0.365	1.306	Male (vs. female)
History of HTN	1	0.23896	0.28672	0.6946	0.4046	1.270	0.724	2.228	History of HTN
History of DM	1	0.58241	0.24019	5.8798	0.0153	1.790	1.118	2.867	
History of HF	1	0.76836	0.28041	7.5082	0.0061	2.156	1.245	3.736	History of HF
History of MI	1	0.47467	0.47318	1.0063	0.3158	1.607	0.636	4.064	
Previous coronary/LEAD intervention	1	0.13664	0.27103	0.2542	0.6142	1.146	0.674	1.950	
History of ischemic stroke/ TIA	1	-0.11694	0.37300	0.0983	0.7539	0.890	0.428	1.848	
Cigarette smoking history	1	0.64824	0.29597	4.7969	0.0285	1.912	1.071	3.416	Cigarette smoking history
fibrate_use	1	-13.16671	513.66883	0.0007	0.9796	0.000	0.000	.	
BMI_WHOAsia_D1	1	0.23345	0.32532	0.5149	0.4730	1.263	0.668	2.389	
BMI_WHOAsia_D2	1	-0.17077	0.27351	0.3898	0.5324	0.843	0.493	1.441	
CKD_refgt60_D2	1	0.62367	0.26370	5.5937	0.0180	1.866	1.113	3.128	
CKD_refgt60_D3	1	1.20351	0.36800	10.6952	0.0011	3.332	1.620	6.854	
Under statin & LDL ≥ 100 mg/dL	1	0.53883	0.23416	5.2952	0.0214	1.714	1.083	2.712	

derive.out_lipid_mi_4099

The PHREG Procedure

Imputation Number=5

Model Information		
Data Set	WORK.OUT_LIPID_MI_4099	
Dependent Variable	Prim_Outc_Time	
Censoring Variable	Prim_Outc	Prim_Outc
Censoring Value(s)	0	
Ties Handling	BRESLOW	

Number of Observations Read	4099
Number of Observations Used	2933

Summary of the Number of Event and Censored Values			
Total	Event	Censored	Percent Censored
2933	75	2858	97.44

Convergence Status
Convergence criterion (GCONV=1E-8) satisfied.

Model Fit Statistics		
Criterion	Without Covariates	With Covariates
-2 LOG L	1108.947	1054.028
AIC	1108.947	1084.028
SBC	1108.947	1118.790

Testing Global Null Hypothesis: BETA=0			
Test	Chi-Square	DF	Pr > ChiSq
Likelihood Ratio	54.9194	15	<.0001
Score	61.7718	15	<.0001
Wald	54.2086	15	<.0001

derive.out_lipid_mi_4099

The PHREG Procedure

Imputation Number=5

Analysis of Maximum Likelihood Estimates									
Parameter	DF	Parameter Estimate	Standard Error	Chi-Square	Pr > ChiSq	Hazard Ratio	95% Hazard Ratio Confidence Limits		Label
Age	1	0.01672	0.01177	2.0183	0.1554	1.017	0.994	1.041	
Male (vs. female)	1	-0.38350	0.32639	1.3805	0.2400	0.681	0.359	1.292	Male (vs. female)
History of HTN	1	0.24189	0.28640	0.7133	0.3983	1.274	0.727	2.233	History of HTN
History of DM	1	0.57464	0.23982	5.7416	0.0166	1.776	1.110	2.842	
History of HF	1	0.78221	0.28134	7.7300	0.0054	2.186	1.260	3.795	History of HF
History of MI	1	0.47413	0.47692	0.9883	0.3202	1.607	0.631	4.091	
Previous coronary/LEAD intervention	1	0.14508	0.27197	0.2845	0.5937	1.156	0.678	1.970	
History of ischemic stroke/ TIA	1	-0.11872	0.37450	0.1005	0.7512	0.888	0.426	1.850	
Cigarette smoking history	1	0.64726	0.29668	4.7597	0.0291	1.910	1.068	3.417	Cigarette smoking history
fibrate_use	1	-13.11387	493.50243	0.0007	0.9788	0.000	0.000	.	
BMI_WHOAsia_D1	1	0.26736	0.32871	0.6615	0.4160	1.307	0.686	2.488	
BMI_WHOAsia_D2	1	-0.10374	0.27496	0.1423	0.7060	0.901	0.526	1.545	
CKD_refgt60_D2	1	0.62146	0.26299	5.5839	0.0181	1.862	1.112	3.117	
CKD_refgt60_D3	1	1.14227	0.36757	9.6572	0.0019	3.134	1.525	6.441	
Under statin & LDL ≥ 100 mg/dL	1	0.49449	0.23350	4.4846	0.0342	1.640	1.038	2.591	

phregparms_lipid_mi_4099

The MIANALYZE Procedure

Model Information	
PARMS Data Set	WORK.PHREGPARMS_LIPID_MI_4099
Number of Imputations	5

Variance Information							
Parameter	Variance			DF	Relative Increase in Variance	Fraction Missing Information	Relative Efficiency
	Between	Within	Total				
age	0.000000396	0.000139	0.000140	345482	0.003414	0.003408	0.999319
MALE (VS. FEMALE)	0.000191	0.105921	0.106150	857682	0.002164	0.002162	0.999568
History of HTN	0.000028298	0.081840	0.081874	2.33E7	0.000415	0.000415	0.999917
History of DM	0.000070460	0.057974	0.058058	1.89E6	0.001458	0.001457	0.999709
History of HF	0.000109	0.078835	0.078965	1.46E6	0.001656	0.001654	0.999669
History of MI	0.000499	0.226305	0.226904	575240	0.002644	0.002640	0.999472
Previous coronary/LEAD intervention	0.000173	0.073674	0.073881	508471	0.002813	0.002809	0.999439
History of ischemic stroke/ TIA	0.000378	0.140036	0.140490	383307	0.003241	0.003236	0.999353
Cigarette smoking history	0.000053357	0.087643	0.087707	7.51E6	0.000731	0.000730	0.999854
fibrate_use	0.002580	256962	256962	276E14	1.2048065E-8	1.2048065E-8	1.000000
BMI_WHOAsia_D1	0.001121	0.105556	0.106901	25259	0.012744	0.012662	0.997474
BMI_WHOAsia_D2	0.001555	0.075212	0.077078	6828.2	0.024804	0.024489	0.995126
CKD_refgt60_D2	0.000070165	0.069350	0.069435	2.72E6	0.001214	0.001213	0.999757
CKD_refgt60_D3	0.002510	0.135499	0.138511	8461.4	0.022226	0.021974	0.995625
Under statin & LDL ≥ 100 mg/dL	0.000481	0.054925	0.055502	37002	0.010506	0.010451	0.997914

phregparms_lipid_mi_4099

The MIANALYZE Procedure

Parameter Estimates							
Parameter	Estimate	Std Error	95% Confidence Limits		DF	Minimum	Maximum
age	0.017055	0.011817	-0.01	0.0402	345482	0.016484	0.018117
MALE (VS. FEMALE)	-0.363287	0.325807	-1.00	0.2753	857682	-0.383496	-0.349183
History of HTN	0.244161	0.286137	-0.32	0.8050	2.33E7	0.238959	0.250052
History of DM	0.570231	0.240953	0.10	1.0425	1.89E6	0.561483	0.582412
History of HF	0.771198	0.281007	0.22	1.3220	1.46E6	0.756358	0.782214
History of MI	0.478518	0.476344	-0.46	1.4121	575240	0.456124	0.516080
Previous coronary/LEAD intervention	0.128626	0.271811	-0.40	0.6614	508471	0.113172	0.145078
History of ischemic stroke/ TIA	-0.123130	0.374820	-0.86	0.6115	383307	-0.143394	-0.095961
Cigarette smoking history	0.645482	0.296154	0.07	1.2259	7.51E6	0.636181	0.655104
fibrate_use	-13.155244	506.914217	-1006.69	980.3784	276E14	-13.204494	-13.091423
BMI_WHOAsia_D1	0.263208	0.326957	-0.38	0.9041	25259	0.225182	0.302463
BMI_WHOAsia_D2	-0.146664	0.277629	-0.69	0.3976	6828.2	-0.189211	-0.103740
CKD_refgt60_D2	0.630118	0.263505	0.11	1.1466	2.72E6	0.621457	0.642094
CKD_refgt60_D3	1.134444	0.372170	0.40	1.8640	8461.4	1.084978	1.203505
Under statin & LDL ≥ 100 mg/dL	0.505238	0.235589	0.04	0.9670	37002	0.479262	0.538828

phregparms_lipid_mi_4099

The MIANALYZE Procedure

Parameter Estimates			
Parameter	Theta0	t for H0: Parameter=Theta0	Pr > |t|
age	0	1.44	0.1489
MALE (VS. FEMALE)	0	-1.12	0.2648
History of HTN	0	0.85	0.3935
History of DM	0	2.37	0.0180
History of HF	0	2.74	0.0061
History of MI	0	1.00	0.3151
Previous coronary/LEAD intervention	0	0.47	0.6361
History of ischemic stroke/ TIA	0	-0.33	0.7425
Cigarette smoking history	0	2.18	0.0293
fibrate_use	0	-0.03	0.9793
BMI_WHOAsia_D1	0	0.81	0.4208
BMI_WHOAsia_D2	0	-0.53	0.5973
CKD_refgt60_D2	0	2.39	0.0168
CKD_refgt60_D3	0	3.05	0.0023
Under statin & LDL ≥ 100 mg/dL	0	2.14	0.0320

phregparms_mianalyze_mi_4099

Obs	Parm	Estimate	EST_exp	LCLMean_exp	UCLMean_exp	Probt
1	age	0.017055	1.01720	0.99391	1.04104	0.1489
2	MALE (VS. FEMALE)	-0.363287	0.69539	0.36720	1.31690	0.2648
3	History of HTN	0.244161	1.27655	0.72858	2.23665	0.3935
4	History of DM	0.570231	1.76868	1.10293	2.83627	0.0180
5	History of HF	0.771198	2.16236	1.24662	3.75078	0.0061
6	History of MI	0.478518	1.61368	0.63438	4.10472	0.3151
7	Previous coronary/LEAD intervention	0.128626	1.13727	0.66757	1.93744	0.6361
8	History of ischemic stroke/ TIA	-0.123130	0.88415	0.42411	1.84321	0.7425
9	Cigarette smoking history	0.645482	1.90691	1.06719	3.40734	0.0293
10	fibrate_use	-13.155244	0.00000	0.00000	.	0.9793
11	BMI_WHOAsia_D1	0.263208	1.30110	0.68547	2.46962	0.4208
12	BMI_WHOAsia_D2	-0.146664	0.86358	0.50112	1.48821	0.5973
13	CKD_refgt60_D2	0.630118	1.87783	1.12037	3.14740	0.0168
14	CKD_refgt60_D3	1.134444	3.10944	1.49915	6.44941	0.0023
15	Under statin & LDL ≥ 100 mg/dL	0.505238	1.65738	1.04443	2.63004	0.0320

derive.out_lipid_mi_4099

The PHREG Procedure

Imputation Number=1

Model Information		
Data Set	WORK.OUT_LIPID_MI_4099	
Dependent Variable	Prim_Outc_Time	
Censoring Variable	Prim_Outc	Prim_Outc
Censoring Value(s)	0	
Ties Handling	BRESLOW	

Number of Observations Read	4099
Number of Observations Used	1510

Summary of the Number of Event and Censored Values			
Total	Event	Censored	Percent Censored
1510	36	1474	97.62

Convergence Status
Convergence criterion (GCONV=1E-8) satisfied.

Model Fit Statistics		
Criterion	Without Covariates	With Covariates
-2 LOG L	480.561	450.428
AIC	480.561	480.428
SBC	480.561	504.180

Testing Global Null Hypothesis: BETA=0			
Test	Chi-Square	DF	Pr > ChiSq
Likelihood Ratio	30.1337	15	0.0114
Score	32.2233	15	0.0060
Wald	28.3280	15	0.0196

derive.out_lipid_mi_4099

The PHREG Procedure

Imputation Number=1

Analysis of Maximum Likelihood Estimates									
Parameter	DF	Parameter Estimate	Standard Error	Chi-Square	Pr > ChiSq	Hazard Ratio	95% Hazard Ratio Confidence Limits		Label
Age	1	0.01565	0.01718	0.8289	0.3626	1.016	0.982	1.051	
Male (vs. female)	1	-0.46146	0.45928	1.0095	0.3150	0.630	0.256	1.551	Male (vs. female)
History of HTN	1	0.40507	0.43410	0.8707	0.3508	1.499	0.640	3.511	History of HTN
History of DM	1	0.50394	0.35034	2.0691	0.1503	1.655	0.833	3.289	
History of HF	1	0.93356	0.38681	5.8250	0.0158	2.544	1.192	5.429	History of HF
History of MI	1	0.77766	0.77380	1.0100	0.3149	2.176	0.478	9.917	
Previous coronary/LEAD intervention	1	0.41885	0.42363	0.9775	0.3228	1.520	0.663	3.487	
History of ischemic stroke/ TIA	1	-0.10138	0.56638	0.0320	0.8579	0.904	0.298	2.742	
Cigarette smoking history	1	0.48940	0.42592	1.3203	0.2505	1.631	0.708	3.759	Cigarette smoking history
fibrate_use	1	-13.09846	677.21097	0.0004	0.9846	0.000	0.000	.	
BMI_WHOAsia_D1	1	0.26965	0.45624	0.3493	0.5545	1.310	0.535	3.202	
BMI_WHOAsia_D2	1	-0.36285	0.40415	0.8061	0.3693	0.696	0.315	1.536	
CKD_refgt60_D2	1	0.66639	0.37466	3.1636	0.0753	1.947	0.934	4.058	
CKD_refgt60_D3	1	0.89640	0.57865	2.3998	0.1214	2.451	0.788	7.618	
high-intensity statin (vs. moderate-intensity)	1	-0.22781	0.73960	0.0949	0.7581	0.796	0.187	3.393	

derive.out_lipid_mi_4099

The PHREG Procedure

Imputation Number=2

Model Information		
Data Set	WORK.OUT_LIPID_MI_4099	
Dependent Variable	Prim_Outc_Time	
Censoring Variable	Prim_Outc	Prim_Outc
Censoring Value(s)	0	
Ties Handling	BRESLOW	

Number of Observations Read	4099
Number of Observations Used	1517

Summary of the Number of Event and Censored Values			
Total	Event	Censored	Percent Censored
1517	36	1481	97.63

Convergence Status
Convergence criterion (GCONV=1E-8) satisfied.

Model Fit Statistics		
Criterion	Without Covariates	With Covariates
-2 LOG L	480.763	450.765
AIC	480.763	480.765
SBC	480.763	504.518

Testing Global Null Hypothesis: BETA=0			
Test	Chi-Square	DF	Pr > ChiSq
Likelihood Ratio	29.9973	15	0.0119
Score	32.3832	15	0.0057
Wald	28.1350	15	0.0207

derive.out_lipid_mi_4099

The PHREG Procedure

Imputation Number=2

Analysis of Maximum Likelihood Estimates									
Parameter	DF	Parameter Estimate	Standard Error	Chi-Square	Pr > ChiSq	Hazard Ratio	95% Hazard Ratio Confidence Limits		Label
Age	1	0.01381	0.01716	0.6475	0.4210	1.014	0.980	1.049	
Male (vs. female)	1	-0.46750	0.46135	1.0269	0.3109	0.627	0.254	1.548	Male (vs. female)
History of HTN	1	0.42511	0.43327	0.9627	0.3265	1.530	0.654	3.576	History of HTN
History of DM	1	0.48355	0.35255	1.8813	0.1702	1.622	0.813	3.237	
History of HF	1	0.95298	0.38765	6.0433	0.0140	2.593	1.213	5.544	History of HF
History of MI	1	0.73914	0.77782	0.9030	0.3420	2.094	0.456	9.618	
Previous coronary/LEAD intervention	1	0.43391	0.42490	1.0429	0.3072	1.543	0.671	3.549	
History of ischemic stroke/ TIA	1	-0.14246	0.56863	0.0628	0.8022	0.867	0.285	2.643	
Cigarette smoking history	1	0.46953	0.42840	1.2012	0.2731	1.599	0.691	3.703	Cigarette smoking history
fibrate_use	1	-13.17496	684.08853	0.0004	0.9846	0.000	0.000	.	
BMI_WHOAsia_D1	1	0.30198	0.45643	0.4377	0.5082	1.353	0.553	3.309	
BMI_WHOAsia_D2	1	-0.29152	0.40266	0.5242	0.4691	0.747	0.339	1.645	
CKD_refgt60_D2	1	0.69411	0.37404	3.4437	0.0635	2.002	0.962	4.167	
CKD_refgt60_D3	1	0.84549	0.58179	2.1120	0.1462	2.329	0.745	7.285	
high-intensity statin (vs. moderate-intensity)	1	-0.20921	0.74600	0.0786	0.7791	0.811	0.188	3.501	

derive.out_lipid_mi_4099

The PHREG Procedure

Imputation Number=3

Model Information		
Data Set	WORK.OUT_LIPID_MI_4099	
Dependent Variable	Prim_Outc_Time	
Censoring Variable	Prim_Outc	Prim_Outc
Censoring Value(s)	0	
Ties Handling	BRESLOW	

Number of Observations Read	4099
Number of Observations Used	1519

Summary of the Number of Event and Censored Values			
Total	Event	Censored	Percent Censored
1519	36	1483	97.63

Convergence Status
Convergence criterion (GCONV=1E-8) satisfied.

Model Fit Statistics		
Criterion	Without Covariates	With Covariates
-2 LOG L	480.637	450.288
AIC	480.637	480.288
SBC	480.637	504.040

Testing Global Null Hypothesis: BETA=0			
Test	Chi-Square	DF	Pr > ChiSq
Likelihood Ratio	30.3493	15	0.0107
Score	32.7513	15	0.0051
Wald	28.5186	15	0.0185

derive.out_lipid_mi_4099

The PHREG Procedure

Imputation Number=3

Analysis of Maximum Likelihood Estimates									
Parameter	DF	Parameter Estimate	Standard Error	Chi-Square	Pr > ChiSq	Hazard Ratio	95% Hazard Ratio Confidence Limits		Label
Age	1	0.01493	0.01734	0.7406	0.3895	1.015	0.981	1.050	
Male (vs. female)	1	-0.46848	0.45709	1.0505	0.3054	0.626	0.256	1.533	Male (vs. female)
History of HTN	1	0.39011	0.43412	0.8075	0.3688	1.477	0.631	3.459	History of HTN
History of DM	1	0.47839	0.35215	1.8455	0.1743	1.613	0.809	3.217	
History of HF	1	0.90845	0.39052	5.4114	0.0200	2.480	1.154	5.333	History of HF
History of MI	1	0.75511	0.77476	0.9499	0.3297	2.128	0.466	9.714	
Previous coronary/LEAD intervention	1	0.46720	0.42309	1.2194	0.2695	1.596	0.696	3.656	
History of ischemic stroke/ TIA	1	-0.09916	0.56879	0.0304	0.8616	0.906	0.297	2.761	
Cigarette smoking history	1	0.50367	0.42369	1.4132	0.2345	1.655	0.721	3.797	Cigarette smoking history
fibrate_use	1	-13.07464	697.69698	0.0004	0.9850	0.000	0.000	.	
BMI_WHOAsia_D1	1	0.26818	0.45701	0.3444	0.5573	1.308	0.534	3.202	
BMI_WHOAsia_D2	1	-0.40920	0.40214	1.0354	0.3089	0.664	0.302	1.461	
CKD_refgt60_D2	1	0.66172	0.37709	3.0794	0.0793	1.938	0.926	4.058	
CKD_refgt60_D3	1	0.93734	0.58859	2.5361	0.1113	2.553	0.806	8.093	
high-intensity statin (vs. moderate-intensity)	1	-0.31061	0.74211	0.1752	0.6755	0.733	0.171	3.139	

derive.out_lipid_mi_4099

The PHREG Procedure

Imputation Number=4

Model Information		
Data Set	WORK.OUT_LIPID_MI_4099	
Dependent Variable	Prim_Outc_Time	
Censoring Variable	Prim_Outc	Prim_Outc
Censoring Value(s)	0	
Ties Handling	BRESLOW	

Number of Observations Read	4099
Number of Observations Used	1526

Summary of the Number of Event and Censored Values			
Total	Event	Censored	Percent Censored
1526	36	1490	97.64

Convergence Status
Convergence criterion (GCONV=1E-8) satisfied.

Model Fit Statistics		
Criterion	Without Covariates	With Covariates
-2 LOG L	481.134	451.335
AIC	481.134	481.335
SBC	481.134	505.087

Testing Global Null Hypothesis: BETA=0			
Test	Chi-Square	DF	Pr > ChiSq
Likelihood Ratio	29.7994	15	0.0127
Score	32.0035	15	0.0064
Wald	27.9487	15	0.0219

derive.out_lipid_mi_4099

The PHREG Procedure

Imputation Number=4

Analysis of Maximum Likelihood Estimates									
Parameter	DF	Parameter Estimate	Standard Error	Chi-Square	Pr > ChiSq	Hazard Ratio	95% Hazard Ratio Confidence Limits		Label
Age	1	0.01554	0.01737	0.8004	0.3710	1.016	0.982	1.051	
Male (vs. female)	1	-0.43403	0.45956	0.8920	0.3449	0.648	0.263	1.595	Male (vs. female)
History of HTN	1	0.43678	0.43331	1.0161	0.3134	1.548	0.662	3.618	History of HTN
History of DM	1	0.46146	0.35343	1.7047	0.1917	1.586	0.794	3.171	
History of HF	1	0.93863	0.38710	5.8796	0.0153	2.556	1.197	5.459	History of HF
History of MI	1	0.71479	0.77597	0.8485	0.3570	2.044	0.447	9.353	
Previous coronary/LEAD intervention	1	0.41455	0.42302	0.9604	0.3271	1.514	0.661	3.468	
History of ischemic stroke/ TIA	1	-0.20408	0.56656	0.1297	0.7187	0.815	0.269	2.475	
Cigarette smoking history	1	0.48080	0.42403	1.2857	0.2568	1.617	0.704	3.713	Cigarette smoking history
fibrate_use	1	-13.07161	688.56154	0.0004	0.9849	0.000	0.000	.	
BMI_WHOAsia_D1	1	0.26255	0.45703	0.3300	0.5656	1.300	0.531	3.185	
BMI_WHOAsia_D2	1	-0.36760	0.40289	0.8325	0.3616	0.692	0.314	1.525	
CKD_refgt60_D2	1	0.67360	0.37609	3.2080	0.0733	1.961	0.938	4.099	
CKD_refgt60_D3	1	0.85083	0.58456	2.1185	0.1455	2.342	0.745	7.364	
high-intensity statin (vs. moderate-intensity)	1	-0.25499	0.74162	0.1182	0.7310	0.775	0.181	3.315	

derive.out_lipid_mi_4099

The PHREG Procedure

Imputation Number=5

Model Information		
Data Set	WORK.OUT_LIPID_MI_4099	
Dependent Variable	Prim_Outc_Time	
Censoring Variable	Prim_Outc	Prim_Outc
Censoring Value(s)	0	
Ties Handling	BRESLOW	

Number of Observations Read	4099
Number of Observations Used	1513

Summary of the Number of Event and Censored Values			
Total	Event	Censored	Percent Censored
1513	36	1477	97.62

Convergence Status
Convergence criterion (GCONV=1E-8) satisfied.

Model Fit Statistics		
Criterion	Without Covariates	With Covariates
-2 LOG L	480.504	450.150
AIC	480.504	480.150
SBC	480.504	503.903

Testing Global Null Hypothesis: BETA=0			
Test	Chi-Square	DF	Pr > ChiSq
Likelihood Ratio	30.3540	15	0.0107
Score	32.3161	15	0.0058
Wald	28.3196	15	0.0197

derive.out_lipid_mi_4099

The PHREG Procedure

Imputation Number=5

Analysis of Maximum Likelihood Estimates									
Parameter	DF	Parameter Estimate	Standard Error	Chi-Square	Pr > ChiSq	Hazard Ratio	95% Hazard Ratio Confidence Limits		Label
Age	1	0.01715	0.01730	0.9828	0.3215	1.017	0.983	1.052	
Male (vs. female)	1	-0.42213	0.45795	0.8497	0.3566	0.656	0.267	1.609	Male (vs. female)
History of HTN	1	0.41121	0.43289	0.9023	0.3422	1.509	0.646	3.524	History of HTN
History of DM	1	0.51984	0.35059	2.1986	0.1381	1.682	0.846	3.343	
History of HF	1	0.92378	0.38927	5.6317	0.0176	2.519	1.174	5.402	History of HF
History of MI	1	0.76690	0.77571	0.9774	0.3228	2.153	0.471	9.848	
Previous coronary/LEAD intervention	1	0.40866	0.42214	0.9371	0.3330	1.505	0.658	3.442	
History of ischemic stroke/ TIA	1	-0.18717	0.56822	0.1085	0.7419	0.829	0.272	2.526	
Cigarette smoking history	1	0.49779	0.42423	1.3769	0.2406	1.645	0.716	3.778	Cigarette smoking history
fibrate_use	1	-13.04952	671.21808	0.0004	0.9845	0.000	0.000	.	
BMI_WHOAsia_D1	1	0.31206	0.45843	0.4634	0.4961	1.366	0.556	3.355	
BMI_WHOAsia_D2	1	-0.34852	0.40326	0.7469	0.3875	0.706	0.320	1.556	
CKD_refgt60_D2	1	0.69235	0.37426	3.4221	0.0643	1.998	0.960	4.162	
CKD_refgt60_D3	1	0.82936	0.58194	2.0311	0.1541	2.292	0.733	7.170	
high-intensity statin (vs. moderate-intensity)	1	-0.23944	0.74500	0.1033	0.7479	0.787	0.183	3.390	

phregparms_lipid_mi_4099

The MIANALYZE Procedure

Model Information	
PARMS Data Set	WORK.PHREGPARMS_LIPID_MI_4099
Number of Imputations	5

Variance Information							
Parameter	Variance			DF	Relative Increase in Variance	Fraction Missing Information	Relative Efficiency
	Between	Within	Total				
age	0.000001472	0.000298	0.000300	115426	0.005922	0.005904	0.998821
MALE (VS. FEMALE)	0.000452	0.210725	0.211267	606843	0.002574	0.002571	0.999486
History of HTN	0.000325	0.187953	0.188343	933129	0.002075	0.002073	0.999586
History of DM	0.000519	0.123772	0.124395	159808	0.005028	0.005015	0.998998
History of HF	0.000277	0.150756	0.151088	827135	0.002204	0.002201	0.999560
History of MI	0.000608	0.601574	0.602304	2.73E6	0.001213	0.001212	0.999758
Previous coronary/LEAD intervention	0.000552	0.179231	0.179894	295000	0.003696	0.003689	0.999263
History of ischemic stroke/ TIA	0.002316	0.322305	0.325084	54746	0.008621	0.008584	0.998286
Cigarette smoking history	0.000184	0.180843	0.181063	2.69E6	0.001221	0.001221	0.999756
fibrate_use	0.002357	467605	467605	109E15	6.0493676E-9	6.0493675E-9	1.000000
BMI_WHOAsia_D1	0.000505	0.208874	0.209480	477715	0.002902	0.002898	0.999421
BMI_WHOAsia_D2	0.001806	0.162426	0.164594	23060	0.013346	0.013256	0.997356
CKD_refgt60_D2	0.000221	0.140797	0.141062	1.13E6	0.001883	0.001881	0.999624
CKD_refgt60_D3	0.001958	0.340023	0.342373	84889	0.006912	0.006888	0.998624
high-intensity statin (vs. moderate-intensity)	0.001488	0.551856	0.553642	384374	0.003236	0.003231	0.999354

phregparms_lipid_mi_4099

The MIANALYZE Procedure

Parameter Estimates							
Parameter	Estimate	Std Error	95% Confidence Limits		DF	Minimum	Maximum
age	0.015414	0.017323	-0.02	0.049	115426	0.013810	0.017148
MALE (VS. FEMALE)	-0.450721	0.459638	-1.35	0.450	606843	-0.468484	-0.422132
History of HTN	0.413656	0.433985	-0.44	1.264	933129	0.390113	0.436779
History of DM	0.489437	0.352696	-0.20	1.181	159808	0.461456	0.519842
History of HF	0.931480	0.388701	0.17	1.693	827135	0.908447	0.952979
History of MI	0.750722	0.776082	-0.77	2.272	2.73E6	0.714789	0.777664
Previous coronary/LEAD intervention	0.428633	0.424139	-0.40	1.260	295000	0.408657	0.467196
History of ischemic stroke/ TIA	-0.146850	0.570162	-1.26	0.971	54746	-0.204078	-0.099159
Cigarette smoking history	0.488239	0.425515	-0.35	1.322	2.69E6	0.469528	0.503670
fibrate_use	-13.093839	683.816293	-1353.35	1327.161	109E15	-13.174961	-13.049521
BMI_WHOAsia_D1	0.282883	0.457690	-0.61	1.180	477715	0.262550	0.312059
BMI_WHOAsia_D2	-0.355937	0.405702	-1.15	0.439	23060	-0.409202	-0.291517
CKD_refgt60_D2	0.677636	0.375582	-0.06	1.414	1.13E6	0.661724	0.694112
CKD_refgt60_D3	0.871883	0.585127	-0.27	2.019	84889	0.829364	0.937344
high-intensity statin (vs. moderate-intensity)	-0.248412	0.744072	-1.71	1.210	384374	-0.310609	-0.209208

phregparms_lipid_mi_4099

The MIANALYZE Procedure

Parameter Estimates			
Parameter	Theta0	t for H0: Parameter=Theta0	Pr > |t|
age	0	0.89	0.3736
MALE (VS. FEMALE)	0	-0.98	0.3268
History of HTN	0	0.95	0.3405
History of DM	0	1.39	0.1652
History of HF	0	2.40	0.0166
History of MI	0	0.97	0.3334
Previous coronary/LEAD intervention	0	1.01	0.3122
History of ischemic stroke/ TIA	0	-0.26	0.7967
Cigarette smoking history	0	1.15	0.2512
fibrate_use	0	-0.02	0.9847
BMI_WHOAsia_D1	0	0.62	0.5365
BMI_WHOAsia_D2	0	-0.88	0.3803
CKD_refgt60_D2	0	1.80	0.0712
CKD_refgt60_D3	0	1.49	0.1362
high-intensity statin (vs. moderate-intensity)	0	-0.33	0.7385

phregparms_mianalyze_mi_4099

Obs	Parm	Estimate	EST_exp	LCLMean_exp	UCLMean_exp	Probt
1	age	0.015414	1.01553	0.98163	1.05061	0.3736
2	MALE (VS. FEMALE)	-0.450721	0.63717	0.25883	1.56856	0.3268
3	History of HTN	0.413656	1.51234	0.64601	3.54044	0.3405
4	History of DM	0.489437	1.63140	0.81723	3.25670	0.1652
5	History of HF	0.931480	2.53826	1.18488	5.43750	0.0166
6	History of MI	0.750722	2.11853	0.46284	9.69700	0.3334
7	Previous coronary/LEAD intervention	0.428633	1.53516	0.66853	3.52519	0.3122
8	History of ischemic stroke/ TIA	-0.146850	0.86342	0.28242	2.63971	0.7967
9	Cigarette smoking history	0.488239	1.62944	0.70768	3.75179	0.2512
10	fibrate_use	-13.093839	0.00000	0.00000	.	0.9847
11	BMI_WHOAsia_D1	0.282883	1.32695	0.54109	3.25418	0.5365
12	BMI_WHOAsia_D2	-0.355937	0.70052	0.31628	1.55157	0.3803
13	CKD_refgt60_D2	0.677636	1.96922	0.94319	4.11140	0.0712
14	CKD_refgt60_D3	0.871883	2.39141	0.75960	7.52874	0.1362
15	high-intensity statin (vs. moderate-intensity)	-0.248412	0.78004	0.18145	3.35330	0.7385

derive.out_lipid_mi_4099

The PHREG Procedure

Imputation Number=1

Model Information		
Data Set	WORK.OUT_LIPID_MI_4099	
Dependent Variable	Prim_Outc_Time	
Censoring Variable	Prim_Outc	Prim_Outc
Censoring Value(s)	0	
Ties Handling	BRESLOW	

Number of Observations Read	4099
Number of Observations Used	1636

Summary of the Number of Event and Censored Values			
Total	Event	Censored	Percent Censored
1636	36	1600	97.80

Convergence Status
Convergence criterion (GCONV=1E-8) satisfied.

Model Fit Statistics		
Criterion	Without Covariates	With Covariates
-2 LOG L	484.959	451.748
AIC	484.959	481.748
SBC	484.959	505.501

Testing Global Null Hypothesis: BETA=0			
Test	Chi-Square	DF	Pr > ChiSq
Likelihood Ratio	33.2106	15	0.0044
Score	33.5822	15	0.0039
Wald	29.6507	15	0.0132

derive.out_lipid_mi_4099

The PHREG Procedure

Imputation Number=1

Analysis of Maximum Likelihood Estimates									
Parameter	DF	Parameter Estimate	Standard Error	Chi-Square	Pr > ChiSq	Hazard Ratio	95% Hazard Ratio Confidence Limits		Label
Age	1	0.01534	0.01766	0.7550	0.3849	1.015	0.981	1.051	
Male (vs. female)	1	-0.40352	0.46183	0.7634	0.3823	0.668	0.270	1.651	Male (vs. female)
History of HTN	1	0.42483	0.43353	0.9603	0.3271	1.529	0.654	3.577	History of HTN
History of DM	1	0.50359	0.35208	2.0458	0.1526	1.655	0.830	3.299	
History of HF	1	0.78791	0.39717	3.9355	0.0473	2.199	1.010	4.789	History of HF
History of MI	1	1.58494	1.06395	2.2191	0.1363	4.879	0.606	39.261	
Previous coronary/LEAD intervention	1	0.48595	0.44307	1.2029	0.2727	1.626	0.682	3.874	
History of ischemic stroke/ TIA	1	0.34264	0.50878	0.4535	0.5007	1.409	0.520	3.818	
Cigarette smoking history	1	0.53239	0.42058	1.6023	0.2056	1.703	0.747	3.883	Cigarette smoking history
fibrate_use	1	-14.03788	1094	0.0002	0.9898	0.000	0.000	.	
BMI_WHOAsia_D1	1	0.30758	0.46287	0.4416	0.5064	1.360	0.549	3.370	
BMI_WHOAsia_D2	1	-0.27662	0.40637	0.4634	0.4961	0.758	0.342	1.682	
CKD_refgt60_D2	1	0.65844	0.37670	3.0552	0.0805	1.932	0.923	4.042	
CKD_refgt60_D3	1	0.93385	0.57888	2.6024	0.1067	2.544	0.818	7.912	
low-intensity statin (vs. moderate-intensity)	1	-0.82390	0.73333	1.2623	0.2612	0.439	0.104	1.847	

derive.out_lipid_mi_4099

The PHREG Procedure

Imputation Number=2

Model Information		
Data Set	WORK.OUT_LIPID_MI_4099	
Dependent Variable	Prim_Outc_Time	
Censoring Variable	Prim_Outc	Prim_Outc
Censoring Value(s)	0	
Ties Handling	BRESLOW	

Number of Observations Read	4099
Number of Observations Used	1650

Summary of the Number of Event and Censored Values			
Total	Event	Censored	Percent Censored
1650	36	1614	97.82

Convergence Status
Convergence criterion (GCONV=1E-8) satisfied.

Model Fit Statistics		
Criterion	Without Covariates	With Covariates
-2 LOG L	485.475	452.256
AIC	485.475	482.256
SBC	485.475	506.009

Testing Global Null Hypothesis: BETA=0			
Test	Chi-Square	DF	Pr > ChiSq
Likelihood Ratio	33.2183	15	0.0044
Score	33.7932	15	0.0036
Wald	29.5990	15	0.0135

derive.out_lipid_mi_4099

The PHREG Procedure

Imputation Number=2

Analysis of Maximum Likelihood Estimates									
Parameter	DF	Parameter Estimate	Standard Error	Chi-Square	Pr > ChiSq	Hazard Ratio	95% Hazard Ratio Confidence Limits		Label
Age	1	0.01360	0.01749	0.6048	0.4367	1.014	0.980	1.049	
Male (vs. female)	1	-0.40915	0.46281	0.7816	0.3767	0.664	0.268	1.645	Male (vs. female)
History of HTN	1	0.44197	0.43320	1.0409	0.3076	1.556	0.666	3.637	History of HTN
History of DM	1	0.51019	0.35242	2.0957	0.1477	1.666	0.835	3.323	
History of HF	1	0.81985	0.39702	4.2643	0.0389	2.270	1.043	4.943	History of HF
History of MI	1	1.59737	1.06534	2.2482	0.1338	4.940	0.612	39.861	
Previous coronary/LEAD intervention	1	0.49865	0.44344	1.2645	0.2608	1.646	0.690	3.927	
History of ischemic stroke/ TIA	1	0.34849	0.50841	0.4698	0.4931	1.417	0.523	3.838	
Cigarette smoking history	1	0.49747	0.42275	1.3847	0.2393	1.645	0.718	3.766	Cigarette smoking history
fibrate_use	1	-14.07798	1085	0.0002	0.9896	0.000	0.000	.	
BMI_WHOAsia_D1	1	0.30611	0.46268	0.4377	0.5082	1.358	0.548	3.363	
BMI_WHOAsia_D2	1	-0.20802	0.40447	0.2645	0.6070	0.812	0.368	1.794	
CKD_refgt60_D2	1	0.67855	0.37509	3.2726	0.0704	1.971	0.945	4.111	
CKD_refgt60_D3	1	0.85901	0.58080	2.1874	0.1391	2.361	0.756	7.370	
low-intensity statin (vs. moderate-intensity)	1	-0.86385	0.73205	1.3925	0.2380	0.422	0.100	1.770	

derive.out_lipid_mi_4099

The PHREG Procedure

Imputation Number=3

Model Information		
Data Set	WORK.OUT_LIPID_MI_4099	
Dependent Variable	Prim_Outc_Time	
Censoring Variable	Prim_Outc	Prim_Outc
Censoring Value(s)	0	
Ties Handling	BRESLOW	

Number of Observations Read	4099
Number of Observations Used	1648

Summary of the Number of Event and Censored Values			
Total	Event	Censored	Percent Censored
1648	36	1612	97.82

Convergence Status
Convergence criterion (GCONV=1E-8) satisfied.

Model Fit Statistics		
Criterion	Without Covariates	With Covariates
-2 LOG L	485.101	451.392
AIC	485.101	481.392
SBC	485.101	505.145

Testing Global Null Hypothesis: BETA=0			
Test	Chi-Square	DF	Pr > ChiSq
Likelihood Ratio	33.7087	15	0.0037
Score	34.0460	15	0.0034
Wald	30.0793	15	0.0116

derive.out_lipid_mi_4099

The PHREG Procedure

Imputation Number=3

Analysis of Maximum Likelihood Estimates									
Parameter	DF	Parameter Estimate	Standard Error	Chi-Square	Pr > ChiSq	Hazard Ratio	95% Hazard Ratio Confidence Limits		Label
Age	1	0.01499	0.01760	0.7259	0.3942	1.015	0.981	1.051	
Male (vs. female)	1	-0.40727	0.45787	0.7912	0.3737	0.665	0.271	1.633	Male (vs. female)
History of HTN	1	0.41419	0.43384	0.9114	0.3397	1.513	0.647	3.541	History of HTN
History of DM	1	0.50731	0.35239	2.0725	0.1500	1.661	0.832	3.313	
History of HF	1	0.77046	0.39861	3.7360	0.0533	2.161	0.989	4.720	History of HF
History of MI	1	1.60480	1.06346	2.2772	0.1313	4.977	0.619	40.010	
Previous coronary/LEAD intervention	1	0.54535	0.44244	1.5193	0.2177	1.725	0.725	4.106	
History of ischemic stroke/ TIA	1	0.39118	0.50857	0.5916	0.4418	1.479	0.546	4.007	
Cigarette smoking history	1	0.55147	0.41852	1.7362	0.1876	1.736	0.764	3.942	Cigarette smoking history
fibrate_use	1	-12.97148	668.27988	0.0004	0.9845	0.000	0.000	.	
BMI_WHOAsia_D1	1	0.31770	0.46320	0.4704	0.4928	1.374	0.554	3.406	
BMI_WHOAsia_D2	1	-0.32575	0.40404	0.6500	0.4201	0.722	0.327	1.594	
CKD_refgt60_D2	1	0.64355	0.37744	2.9071	0.0882	1.903	0.908	3.988	
CKD_refgt60_D3	1	0.95300	0.58608	2.6440	0.1039	2.593	0.822	8.180	
low-intensity statin (vs. moderate-intensity)	1	-0.88826	0.73169	1.4737	0.2248	0.411	0.098	1.726	

derive.out_lipid_mi_4099

The PHREG Procedure

Imputation Number=4

Model Information		
Data Set	WORK.OUT_LIPID_MI_4099	
Dependent Variable	Prim_Outc_Time	
Censoring Variable	Prim_Outc	Prim_Outc
Censoring Value(s)	0	
Ties Handling	BRESLOW	

Number of Observations Read	4099
Number of Observations Used	1660

Summary of the Number of Event and Censored Values			
Total	Event	Censored	Percent Censored
1660	36	1624	97.83

Convergence Status
Convergence criterion (GCONV=1E-8) satisfied.

Model Fit Statistics		
Criterion	Without Covariates	With Covariates
-2 LOG L	485.821	452.811
AIC	485.821	482.811
SBC	485.821	506.564

Testing Global Null Hypothesis: BETA=0			
Test	Chi-Square	DF	Pr > ChiSq
Likelihood Ratio	33.0099	15	0.0047
Score	33.8098	15	0.0036
Wald	29.5873	15	0.0135

derive.out_lipid_mi_4099

The PHREG Procedure

Imputation Number=4

Analysis of Maximum Likelihood Estimates									
Parameter	DF	Parameter Estimate	Standard Error	Chi-Square	Pr > ChiSq	Hazard Ratio	95% Hazard Ratio Confidence Limits		Label
Age	1	0.01537	0.01762	0.7609	0.3830	1.015	0.981	1.051	
Male (vs. female)	1	-0.38131	0.46051	0.6856	0.4077	0.683	0.277	1.684	Male (vs. female)
History of HTN	1	0.46533	0.43272	1.1564	0.2822	1.593	0.682	3.719	History of HTN
History of DM	1	0.48866	0.35397	1.9058	0.1674	1.630	0.815	3.262	
History of HF	1	0.78502	0.39745	3.9012	0.0483	2.192	1.006	4.778	History of HF
History of MI	1	1.55226	1.06642	2.1187	0.1455	4.722	0.584	38.183	
Previous coronary/LEAD intervention	1	0.48482	0.44243	1.2008	0.2732	1.624	0.682	3.865	
History of ischemic stroke/ TIA	1	0.28414	0.51015	0.3102	0.5775	1.329	0.489	3.611	
Cigarette smoking history	1	0.52407	0.41800	1.5719	0.2099	1.689	0.744	3.832	Cigarette smoking history
fibrate_use	1	-13.00958	669.71398	0.0004	0.9845	0.000	0.000	.	
BMI_WHOAsia_D1	1	0.29579	0.46293	0.4083	0.5229	1.344	0.543	3.330	
BMI_WHOAsia_D2	1	-0.26698	0.40456	0.4355	0.5093	0.766	0.346	1.692	
CKD_refgt60_D2	1	0.65220	0.37612	3.0069	0.0829	1.920	0.919	4.012	
CKD_refgt60_D3	1	0.89076	0.58511	2.3176	0.1279	2.437	0.774	7.672	
low-intensity statin (vs. moderate-intensity)	1	-0.83198	0.73250	1.2901	0.2560	0.435	0.104	1.829	

derive.out_lipid_mi_4099

The PHREG Procedure

Imputation Number=5

Model Information		
Data Set	WORK.OUT_LIPID_MI_4099	
Dependent Variable	Prim_Outc_Time	
Censoring Variable	Prim_Outc	Prim_Outc
Censoring Value(s)	0	
Ties Handling	BRESLOW	

Number of Observations Read	4099
Number of Observations Used	1643

Summary of the Number of Event and Censored Values			
Total	Event	Censored	Percent Censored
1643	36	1607	97.81

Convergence Status
Convergence criterion (GCONV=1E-8) satisfied.

Model Fit Statistics		
Criterion	Without Covariates	With Covariates
-2 LOG L	485.068	451.580
AIC	485.068	481.580
SBC	485.068	505.332

Testing Global Null Hypothesis: BETA=0			
Test	Chi-Square	DF	Pr > ChiSq
Likelihood Ratio	33.4888	15	0.0040
Score	33.7719	15	0.0037
Wald	29.6140	15	0.0134

derive.out_lipid_mi_4099

The PHREG Procedure

Imputation Number=5

Analysis of Maximum Likelihood Estimates									
Parameter	DF	Parameter Estimate	Standard Error	Chi-Square	Pr > ChiSq	Hazard Ratio	95% Hazard Ratio Confidence Limits		Label
Age	1	0.01648	0.01749	0.8871	0.3463	1.017	0.982	1.052	
Male (vs. female)	1	-0.36504	0.45940	0.6314	0.4268	0.694	0.282	1.708	Male (vs. female)
History of HTN	1	0.42318	0.43286	0.9558	0.3283	1.527	0.654	3.566	History of HTN
History of DM	1	0.55488	0.35031	2.5090	0.1132	1.742	0.877	3.461	
History of HF	1	0.77767	0.39924	3.7941	0.0514	2.176	0.995	4.760	History of HF
History of MI	1	1.61037	1.06600	2.2821	0.1309	5.005	0.619	40.435	
Previous coronary/LEAD intervention	1	0.48329	0.44108	1.2005	0.2732	1.621	0.683	3.849	
History of ischemic stroke/ TIA	1	0.30307	0.51054	0.3524	0.5528	1.354	0.498	3.683	
Cigarette smoking history	1	0.52819	0.41857	1.5924	0.2070	1.696	0.747	3.852	Cigarette smoking history
fibrate_use	1	-13.97493	1069	0.0002	0.9896	0.000	0.000	.	
BMI_WHOAsia_D1	1	0.34588	0.46459	0.5543	0.4566	1.413	0.569	3.513	
BMI_WHOAsia_D2	1	-0.26053	0.40577	0.4123	0.5208	0.771	0.348	1.707	
CKD_refgt60_D2	1	0.66836	0.37480	3.1800	0.0745	1.951	0.936	4.067	
CKD_refgt60_D3	1	0.84243	0.58164	2.0978	0.1475	2.322	0.743	7.260	
low-intensity statin (vs. moderate-intensity)	1	-0.86556	0.73274	1.3954	0.2375	0.421	0.100	1.769	

phregparms_lipid_mi_4099

The MIANALYZE Procedure

Model Information	
PARMS Data Set	WORK.PHREGPARMS_LIPID_MI_4099
Number of Imputations	5

Variance Information							
Parameter	Variance			DF	Relative Increase in Variance	Fraction Missing Information	Relative Efficiency
	Between	Within	Total				
age	0.000001066	0.000309	0.000310	235064	0.004142	0.004134	0.999174
MALE (VS. FEMALE)	0.000373	0.212046	0.212494	899567	0.002113	0.002111	0.999578
History of HTN	0.000410	0.187687	0.188179	585930	0.002620	0.002616	0.999477
History of DM	0.000619	0.124072	0.124814	113000	0.005985	0.005967	0.998808
History of HF	0.000359	0.158325	0.158756	541931	0.002724	0.002720	0.999456
History of MI	0.000535	1.134295	1.134937	1.25E7	0.000566	0.000565	0.999887
Previous coronary/LEAD intervention	0.000691	0.195800	0.196629	224798	0.004236	0.004227	0.999155
History of ischemic stroke/ TIA	0.001749	0.259377	0.261476	62084	0.008092	0.008059	0.998391
Cigarette smoking history	0.000377	0.176137	0.176590	608293	0.002571	0.002568	0.999487
fibrate_use	0.325845	882215	882215	204E11	0.000000443	0.000000443	1.000000
BMI_WHOAsia_D1	0.000366	0.214603	0.215042	959994	0.002045	0.002043	0.999592
BMI_WHOAsia_D2	0.001766	0.164060	0.166179	24606	0.012915	0.012830	0.997441
CKD_refgt60_D2	0.000187	0.141398	0.141622	1.59E6	0.001586	0.001585	0.999683
CKD_refgt60_D3	0.002237	0.339319	0.342003	64945	0.007910	0.007879	0.998427
low-intensity statin (vs. moderate-intensity)	0.000698	0.536498	0.537336	1.65E6	0.001562	0.001560	0.999688

phregparms_lipid_mi_4099

The MIANALYZE Procedure

Parameter Estimates							
Parameter	Estimate	Std Error	95% Confidence Limits		DF	Minimum	Maximum
age	0.015156	0.017607	-0.02	0.050	235064	0.013602	0.016475
MALE (VS. FEMALE)	-0.393258	0.460971	-1.30	0.510	899567	-0.409154	-0.365037
History of HTN	0.433900	0.433796	-0.42	1.284	585930	0.414186	0.465331
History of DM	0.512928	0.353290	-0.18	1.205	113000	0.488663	0.554884
History of HF	0.788183	0.398442	0.01	1.569	541931	0.770457	0.819852
History of MI	1.589948	1.065334	-0.50	3.678	1.25E7	1.552260	1.610374
Previous coronary/LEAD intervention	0.499611	0.443429	-0.37	1.369	224798	0.483289	0.545349
History of ischemic stroke/ TIA	0.333904	0.511347	-0.67	1.336	62084	0.284143	0.391180
Cigarette smoking history	0.526718	0.420226	-0.30	1.350	608293	0.497470	0.551471
fibrate_use	-13.614368	939.263123	-1854.54	1827.308	204E11	-14.077978	-12.971477
BMI_WHOAsia_D1	0.314610	0.463726	-0.59	1.223	959994	0.295788	0.345877
BMI_WHOAsia_D2	-0.267581	0.407651	-1.07	0.531	24606	-0.325749	-0.208022
CKD_refgt60_D2	0.660218	0.376327	-0.08	1.398	1.59E6	0.643545	0.678546
CKD_refgt60_D3	0.895810	0.584810	-0.25	2.042	64945	0.842435	0.953003
low-intensity statin (vs. moderate-intensity)	-0.854710	0.733032	-2.29	0.582	1.65E6	-0.888260	-0.823900

phregparms_lipid_mi_4099

The MIANALYZE Procedure

Parameter Estimates			
Parameter	Theta0	t for H0: Parameter=Theta0	Pr > |t|
age	0	0.86	0.3894
MALE (VS. FEMALE)	0	-0.85	0.3936
History of HTN	0	1.00	0.3172
History of DM	0	1.45	0.1465
History of HF	0	1.98	0.0479
History of MI	0	1.49	0.1356
Previous coronary/LEAD intervention	0	1.13	0.2599
History of ischemic stroke/ TIA	0	0.65	0.5138
Cigarette smoking history	0	1.25	0.2101
fibrate_use	0	-0.01	0.9884
BMI_WHOAsia_D1	0	0.68	0.4975
BMI_WHOAsia_D2	0	-0.66	0.5116
CKD_refgt60_D2	0	1.75	0.0794
CKD_refgt60_D3	0	1.53	0.1256
low-intensity statin (vs. moderate-intensity)	0	-1.17	0.2436

phregparms_mianalyze_mi_4099

Obs	Parm	Estimate	EST_exp	LCLMean_exp	UCLMean_exp	Probt
1	age	0.015156	1.01527	0.98083	1.0509	0.3894
2	MALE (VS. FEMALE)	-0.393258	0.67485	0.27342	1.6657	0.3936
3	History of HTN	0.433900	1.54326	0.65947	3.6115	0.3172
4	History of DM	0.512928	1.67017	0.83567	3.3380	0.1465
5	History of HF	0.788183	2.19940	1.00728	4.8024	0.0479
6	History of MI	1.589948	4.90349	0.60770	39.5658	0.1356
7	Previous coronary/LEAD intervention	0.499611	1.64808	0.69108	3.9303	0.2599
8	History of ischemic stroke/ TIA	0.333904	1.39641	0.51256	3.8044	0.5138
9	Cigarette smoking history	0.526718	1.69337	0.74311	3.8588	0.2101
10	fibrate_use	-13.614368	0.00000	0.00000	.	0.9884
11	BMI_WHOAsia_D1	0.314610	1.36973	0.55196	3.3991	0.4975
12	BMI_WHOAsia_D2	-0.267581	0.76523	0.34418	1.7014	0.5116
13	CKD_refgt60_D2	0.660218	1.93521	0.92555	4.0463	0.0794
14	CKD_refgt60_D3	0.895810	2.44932	0.77848	7.7063	0.1256
15	low-intensity statin (vs. moderate-intensity)	-0.854710	0.42541	0.10112	1.7896	0.2436

derive.out_lipid_mi_4099

The PHREG Procedure

Imputation Number=1

Model Information		
Data Set	WORK.OUT_LIPID_MI_4099	
Dependent Variable	Prim_Outc_Time	
Censoring Variable	Prim_Outc	Prim_Outc
Censoring Value(s)	0	
Ties Handling	BRESLOW	

Number of Observations Read	4099
Number of Observations Used	1991

Summary of the Number of Event and Censored Values			
Total	Event	Censored	Percent Censored
1991	52	1939	97.39

Convergence Status
Convergence criterion (GCONV=1E-8) satisfied.

Model Fit Statistics		
Criterion	Without Covariates	With Covariates
-2 LOG L	723.314	692.781
AIC	723.314	722.781
SBC	723.314	752.050

Testing Global Null Hypothesis: BETA=0			
Test	Chi-Square	DF	Pr > ChiSq
Likelihood Ratio	30.5321	15	0.0101
Score	38.7813	15	0.0007
Wald	35.0855	15	0.0024

derive.out_lipid_mi_4099

The PHREG Procedure

Imputation Number=1

Analysis of Maximum Likelihood Estimates									
Parameter	DF	Parameter Estimate	Standard Error	Chi-Square	Pr > ChiSq	Hazard Ratio	95% Hazard Ratio Confidence Limits		Label
Age	1	0.01306	0.01394	0.8776	0.3488	1.013	0.986	1.041	
Male (vs. female)	1	-0.42678	0.35150	1.4742	0.2247	0.653	0.328	1.300	Male (vs. female)
History of HTN	1	0.12709	0.34035	0.1394	0.7088	1.136	0.583	2.213	History of HTN
History of DM	1	0.13549	0.29017	0.2180	0.6406	1.145	0.648	2.022	
History of HF	1	0.94827	0.31223	9.2242	0.0024	2.581	1.400	4.760	History of HF
History of MI	1	0.83002	0.51222	2.6258	0.1051	2.293	0.840	6.259	
Previous coronary/LEAD intervention	1	0.13870	0.32314	0.1842	0.6678	1.149	0.610	2.164	
History of ischemic stroke/ TIA	1	0.51293	0.39915	1.6514	0.1988	1.670	0.764	3.652	
Cigarette smoking history	1	0.18030	0.33403	0.2914	0.5893	1.198	0.622	2.305	Cigarette smoking history
fibrate_use	1	-0.00741	0.61728	0.0001	0.9904	0.993	0.296	3.328	
BMI_WHOAsia_D1	1	0.26154	0.39878	0.4302	0.5119	1.299	0.594	2.838	
BMI_WHOAsia_D2	1	-0.02409	0.33691	0.0051	0.9430	0.976	0.504	1.889	
CKD_refgt60_D2	1	0.39579	0.31770	1.5520	0.2128	1.486	0.797	2.769	
CKD_refgt60_D3	1	1.14603	0.44283	6.6977	0.0097	3.146	1.321	7.493	
Not under statin (vs. moderate-intensity)	1	0.22857	0.32094	0.5072	0.4764	1.257	0.670	2.358	

derive.out_lipid_mi_4099

The PHREG Procedure

Imputation Number=2

Model Information		
Data Set	WORK.OUT_LIPID_MI_4099	
Dependent Variable	Prim_Outc_Time	
Censoring Variable	Prim_Outc	Prim_Outc
Censoring Value(s)	0	
Ties Handling	BRESLOW	

Number of Observations Read	4099
Number of Observations Used	1994

Summary of the Number of Event and Censored Values			
Total	Event	Censored	Percent Censored
1994	52	1942	97.39

Convergence Status
Convergence criterion (GCONV=1E-8) satisfied.

Model Fit Statistics		
Criterion	Without Covariates	With Covariates
-2 LOG L	722.785	692.431
AIC	722.785	722.431
SBC	722.785	751.700

Testing Global Null Hypothesis: BETA=0			
Test	Chi-Square	DF	Pr > ChiSq
Likelihood Ratio	30.3538	15	0.0107
Score	38.4084	15	0.0008
Wald	34.8797	15	0.0026

derive.out_lipid_mi_4099

The PHREG Procedure

Imputation Number=2

Analysis of Maximum Likelihood Estimates									
Parameter	DF	Parameter Estimate	Standard Error	Chi-Square	Pr > ChiSq	Hazard Ratio	95% Hazard Ratio Confidence Limits		Label
Age	1	0.01403	0.01399	1.0056	0.3160	1.014	0.987	1.042	
Male (vs. female)	1	-0.47350	0.34849	1.8462	0.1742	0.623	0.315	1.233	Male (vs. female)
History of HTN	1	0.13061	0.34074	0.1469	0.7015	1.140	0.584	2.222	History of HTN
History of DM	1	0.10718	0.29048	0.1361	0.7122	1.113	0.630	1.967	
History of HF	1	0.98881	0.31137	10.0853	0.0015	2.688	1.460	4.948	History of HF
History of MI	1	0.86310	0.51315	2.8290	0.0926	2.371	0.867	6.481	
Previous coronary/LEAD intervention	1	0.15711	0.32246	0.2374	0.6261	1.170	0.622	2.201	
History of ischemic stroke/ TIA	1	0.52153	0.39975	1.7021	0.1920	1.685	0.770	3.688	
Cigarette smoking history	1	0.19869	0.33124	0.3598	0.5486	1.220	0.637	2.335	Cigarette smoking history
fibrate_use	1	-0.00889	0.61646	0.0002	0.9885	0.991	0.296	3.318	
BMI_WHOAsia_D1	1	0.02066	0.40343	0.0026	0.9592	1.021	0.463	2.251	
BMI_WHOAsia_D2	1	-0.09109	0.33095	0.0758	0.7831	0.913	0.477	1.746	
CKD_refgt60_D2	1	0.38273	0.31862	1.4429	0.2297	1.466	0.785	2.738	
CKD_refgt60_D3	1	1.10588	0.44440	6.1927	0.0128	3.022	1.265	7.220	
Not under statin (vs. moderate-intensity)	1	0.25550	0.32050	0.6355	0.4253	1.291	0.689	2.420	

derive.out_lipid_mi_4099

The PHREG Procedure

Imputation Number=3

Model Information		
Data Set	WORK.OUT_LIPID_MI_4099	
Dependent Variable	Prim_Outc_Time	
Censoring Variable	Prim_Outc	Prim_Outc
Censoring Value(s)	0	
Ties Handling	BRESLOW	

Number of Observations Read	4099
Number of Observations Used	2000

Summary of the Number of Event and Censored Values			
Total	Event	Censored	Percent Censored
2000	52	1948	97.40

Convergence Status
Convergence criterion (GCONV=1E-8) satisfied.

Model Fit Statistics		
Criterion	Without Covariates	With Covariates
-2 LOG L	723.085	692.698
AIC	723.085	722.698
SBC	723.085	751.967

Testing Global Null Hypothesis: BETA=0			
Test	Chi-Square	DF	Pr > ChiSq
Likelihood Ratio	30.3867	15	0.0106
Score	39.5288	15	0.0005
Wald	35.3838	15	0.0022

derive.out_lipid_mi_4099

The PHREG Procedure

Imputation Number=3

Analysis of Maximum Likelihood Estimates									
Parameter	DF	Parameter Estimate	Standard Error	Chi-Square	Pr > ChiSq	Hazard Ratio	95% Hazard Ratio Confidence Limits		Label
Age	1	0.01400	0.01384	1.0237	0.3116	1.014	0.987	1.042	
Male (vs. female)	1	-0.44522	0.34863	1.6309	0.2016	0.641	0.324	1.269	Male (vs. female)
History of HTN	1	0.10613	0.34127	0.0967	0.7558	1.112	0.570	2.171	History of HTN
History of DM	1	0.08959	0.29167	0.0943	0.7587	1.094	0.617	1.937	
History of HF	1	0.94716	0.31291	9.1623	0.0025	2.578	1.396	4.761	History of HF
History of MI	1	0.85188	0.51148	2.7740	0.0958	2.344	0.860	6.388	
Previous coronary/LEAD intervention	1	0.14902	0.31829	0.2192	0.6396	1.161	0.622	2.166	
History of ischemic stroke/ TIA	1	0.51637	0.39954	1.6703	0.1962	1.676	0.766	3.667	
Cigarette smoking history	1	0.21366	0.33145	0.4155	0.5192	1.238	0.647	2.371	Cigarette smoking history
fibrate_use	1	0.05922	0.61788	0.0092	0.9236	1.061	0.316	3.562	
BMI_WHOAsia_D1	1	0.13250	0.40763	0.1057	0.7451	1.142	0.514	2.538	
BMI_WHOAsia_D2	1	-0.00439	0.33320	0.0002	0.9895	0.996	0.518	1.913	
CKD_refgt60_D2	1	0.37656	0.32004	1.3844	0.2393	1.457	0.778	2.729	
CKD_refgt60_D3	1	1.20003	0.44976	7.1190	0.0076	3.320	1.375	8.017	
Not under statin (vs. moderate-intensity)	1	0.22576	0.31886	0.5013	0.4789	1.253	0.671	2.341	

derive.out_lipid_mi_4099

The PHREG Procedure

Imputation Number=4

Model Information		
Data Set	WORK.OUT_LIPID_MI_4099	
Dependent Variable	Prim_Outc_Time	
Censoring Variable	Prim_Outc	Prim_Outc
Censoring Value(s)	0	
Ties Handling	BRESLOW	

Number of Observations Read	4099
Number of Observations Used	1989

Summary of the Number of Event and Censored Values			
Total	Event	Censored	Percent Censored
1989	52	1937	97.39

Convergence Status
Convergence criterion (GCONV=1E-8) satisfied.

Model Fit Statistics		
Criterion	Without Covariates	With Covariates
-2 LOG L	722.963	693.200
AIC	722.963	723.200
SBC	722.963	752.469

Testing Global Null Hypothesis: BETA=0			
Test	Chi-Square	DF	Pr > ChiSq
Likelihood Ratio	29.7630	15	0.0128
Score	37.7820	15	0.0010
Wald	34.0854	15	0.0033

derive.out_lipid_mi_4099

The PHREG Procedure

Imputation Number=4

Analysis of Maximum Likelihood Estimates									
Parameter	DF	Parameter Estimate	Standard Error	Chi-Square	Pr > ChiSq	Hazard Ratio	95% Hazard Ratio Confidence Limits		Label
Age	1	0.01324	0.01397	0.8976	0.3434	1.013	0.986	1.041	
Male (vs. female)	1	-0.41722	0.35237	1.4019	0.2364	0.659	0.330	1.314	Male (vs. female)
History of HTN	1	0.13670	0.34133	0.1604	0.6888	1.146	0.587	2.238	History of HTN
History of DM	1	0.09535	0.29147	0.1070	0.7436	1.100	0.621	1.948	
History of HF	1	0.91198	0.31198	8.5449	0.0035	2.489	1.351	4.588	History of HF
History of MI	1	0.81702	0.51370	2.5295	0.1117	2.264	0.827	6.196	
Previous coronary/LEAD intervention	1	0.14901	0.32171	0.2145	0.6432	1.161	0.618	2.181	
History of ischemic stroke/ TIA	1	0.47440	0.39765	1.4233	0.2329	1.607	0.737	3.504	
Cigarette smoking history	1	0.18320	0.33271	0.3032	0.5819	1.201	0.626	2.305	Cigarette smoking history
fibrate_use	1	0.00536	0.61440	0.0001	0.9930	1.005	0.302	3.352	
BMI_WHOAsia_D1	1	0.21448	0.40046	0.2868	0.5923	1.239	0.565	2.717	
BMI_WHOAsia_D2	1	-0.05146	0.33605	0.0234	0.8783	0.950	0.492	1.835	
CKD_refgt60_D2	1	0.41141	0.31828	1.6708	0.1962	1.509	0.809	2.816	
CKD_refgt60_D3	1	1.12783	0.44779	6.3437	0.0118	3.089	1.284	7.430	
Not under statin (vs. moderate-intensity)	1	0.27209	0.32115	0.7178	0.3969	1.313	0.700	2.463	

derive.out_lipid_mi_4099

The PHREG Procedure

Imputation Number=5

Model Information		
Data Set	WORK.OUT_LIPID_MI_4099	
Dependent Variable	Prim_Outc_Time	
Censoring Variable	Prim_Outc	Prim_Outc
Censoring Value(s)	0	
Ties Handling	BRESLOW	

Number of Observations Read	4099
Number of Observations Used	1971

Summary of the Number of Event and Censored Values			
Total	Event	Censored	Percent Censored
1971	52	1919	97.36

Convergence Status
Convergence criterion (GCONV=1E-8) satisfied.

Model Fit Statistics		
Criterion	Without Covariates	With Covariates
-2 LOG L	721.643	691.883
AIC	721.643	721.883
SBC	721.643	751.152

Testing Global Null Hypothesis: BETA=0			
Test	Chi-Square	DF	Pr > ChiSq
Likelihood Ratio	29.7605	15	0.0128
Score	37.4438	15	0.0011
Wald	34.0386	15	0.0034

derive.out_lipid_mi_4099

The PHREG Procedure

Imputation Number=5

Analysis of Maximum Likelihood Estimates									
Parameter	DF	Parameter Estimate	Standard Error	Chi-Square	Pr > ChiSq	Hazard Ratio	95% Hazard Ratio Confidence Limits		Label
Age	1	0.01483	0.01380	1.1548	0.2825	1.015	0.988	1.043	
Male (vs. female)	1	-0.43779	0.34954	1.5687	0.2104	0.645	0.325	1.281	Male (vs. female)
History of HTN	1	0.10750	0.34091	0.0994	0.7525	1.113	0.571	2.172	History of HTN
History of DM	1	0.10744	0.29025	0.1370	0.7113	1.113	0.630	1.967	
History of HF	1	0.90962	0.31243	8.4763	0.0036	2.483	1.346	4.581	History of HF
History of MI	1	0.85008	0.51279	2.7482	0.0974	2.340	0.856	6.392	
Previous coronary/LEAD intervention	1	0.10713	0.31872	0.1130	0.7368	1.113	0.596	2.079	
History of ischemic stroke/ TIA	1	0.51929	0.39786	1.7035	0.1918	1.681	0.771	3.666	
Cigarette smoking history	1	0.20573	0.33027	0.3880	0.5333	1.228	0.643	2.347	Cigarette smoking history
fibrate_use	1	0.03582	0.61503	0.0034	0.9536	1.036	0.310	3.460	
BMI_WHOAsia_D1	1	0.18202	0.40946	0.1976	0.6567	1.200	0.538	2.677	
BMI_WHOAsia_D2	1	0.01629	0.33382	0.0024	0.9611	1.016	0.528	1.955	
CKD_refgt60_D2	1	0.42448	0.31768	1.7854	0.1815	1.529	0.820	2.849	
CKD_refgt60_D3	1	1.10798	0.44666	6.1532	0.0131	3.028	1.262	7.268	
Not under statin (vs. moderate-intensity)	1	0.26417	0.31926	0.6847	0.4080	1.302	0.697	2.435	

phregparms_lipid_mi_4099

The MIANALYZE Procedure

Model Information	
PARMS Data Set	WORK.PHREGPARMS_LIPID_MI_4099
Number of Imputations	5

Variance Information							
Parameter	Variance			DF	Relative Increase in Variance	Fraction Missing Information	Relative Efficiency
	Between	Within	Total				
age	0.000000504	0.000193	0.000194	411333	0.003128	0.003123	0.999376
MALE (VS. FEMALE)	0.000462	0.122576	0.123130	197364	0.004522	0.004512	0.999098
History of HTN	0.000194	0.116226	0.116460	997233	0.002007	0.002005	0.999599
History of DM	0.000313	0.084569	0.084945	205047	0.004436	0.004426	0.999115
History of HF	0.001051	0.097459	0.098720	24516	0.012939	0.012854	0.997436
History of MI	0.000344	0.262828	0.263240	1.63E6	0.001569	0.001568	0.999686
Previous coronary/LEAD intervention	0.000384	0.102957	0.103418	201097	0.004480	0.004470	0.999107
History of ischemic stroke/ TIA	0.000382	0.159036	0.159495	483273	0.002885	0.002881	0.999424
Cigarette smoking history	0.000206	0.110186	0.110434	799074	0.002242	0.002240	0.999552
fibrate_use	0.000885	0.379715	0.380777	514544	0.002796	0.002792	0.999442
BMI_WHOAsia_D1	0.008477	0.163196	0.173369	1161.8	0.062335	0.060294	0.988085
BMI_WHOAsia_D2	0.001755	0.111684	0.113791	11672	0.018862	0.018681	0.996278
CKD_refgt60_D2	0.000395	0.101421	0.101895	185175	0.004669	0.004658	0.999069
CKD_refgt60_D3	0.001487	0.199178	0.200963	50740	0.008958	0.008918	0.998220
Not under statin (vs. moderate-intensity)	0.000441	0.102493	0.103022	151787	0.005160	0.005147	0.998972

phregparms_lipid_mi_4099

The MIANALYZE Procedure

Parameter Estimates							
Parameter	Estimate	Std Error	95% Confidence Limits		DF	Minimum	Maximum
age	0.013832	0.013930	-0.01347	0.041134	411333	0.013057	0.014831
MALE (VS. FEMALE)	-0.440101	0.350899	-1.12785	0.247652	197364	-0.473498	-0.417222
History of HTN	0.121609	0.341262	-0.54725	0.790471	997233	0.106134	0.136704
History of DM	0.107007	0.291452	-0.46423	0.678247	205047	0.089586	0.135486
History of HF	0.941169	0.314197	0.32532	1.557015	24516	0.909624	0.988814
History of MI	0.842419	0.513069	-0.16318	1.848017	1.63E6	0.817019	0.863103
Previous coronary/LEAD intervention	0.140193	0.321587	-0.49011	0.770497	201097	0.107128	0.157110
History of ischemic stroke/ TIA	0.508904	0.399368	-0.27384	1.291653	483273	0.474402	0.521526
Cigarette smoking history	0.196317	0.332315	-0.45501	0.847644	799074	0.180300	0.213660
fibrate_use	0.016819	0.617071	-1.19262	1.226259	514544	-0.008894	0.059224
BMI_WHOAsia_D1	0.162242	0.416375	-0.65469	0.979174	1161.8	0.020664	0.261544
BMI_WHOAsia_D2	-0.030946	0.337329	-0.69217	0.630276	11672	-0.091089	0.016295
CKD_refgt60_D2	0.398196	0.319210	-0.22745	1.023840	185175	0.376564	0.424483
CKD_refgt60_D3	1.137549	0.448289	0.25890	2.016199	50740	1.105882	1.200031
Not under statin (vs. moderate-intensity)	0.249218	0.320970	-0.37988	0.878313	151787	0.225762	0.272092

phregparms_lipid_mi_4099

The MIANALYZE Procedure

Parameter Estimates			
Parameter	Theta0	t for H0: Parameter=Theta0	Pr > |t|
age	0	0.99	0.3207
MALE (VS. FEMALE)	0	-1.25	0.2098
History of HTN	0	0.36	0.7216
History of DM	0	0.37	0.7135
History of HF	0	3.00	0.0027
History of MI	0	1.64	0.1006
Previous coronary/LEAD intervention	0	0.44	0.6629
History of ischemic stroke/ TIA	0	1.27	0.2026
Cigarette smoking history	0	0.59	0.5547
fibrate_use	0	0.03	0.9783
BMI_WHOAsia_D1	0	0.39	0.6969
BMI_WHOAsia_D2	0	-0.09	0.9269
CKD_refgt60_D2	0	1.25	0.2122
CKD_refgt60_D3	0	2.54	0.0112
Not under statin (vs. moderate-intensity)	0	0.78	0.4375

phregparms_mianalyze_mi_4099

Obs	Parm	Estimate	EST_exp	LCLMean_exp	UCLMean_exp	Probt
1	age	0.013832	1.01393	0.98662	1.04199	0.3207
2	MALE (VS. FEMALE)	-0.440101	0.64397	0.32373	1.28101	0.2098
3	History of HTN	0.121609	1.12931	0.57854	2.20443	0.7216
4	History of DM	0.107007	1.11294	0.62862	1.97042	0.7135
5	History of HF	0.941169	2.56298	1.38448	4.74464	0.0027
6	History of MI	0.842419	2.32198	0.84944	6.34722	0.1006
7	Previous coronary/LEAD intervention	0.140193	1.15050	0.61256	2.16084	0.6629
8	History of ischemic stroke/ TIA	0.508904	1.66347	0.76045	3.63879	0.2026
9	Cigarette smoking history	0.196317	1.21691	0.63444	2.33414	0.5547
10	fibrate_use	0.016819	1.01696	0.30343	3.40846	0.9783
11	BMI_WHOAsia_D1	0.162242	1.17614	0.51960	2.66226	0.6969
12	BMI_WHOAsia_D2	-0.030946	0.96953	0.50049	1.87813	0.9269
13	CKD_refgt60_D2	0.398196	1.48914	0.79656	2.78386	0.2122
14	CKD_refgt60_D3	1.137549	3.11911	1.29550	7.50973	0.0112
15	Not under statin (vs. moderate-intensity)	0.249218	1.28302	0.68395	2.40684	0.4375

derive.out_lipid_mi_4099

The PHREG Procedure

Imputation Number=1

Model Information		
Data Set	WORK.OUT_LIPID_MI_4099	
Dependent Variable	Prim_Outc_Time	
Censoring Variable	Prim_Outc	Prim_Outc
Censoring Value(s)	0	
Ties Handling	BRESLOW	

Number of Observations Read	4099
Number of Observations Used	4099

Summary of the Number of Event and Censored Values			
Total	Event	Censored	Percent Censored
4099	109	3990	97.34

Convergence Status
Convergence criterion (GCONV=1E-8) satisfied.

Model Fit Statistics		
Criterion	Without Covariates	With Covariates
-2 LOG L	1689.539	1623.665
AIC	1689.539	1659.665
SBC	1689.539	1708.109

Testing Global Null Hypothesis: BETA=0			
Test	Chi-Square	DF	Pr > ChiSq
Likelihood Ratio	65.8736	18	<.0001
Score	77.6169	18	<.0001
Wald	70.9334	18	<.0001

derive.out_lipid_mi_4099

The PHREG Procedure

Imputation Number=1

Analysis of Maximum Likelihood Estimates									
Parameter	DF	Parameter Estimate	Standard Error	Chi-Square	Pr > ChiSq	Hazard Ratio	95% Hazard Ratio Confidence Limits		Label
Age	1	0.01004	0.00948	1.1232	0.2892	1.010	0.992	1.029	
Male (vs. female)	1	-0.42064	0.25542	2.7120	0.0996	0.657	0.398	1.083	Male (vs. female)
History of HTN	1	0.25431	0.23881	1.1341	0.2869	1.290	0.808	2.059	History of HTN
History of DM	1	0.47718	0.19973	5.7078	0.0169	1.612	1.089	2.384	
History of HF	1	0.81238	0.22764	12.7352	0.0004	2.253	1.442	3.520	History of HF
History of MI	1	0.61344	0.34996	3.0727	0.0796	1.847	0.930	3.667	
Previous coronary/LEAD intervention	1	0.01704	0.22127	0.0059	0.9386	1.017	0.659	1.569	
History of ischemic stroke/ TIA	1	-0.04600	0.29964	0.0236	0.8780	0.955	0.531	1.718	
Cigarette smoking history	1	0.50392	0.23668	4.5329	0.0332	1.655	1.041	2.632	Cigarette smoking history
statin_nouse_D0	1	0.09142	0.23188	0.1554	0.6934	1.096	0.696	1.726	
statin_low_D1	1	-0.80619	0.46570	2.9969	0.0834	0.447	0.179	1.112	
statin_high_D3	1	-0.99823	0.71922	1.9263	0.1652	0.369	0.090	1.509	
fibrate_use	1	-0.22588	0.47247	0.2286	0.6326	0.798	0.316	2.014	
BMI_WHOAsia_D1	1	0.24520	0.28075	0.7628	0.3824	1.278	0.737	2.215	
BMI_WHOAsia_D2	1	0.06804	0.22802	0.0890	0.7654	1.070	0.685	1.674	
CKD_refgt60_D2	1	0.45793	0.21997	4.3339	0.0374	1.581	1.027	2.433	
CKD_refgt60_D3	1	1.05889	0.30904	11.7404	0.0006	2.883	1.573	5.283	
LDL-C ≥ 100 mg/dL (vs. <100)	1	0.34016	0.19502	3.0424	0.0811	1.405	0.959	2.059	

derive.out_lipid_mi_4099

The PHREG Procedure

Imputation Number=2

Model Information		
Data Set	WORK.OUT_LIPID_MI_4099	
Dependent Variable	Prim_Outc_Time	
Censoring Variable	Prim_Outc	Prim_Outc
Censoring Value(s)	0	
Ties Handling	BRESLOW	

Number of Observations Read	4099
Number of Observations Used	4099

Summary of the Number of Event and Censored Values			
Total	Event	Censored	Percent Censored
4099	109	3990	97.34

Convergence Status
Convergence criterion (GCONV=1E-8) satisfied.

Model Fit Statistics		
Criterion	Without Covariates	With Covariates
-2 LOG L	1689.539	1623.995
AIC	1689.539	1659.995
SBC	1689.539	1708.439

Testing Global Null Hypothesis: BETA=0			
Test	Chi-Square	DF	Pr > ChiSq
Likelihood Ratio	65.5436	18	<.0001
Score	76.9971	18	<.0001
Wald	70.2898	18	<.0001

derive.out_lipid_mi_4099

The PHREG Procedure

Imputation Number=2

Analysis of Maximum Likelihood Estimates									
Parameter	DF	Parameter Estimate	Standard Error	Chi-Square	Pr > ChiSq	Hazard Ratio	95% Hazard Ratio Confidence Limits		Label
Age	1	0.01063	0.00947	1.2623	0.2612	1.011	0.992	1.030	
Male (vs. female)	1	-0.42877	0.25540	2.8183	0.0932	0.651	0.395	1.074	Male (vs. female)
History of HTN	1	0.24016	0.23839	1.0149	0.3137	1.271	0.797	2.029	History of HTN
History of DM	1	0.47807	0.19902	5.7701	0.0163	1.613	1.092	2.383	
History of HF	1	0.79856	0.22765	12.3054	0.0005	2.222	1.422	3.472	History of HF
History of MI	1	0.61981	0.34953	3.1444	0.0762	1.859	0.937	3.687	
Previous coronary/LEAD intervention	1	0.00535	0.22078	0.0006	0.9807	1.005	0.652	1.550	
History of ischemic stroke/ TIA	1	-0.02516	0.29967	0.0071	0.9331	0.975	0.542	1.754	
Cigarette smoking history	1	0.49974	0.23711	4.4421	0.0351	1.648	1.036	2.623	Cigarette smoking history
statin_nouse_D0	1	0.08795	0.23202	0.1437	0.7047	1.092	0.693	1.721	
statin_low_D1	1	-0.79591	0.46577	2.9200	0.0875	0.451	0.181	1.124	
statin_high_D3	1	-0.99244	0.71910	1.9047	0.1676	0.371	0.091	1.517	
fibrate_use	1	-0.26420	0.47240	0.3128	0.5760	0.768	0.304	1.938	
BMI_WHOAsia_D1	1	0.13788	0.28120	0.2404	0.6239	1.148	0.661	1.992	
BMI_WHOAsia_D2	1	-0.00325	0.22479	0.0002	0.9885	0.997	0.642	1.549	
CKD_refgt60_D2	1	0.48304	0.21939	4.8479	0.0277	1.621	1.054	2.492	
CKD_refgt60_D3	1	1.07162	0.30648	12.2258	0.0005	2.920	1.601	5.324	
LDL-C ≥ 100 mg/dL (vs. <100)	1	0.32955	0.19489	2.8594	0.0908	1.390	0.949	2.037	

derive.out_lipid_mi_4099

The PHREG Procedure

Imputation Number=3

Model Information		
Data Set	WORK.OUT_LIPID_MI_4099	
Dependent Variable	Prim_Outc_Time	
Censoring Variable	Prim_Outc	Prim_Outc
Censoring Value(s)	0	
Ties Handling	BRESLOW	

Number of Observations Read	4099
Number of Observations Used	4099

Summary of the Number of Event and Censored Values			
Total	Event	Censored	Percent Censored
4099	109	3990	97.34

Convergence Status
Convergence criterion (GCONV=1E-8) satisfied.

Model Fit Statistics		
Criterion	Without Covariates	With Covariates
-2 LOG L	1689.539	1622.544
AIC	1689.539	1658.544
SBC	1689.539	1706.988

Testing Global Null Hypothesis: BETA=0			
Test	Chi-Square	DF	Pr > ChiSq
Likelihood Ratio	66.9950	18	<.0001
Score	79.8701	18	<.0001
Wald	72.5166	18	<.0001

derive.out_lipid_mi_4099

The PHREG Procedure

Imputation Number=3

Analysis of Maximum Likelihood Estimates									
Parameter	DF	Parameter Estimate	Standard Error	Chi-Square	Pr > ChiSq	Hazard Ratio	95% Hazard Ratio Confidence Limits		Label
Age	1	0.01031	0.00955	1.1662	0.2802	1.010	0.992	1.029	
Male (vs. female)	1	-0.41972	0.25582	2.6919	0.1009	0.657	0.398	1.085	Male (vs. female)
History of HTN	1	0.25294	0.23805	1.1290	0.2880	1.288	0.808	2.053	History of HTN
History of DM	1	0.47646	0.19978	5.6879	0.0171	1.610	1.089	2.382	
History of HF	1	0.80594	0.22782	12.5145	0.0004	2.239	1.432	3.499	History of HF
History of MI	1	0.59985	0.34916	2.9515	0.0858	1.822	0.919	3.612	
Previous coronary/LEAD intervention	1	0.01123	0.22048	0.0026	0.9594	1.011	0.656	1.558	
History of ischemic stroke/ TIA	1	-0.05260	0.29892	0.0310	0.8603	0.949	0.528	1.704	
Cigarette smoking history	1	0.50815	0.23701	4.5970	0.0320	1.662	1.045	2.645	Cigarette smoking history
statin_nouse_D0	1	0.08025	0.23119	0.1205	0.7285	1.084	0.689	1.705	
statin_low_D1	1	-0.84376	0.46633	3.2739	0.0704	0.430	0.172	1.073	
statin_high_D3	1	-0.98555	0.71896	1.8791	0.1704	0.373	0.091	1.527	
fibrate_use	1	-0.21029	0.47156	0.1989	0.6556	0.810	0.322	2.042	
BMI_WHOAsia_D1	1	0.21761	0.27946	0.6063	0.4362	1.243	0.719	2.150	
BMI_WHOAsia_D2	1	0.02069	0.22622	0.0084	0.9271	1.021	0.655	1.591	
CKD_refgt60_D2	1	0.45473	0.22072	4.2443	0.0394	1.576	1.022	2.429	
CKD_refgt60_D3	1	1.13242	0.30857	13.4683	0.0002	3.103	1.695	5.681	
LDL-C ≥ 100 mg/dL (vs. <100)	1	0.34906	0.19509	3.2012	0.0736	1.418	0.967	2.078	

derive.out_lipid_mi_4099

The PHREG Procedure

Imputation Number=4

Model Information		
Data Set	WORK.OUT_LIPID_MI_4099	
Dependent Variable	Prim_Outc_Time	
Censoring Variable	Prim_Outc	Prim_Outc
Censoring Value(s)	0	
Ties Handling	BRESLOW	

Number of Observations Read	4099
Number of Observations Used	4099

Summary of the Number of Event and Censored Values			
Total	Event	Censored	Percent Censored
4099	109	3990	97.34

Convergence Status
Convergence criterion (GCONV=1E-8) satisfied.

Model Fit Statistics		
Criterion	Without Covariates	With Covariates
-2 LOG L	1689.539	1621.516
AIC	1689.539	1657.516
SBC	1689.539	1705.960

Testing Global Null Hypothesis: BETA=0			
Test	Chi-Square	DF	Pr > ChiSq
Likelihood Ratio	68.0226	18	<.0001
Score	81.6098	18	<.0001
Wald	73.9362	18	<.0001

derive.out_lipid_mi_4099

The PHREG Procedure

Imputation Number=4

Analysis of Maximum Likelihood Estimates									
Parameter	DF	Parameter Estimate	Standard Error	Chi-Square	Pr > ChiSq	Hazard Ratio	95% Hazard Ratio Confidence Limits		Label
Age	1	0.00998	0.00948	1.1088	0.2923	1.010	0.991	1.029	
Male (vs. female)	1	-0.41777	0.25581	2.6672	0.1024	0.659	0.399	1.087	Male (vs. female)
History of HTN	1	0.24678	0.23879	1.0680	0.3014	1.280	0.802	2.044	History of HTN
History of DM	1	0.48236	0.19892	5.8804	0.0153	1.620	1.097	2.392	
History of HF	1	0.80736	0.22779	12.5626	0.0004	2.242	1.435	3.504	History of HF
History of MI	1	0.60694	0.34723	3.0552	0.0805	1.835	0.929	3.624	
Previous coronary/LEAD intervention	1	0.02232	0.22034	0.0103	0.9193	1.023	0.664	1.575	
History of ischemic stroke/ TIA	1	-0.03664	0.29733	0.0152	0.9019	0.964	0.538	1.727	
Cigarette smoking history	1	0.50386	0.23682	4.5267	0.0334	1.655	1.041	2.633	Cigarette smoking history
statin_nouse_D0	1	0.08998	0.23151	0.1510	0.6975	1.094	0.695	1.722	
statin_low_D1	1	-0.80449	0.46560	2.9855	0.0840	0.447	0.180	1.114	
statin_high_D3	1	-0.98847	0.71924	1.8888	0.1693	0.372	0.091	1.524	
fibrate_use	1	-0.23543	0.47181	0.2490	0.6178	0.790	0.313	1.992	
BMI_WHOAsia_D1	1	0.20361	0.27988	0.5293	0.4669	1.226	0.708	2.122	
BMI_WHOAsia_D2	1	0.01230	0.22655	0.0029	0.9567	1.012	0.649	1.578	
CKD_refgt60_D2	1	0.46735	0.21964	4.5273	0.0334	1.596	1.038	2.454	
CKD_refgt60_D3	1	1.18914	0.30815	14.8914	0.0001	3.284	1.795	6.008	
LDL-C ≥ 100 mg/dL (vs. <100)	1	0.36801	0.19526	3.5522	0.0595	1.445	0.985	2.118	

derive.out_lipid_mi_4099

The PHREG Procedure

Imputation Number=5

Model Information		
Data Set	WORK.OUT_LIPID_MI_4099	
Dependent Variable	Prim_Outc_Time	
Censoring Variable	Prim_Outc	Prim_Outc
Censoring Value(s)	0	
Ties Handling	BRESLOW	

Number of Observations Read	4099
Number of Observations Used	4099

Summary of the Number of Event and Censored Values			
Total	Event	Censored	Percent Censored
4099	109	3990	97.34

Convergence Status
Convergence criterion (GCONV=1E-8) satisfied.

Model Fit Statistics		
Criterion	Without Covariates	With Covariates
-2 LOG L	1689.539	1623.461
AIC	1689.539	1659.461
SBC	1689.539	1707.905

Testing Global Null Hypothesis: BETA=0			
Test	Chi-Square	DF	Pr > ChiSq
Likelihood Ratio	66.0783	18	<.0001
Score	78.2593	18	<.0001
Wald	71.4343	18	<.0001

derive.out_lipid_mi_4099

The PHREG Procedure

Imputation Number=5

Analysis of Maximum Likelihood Estimates									
Parameter	DF	Parameter Estimate	Standard Error	Chi-Square	Pr > ChiSq	Hazard Ratio	95% Hazard Ratio Confidence Limits		Label
Age	1	0.00983	0.00950	1.0722	0.3005	1.010	0.991	1.029	
Male (vs. female)	1	-0.43650	0.25610	2.9051	0.0883	0.646	0.391	1.068	Male (vs. female)
History of HTN	1	0.24851	0.23905	1.0808	0.2985	1.282	0.803	2.048	History of HTN
History of DM	1	0.49224	0.19891	6.1244	0.0133	1.636	1.108	2.416	
History of HF	1	0.81689	0.22824	12.8104	0.0003	2.263	1.447	3.540	History of HF
History of MI	1	0.61998	0.34871	3.1610	0.0754	1.859	0.938	3.682	
Previous coronary/LEAD intervention	1	0.01625	0.22071	0.0054	0.9413	1.016	0.659	1.566	
History of ischemic stroke/ TIA	1	-0.04863	0.29908	0.0264	0.8708	0.953	0.530	1.712	
Cigarette smoking history	1	0.49992	0.23691	4.4530	0.0348	1.649	1.036	2.623	Cigarette smoking history
statin_nouse_D0	1	0.09434	0.23189	0.1655	0.6841	1.099	0.698	1.731	
statin_low_D1	1	-0.80924	0.46580	3.0183	0.0823	0.445	0.179	1.109	
statin_high_D3	1	-0.98286	0.71917	1.8678	0.1717	0.374	0.091	1.532	
fibrate_use	1	-0.24011	0.47201	0.2588	0.6110	0.787	0.312	1.984	
BMI_WHOAsia_D1	1	0.20867	0.28490	0.5365	0.4639	1.232	0.705	2.153	
BMI_WHOAsia_D2	1	0.09220	0.22747	0.1643	0.6852	1.097	0.702	1.713	
CKD_refgt60_D2	1	0.45190	0.22029	4.2082	0.0402	1.571	1.020	2.420	
CKD_refgt60_D3	1	1.10202	0.30739	12.8532	0.0003	3.010	1.648	5.499	
LDL-C ≥ 100 mg/dL (vs. <100)	1	0.32721	0.19499	2.8161	0.0933	1.387	0.947	2.033	

phregparms_lipid_mi_4099

The MIANALYZE Procedure

Model Information	
PARMS Data Set	WORK.PHREGPARMS_LIPID_MI_4099
Number of Imputations	5

Variance Information							
Parameter	Variance			DF	Relative Increase in Variance	Fraction Missing Information	Relative Efficiency
	Between	Within	Total				
age	0.000000100	0.000090121	0.000090241	2.25E6	0.001336	0.001335	0.999733
MALE (VS. FEMALE)	0.000061243	0.065387	0.065461	3.17E6	0.001124	0.001123	0.999775
History of HTN	0.000031516	0.056938	0.056976	9.08E6	0.000664	0.000664	0.999867
History of DM	0.000042946	0.039709	0.039760	2.38E6	0.001298	0.001297	0.999741
History of HF	0.000047942	0.051905	0.051963	3.26E6	0.001108	0.001108	0.999778
History of MI	0.000074964	0.121746	0.121836	7.34E6	0.000739	0.000739	0.999852
Previous coronary/LEAD intervention	0.000041299	0.048715	0.048765	3.87E6	0.001017	0.001017	0.999797
History of ischemic stroke/ TIA	0.000121	0.089358	0.089503	1.52E6	0.001626	0.001625	0.999675
Cigarette smoking history	0.000012047	0.056124	0.056139	6.03E7	0.000258	0.000258	0.999948
statin_nouse_D0	0.000028205	0.053684	0.053718	1.01E7	0.000630	0.000630	0.999874
statin_low_D1	0.000341	0.217006	0.217416	1.13E6	0.001888	0.001886	0.999623
statin_high_D3	0.000036392	0.517161	0.517204	5.61E8	0.000084443	0.000084440	0.999983
fibrate_use	0.000393	0.222830	0.223302	896081	0.002117	0.002115	0.999577
BMI_WHOAsia_D1	0.001567	0.079097	0.080977	7420.4	0.023769	0.023481	0.995326
BMI_WHOAsia_D2	0.001625	0.051353	0.053304	2987.4	0.037982	0.037236	0.992608
CKD_refgt60_D2	0.000160	0.048401	0.048593	257799	0.003955	0.003947	0.999211
CKD_refgt60_D3	0.002728	0.094818	0.098091	3592.2	0.034522	0.033907	0.993264
LDL-C ≥ 100 mg/dL (vs. <100)	0.000275	0.038044	0.038374	54082	0.008675	0.008637	0.998276

Parameter Estimates							
Parameter	Estimate	Std Error	95% Confidence Limits		DF	Minimum	Maximum
age	0.010161	0.009500	-0.00846	0.028779	2.25E6	0.009835	0.010635
MALE (VS. FEMALE)	-0.424678	0.255853	-0.92614	0.076786	3.17E6	-0.436495	-0.417773
History of HTN	0.248540	0.238696	-0.21930	0.716376	9.08E6	0.240156	0.254314

phregparms_lipid_mi_4099

The MIANALYZE Procedure

Parameter Estimates							
Parameter	Estimate	Std Error	95% Confidence Limits		DF	Minimum	Maximum
History of DM	0.481262	0.199400	0.09044	0.872079	2.38E6	0.476455	0.492244
History of HF	0.808227	0.227953	0.36145	1.255007	3.26E6	0.798560	0.816894
History of MI	0.612004	0.349050	-0.07212	1.296130	7.34E6	0.599855	0.619981
Previous coronary/LEAD intervention	0.014438	0.220828	-0.41838	0.447253	3.87E6	0.005348	0.022325
History of ischemic stroke/ TIA	-0.041807	0.299171	-0.62817	0.544559	1.52E6	-0.052600	-0.025162
Cigarette smoking history	0.503119	0.236936	0.03873	0.967505	6.03E7	0.499738	0.508155
statin_nouse_D0	0.088785	0.231772	-0.36548	0.543050	1.01E7	0.080246	0.094340
statin_low_D1	-0.811919	0.466278	-1.72581	0.101971	1.13E6	-0.843762	-0.795909
statin_high_D3	-0.989510	0.719169	-2.39906	0.420035	5.61E8	-0.998229	-0.982859
fibrate_use	-0.235182	0.472548	-1.16136	0.690996	896081	-0.264201	-0.210291
BMI_WHOAsia_D1	0.202595	0.284565	-0.35523	0.760422	7420.4	0.137879	0.245203
BMI_WHOAsia_D2	0.037998	0.230876	-0.41469	0.490690	2987.4	-0.003245	0.092202
CKD_refgt60_D2	0.462989	0.220437	0.03094	0.895041	257799	0.451896	0.483043
CKD_refgt60_D3	1.110817	0.313195	0.49676	1.724876	3592.2	1.058886	1.189137
LDL-C ≥ 100 mg/dL (vs. <100)	0.342799	0.195894	-0.04115	0.726752	54082	0.327213	0.368009

Parameter Estimates			
Parameter	Theta0	t for H0: Parameter=Theta0	Pr > |t|
age	0	1.07	0.2848
MALE (VS. FEMALE)	0	-1.66	0.0969
History of HTN	0	1.04	0.2978
History of DM	0	2.41	0.0158
History of HF	0	3.55	0.0004
History of MI	0	1.75	0.0795
Previous coronary/LEAD intervention	0	0.07	0.9479
History of ischemic stroke/ TIA	0	-0.14	0.8889

phregparms_lipid_mi_4099

The MIANALYZE Procedure

Parameter Estimates			
Parameter	Theta0	t for H0: Parameter=Theta0	Pr > |t|
Cigarette smoking history	0	2.12	0.0337
statin_nouse_D0	0	0.38	0.7017
statin_low_D1	0	-1.74	0.0816
statin_high_D3	0	-1.38	0.1689
fibrate_use	0	-0.50	0.6187
BMI_WHOAsia_D1	0	0.71	0.4765
BMI_WHOAsia_D2	0	0.16	0.8693
CKD_refgt60_D2	0	2.10	0.0357
CKD_refgt60_D3	0	3.55	0.0004
LDL-C ≥ 100 mg/dL (vs. <100)	0	1.75	0.0801

phregparms_mianalyze_mi_4099

Obs	Parm	Estimate	EST_exp	LCLMean_exp	UCLMean_exp	Probt
1	age	0.010161	1.01021	0.99158	1.02920	0.2848
2	Male (vs. female)	-0.424678	0.65398	0.39608	1.07981	0.0969
3	History of HTN	0.248540	1.28215	0.80308	2.04700	0.2978
4	History of DM	0.481262	1.61811	1.09466	2.39188	0.0158
5	History of HF	0.808227	2.24393	1.43540	3.50786	0.0004
6	History of MI	0.612004	1.84412	0.93042	3.65512	0.0795
7	Previous coronary/LEAD intervention	0.014438	1.01454	0.65811	1.56401	0.9479
8	History of ischemic stroke/ TIA	-0.041807	0.95906	0.53357	1.72385	0.8889
9	Cigarette Cigarette smoking history history	0.503119	1.65387	1.03949	2.63137	0.0337
10	No statin use (vs. moderate-intensity)	0.088785	1.09285	0.69386	1.72125	0.7017
11	Low-intensity (vs. moderate-intensity)	-0.811919	0.44401	0.17803	1.10735	0.0816
12	High-intensity (vs. moderate-intensity)	-0.989510	0.37176	0.09080	1.52202	0.1689
13	Fibrate_use	-0.235182	0.79043	0.31306	1.99570	0.6187
14	BMI_WHOAsia_D1	0.202595	1.22458	0.70101	2.13918	0.4765
15	BMI_WHOAsia_D2	0.037998	1.03873	0.66054	1.63344	0.8693
16	CKD_refgt60_D2	0.462989	1.58882	1.03142	2.44744	0.0357
17	CKD_refgt60_D3	1.110817	3.03684	1.64339	5.61182	0.0004
18	LDL-C ≥ 100 mg/dL (vs. <100)	0.342799	1.40889	0.95968	2.06835	0.0801

phregparms_mianalyze_mi_4099

The LIFETEST Procedure

Stratum 1: Statin intensity at study enrollment = High-intensity statin dose

Product-Limit Survival Estimates						
Prim_Outc_Time		Survival	Failure	Survival Standard Error	Number Failed	Number Left
0.00000		1.0000	0	0	0	183
0.00500	*	.	.	.	0	182
0.23000	*	.	.	.	0	181
0.23000	*	.	.	.	0	180
0.24900	*	.	.	.	0	179
0.29600	*	.	.	.	0	178
0.34500	*	.	.	.	0	177
0.36700	*	.	.	.	0	176
0.38300	*	.	.	.	0	175
0.38600	*	.	.	.	0	174
0.39200	*	.	.	.	0	173
0.44100	*	.	.	.	0	172
0.44100	*	.	.	.	0	171
0.45700	*	.	.	.	0	170
0.46000	*	.	.	.	0	169
0.46000	*	.	.	.	0	168
0.46800	*	.	.	.	0	167
0.47600	*	.	.	.	0	166
0.47900	*	.	.	.	0	165
0.47900	*	.	.	.	0	164
0.52800	*	.	.	.	0	163
0.56900	*	.	.	.	0	162
0.67600	*	.	.	.	0	161
0.69000	*	.	.	.	0	160
0.69000	*	.	.	.	0	159
0.69000	*	.	.	.	0	158
0.69000	*	.	.	.	0	157
0.69000	*	.	.	.	0	156
0.69000	*	.	.	.	0	155

phregparms_mianalyze_mi_4099

The LIFETEST Procedure

Stratum 1: Statin intensity at study enrollment = High-intensity statin dose

Product-Limit Survival Estimates						
Prim_Outc_Time		Survival	Failure	Survival Standard Error	Number Failed	Number Left
0.69000	*	.	.	.	0	154
0.70900	*	.	.	.	0	153
0.72000	*	.	.	.	0	152
0.72800	*	.	.	.	0	151
0.74700	*	.	.	.	0	150
0.75600	*	.	.	.	0	149
0.77500		0.9933	0.00671	0.00669	1	148
0.78600	*	.	.	.	1	147
0.79400	*	.	.	.	1	146
0.82700	*	.	.	.	1	145
0.84300	*	.	.	.	1	144
0.84300	*	.	.	.	1	143
0.84300	*	.	.	.	1	142
0.90100	*	.	.	.	1	141
0.92000	*	.	.	.	1	140
0.92000	*	.	.	.	1	139
0.92000	*	.	.	.	1	138
0.92000	*	.	.	.	1	137
0.92000	*	.	.	.	1	136
0.92000	*	.	.	.	1	135
0.92000	*	.	.	.	1	134
0.92000	*	.	.	.	1	133
0.92000	*	.	.	.	1	132
0.92000	*	.	.	.	1	131
0.92000	*	.	.	.	1	130
0.92000	*	.	.	.	1	129
0.92000	*	.	.	.	1	128
0.92000	*	.	.	.	1	127
0.93900	*	.	.	.	1	126

phregparms_mianalyze_mi_4099

The LIFETEST Procedure

Stratum 1: Statin intensity at study enrollment = High-intensity statin dose

Product-Limit Survival Estimates						
Prim_Outc_Time		Survival	Failure	Survival Standard Error	Number Failed	Number Left
0.95600	*	.	.	.	1	125
0.95800	*	.	.	.	1	124
0.95800	*	.	.	.	1	123
0.95800	*	.	.	.	1	122
0.97200	*	.	.	.	1	121
0.97700	*	.	.	.	1	120
0.97700	*	.	.	.	1	119
0.97700	*	.	.	.	1	118
0.98600	*	.	.	.	1	117
0.99700	*	.	.	.	1	116
0.99700	*	.	.	.	1	115
1.02900	*	.	.	.	1	114
1.05400	*	.	.	.	1	113
1.06500	*	.	.	.	1	112
1.07300	*	.	.	.	1	111
1.07300	*	.	.	.	1	110
1.07300	*	.	.	.	1	109
1.07300	*	.	.	.	1	108
1.07300	*	.	.	.	1	107
1.07300	*	.	.	.	1	106
1.07900	*	.	.	.	1	105
1.09200	*	.	.	.	1	104
1.13100	*	.	.	.	1	103
1.15000	*	.	.	.	1	102
1.15000	*	.	.	.	1	101
1.15000	*	.	.	.	1	100
1.15000	*	.	.	.	1	99
1.16600	*	.	.	.	1	98
1.18800	*	.	.	.	1	97

phregparms_mianalyze_mi_4099

The LIFETEST Procedure

Stratum 1: Statin intensity at study enrollment = High-intensity statin dose

Product-Limit Survival Estimates						
Prim_Outc_Time		Survival	Failure	Survival Standard Error	Number Failed	Number Left
1.22700	*	.	.	.	1	96
1.55000	*	.	.	.	1	95
1.64800	*	.	.	.	1	94
1.82900	*	.	.	.	1	93
1.84000	*	.	.	.	1	92
1.84000	*	.	.	.	1	91
1.84800	*	.	.	.	1	90
1.89700	*	.	.	.	1	89
1.92700	*	.	.	.	1	88
1.95500		0.9820	0.0180	0.0130	2	87
1.99300	*	.	.	.	2	86
2.03100	*	.	.	.	2	85
2.05600	*	.	.	.	2	84
2.17100	*	.	.	.	2	83
2.17700	*	.	.	.	2	82
2.33800	*	.	.	.	2	81
2.49100	*	.	.	.	2	80
2.50800	*	.	.	.	2	79
2.53000	*	.	.	.	2	78
2.53000	*	.	.	.	2	77
2.53800	*	.	.	.	2	76
2.55200	*	.	.	.	2	75
2.60600	*	.	.	.	2	74
2.68600	*	.	.	.	2	73
2.70200	*	.	.	.	2	72
2.70500	*	.	.	.	2	71
2.71600	*	.	.	.	2	70
2.72100	*	.	.	.	2	69
2.72100	*	.	.	.	2	68

phregparms_mianalyze_mi_4099

The LIFETEST Procedure

Stratum 1: Statin intensity at study enrollment = High-intensity statin dose

Product-Limit Survival Estimates						
Prim_Outc_Time		Survival	Failure	Survival Standard Error	Number Failed	Number Left
2.72100	*	.	.	.	2	67
2.73200	*	.	.	.	2	66
2.73200	*	.	.	.	2	65
2.74100	*	.	.	.	2	64
2.74300	*	.	.	.	2	63
2.76000	*	.	.	.	2	62
2.76000	*	.	.	.	2	61
2.76200	*	.	.	.	2	60
2.76800	*	.	.	.	2	59
2.77100	*	.	.	.	2	58
2.83600	*	.	.	.	2	57
2.87500	*	.	.	.	2	56
2.94300	*	.	.	.	2	55
2.94300	*	.	.	.	2	54
2.99000	*	.	.	.	2	53
2.99000	*	.	.	.	2	52
2.99500	*	.	.	.	2	51
3.00100	*	.	.	.	2	50
3.01400	*	.	.	.	2	49
3.02500	*	.	.	.	2	48
3.02800	*	.	.	.	2	47
3.06600	*	.	.	.	2	46
3.06600	*	.	.	.	2	45
3.09700	*	.	.	.	2	44
3.10500	*	.	.	.	2	43
3.11600	*	.	.	.	2	42
3.12400	*	.	.	.	2	41
3.14000	*	.	.	.	2	40
3.15400	*	.	.	.	2	39

phregparms_mianalyze_mi_4099

The LIFETEST Procedure

Stratum 1: Statin intensity at study enrollment = High-intensity statin dose

Product-Limit Survival Estimates						
Prim_Outc_Time		Survival	Failure	Survival Standard Error	Number Failed	Number Left
3.17600	*	.	.	.	2	38
3.18100	*	.	.	.	2	37
3.19200	*	.	.	.	2	36
3.22000	*	.	.	.	2	35
3.22000	*	.	.	.	2	34
3.23100	*	.	.	.	2	33
3.28800	*	.	.	.	2	32
3.33500	*	.	.	.	2	31
3.34000	*	.	.	.	2	30
3.35400	*	.	.	.	2	29
3.38400	*	.	.	.	2	28
3.60000	*	.	.	.	2	27
3.62200	*	.	.	.	2	26
3.63000	*	.	.	.	2	25
3.65000	*	.	.	.	2	24
3.77500	*	.	.	.	2	23
3.81100	*	.	.	.	2	22
3.85500	*	.	.	.	2	21
3.87100	*	.	.	.	2	20
3.91000	*	.	.	.	2	19
3.92300	*	.	.	.	2	18
3.94800	*	.	.	.	2	17
3.95600	*	.	.	.	2	16
4.01900	*	.	.	.	2	15
4.03000	*	.	.	.	2	14
4.03300	*	.	.	.	2	13
4.04400	*	.	.	.	2	12
4.13100	*	.	.	.	2	11
4.16200	*	.	.	.	2	10

phregparms_mianalyze_mi_4099

The LIFETEST Procedure

Stratum 1: Statin intensity at study enrollment = High-intensity statin dose

Product-Limit Survival Estimates						
Prim_Outc_Time		Survival	Failure	Survival Standard Error	Number Failed	Number Left
4.16400	*	.	.	.	2	9
4.16400	*	.	.	.	2	8
4.17000	*	.	.	.	2	7
4.23500	*	.	.	.	2	6
4.25200	*	.	.	.	2	5
4.25200	*	.	.	.	2	4
4.25200	*	.	.	.	2	3
4.25500	*	.	.	.	2	2
4.37200	*	.	.	.	2	1
4.47900	*	.	.	.	2	0

Note: The marked survival times are censored observations.

phregparms_mianalyze_mi_4099

The LIFETEST Procedure

Stratum 1: Statin intensity at study enrollment = High-intensity statin dose

Summary Statistics for Time Variable Prim_Outc_Time

Quartile Estimates				
	Point	95% Confidence Interval		
Percent	Estimate	Transform	[Lower	Upper)
75	.	LOGLOG	.	.
50	.	LOGLOG	.	.
25	.	LOGLOG	.	.

Mean	Standard Error
1.94708	0.01116

Note: The mean survival time and its standard error were underestimated because the largest observation was censored and the estimation was restricted to the largest event time.

phregparms_mianalyze_mi_4099

The LIFETEST Procedure

Stratum 2: Statin intensity at study enrollment = Low-intensity statin dose

Product-Limit Survival Estimates						
Prim_Outc_Time		Survival	Failure	Survival Standard Error	Number Failed	Number Left
0.00000		1.0000	0	0	0	412
0.00300	*	.	.	.	0	411
0.06000	*	.	.	.	0	410
0.09000	*	.	.	.	0	409
0.22200		0.9976	0.00244	0.00244	1	408
0.22500	*	.	.	.	1	407
0.23000	*	.	.	.	1	406
0.23000	*	.	.	.	1	405
0.23000	*	.	.	.	1	404
0.23800	*	.	.	.	1	403
0.25500	*	.	.	.	1	402
0.28700	*	.	.	.	1	401
0.34800	*	.	.	.	1	400
0.38300	*	.	.	.	1	399
0.38300	*	.	.	.	1	398
0.42700	*	.	.	.	1	397
0.42700	*	.	.	.	1	396
0.44100	*	.	.	.	1	395
0.45200	*	.	.	.	1	394
0.46000	*	.	.	.	1	393
0.46000	*	.	.	.	1	392
0.46000	*	.	.	.	1	391
0.46500	*	.	.	.	1	390
0.53700	*	.	.	.	1	389
0.53700	*	.	.	.	1	388
0.56400	*	.	.	.	1	387
0.59400		0.9950	0.00502	0.00354	2	386
0.61900	*	.	.	.	2	385
0.64300	*	.	.	.	2	384

phregparms_mianalyze_mi_4099

The LIFETEST Procedure

Stratum 2: Statin intensity at study enrollment = Low-intensity statin dose

Product-Limit Survival Estimates						
Prim_Outc_Time		Survival	Failure	Survival Standard Error	Number Failed	Number Left
0.64900	*	.	.	.	2	383
0.65200	*	.	.	.	2	382
0.66800	*	.	.	.	2	381
0.67100	*	.	.	.	2	380
0.67100	*	.	.	.	2	379
0.67100	*	.	.	.	2	378
0.67400	*	.	.	.	2	377
0.68700	*	.	.	.	2	376
0.69000	*	.	.	.	2	375
0.69000	*	.	.	.	2	374
0.69000	*	.	.	.	2	373
0.69000	*	.	.	.	2	372
0.69000	*	.	.	.	2	371
0.69000	*	.	.	.	2	370
0.69000	*	.	.	.	2	369
0.69000	*	.	.	.	2	368
0.69000	*	.	.	.	2	367
0.70100	*	.	.	.	2	366
0.70100	*	.	.	.	2	365
0.70900	*	.	.	.	2	364
0.70900	*	.	.	.	2	363
0.70900	*	.	.	.	2	362
0.71200	*	.	.	.	2	361
0.76400		0.9922	0.00778	0.00448	3	360
0.76400	*	.	.	.	3	359
0.76700	*	.	.	.	3	358
0.76700	*	.	.	.	3	357
0.76700	*	.	.	.	3	356
0.78600	*	.	.	.	3	355

phregparms_mianalyze_mi_4099

The LIFETEST Procedure

Stratum 2: Statin intensity at study enrollment = Low-intensity statin dose

Product-Limit Survival Estimates						
Prim_Outc_Time		Survival	Failure	Survival Standard Error	Number Failed	Number Left
0.78600	*	.	.	.	3	354
0.82400	*	.	.	.	3	353
0.84300	*	.	.	.	3	352
0.84300	*	.	.	.	3	351
0.84300	*	.	.	.	3	350
0.84300	*	.	.	.	3	349
0.84300	*	.	.	.	3	348
0.88200	*	.	.	.	3	347
0.90100	*	.	.	.	3	346
0.90100	*	.	.	.	3	345
0.91200	*	.	.	.	3	344
0.91700	*	.	.	.	3	343
0.92000	*	.	.	.	3	342
0.92000	*	.	.	.	3	341
0.92000	*	.	.	.	3	340
0.92000	*	.	.	.	3	339
0.92000	*	.	.	.	3	338
0.92000	*	.	.	.	3	337
0.92000	*	.	.	.	3	336
0.92000	*	.	.	.	3	335
0.92000	*	.	.	.	3	334
0.92000	*	.	.	.	3	333
0.92000	*	.	.	.	3	332
0.92000	*	.	.	.	3	331
0.92000	*	.	.	.	3	330
0.92000	*	.	.	.	3	329
0.92000	*	.	.	.	3	328
0.92000	*	.	.	.	3	327
0.92000	*	.	.	.	3	326

phregparms_mianalyze_mi_4099

The LIFETEST Procedure

Stratum 2: Statin intensity at study enrollment = Low-intensity statin dose

Product-Limit Survival Estimates						
Prim_Outc_Time		Survival	Failure	Survival Standard Error	Number Failed	Number Left
0.92000	*	.	.	.	3	325
0.92000	*	.	.	.	3	324
0.92000	*	.	.	.	3	323
0.92000	*	.	.	.	3	322
0.92000	*	.	.	.	3	321
0.92000	*	.	.	.	3	320
0.92000	*	.	.	.	3	319
0.92000	*	.	.	.	3	318
0.92000	*	.	.	.	3	317
0.92000	*	.	.	.	3	316
0.92000	*	.	.	.	3	315
0.92000	*	.	.	.	3	314
0.92000	*	.	.	.	3	313
0.92000	*	.	.	.	3	312
0.92000	*	.	.	.	3	311
0.92000	*	.	.	.	3	310
0.92000	*	.	.	.	3	309
0.92000	*	.	.	.	3	308
0.92000	*	.	.	.	3	307
0.92000	*	.	.	.	3	306
0.92000	*	.	.	.	3	305
0.92000	*	.	.	.	3	304
0.92000	*	.	.	.	3	303
0.92000	*	.	.	.	3	302
0.92000	*	.	.	.	3	301
0.92000	*	.	.	.	3	300
0.92000	*	.	.	.	3	299
0.92000	*	.	.	.	3	298
0.92000	*	.	.	.	3	297

phregparms_mianalyze_mi_4099

The LIFETEST Procedure

Stratum 2: Statin intensity at study enrollment = Low-intensity statin dose

Product-Limit Survival Estimates						
Prim_Outc_Time		Survival	Failure	Survival Standard Error	Number Failed	Number Left
0.92300	*	.	.	.	3	296
0.93900	*	.	.	.	3	295
0.93900	*	.	.	.	3	294
0.93900	*	.	.	.	3	293
0.93900	*	.	.	.	3	292
0.93900	*	.	.	.	3	291
0.93900	*	.	.	.	3	290
0.93900	*	.	.	.	3	289
0.93900	*	.	.	.	3	288
0.95800	*	.	.	.	3	287
0.97500	*	.	.	.	3	286
0.97700	*	.	.	.	3	285
0.97700	*	.	.	.	3	284
0.97700	*	.	.	.	3	283
0.98600	*	.	.	.	3	282
0.99700	*	.	.	.	3	281
0.99700	*	.	.	.	3	280
0.99700	*	.	.	.	3	279
0.99700	*	.	.	.	3	278
0.99700	*	.	.	.	3	277
0.99700	*	.	.	.	3	276
0.99700	*	.	.	.	3	275
0.99700	*	.	.	.	3	274
0.99700	*	.	.	.	3	273
0.99700	*	.	.	.	3	272
0.99700	*	.	.	.	3	271
0.99700	*	.	.	.	3	270
1.00800	*	.	.	.	3	269
1.01000	*	.	.	.	3	268

phregparms_mianalyze_mi_4099

The LIFETEST Procedure

Stratum 2: Statin intensity at study enrollment = Low-intensity statin dose

Product-Limit Survival Estimates						
Prim_Outc_Time		Survival	Failure	Survival Standard Error	Number Failed	Number Left
1.01300	*	.	.	.	3	267
1.01600	*	.	.	.	3	266
1.01600	*	.	.	.	3	265
1.01600	*	.	.	.	3	264
1.01600	*	.	.	.	3	263
1.03500	*	.	.	.	3	262
1.03500	*	.	.	.	3	261
1.03800	*	.	.	.	3	260
1.05400	*	.	.	.	3	259
1.07000	*	.	.	.	3	258
1.07300	*	.	.	.	3	257
1.07300	*	.	.	.	3	256
1.07300	*	.	.	.	3	255
1.07300	*	.	.	.	3	254
1.08400	*	.	.	.	3	253
1.09200	*	.	.	.	3	252
1.11200	*	.	.	.	3	251
1.13100	*	.	.	.	3	250
1.14700	*	.	.	.	3	249
1.15000	*	.	.	.	3	248
1.15000	*	.	.	.	3	247
1.15000	*	.	.	.	3	246
1.15000	*	.	.	.	3	245
1.15000	*	.	.	.	3	244
1.15000	*	.	.	.	3	243
1.15000	*	.	.	.	3	242
1.15000	*	.	.	.	3	241
1.15000	*	.	.	.	3	240
1.15000	*	.	.	.	3	239

phregparms_mianalyze_mi_4099

The LIFETEST Procedure

Stratum 2: Statin intensity at study enrollment = Low-intensity statin dose

Product-Limit Survival Estimates						
Prim_Outc_Time		Survival	Failure	Survival Standard Error	Number Failed	Number Left
1.15000	*	.	.	.	3	238
1.15000	*	.	.	.	3	237
1.15000	*	.	.	.	3	236
1.15000	*	.	.	.	3	235
1.15000	*	.	.	.	3	234
1.15000	*	.	.	.	3	233
1.15000	*	.	.	.	3	232
1.15000	*	.	.	.	3	231
1.15000	*	.	.	.	3	230
1.15000	*	.	.	.	3	229
1.15000	*	.	.	.	3	228
1.15000	*	.	.	.	3	227
1.15000	*	.	.	.	3	226
1.15000	*	.	.	.	3	225
1.15000	*	.	.	.	3	224
1.15000	*	.	.	.	3	223
1.16400	*	.	.	.	3	222
1.16900	*	.	.	.	3	221
1.16900	*	.	.	.	3	220
1.18000	*	.	.	.	3	219
1.18300	*	.	.	.	3	218
1.18800	*	.	.	.	3	217
1.18800	*	.	.	.	3	216
1.18800	*	.	.	.	3	215
1.18800	*	.	.	.	3	214
1.20200	*	.	.	.	3	213
1.20700	*	.	.	.	3	212
1.20700	*	.	.	.	3	211
1.21600	*	.	.	.	3	210

phregparms_mianalyze_mi_4099

The LIFETEST Procedure

Stratum 2: Statin intensity at study enrollment = Low-intensity statin dose

Product-Limit Survival Estimates						
Prim_Outc_Time		Survival	Failure	Survival Standard Error	Number Failed	Number Left
1.22700	*	.	.	.	3	209
1.22700	*	.	.	.	3	208
1.24600	*	.	.	.	3	207
1.24800	*	.	.	.	3	206
1.29200	*	.	.	.	3	205
1.32200	*	.	.	.	3	204
1.32200	*	.	.	.	3	203
1.34700	*	.	.	.	3	202
1.35200	*	.	.	.	3	201
1.36100	*	.	.	.	3	200
1.36100	*	.	.	.	3	199
1.38000	*	.	.	.	3	198
1.38500	*	.	.	.	3	197
1.39400	*	.	.	.	3	196
1.39900	*	.	.	.	3	195
1.49200	*	.	.	.	3	194
1.50000	*	.	.	.	3	193
1.57200	*	.	.	.	3	192
1.60200	*	.	.	.	3	191
1.60400	*	.	.	.	3	190
1.61000	*	.	.	.	3	189
1.61000	*	.	.	.	3	188
1.61000	*	.	.	.	3	187
1.61000	*	.	.	.	3	186
1.61300	*	.	.	.	3	185
1.61800	*	.	.	.	3	184
1.62100	*	.	.	.	3	183
1.62900	*	.	.	.	3	182
1.65100	*	.	.	.	3	181

phregparms_mianalyze_mi_4099

The LIFETEST Procedure

Stratum 2: Statin intensity at study enrollment = Low-intensity statin dose

Product-Limit Survival Estimates						
Prim_Outc_Time		Survival	Failure	Survival Standard Error	Number Failed	Number Left
1.66700	*	.	.	.	3	180
1.66700	*	.	.	.	3	179
1.68700	*	.	.	.	3	178
1.69700	*	.	.	.	3	177
1.74400	*	.	.	.	3	176
1.74400	*	.	.	.	3	175
1.77700	*	.	.	.	3	174
1.78200	*	.	.	.	3	173
1.80200	*	.	.	.	3	172
1.80700	*	.	.	.	3	171
1.82600	*	.	.	.	3	170
1.85100	*	.	.	.	3	169
1.85400	*	.	.	.	3	168
1.85900	*	.	.	.	3	167
1.86400		0.9863	0.0137	0.00741	4	166
1.87800	*	.	.	.	4	165
1.92200	*	.	.	.	4	164
1.97400	*	.	.	.	4	163
1.97700	*	.	.	.	4	162
1.98800	*	.	.	.	4	161
1.99300	*	.	.	.	4	160
2.01500	*	.	.	.	4	159
2.05900	*	.	.	.	4	158
2.05900	*	.	.	.	4	157
2.07500	*	.	.	.	4	156
2.17900	*	.	.	.	4	155
2.26700	*	.	.	.	4	154
2.31300	*	.	.	.	4	153
2.31900	*	.	.	.	4	152

phregparms_mianalyze_mi_4099

The LIFETEST Procedure

Stratum 2: Statin intensity at study enrollment = Low-intensity statin dose

Product-Limit Survival Estimates						
Prim_Outc_Time		Survival	Failure	Survival Standard Error	Number Failed	Number Left
2.33800	*	.	.	.	4	151
2.37900	*	.	.	.	4	150
2.42000		0.9797	0.0203	0.00986	5	149
2.42800	*	.	.	.	5	148
2.43100	*	.	.	.	5	147
2.45000	*	.	.	.	5	146
2.45300	*	.	.	.	5	145
2.45300	*	.	.	.	5	144
2.47200	*	.	.	.	5	143
2.49100	*	.	.	.	5	142
2.51100	*	.	.	.	5	141
2.51100	*	.	.	.	5	140
2.51100	*	.	.	.	5	139
2.51100	*	.	.	.	5	138
2.52700	*	.	.	.	5	137
2.53000	*	.	.	.	5	136
2.53000	*	.	.	.	5	135
2.53000	*	.	.	.	5	134
2.53000	*	.	.	.	5	133
2.53000	*	.	.	.	5	132
2.53000	*	.	.	.	5	131
2.53000	*	.	.	.	5	130
2.53000	*	.	.	.	5	129
2.53800	*	.	.	.	5	128
2.53800	*	.	.	.	5	127
2.54900	*	.	.	.	5	126
2.56300	*	.	.	.	5	125
2.56800	*	.	.	.	5	124
2.58700	*	.	.	.	5	123

phregparms_mianalyze_mi_4099

The LIFETEST Procedure

Stratum 2: Statin intensity at study enrollment = Low-intensity statin dose

Product-Limit Survival Estimates						
Prim_Outc_Time		Survival	Failure	Survival Standard Error	Number Failed	Number Left
2.60600	*	.	.	.	5	122
2.60600	*	.	.	.	5	121
2.60600	*	.	.	.	5	120
2.61200	*	.	.	.	5	119
2.62000	*	.	.	.	5	118
2.62600	*	.	.	.	5	117
2.62600	*	.	.	.	5	116
2.62600	*	.	.	.	5	115
2.62600	*	.	.	.	5	114
2.64200	*	.	.	.	5	113
2.64500	*	.	.	.	5	112
2.65300	*	.	.	.	5	111
2.66100	*	.	.	.	5	110
2.66400	*	.	.	.	5	109
2.66400	*	.	.	.	5	108
2.66400	*	.	.	.	5	107
2.66400	*	.	.	.	5	106
2.66700	*	.	.	.	5	105
2.68000	*	.	.	.	5	104
2.68300	*	.	.	.	5	103
2.68300	*	.	.	.	5	102
2.69100	*	.	.	.	5	101
2.70000	*	.	.	.	5	100
2.70200	*	.	.	.	5	99
2.70200	*	.	.	.	5	98
2.70200	*	.	.	.	5	97
2.70200	*	.	.	.	5	96
2.72100	*	.	.	.	5	95
2.72100	*	.	.	.	5	94

phregparms_mianalyze_mi_4099

The LIFETEST Procedure

Stratum 2: Statin intensity at study enrollment = Low-intensity statin dose

Product-Limit Survival Estimates						
Prim_Outc_Time		Survival	Failure	Survival Standard Error	Number Failed	Number Left
2.72100	*	.	.	.	5	93
2.72100	*	.	.	.	5	92
2.72100	*	.	.	.	5	91
2.73200	*	.	.	.	5	90
2.73800	*	.	.	.	5	89
2.73800	*	.	.	.	5	88
2.74100	*	.	.	.	5	87
2.74100	*	.	.	.	5	86
2.74100	*	.	.	.	5	85
2.74100	*	.	.	.	5	84
2.74300	*	.	.	.	5	83
2.75700	*	.	.	.	5	82
2.76000	*	.	.	.	5	81
2.76000	*	.	.	.	5	80
2.76000	*	.	.	.	5	79
2.76000	*	.	.	.	5	78
2.76000	*	.	.	.	5	77
2.76000	*	.	.	.	5	76
2.76000	*	.	.	.	5	75
2.76000	*	.	.	.	5	74
2.77300	*	.	.	.	5	73
2.77900	*	.	.	.	5	72
2.77900	*	.	.	.	5	71
2.79800	*	.	.	.	5	70
2.79800	*	.	.	.	5	69
2.79800	*	.	.	.	5	68
2.79800	*	.	.	.	5	67
2.81500	*	.	.	.	5	66
2.81700	*	.	.	.	5	65

phregparms_mianalyze_mi_4099

The LIFETEST Procedure

Stratum 2: Statin intensity at study enrollment = Low-intensity statin dose

Product-Limit Survival Estimates						
Prim_Outc_Time		Survival	Failure	Survival Standard Error	Number Failed	Number Left
2.81700	*	.	.	.	5	64
2.81700	*	.	.	.	5	63
2.82500	*	.	.	.	5	62
2.83400	*	.	.	.	5	61
2.83600	*	.	.	.	5	60
2.83900	*	.	.	.	5	59
2.84500	*	.	.	.	5	58
2.85600	*	.	.	.	5	57
2.85600	*	.	.	.	5	56
2.85800	*	.	.	.	5	55
2.88300	*	.	.	.	5	54
2.90500	*	.	.	.	5	53
2.91300	*	.	.	.	5	52
2.91300	*	.	.	.	5	51
2.97100	*	.	.	.	5	50
2.97100	*	.	.	.	5	49
2.97100	*	.	.	.	5	48
2.97600	*	.	.	.	5	47
2.98400	*	.	.	.	5	46
2.99000	*	.	.	.	5	45
2.99000	*	.	.	.	5	44
2.99500	*	.	.	.	5	43
2.99500	*	.	.	.	5	42
3.00100	*	.	.	.	5	41
3.00100	*	.	.	.	5	40
3.00600	*	.	.	.	5	39
3.00900	*	.	.	.	5	38
3.00900	*	.	.	.	5	37
3.00900	*	.	.	.	5	36

phregparms_mianalyze_mi_4099

The LIFETEST Procedure

Stratum 2: Statin intensity at study enrollment = Low-intensity statin dose

Product-Limit Survival Estimates						
Prim_Outc_Time		Survival	Failure	Survival Standard Error	Number Failed	Number Left
3.02800	*	.	.	.	5	35
3.03400	*	.	.	.	5	34
3.07500	*	.	.	.	5	33
3.08600	*	.	.	.	5	32
3.17300	*	.	.	.	5	31
3.23900	*	.	.	.	5	30
3.41100	*	.	.	.	5	29
3.45000	*	.	.	.	5	28
3.53500	*	.	.	.	5	27
3.60000	*	.	.	.	5	26
3.60300	*	.	.	.	5	25
3.60300	*	.	.	.	5	24
3.66300	*	.	.	.	5	23
3.68000	*	.	.	.	5	22
3.69900	*	.	.	.	5	21
3.78900	*	.	.	.	5	20
3.81100	*	.	.	.	5	19
3.81100	*	.	.	.	5	18
3.83300	*	.	.	.	5	17
3.83300	*	.	.	.	5	16
3.83800	*	.	.	.	5	15
3.84700	*	.	.	.	5	14
3.88200	*	.	.	.	5	13
3.88800	*	.	.	.	5	12
3.91000	*	.	.	.	5	11
3.91000	*	.	.	.	5	10
3.91000	*	.	.	.	5	9
3.94300	*	.	.	.	5	8
3.99700	*	.	.	.	5	7

phregparms_mianalyze_mi_4099

The LIFETEST Procedure

Stratum 2: Statin intensity at study enrollment = Low-intensity statin dose

Product-Limit Survival Estimates						
Prim_Outc_Time		Survival	Failure	Survival Standard Error	Number Failed	Number Left
4.00000	*	.	.	.	5	6
4.00500	*	.	.	.	5	5
4.02500	*	.	.	.	5	4
4.02500	*	.	.	.	5	3
4.08500	*	.	.	.	5	2
4.10400	*	.	.	.	5	1
4.15900	*	.	.	.	5	0

Note: The marked survival times are censored observations.

phregparms_mianalyze_mi_4099

The LIFETEST Procedure

Stratum 2: Statin intensity at study enrollment = Low-intensity statin dose

Summary Statistics for Time Variable Prim_Outc_Time

Quartile Estimates				
	Point	95% Confidence Interval		
Percent	Estimate	Transform	[Lower	Upper)
75	.	LOGLOG	.	.
50	.	LOGLOG	.	.
25	.	LOGLOG	.	.

Mean	Standard Error
2.40205	0.01012

Note: The mean survival time and its standard error were underestimated because the largest observation was censored and the estimation was restricted to the largest event time.

phregparms_mianalyze_mi_4099

The LIFETEST Procedure

Stratum 3: Statin intensity at study enrollment = Medium-intensity statin dose

Product-Limit Survival Estimates						
Prim_Outc_Time		Survival	Failure	Survival Standard Error	Number Failed	Number Left
0.00000		1.0000	0	0	0	2338
0.00300	*	.	.	.	0	2337
0.00500		0.9996	0.000428	0.000428	1	2336
0.01400		0.9991	0.000856	0.000605	2	2335
0.01600		0.9987	0.00128	0.000741	3	2334
0.01900		0.9983	0.00171	0.000855	4	2333
0.02500		0.9979	0.00214	0.000956	5	2332
0.03800	*	.	.	.	5	2331
0.04100	*	.	.	.	5	2330
0.05700	*	.	.	.	5	2329
0.07100		0.9974	0.00257	0.00105	6	2328
0.08500	*	.	.	.	6	2327
0.08500	*	.	.	.	6	2326
0.08500	*	.	.	.	6	2325
0.10400	*	.	.	.	6	2324
0.10400	*	.	.	.	6	2323
0.10700		0.9970	0.00300	0.00113	7	2322
0.11200	*	.	.	.	7	2321
0.13400		0.9966	0.00343	0.00121	8	2320
0.13700	*	.	.	.	8	2319
0.14000		0.9961	0.00386	0.00128	9	2318
0.15600	*	.	.	.	9	2317
0.15900		0.9957	0.00429	0.00135	10	2316
0.16200	*	.	.	.	10	2315
0.16200	*	.	.	.	10	2314
0.16700	*	.	.	.	10	2313
0.17500	*	.	.	.	10	2312
0.17500	*	.	.	.	10	2311
0.18600		0.9953	0.00472	0.00142	11	2310

phregparms_mianalyze_mi_4099

The LIFETEST Procedure

Stratum 3: Statin intensity at study enrollment = Medium-intensity statin dose

Product-Limit Survival Estimates						
Prim_Outc_Time		Survival	Failure	Survival Standard Error	Number Failed	Number Left
0.20000	*	.	.	.	11	2309
0.20000	*	.	.	.	11	2308
0.21900	*	.	.	.	11	2307
0.22700	*	.	.	.	11	2306
0.23000	*	.	.	.	11	2305
0.23000	*	.	.	.	11	2304
0.23000	*	.	.	.	11	2303
0.23000	*	.	.	.	11	2302
0.23000	*	.	.	.	11	2301
0.23000	*	.	.	.	11	2300
0.23000	*	.	.	.	11	2299
0.23000	*	.	.	.	11	2298
0.23000	*	.	.	.	11	2297
0.23000	*	.	.	.	11	2296
0.23000	*	.	.	.	11	2295
0.23000	*	.	.	.	11	2294
0.23000	*	.	.	.	11	2293
0.23000	*	.	.	.	11	2292
0.23000	*	.	.	.	11	2291
0.23000	*	.	.	.	11	2290
0.23000	*	.	.	.	11	2289
0.23000	*	.	.	.	11	2288
0.23000	*	.	.	.	11	2287
0.23300	*	.	.	.	11	2286
0.23500	*	.	.	.	11	2285
0.23500	*	.	.	.	11	2284
0.23800	*	.	.	.	11	2283
0.23800	*	.	.	.	11	2282
0.24600	*	.	.	.	11	2281

phregparms_mianalyze_mi_4099

The LIFETEST Procedure

Stratum 3: Statin intensity at study enrollment = Medium-intensity statin dose

Product-Limit Survival Estimates						
Prim_Outc_Time		Survival	Failure	Survival Standard Error	Number Failed	Number Left
0.24600	*	.	.	.	11	2280
0.24900	*	.	.	.	11	2279
0.24900	*	.	.	.	11	2278
0.24900	*	.	.	.	11	2277
0.24900	*	.	.	.	11	2276
0.24900	*	.	.	.	11	2275
0.24900	*	.	.	.	11	2274
0.24900	*	.	.	.	11	2273
0.24900	*	.	.	.	11	2272
0.24900	*	.	.	.	11	2271
0.24900	*	.	.	.	11	2270
0.24900	*	.	.	.	11	2269
0.25200	*	.	.	.	11	2268
0.25200	*	.	.	.	11	2267
0.25200	*	.	.	.	11	2266
0.25200	*	.	.	.	11	2265
0.25200	*	.	.	.	11	2264
0.25200	*	.	.	.	11	2263
0.25500	*	.	.	.	11	2262
0.25500	*	.	.	.	11	2261
0.25500	*	.	.	.	11	2260
0.25500	*	.	.	.	11	2259
0.25700	*	.	.	.	11	2258
0.26000	*	.	.	.	11	2257
0.26800	*	.	.	.	11	2256
0.27700		0.9948	0.00516	0.00149	12	2255
0.27700	*	.	.	.	12	2254
0.28200		0.9944	0.00560	0.00155	13	2253
0.28500	*	.	.	.	13	2252

phregparms_mianalyze_mi_4099

The LIFETEST Procedure

Stratum 3: Statin intensity at study enrollment = Medium-intensity statin dose

Product-Limit Survival Estimates						
Prim_Outc_Time		Survival	Failure	Survival Standard Error	Number Failed	Number Left
0.28700	*	.	.	.	13	2251
0.28700	*	.	.	.	13	2250
0.29000	*	.	.	.	13	2249
0.29800	*	.	.	.	13	2248
0.30100	*	.	.	.	13	2247
0.30100	*	.	.	.	13	2246
0.30100	*	.	.	.	13	2245
0.30400	*	.	.	.	13	2244
0.30700	*	.	.	.	13	2243
0.30700	*	.	.	.	13	2242
0.30700	*	.	.	.	13	2241
0.30700	*	.	.	.	13	2240
0.30900	*	.	.	.	13	2239
0.31200	*	.	.	.	13	2238
0.32600	*	.	.	.	13	2237
0.32600	*	.	.	.	13	2236
0.32600	*	.	.	.	13	2235
0.33900	*	.	.	.	13	2234
0.34500	*	.	.	.	13	2233
0.34500	*	.	.	.	13	2232
0.35600	*	.	.	.	13	2231
0.36100		0.9940	0.00605	0.00161	14	2230
0.36100	*	.	.	.	14	2229
0.36400	*	.	.	.	14	2228
0.36700	*	.	.	.	14	2227
0.37800	*	.	.	.	14	2226
0.38100		0.9935	0.00649	0.00167	15	2225
0.38300	*	.	.	.	15	2224
0.38300	*	.	.	.	15	2223

phregparms_mianalyze_mi_4099

The LIFETEST Procedure

Stratum 3: Statin intensity at study enrollment = Medium-intensity statin dose

Product-Limit Survival Estimates						
Prim_Outc_Time		Survival	Failure	Survival Standard Error	Number Failed	Number Left
0.38300	*	.	.	.	15	2222
0.38300	*	.	.	.	15	2221
0.38300	*	.	.	.	15	2220
0.38600	*	.	.	.	15	2219
0.39700		0.9931	0.00694	0.00173	16	2218
0.39700	*	.	.	.	16	2217
0.40800	*	.	.	.	16	2216
0.40800	*	.	.	.	16	2215
0.41100	*	.	.	.	16	2214
0.41100	*	.	.	.	16	2213
0.41300	*	.	.	.	16	2212
0.41600	*	.	.	.	16	2211
0.41900	*	.	.	.	16	2210
0.41900	*	.	.	.	16	2209
0.41900	*	.	.	.	16	2208
0.42200	*	.	.	.	16	2207
0.42200	*	.	.	.	16	2206
0.42200	*	.	.	.	16	2205
0.44100	*	.	.	.	16	2204
0.44100	*	.	.	.	16	2203
0.44100	*	.	.	.	16	2202
0.44100	*	.	.	.	16	2201
0.44100	*	.	.	.	16	2200
0.44100	*	.	.	.	16	2199
0.44100	*	.	.	.	16	2198
0.44100	*	.	.	.	16	2197
0.44100	*	.	.	.	16	2196
0.44100	*	.	.	.	16	2195
0.44100	*	.	.	.	16	2194

phregparms_mianalyze_mi_4099

The LIFETEST Procedure

Stratum 3: Statin intensity at study enrollment = Medium-intensity statin dose

Product-Limit Survival Estimates						
Prim_Outc_Time		Survival	Failure	Survival Standard Error	Number Failed	Number Left
0.44100	*	.	.	.	16	2193
0.44100	*	.	.	.	16	2192
0.44600	*	.	.	.	16	2191
0.44600	*	.	.	.	16	2190
0.44900	*	.	.	.	16	2189
0.45200	*	.	.	.	16	2188
0.45200	*	.	.	.	16	2187
0.46000	*	.	.	.	16	2186
0.46000	*	.	.	.	16	2185
0.46000	*	.	.	.	16	2184
0.46000	*	.	.	.	16	2183
0.46000	*	.	.	.	16	2182
0.46000	*	.	.	.	16	2181
0.46000	*	.	.	.	16	2180
0.46000	*	.	.	.	16	2179
0.46000	*	.	.	.	16	2178
0.46000	*	.	.	.	16	2177
0.46000	*	.	.	.	16	2176
0.46000	*	.	.	.	16	2175
0.46000	*	.	.	.	16	2174
0.46000	*	.	.	.	16	2173
0.46000	*	.	.	.	16	2172
0.46000	*	.	.	.	16	2171
0.46000	*	.	.	.	16	2170
0.46000	*	.	.	.	16	2169
0.46000	*	.	.	.	16	2168
0.46000	*	.	.	.	16	2167
0.46000	*	.	.	.	16	2166
0.46000	*	.	.	.	16	2165

phregparms_mianalyze_mi_4099

The LIFETEST Procedure

Stratum 3: Statin intensity at study enrollment = Medium-intensity statin dose

Product-Limit Survival Estimates						
Prim_Outc_Time		Survival	Failure	Survival Standard Error	Number Failed	Number Left
0.46000	*	.	.	.	16	2164
0.46000	*	.	.	.	16	2163
0.46000	*	.	.	.	16	2162
0.46000	*	.	.	.	16	2161
0.46000	*	.	.	.	16	2160
0.46000	*	.	.	.	16	2159
0.46000	*	.	.	.	16	2158
0.46000	*	.	.	.	16	2157
0.46000	*	.	.	.	16	2156
0.46000	*	.	.	.	16	2155
0.46000	*	.	.	.	16	2154
0.46000	*	.	.	.	16	2153
0.46000	*	.	.	.	16	2152
0.46000	*	.	.	.	16	2151
0.46000	*	.	.	.	16	2150
0.46000	*	.	.	.	16	2149
0.46000	*	.	.	.	16	2148
0.46000	*	.	.	.	16	2147
0.46000	*	.	.	.	16	2146
0.46000	*	.	.	.	16	2145
0.46000	*	.	.	.	16	2144
0.46000	*	.	.	.	16	2143
0.46000	*	.	.	.	16	2142
0.46000	*	.	.	.	16	2141
0.46000	*	.	.	.	16	2140
0.46000	*	.	.	.	16	2139
0.46000	*	.	.	.	16	2138
0.46000	*	.	.	.	16	2137
0.46000	*	.	.	.	16	2136

phregparms_mianalyze_mi_4099

The LIFETEST Procedure

Stratum 3: Statin intensity at study enrollment = Medium-intensity statin dose

Product-Limit Survival Estimates						
Prim_Outc_Time		Survival	Failure	Survival Standard Error	Number Failed	Number Left
0.46000	*	.	.	.	16	2135
0.46000	*	.	.	.	16	2134
0.46000	*	.	.	.	16	2133
0.46000	*	.	.	.	16	2132
0.46000	*	.	.	.	16	2131
0.46000	*	.	.	.	16	2130
0.46000	*	.	.	.	16	2129
0.46000	*	.	.	.	16	2128
0.46000	*	.	.	.	16	2127
0.46000	*	.	.	.	16	2126
0.46000	*	.	.	.	16	2125
0.46000	*	.	.	.	16	2124
0.46000	*	.	.	.	16	2123
0.46000	*	.	.	.	16	2122
0.46000	*	.	.	.	16	2121
0.46000	*	.	.	.	16	2120
0.46000	*	.	.	.	16	2119
0.46000	*	.	.	.	16	2118
0.46000	*	.	.	.	16	2117
0.46000	*	.	.	.	16	2116
0.46000	*	.	.	.	16	2115
0.46000	*	.	.	.	16	2114
0.46300	*	.	.	.	16	2113
0.46300	*	.	.	.	16	2112
0.46300	*	.	.	.	16	2111
0.46500	*	.	.	.	16	2110
0.46500	*	.	.	.	16	2109
0.46800	*	.	.	.	16	2108
0.47100		0.9926	0.00741	0.00179	17	2107

phregparms_mianalyze_mi_4099

The LIFETEST Procedure

Stratum 3: Statin intensity at study enrollment = Medium-intensity statin dose

Product-Limit Survival Estimates						
Prim_Outc_Time		Survival	Failure	Survival Standard Error	Number Failed	Number Left
0.47100	*	.	.	.	17	2106
0.47400	*	.	.	.	17	2105
0.47600		0.9921	0.00788	0.00185	18	2104
0.47600	*	.	.	.	18	2103
0.47900	*	.	.	.	18	2102
0.47900	*	.	.	.	18	2101
0.47900	*	.	.	.	18	2100
0.47900	*	.	.	.	18	2099
0.47900	*	.	.	.	18	2098
0.47900	*	.	.	.	18	2097
0.47900	*	.	.	.	18	2096
0.47900	*	.	.	.	18	2095
0.48200	*	.	.	.	18	2094
0.48200	*	.	.	.	18	2093
0.49000	*	.	.	.	18	2092
0.49600		0.9916	0.00836	0.00191	19	2091
0.49800	*	.	.	.	19	2090
0.49800	*	.	.	.	19	2089
0.49800	*	.	.	.	19	2088
0.49800	*	.	.	.	19	2087
0.49800	*	.	.	.	19	2086
0.50100	*	.	.	.	19	2085
0.50700	*	.	.	.	19	2084
0.50900	*	.	.	.	19	2083
0.50900	*	.	.	.	19	2082
0.51500		0.9912	0.00883	0.00197	20	2081
0.51500	*	.	.	.	20	2080
0.51700	*	.	.	.	20	2079
0.51700	*	.	.	.	20	2078

phregparms_mianalyze_mi_4099

The LIFETEST Procedure

Stratum 3: Statin intensity at study enrollment = Medium-intensity statin dose

Product-Limit Survival Estimates						
Prim_Outc_Time		Survival	Failure	Survival Standard Error	Number Failed	Number Left
0.51700	*	.	.	.	20	2077
0.51700	*	.	.	.	20	2076
0.51700	*	.	.	.	20	2075
0.51700	*	.	.	.	20	2074
0.52000	*	.	.	.	20	2073
0.52300	*	.	.	.	20	2072
0.52300	*	.	.	.	20	2071
0.52800	*	.	.	.	20	2070
0.53100		0.9907	0.00931	0.00202	21	2069
0.53700		0.9902	0.00979	0.00208	22	2068
0.53700	*	.	.	.	22	2067
0.53700	*	.	.	.	22	2066
0.53700	*	.	.	.	22	2065
0.53700	*	.	.	.	22	2064
0.54200	*	.	.	.	22	2063
0.54200	*	.	.	.	22	2062
0.55600	*	.	.	.	22	2061
0.55900	*	.	.	.	22	2060
0.55900	*	.	.	.	22	2059
0.56100	*	.	.	.	22	2058
0.56100	*	.	.	.	22	2057
0.57200	*	.	.	.	22	2056
0.57500	*	.	.	.	22	2055
0.57500	*	.	.	.	22	2054
0.57500	*	.	.	.	22	2053
0.57800	*	.	.	.	22	2052
0.58000	*	.	.	.	22	2051
0.58900	*	.	.	.	22	2050
0.58900	*	.	.	.	22	2049

phregparms_mianalyze_mi_4099

The LIFETEST Procedure

Stratum 3: Statin intensity at study enrollment = Medium-intensity statin dose

Product-Limit Survival Estimates						
Prim_Outc_Time		Survival	Failure	Survival Standard Error	Number Failed	Number Left
0.59400	*	.	.	.	22	2048
0.59400	*	.	.	.	22	2047
0.60200		0.9897	0.0103	0.00213	23	2046
0.60500	*	.	.	.	23	2045
0.60500	*	.	.	.	23	2044
0.60500	*	.	.	.	23	2043
0.60800	*	.	.	.	23	2042
0.61100	*	.	.	.	23	2041
0.61300	*	.	.	.	23	2040
0.61300	*	.	.	.	23	2039
0.61300	*	.	.	.	23	2038
0.61300	*	.	.	.	23	2037
0.61300	*	.	.	.	23	2036
0.61300	*	.	.	.	23	2035
0.61300	*	.	.	.	23	2034
0.61900	*	.	.	.	23	2033
0.62100		0.9892	0.0108	0.00219	24	2032
0.62100	*	.	.	.	24	2031
0.63200	*	.	.	.	24	2030
0.63200	*	.	.	.	24	2029
0.63800		0.9888	0.0112	0.00224	25	2028
0.63800	*	.	.	.	25	2027
0.64900		0.9883	0.0117	0.00229	26	2026
0.64900	*	.	.	.	26	2025
0.65200		0.9878	0.0122	0.00234	27	2024
0.65200	*	.	.	.	27	2023
0.65200	*	.	.	.	27	2022
0.65400		0.9873	0.0127	0.00239	28	2021
0.66300		0.9868	0.0132	0.00244	29	2020

phregparms_mianalyze_mi_4099

The LIFETEST Procedure

Stratum 3: Statin intensity at study enrollment = Medium-intensity statin dose

Product-Limit Survival Estimates						
Prim_Outc_Time		Survival	Failure	Survival Standard Error	Number Failed	Number Left
0.66300	*	.	.	.	29	2019
0.66800	*	.	.	.	29	2018
0.67100	*	.	.	.	29	2017
0.67100	*	.	.	.	29	2016
0.67100	*	.	.	.	29	2015
0.67100	*	.	.	.	29	2014
0.67100	*	.	.	.	29	2013
0.67100	*	.	.	.	29	2012
0.67100	*	.	.	.	29	2011
0.67100	*	.	.	.	29	2010
0.67100	*	.	.	.	29	2009
0.67100	*	.	.	.	29	2008
0.67100	*	.	.	.	29	2007
0.67100	*	.	.	.	29	2006
0.67100	*	.	.	.	29	2005
0.67100	*	.	.	.	29	2004
0.67400	*	.	.	.	29	2003
0.67900	*	.	.	.	29	2002
0.68200	*	.	.	.	29	2001
0.68700	*	.	.	.	29	2000
0.69000	*	.	.	.	29	1999
0.69000	*	.	.	.	29	1998
0.69000	*	.	.	.	29	1997
0.69000	*	.	.	.	29	1996
0.69000	*	.	.	.	29	1995
0.69000	*	.	.	.	29	1994
0.69000	*	.	.	.	29	1993
0.69000	*	.	.	.	29	1992
0.69000	*	.	.	.	29	1991

phregparms_mianalyze_mi_4099

The LIFETEST Procedure

Stratum 3: Statin intensity at study enrollment = Medium-intensity statin dose

Product-Limit Survival Estimates						
Prim_Outc_Time		Survival	Failure	Survival Standard Error	Number Failed	Number Left
0.69000	*	.	.	.	29	1990
0.69000	*	.	.	.	29	1989
0.69000	*	.	.	.	29	1988
0.69000	*	.	.	.	29	1987
0.69000	*	.	.	.	29	1986
0.69000	*	.	.	.	29	1985
0.69000	*	.	.	.	29	1984
0.69000	*	.	.	.	29	1983
0.69000	*	.	.	.	29	1982
0.69000	*	.	.	.	29	1981
0.69000	*	.	.	.	29	1980
0.69000	*	.	.	.	29	1979
0.69000	*	.	.	.	29	1978
0.69000	*	.	.	.	29	1977
0.69000	*	.	.	.	29	1976
0.69000	*	.	.	.	29	1975
0.69000	*	.	.	.	29	1974
0.69000	*	.	.	.	29	1973
0.69000	*	.	.	.	29	1972
0.69000	*	.	.	.	29	1971
0.69000	*	.	.	.	29	1970
0.69000	*	.	.	.	29	1969
0.69000	*	.	.	.	29	1968
0.69000	*	.	.	.	29	1967
0.69000	*	.	.	.	29	1966
0.69000	*	.	.	.	29	1965
0.69000	*	.	.	.	29	1964
0.69000	*	.	.	.	29	1963
0.69000	*	.	.	.	29	1962

phregparms_mianalyze_mi_4099

The LIFETEST Procedure

Stratum 3: Statin intensity at study enrollment = Medium-intensity statin dose

Product-Limit Survival Estimates						
Prim_Outc_Time		Survival	Failure	Survival Standard Error	Number Failed	Number Left
0.69000	*	.	.	.	29	1961
0.69000	*	.	.	.	29	1960
0.69000	*	.	.	.	29	1959
0.69000	*	.	.	.	29	1958
0.69000	*	.	.	.	29	1957
0.69000	*	.	.	.	29	1956
0.69000	*	.	.	.	29	1955
0.69000	*	.	.	.	29	1954
0.69000	*	.	.	.	29	1953
0.69000	*	.	.	.	29	1952
0.69000	*	.	.	.	29	1951
0.69000	*	.	.	.	29	1950
0.69000	*	.	.	.	29	1949
0.69000	*	.	.	.	29	1948
0.69000	*	.	.	.	29	1947
0.69000	*	.	.	.	29	1946
0.69000	*	.	.	.	29	1945
0.69000	*	.	.	.	29	1944
0.69000	*	.	.	.	29	1943
0.69000	*	.	.	.	29	1942
0.69000	*	.	.	.	29	1941
0.69000	*	.	.	.	29	1940
0.69300	*	.	.	.	29	1939
0.69500	*	.	.	.	29	1938
0.70100		0.9863	0.0137	0.00249	30	1937
0.70400	*	.	.	.	30	1936
0.70600	*	.	.	.	30	1935
0.70600	*	.	.	.	30	1934
0.70600	*	.	.	.	30	1933

phregparms_mianalyze_mi_4099

The LIFETEST Procedure

Stratum 3: Statin intensity at study enrollment = Medium-intensity statin dose

Product-Limit Survival Estimates						
Prim_Outc_Time		Survival	Failure	Survival Standard Error	Number Failed	Number Left
0.70900	*	.	.	.	30	1932
0.70900	*	.	.	.	30	1931
0.70900	*	.	.	.	30	1930
0.70900	*	.	.	.	30	1929
0.70900	*	.	.	.	30	1928
0.70900	*	.	.	.	30	1927
0.70900	*	.	.	.	30	1926
0.70900	*	.	.	.	30	1925
0.70900	*	.	.	.	30	1924
0.70900	*	.	.	.	30	1923
0.70900	*	.	.	.	30	1922
0.70900	*	.	.	.	30	1921
0.70900	*	.	.	.	30	1920
0.70900	*	.	.	.	30	1919
0.70900	*	.	.	.	30	1918
0.70900	*	.	.	.	30	1917
0.70900	*	.	.	.	30	1916
0.70900	*	.	.	.	30	1915
0.70900	*	.	.	.	30	1914
0.72800	*	.	.	.	30	1913
0.72800	*	.	.	.	30	1912
0.72800	*	.	.	.	30	1911
0.72800	*	.	.	.	30	1910
0.74200		0.9858	0.0142	0.00254	31	1909
0.74200	*	.	.	.	31	1908
0.74200	*	.	.	.	31	1907
0.74200	*	.	.	.	31	1906
0.74700	*	.	.	.	31	1905
0.74700	*	.	.	.	31	1904

phregparms_mianalyze_mi_4099

The LIFETEST Procedure

Stratum 3: Statin intensity at study enrollment = Medium-intensity statin dose

Product-Limit Survival Estimates						
Prim_Outc_Time		Survival	Failure	Survival Standard Error	Number Failed	Number Left
0.74700	*	.	.	.	31	1903
0.74700	*	.	.	.	31	1902
0.74700	*	.	.	.	31	1901
0.74700	*	.	.	.	31	1900
0.74700	*	.	.	.	31	1899
0.76700	*	.	.	.	31	1898
0.76700	*	.	.	.	31	1897
0.76700	*	.	.	.	31	1896
0.76700	*	.	.	.	31	1895
0.76700	*	.	.	.	31	1894
0.76900	*	.	.	.	31	1893
0.76900	*	.	.	.	31	1892
0.77800	*	.	.	.	31	1891
0.78000	*	.	.	.	31	1890
0.78300	*	.	.	.	31	1889
0.78600	*	.	.	.	31	1888
0.79100	*	.	.	.	31	1887
0.80200	*	.	.	.	31	1886
0.82100	*	.	.	.	31	1885
0.82100	*	.	.	.	31	1884
0.82100	*	.	.	.	31	1883
0.82400		0.9852	0.0148	0.00259	32	1882
0.82400	*	.	.	.	32	1881
0.82400	*	.	.	.	32	1880
0.82700	*	.	.	.	32	1879
0.83000		0.9847	0.0153	0.00265	33	1878
0.83500	*	.	.	.	33	1877
0.83800	*	.	.	.	33	1876
0.84300	*	.	.	.	33	1875

phregparms_mianalyze_mi_4099

The LIFETEST Procedure

Stratum 3: Statin intensity at study enrollment = Medium-intensity statin dose

Product-Limit Survival Estimates						
Prim_Outc_Time		Survival	Failure	Survival Standard Error	Number Failed	Number Left
0.84300	*	.	.	.	33	1874
0.84300	*	.	.	.	33	1873
0.84300	*	.	.	.	33	1872
0.84300	*	.	.	.	33	1871
0.84300	*	.	.	.	33	1870
0.84900	*	.	.	.	33	1869
0.85100	*	.	.	.	33	1868
0.85400	*	.	.	.	33	1867
0.85700	*	.	.	.	33	1866
0.85700	*	.	.	.	33	1865
0.86200	*	.	.	.	33	1864
0.86200	*	.	.	.	33	1863
0.86200	*	.	.	.	33	1862
0.86500	*	.	.	.	33	1861
0.87300	*	.	.	.	33	1860
0.87300	*	.	.	.	33	1859
0.87600	*	.	.	.	33	1858
0.87900	*	.	.	.	33	1857
0.87900	*	.	.	.	33	1856
0.88200	*	.	.	.	33	1855
0.88200	*	.	.	.	33	1854
0.88200	*	.	.	.	33	1853
0.88200	*	.	.	.	33	1852
0.88200	*	.	.	.	33	1851
0.88400	*	.	.	.	33	1850
0.89000		0.9842	0.0158	0.00270	34	1849
0.89500	*	.	.	.	34	1848
0.89800	*	.	.	.	34	1847
0.90100	*	.	.	.	34	1846

phregparms_mianalyze_mi_4099

The LIFETEST Procedure

Stratum 3: Statin intensity at study enrollment = Medium-intensity statin dose

Product-Limit Survival Estimates						
Prim_Outc_Time		Survival	Failure	Survival Standard Error	Number Failed	Number Left
0.90100	*	.	.	.	34	1845
0.90100	*	.	.	.	34	1844
0.90100	*	.	.	.	34	1843
0.90100	*	.	.	.	34	1842
0.90100	*	.	.	.	34	1841
0.90100	*	.	.	.	34	1840
0.90100	*	.	.	.	34	1839
0.90100	*	.	.	.	34	1838
0.90100	*	.	.	.	34	1837
0.90100	*	.	.	.	34	1836
0.90100	*	.	.	.	34	1835
0.90100	*	.	.	.	34	1834
0.90100	*	.	.	.	34	1833
0.90300	*	.	.	.	34	1832
0.90300	*	.	.	.	34	1831
0.90300	*	.	.	.	34	1830
0.90300	*	.	.	.	34	1829
0.90600	*	.	.	.	34	1828
0.91200	*	.	.	.	34	1827
0.91200	*	.	.	.	34	1826
0.91400	*	.	.	.	34	1825
0.91400	*	.	.	.	34	1824
0.91400	*	.	.	.	34	1823
0.91400	*	.	.	.	34	1822
0.91400	*	.	.	.	34	1821
0.91700	*	.	.	.	34	1820
0.91700	*	.	.	.	34	1819
0.91700	*	.	.	.	34	1818
0.92000	*	.	.	.	34	1817

phregparms_mianalyze_mi_4099

The LIFETEST Procedure

Stratum 3: Statin intensity at study enrollment = Medium-intensity statin dose

Product-Limit Survival Estimates						
Prim_Outc_Time		Survival	Failure	Survival Standard Error	Number Failed	Number Left
0.92000	*	.	.	.	34	1816
0.92000	*	.	.	.	34	1815
0.92000	*	.	.	.	34	1814
0.92000	*	.	.	.	34	1813
0.92000	*	.	.	.	34	1812
0.92000	*	.	.	.	34	1811
0.92000	*	.	.	.	34	1810
0.92000	*	.	.	.	34	1809
0.92000	*	.	.	.	34	1808
0.92000	*	.	.	.	34	1807
0.92000	*	.	.	.	34	1806
0.92000	*	.	.	.	34	1805
0.92000	*	.	.	.	34	1804
0.92000	*	.	.	.	34	1803
0.92000	*	.	.	.	34	1802
0.92000	*	.	.	.	34	1801
0.92000	*	.	.	.	34	1800
0.92000	*	.	.	.	34	1799
0.92000	*	.	.	.	34	1798
0.92000	*	.	.	.	34	1797
0.92000	*	.	.	.	34	1796
0.92000	*	.	.	.	34	1795
0.92000	*	.	.	.	34	1794
0.92000	*	.	.	.	34	1793
0.92000	*	.	.	.	34	1792
0.92000	*	.	.	.	34	1791
0.92000	*	.	.	.	34	1790
0.92000	*	.	.	.	34	1789
0.92000	*	.	.	.	34	1788

phregparms_mianalyze_mi_4099

The LIFETEST Procedure

Stratum 3: Statin intensity at study enrollment = Medium-intensity statin dose

Product-Limit Survival Estimates						
Prim_Outc_Time		Survival	Failure	Survival Standard Error	Number Failed	Number Left
0.92000	*	.	.	.	34	1787
0.92000	*	.	.	.	34	1786
0.92000	*	.	.	.	34	1785
0.92000	*	.	.	.	34	1784
0.92000	*	.	.	.	34	1783
0.92000	*	.	.	.	34	1782
0.92000	*	.	.	.	34	1781
0.92000	*	.	.	.	34	1780
0.92000	*	.	.	.	34	1779
0.92000	*	.	.	.	34	1778
0.92000	*	.	.	.	34	1777
0.92000	*	.	.	.	34	1776
0.92000	*	.	.	.	34	1775
0.92000	*	.	.	.	34	1774
0.92000	*	.	.	.	34	1773
0.92000	*	.	.	.	34	1772
0.92000	*	.	.	.	34	1771
0.92000	*	.	.	.	34	1770
0.92000	*	.	.	.	34	1769
0.92000	*	.	.	.	34	1768
0.92000	*	.	.	.	34	1767
0.92000	*	.	.	.	34	1766
0.92000	*	.	.	.	34	1765
0.92000	*	.	.	.	34	1764
0.92000	*	.	.	.	34	1763
0.92000	*	.	.	.	34	1762
0.92000	*	.	.	.	34	1761
0.92000	*	.	.	.	34	1760
0.92000	*	.	.	.	34	1759

phregparms_mianalyze_mi_4099

The LIFETEST Procedure

Stratum 3: Statin intensity at study enrollment = Medium-intensity statin dose

Product-Limit Survival Estimates						
Prim_Outc_Time		Survival	Failure	Survival Standard Error	Number Failed	Number Left
0.92000	*	.	.	.	34	1758
0.92000	*	.	.	.	34	1757
0.92000	*	.	.	.	34	1756
0.92000	*	.	.	.	34	1755
0.92000	*	.	.	.	34	1754
0.92000	*	.	.	.	34	1753
0.92000	*	.	.	.	34	1752
0.92000	*	.	.	.	34	1751
0.92000	*	.	.	.	34	1750
0.92000	*	.	.	.	34	1749
0.92000	*	.	.	.	34	1748
0.92000	*	.	.	.	34	1747
0.92000	*	.	.	.	34	1746
0.92000	*	.	.	.	34	1745
0.92000	*	.	.	.	34	1744
0.92000	*	.	.	.	34	1743
0.92000	*	.	.	.	34	1742
0.92000	*	.	.	.	34	1741
0.92000	*	.	.	.	34	1740
0.92000	*	.	.	.	34	1739
0.92000	*	.	.	.	34	1738
0.92000	*	.	.	.	34	1737
0.92000	*	.	.	.	34	1736
0.92000	*	.	.	.	34	1735
0.92000	*	.	.	.	34	1734
0.92000	*	.	.	.	34	1733
0.92000	*	.	.	.	34	1732
0.92000	*	.	.	.	34	1731
0.92000	*	.	.	.	34	1730

phregparms_mianalyze_mi_4099

The LIFETEST Procedure

Stratum 3: Statin intensity at study enrollment = Medium-intensity statin dose

Product-Limit Survival Estimates						
Prim_Outc_Time		Survival	Failure	Survival Standard Error	Number Failed	Number Left
0.92000	*	.	.	.	34	1729
0.92000	*	.	.	.	34	1728
0.92000	*	.	.	.	34	1727
0.92000	*	.	.	.	34	1726
0.92000	*	.	.	.	34	1725
0.92000	*	.	.	.	34	1724
0.92000	*	.	.	.	34	1723
0.92000	*	.	.	.	34	1722
0.92000	*	.	.	.	34	1721
0.92000	*	.	.	.	34	1720
0.92000	*	.	.	.	34	1719
0.92000	*	.	.	.	34	1718
0.92000	*	.	.	.	34	1717
0.92000	*	.	.	.	34	1716
0.92000	*	.	.	.	34	1715
0.92000	*	.	.	.	34	1714
0.92000	*	.	.	.	34	1713
0.92000	*	.	.	.	34	1712
0.92000	*	.	.	.	34	1711
0.92000	*	.	.	.	34	1710
0.92000	*	.	.	.	34	1709
0.92000	*	.	.	.	34	1708
0.92000	*	.	.	.	34	1707
0.92000	*	.	.	.	34	1706
0.92000	*	.	.	.	34	1705
0.92000	*	.	.	.	34	1704
0.92000	*	.	.	.	34	1703
0.92000	*	.	.	.	34	1702
0.92000	*	.	.	.	34	1701

phregparms_mianalyze_mi_4099

The LIFETEST Procedure

Stratum 3: Statin intensity at study enrollment = Medium-intensity statin dose

Product-Limit Survival Estimates						
Prim_Outc_Time		Survival	Failure	Survival Standard Error	Number Failed	Number Left
0.92000	*	.	.	.	34	1700
0.92000	*	.	.	.	34	1699
0.92000	*	.	.	.	34	1698
0.92000	*	.	.	.	34	1697
0.92000	*	.	.	.	34	1696
0.92000	*	.	.	.	34	1695
0.92000	*	.	.	.	34	1694
0.92000	*	.	.	.	34	1693
0.92000	*	.	.	.	34	1692
0.92000	*	.	.	.	34	1691
0.92000	*	.	.	.	34	1690
0.92000	*	.	.	.	34	1689
0.92000	*	.	.	.	34	1688
0.92000	*	.	.	.	34	1687
0.92000	*	.	.	.	34	1686
0.92000	*	.	.	.	34	1685
0.92000	*	.	.	.	34	1684
0.92000	*	.	.	.	34	1683
0.92000	*	.	.	.	34	1682
0.92000	*	.	.	.	34	1681
0.92000	*	.	.	.	34	1680
0.92000	*	.	.	.	34	1679
0.92000	*	.	.	.	34	1678
0.92000	*	.	.	.	34	1677
0.92000	*	.	.	.	34	1676
0.92000	*	.	.	.	34	1675
0.92000	*	.	.	.	34	1674
0.92000	*	.	.	.	34	1673
0.92000	*	.	.	.	34	1672

phregparms_mianalyze_mi_4099

The LIFETEST Procedure

Stratum 3: Statin intensity at study enrollment = Medium-intensity statin dose

Product-Limit Survival Estimates						
Prim_Outc_Time		Survival	Failure	Survival Standard Error	Number Failed	Number Left
0.92000	*	.	.	.	34	1671
0.92000	*	.	.	.	34	1670
0.92000	*	.	.	.	34	1669
0.92000	*	.	.	.	34	1668
0.92000	*	.	.	.	34	1667
0.92000	*	.	.	.	34	1666
0.92000	*	.	.	.	34	1665
0.92000	*	.	.	.	34	1664
0.92000	*	.	.	.	34	1663
0.92000	*	.	.	.	34	1662
0.92000	*	.	.	.	34	1661
0.92000	*	.	.	.	34	1660
0.92000	*	.	.	.	34	1659
0.92000	*	.	.	.	34	1658
0.92000	*	.	.	.	34	1657
0.92000	*	.	.	.	34	1656
0.92000	*	.	.	.	34	1655
0.92000	*	.	.	.	34	1654
0.92000	*	.	.	.	34	1653
0.92000	*	.	.	.	34	1652
0.92000	*	.	.	.	34	1651
0.92000	*	.	.	.	34	1650
0.92000	*	.	.	.	34	1649
0.92000	*	.	.	.	34	1648
0.92000	*	.	.	.	34	1647
0.92000	*	.	.	.	34	1646
0.92000	*	.	.	.	34	1645
0.92000	*	.	.	.	34	1644
0.92000	*	.	.	.	34	1643

phregparms_mianalyze_mi_4099

The LIFETEST Procedure

Stratum 3: Statin intensity at study enrollment = Medium-intensity statin dose

Product-Limit Survival Estimates						
Prim_Outc_Time		Survival	Failure	Survival Standard Error	Number Failed	Number Left
0.92000	*	.	.	.	34	1642
0.92000	*	.	.	.	34	1641
0.92000	*	.	.	.	34	1640
0.92000	*	.	.	.	34	1639
0.92000	*	.	.	.	34	1638
0.92000	*	.	.	.	34	1637
0.92000	*	.	.	.	34	1636
0.92000	*	.	.	.	34	1635
0.92000	*	.	.	.	34	1634
0.92000	*	.	.	.	34	1633
0.92000	*	.	.	.	34	1632
0.92000	*	.	.	.	34	1631
0.92000	*	.	.	.	34	1630
0.92000	*	.	.	.	34	1629
0.92000	*	.	.	.	34	1628
0.92000	*	.	.	.	34	1627
0.92000	*	.	.	.	34	1626
0.92000	*	.	.	.	34	1625
0.92000	*	.	.	.	34	1624
0.92000	*	.	.	.	34	1623
0.92000	*	.	.	.	34	1622
0.92000	*	.	.	.	34	1621
0.92000	*	.	.	.	34	1620
0.92000	*	.	.	.	34	1619
0.92000	*	.	.	.	34	1618
0.92000	*	.	.	.	34	1617
0.92000	*	.	.	.	34	1616
0.92000	*	.	.	.	34	1615
0.92000	*	.	.	.	34	1614

phregparms_mianalyze_mi_4099

The LIFETEST Procedure

Stratum 3: Statin intensity at study enrollment = Medium-intensity statin dose

Product-Limit Survival Estimates						
Prim_Outc_Time		Survival	Failure	Survival Standard Error	Number Failed	Number Left
0.92000	*	.	.	.	34	1613
0.92300	*	.	.	.	34	1612
0.92300	*	.	.	.	34	1611
0.92300	*	.	.	.	34	1610
0.92500	*	.	.	.	34	1609
0.92500	*	.	.	.	34	1608
0.93100	*	.	.	.	34	1607
0.93100	*	.	.	.	34	1606
0.93400	*	.	.	.	34	1605
0.93600	*	.	.	.	34	1604
0.93600	*	.	.	.	34	1603
0.93600	*	.	.	.	34	1602
0.93900	*	.	.	.	34	1601
0.93900	*	.	.	.	34	1600
0.93900	*	.	.	.	34	1599
0.93900	*	.	.	.	34	1598
0.93900	*	.	.	.	34	1597
0.93900	*	.	.	.	34	1596
0.93900	*	.	.	.	34	1595
0.93900	*	.	.	.	34	1594
0.93900	*	.	.	.	34	1593
0.93900	*	.	.	.	34	1592
0.93900	*	.	.	.	34	1591
0.93900	*	.	.	.	34	1590
0.93900	*	.	.	.	34	1589
0.93900	*	.	.	.	34	1588
0.93900	*	.	.	.	34	1587
0.93900	*	.	.	.	34	1586
0.93900	*	.	.	.	34	1585

phregparms_mianalyze_mi_4099

The LIFETEST Procedure

Stratum 3: Statin intensity at study enrollment = Medium-intensity statin dose

Product-Limit Survival Estimates						
Prim_Outc_Time		Survival	Failure	Survival Standard Error	Number Failed	Number Left
0.93900	*	.	.	.	34	1584
0.93900	*	.	.	.	34	1583
0.93900	*	.	.	.	34	1582
0.93900	*	.	.	.	34	1581
0.93900	*	.	.	.	34	1580
0.93900	*	.	.	.	34	1579
0.93900	*	.	.	.	34	1578
0.93900	*	.	.	.	34	1577
0.93900	*	.	.	.	34	1576
0.93900	*	.	.	.	34	1575
0.93900	*	.	.	.	34	1574
0.93900	*	.	.	.	34	1573
0.94200	*	.	.	.	34	1572
0.94200	*	.	.	.	34	1571
0.94200	*	.	.	.	34	1570
0.94500	*	.	.	.	34	1569
0.94700	*	.	.	.	34	1568
0.95000	*	.	.	.	34	1567
0.95000	*	.	.	.	34	1566
0.95000	*	.	.	.	34	1565
0.95000	*	.	.	.	34	1564
0.95300	*	.	.	.	34	1563
0.95300	*	.	.	.	34	1562
0.95600	*	.	.	.	34	1561
0.95600	*	.	.	.	34	1560
0.95600	*	.	.	.	34	1559
0.95600	*	.	.	.	34	1558
0.95800	*	.	.	.	34	1557
0.95800	*	.	.	.	34	1556

phregparms_mianalyze_mi_4099

The LIFETEST Procedure

Stratum 3: Statin intensity at study enrollment = Medium-intensity statin dose

Product-Limit Survival Estimates						
Prim_Outc_Time		Survival	Failure	Survival Standard Error	Number Failed	Number Left
0.95800	*	.	.	.	34	1555
0.95800	*	.	.	.	34	1554
0.95800	*	.	.	.	34	1553
0.95800	*	.	.	.	34	1552
0.95800	*	.	.	.	34	1551
0.95800	*	.	.	.	34	1550
0.95800	*	.	.	.	34	1549
0.95800	*	.	.	.	34	1548
0.95800	*	.	.	.	34	1547
0.95800	*	.	.	.	34	1546
0.95800	*	.	.	.	34	1545
0.95800	*	.	.	.	34	1544
0.95800	*	.	.	.	34	1543
0.96400	*	.	.	.	34	1542
0.96400	*	.	.	.	34	1541
0.96400	*	.	.	.	34	1540
0.96600	*	.	.	.	34	1539
0.97200		0.9836	0.0164	0.00277	35	1538
0.97200	*	.	.	.	35	1537
0.97200	*	.	.	.	35	1536
0.97500	*	.	.	.	35	1535
0.97700	*	.	.	.	35	1534
0.97700	*	.	.	.	35	1533
0.97700	*	.	.	.	35	1532
0.97700	*	.	.	.	35	1531
0.97700	*	.	.	.	35	1530
0.97700	*	.	.	.	35	1529
0.97700	*	.	.	.	35	1528
0.97700	*	.	.	.	35	1527

phregparms_mianalyze_mi_4099

The LIFETEST Procedure

Stratum 3: Statin intensity at study enrollment = Medium-intensity statin dose

Product-Limit Survival Estimates						
Prim_Outc_Time		Survival	Failure	Survival Standard Error	Number Failed	Number Left
0.97700	*	.	.	.	35	1526
0.97700	*	.	.	.	35	1525
0.97700	*	.	.	.	35	1524
0.97700	*	.	.	.	35	1523
0.97700	*	.	.	.	35	1522
0.98000	*	.	.	.	35	1521
0.98000	*	.	.	.	35	1520
0.98600	*	.	.	.	35	1519
0.98600	*	.	.	.	35	1518
0.99100		0.9829	0.0171	0.00284	36	1517
0.99400	*	.	.	.	36	1516
0.99700	*	.	.	.	36	1515
0.99700	*	.	.	.	36	1514
0.99700	*	.	.	.	36	1513
0.99700	*	.	.	.	36	1512
0.99700	*	.	.	.	36	1511
0.99700	*	.	.	.	36	1510
0.99700	*	.	.	.	36	1509
0.99700	*	.	.	.	36	1508
0.99700	*	.	.	.	36	1507
0.99700	*	.	.	.	36	1506
0.99700	*	.	.	.	36	1505
0.99700	*	.	.	.	36	1504
0.99700	*	.	.	.	36	1503
0.99700	*	.	.	.	36	1502
0.99700	*	.	.	.	36	1501
0.99700	*	.	.	.	36	1500
0.99700	*	.	.	.	36	1499
0.99700	*	.	.	.	36	1498

phregparms_mianalyze_mi_4099

The LIFETEST Procedure

Stratum 3: Statin intensity at study enrollment = Medium-intensity statin dose

Product-Limit Survival Estimates						
Prim_Outc_Time		Survival	Failure	Survival Standard Error	Number Failed	Number Left
0.99700	*	.	.	.	36	1497
0.99700	*	.	.	.	36	1496
0.99700	*	.	.	.	36	1495
0.99700	*	.	.	.	36	1494
0.99700	*	.	.	.	36	1493
0.99700	*	.	.	.	36	1492
0.99900	*	.	.	.	36	1491
1.00200	*	.	.	.	36	1490
1.00200	*	.	.	.	36	1489
1.00200	*	.	.	.	36	1488
1.00200	*	.	.	.	36	1487
1.00200	*	.	.	.	36	1486
1.00200	*	.	.	.	36	1485
1.00200	*	.	.	.	36	1484
1.00200	*	.	.	.	36	1483
1.00200	*	.	.	.	36	1482
1.00200	*	.	.	.	36	1481
1.00200	*	.	.	.	36	1480
1.01300		0.9822	0.0178	0.00292	37	1479
1.01300	*	.	.	.	37	1478
1.01300	*	.	.	.	37	1477
1.01300	*	.	.	.	37	1476
1.01600	*	.	.	.	37	1475
1.01600	*	.	.	.	37	1474
1.01600	*	.	.	.	37	1473
1.01600	*	.	.	.	37	1472
1.01600	*	.	.	.	37	1471
1.01600	*	.	.	.	37	1470
1.01600	*	.	.	.	37	1469

phregparms_mianalyze_mi_4099

The LIFETEST Procedure

Stratum 3: Statin intensity at study enrollment = Medium-intensity statin dose

Product-Limit Survival Estimates						
Prim_Outc_Time		Survival	Failure	Survival Standard Error	Number Failed	Number Left
1.02100		0.9816	0.0184	0.00299	38	1468
1.02100	*	.	.	.	38	1467
1.02100	*	.	.	.	38	1466
1.02700		0.9809	0.0191	0.00306	39	1465
1.02700	*	.	.	.	39	1464
1.02700	*	.	.	.	39	1463
1.02900	*	.	.	.	39	1462
1.02900	*	.	.	.	39	1461
1.02900	*	.	.	.	39	1460
1.03200	*	.	.	.	39	1459
1.03200	*	.	.	.	39	1458
1.03500	*	.	.	.	39	1457
1.03500	*	.	.	.	39	1456
1.03500	*	.	.	.	39	1455
1.03500	*	.	.	.	39	1454
1.03500	*	.	.	.	39	1453
1.03500	*	.	.	.	39	1452
1.03500	*	.	.	.	39	1451
1.03800	*	.	.	.	39	1450
1.04600	*	.	.	.	39	1449
1.04900	*	.	.	.	39	1448
1.05100	*	.	.	.	39	1447
1.05400	*	.	.	.	39	1446
1.05400	*	.	.	.	39	1445
1.05400	*	.	.	.	39	1444
1.05400	*	.	.	.	39	1443
1.05400	*	.	.	.	39	1442
1.05400	*	.	.	.	39	1441
1.05400	*	.	.	.	39	1440

phregparms_mianalyze_mi_4099

The LIFETEST Procedure

Stratum 3: Statin intensity at study enrollment = Medium-intensity statin dose

Product-Limit Survival Estimates						
Prim_Outc_Time		Survival	Failure	Survival Standard Error	Number Failed	Number Left
1.05400	*	.	.	.	39	1439
1.06800	*	.	.	.	39	1438
1.07000	*	.	.	.	39	1437
1.07300	*	.	.	.	39	1436
1.07300	*	.	.	.	39	1435
1.07300	*	.	.	.	39	1434
1.07300	*	.	.	.	39	1433
1.07300	*	.	.	.	39	1432
1.07300	*	.	.	.	39	1431
1.07300	*	.	.	.	39	1430
1.07300	*	.	.	.	39	1429
1.07300	*	.	.	.	39	1428
1.07300	*	.	.	.	39	1427
1.07300	*	.	.	.	39	1426
1.07300	*	.	.	.	39	1425
1.07300	*	.	.	.	39	1424
1.07300	*	.	.	.	39	1423
1.07300	*	.	.	.	39	1422
1.07300	*	.	.	.	39	1421
1.07300	*	.	.	.	39	1420
1.07300	*	.	.	.	39	1419
1.07300	*	.	.	.	39	1418
1.07300	*	.	.	.	39	1417
1.07300	*	.	.	.	39	1416
1.07300	*	.	.	.	39	1415
1.07300	*	.	.	.	39	1414
1.07300	*	.	.	.	39	1413
1.07300	*	.	.	.	39	1412
1.07300	*	.	.	.	39	1411

phregparms_mianalyze_mi_4099

The LIFETEST Procedure

Stratum 3: Statin intensity at study enrollment = Medium-intensity statin dose

Product-Limit Survival Estimates						
Prim_Outc_Time		Survival	Failure	Survival Standard Error	Number Failed	Number Left
1.07300	*	.	.	.	39	1410
1.07300	*	.	.	.	39	1409
1.07300	*	.	.	.	39	1408
1.07900	*	.	.	.	39	1407
1.08100	*	.	.	.	39	1406
1.08700	*	.	.	.	39	1405
1.08700	*	.	.	.	39	1404
1.09000	*	.	.	.	39	1403
1.09000	*	.	.	.	39	1402
1.09200	*	.	.	.	39	1401
1.09200	*	.	.	.	39	1400
1.09200	*	.	.	.	39	1399
1.09200	*	.	.	.	39	1398
1.09200	*	.	.	.	39	1397
1.09200	*	.	.	.	39	1396
1.09200	*	.	.	.	39	1395
1.09800	*	.	.	.	39	1394
1.10100	*	.	.	.	39	1393
1.10600	*	.	.	.	39	1392
1.10600	*	.	.	.	39	1391
1.10900	*	.	.	.	39	1390
1.11200	*	.	.	.	39	1389
1.11200	*	.	.	.	39	1388
1.11200	*	.	.	.	39	1387
1.11200	*	.	.	.	39	1386
1.11200	*	.	.	.	39	1385
1.11400	*	.	.	.	39	1384
1.12300	*	.	.	.	39	1383
1.12300	*	.	.	.	39	1382

phregparms_mianalyze_mi_4099

The LIFETEST Procedure

Stratum 3: Statin intensity at study enrollment = Medium-intensity statin dose

Product-Limit Survival Estimates						
Prim_Outc_Time		Survival	Failure	Survival Standard Error	Number Failed	Number Left
1.12300	*	.	.	.	39	1381
1.12500	*	.	.	.	39	1380
1.12500	*	.	.	.	39	1379
1.12500	*	.	.	.	39	1378
1.13100	*	.	.	.	39	1377
1.13100	*	.	.	.	39	1376
1.13100	*	.	.	.	39	1375
1.13100	*	.	.	.	39	1374
1.13600	*	.	.	.	39	1373
1.14400	*	.	.	.	39	1372
1.14400	*	.	.	.	39	1371
1.14700		0.9802	0.0198	0.00314	40	1370
1.14700	*	.	.	.	40	1369
1.15000	*	.	.	.	40	1368
1.15000	*	.	.	.	40	1367
1.15000	*	.	.	.	40	1366
1.15000	*	.	.	.	40	1365
1.15000	*	.	.	.	40	1364
1.15000	*	.	.	.	40	1363
1.15000	*	.	.	.	40	1362
1.15000	*	.	.	.	40	1361
1.15000	*	.	.	.	40	1360
1.15000	*	.	.	.	40	1359
1.15000	*	.	.	.	40	1358
1.15000	*	.	.	.	40	1357
1.15000	*	.	.	.	40	1356
1.15000	*	.	.	.	40	1355
1.15000	*	.	.	.	40	1354
1.15000	*	.	.	.	40	1353

phregparms_mianalyze_mi_4099

The LIFETEST Procedure

Stratum 3: Statin intensity at study enrollment = Medium-intensity statin dose

Product-Limit Survival Estimates						
Prim_Outc_Time		Survival	Failure	Survival Standard Error	Number Failed	Number Left
1.15000	*	.	.	.	40	1352
1.15000	*	.	.	.	40	1351
1.15000	*	.	.	.	40	1350
1.15000	*	.	.	.	40	1349
1.15000	*	.	.	.	40	1348
1.15000	*	.	.	.	40	1347
1.15000	*	.	.	.	40	1346
1.15000	*	.	.	.	40	1345
1.15000	*	.	.	.	40	1344
1.15000	*	.	.	.	40	1343
1.15000	*	.	.	.	40	1342
1.15000	*	.	.	.	40	1341
1.15000	*	.	.	.	40	1340
1.15000	*	.	.	.	40	1339
1.15000	*	.	.	.	40	1338
1.15000	*	.	.	.	40	1337
1.15000	*	.	.	.	40	1336
1.15000	*	.	.	.	40	1335
1.15000	*	.	.	.	40	1334
1.15000	*	.	.	.	40	1333
1.15000	*	.	.	.	40	1332
1.15000	*	.	.	.	40	1331
1.15000	*	.	.	.	40	1330
1.15000	*	.	.	.	40	1329
1.15000	*	.	.	.	40	1328
1.15000	*	.	.	.	40	1327
1.15000	*	.	.	.	40	1326
1.15000	*	.	.	.	40	1325
1.15000	*	.	.	.	40	1324

phregparms_mianalyze_mi_4099

The LIFETEST Procedure

Stratum 3: Statin intensity at study enrollment = Medium-intensity statin dose

Product-Limit Survival Estimates						
Prim_Outc_Time		Survival	Failure	Survival Standard Error	Number Failed	Number Left
1.15000	*	.	.	.	40	1323
1.15000	*	.	.	.	40	1322
1.15000	*	.	.	.	40	1321
1.15000	*	.	.	.	40	1320
1.15000	*	.	.	.	40	1319
1.15000	*	.	.	.	40	1318
1.15000	*	.	.	.	40	1317
1.15000	*	.	.	.	40	1316
1.15000	*	.	.	.	40	1315
1.15000	*	.	.	.	40	1314
1.15000	*	.	.	.	40	1313
1.15000	*	.	.	.	40	1312
1.15000	*	.	.	.	40	1311
1.15000	*	.	.	.	40	1310
1.15000	*	.	.	.	40	1309
1.15000	*	.	.	.	40	1308
1.15000	*	.	.	.	40	1307
1.15000	*	.	.	.	40	1306
1.15000	*	.	.	.	40	1305
1.15000	*	.	.	.	40	1304
1.15300	*	.	.	.	40	1303
1.15300	*	.	.	.	40	1302
1.15300	*	.	.	.	40	1301
1.15300	*	.	.	.	40	1300
1.15300	*	.	.	.	40	1299
1.15500	*	.	.	.	40	1298
1.15500	*	.	.	.	40	1297
1.15800	*	.	.	.	40	1296
1.16400	*	.	.	.	40	1295

phregparms_mianalyze_mi_4099

The LIFETEST Procedure

Stratum 3: Statin intensity at study enrollment = Medium-intensity statin dose

Product-Limit Survival Estimates						
Prim_Outc_Time		Survival	Failure	Survival Standard Error	Number Failed	Number Left
1.16400	*	.	.	.	40	1294
1.16600	*	.	.	.	40	1293
1.16900	*	.	.	.	40	1292
1.16900	*	.	.	.	40	1291
1.16900	*	.	.	.	40	1290
1.16900	*	.	.	.	40	1289
1.16900	*	.	.	.	40	1288
1.16900	*	.	.	.	40	1287
1.16900	*	.	.	.	40	1286
1.16900	*	.	.	.	40	1285
1.16900	*	.	.	.	40	1284
1.16900	*	.	.	.	40	1283
1.16900	*	.	.	.	40	1282
1.16900	*	.	.	.	40	1281
1.17700		0.9794	0.0206	0.00323	41	1280
1.18000	*	.	.	.	41	1279
1.18000	*	.	.	.	41	1278
1.18000	*	.	.	.	41	1277
1.18300	*	.	.	.	41	1276
1.18800	*	.	.	.	41	1275
1.18800	*	.	.	.	41	1274
1.18800	*	.	.	.	41	1273
1.18800	*	.	.	.	41	1272
1.18800	*	.	.	.	41	1271
1.18800	*	.	.	.	41	1270
1.18800	*	.	.	.	41	1269
1.19400	*	.	.	.	41	1268
1.19600	*	.	.	.	41	1267
1.19600	*	.	.	.	41	1266

phregparms_mianalyze_mi_4099

The LIFETEST Procedure

Stratum 3: Statin intensity at study enrollment = Medium-intensity statin dose

Product-Limit Survival Estimates						
Prim_Outc_Time		Survival	Failure	Survival Standard Error	Number Failed	Number Left
1.19900	*	.	.	.	41	1265
1.19900	*	.	.	.	41	1264
1.19900	*	.	.	.	41	1263
1.19900	*	.	.	.	41	1262
1.20200	*	.	.	.	41	1261
1.20200	*	.	.	.	41	1260
1.20200	*	.	.	.	41	1259
1.20200	*	.	.	.	41	1258
1.20200	*	.	.	.	41	1257
1.20200	*	.	.	.	41	1256
1.20200	*	.	.	.	41	1255
1.20200	*	.	.	.	41	1254
1.20700	*	.	.	.	41	1253
1.20700	*	.	.	.	41	1252
1.20700	*	.	.	.	41	1251
1.20700	*	.	.	.	41	1250
1.20700	*	.	.	.	41	1249
1.20700	*	.	.	.	41	1248
1.20700	*	.	.	.	41	1247
1.20700	*	.	.	.	41	1246
1.20700	*	.	.	.	41	1245
1.21000	*	.	.	.	41	1244
1.21300	*	.	.	.	41	1243
1.21300	*	.	.	.	41	1242
1.21600	*	.	.	.	41	1241
1.21800	*	.	.	.	41	1240
1.22400	*	.	.	.	41	1239
1.22700	*	.	.	.	41	1238
1.22700	*	.	.	.	41	1237

phregparms_mianalyze_mi_4099

The LIFETEST Procedure

Stratum 3: Statin intensity at study enrollment = Medium-intensity statin dose

Product-Limit Survival Estimates						
Prim_Outc_Time		Survival	Failure	Survival Standard Error	Number Failed	Number Left
1.22700	*	.	.	.	41	1236
1.22700	*	.	.	.	41	1235
1.22700	*	.	.	.	41	1234
1.22700	*	.	.	.	41	1233
1.22700	*	.	.	.	41	1232
1.22700	*	.	.	.	41	1231
1.22700	*	.	.	.	41	1230
1.22700	*	.	.	.	41	1229
1.22700	*	.	.	.	41	1228
1.22700	*	.	.	.	41	1227
1.22700	*	.	.	.	41	1226
1.22700	*	.	.	.	41	1225
1.23200		0.9786	0.0214	0.00333	42	1224
1.23500	*	.	.	.	42	1223
1.23800	*	.	.	.	42	1222
1.24000	*	.	.	.	42	1221
1.24600	*	.	.	.	42	1220
1.24600	*	.	.	.	42	1219
1.24600	*	.	.	.	42	1218
1.24600	*	.	.	.	42	1217
1.24600	*	.	.	.	42	1216
1.25400	*	.	.	.	42	1215
1.26200		0.9778	0.0222	0.00342	43	1214
1.27900	*	.	.	.	43	1213
1.30600	*	.	.	.	43	1212
1.31100	*	.	.	.	43	1211
1.31100	*	.	.	.	43	1210
1.31100	*	.	.	.	43	1209
1.32000	*	.	.	.	43	1208

phregparms_mianalyze_mi_4099

The LIFETEST Procedure

Stratum 3: Statin intensity at study enrollment = Medium-intensity statin dose

Product-Limit Survival Estimates						
Prim_Outc_Time		Survival	Failure	Survival Standard Error	Number Failed	Number Left
1.32500		0.9770	0.0230	0.00351	44	1207
1.34200	*	.	.	.	44	1206
1.36600	*	.	.	.	44	1205
1.38000	*	.	.	.	44	1204
1.38000	*	.	.	.	44	1203
1.38500	*	.	.	.	44	1202
1.39900	*	.	.	.	44	1201
1.39900	*	.	.	.	44	1200
1.40200	*	.	.	.	44	1199
1.40200	*	.	.	.	44	1198
1.41000	*	.	.	.	44	1197
1.41800	*	.	.	.	44	1196
1.44800	*	.	.	.	44	1195
1.45700	*	.	.	.	44	1194
1.47600	*	.	.	.	44	1193
1.47800	*	.	.	.	44	1192
1.48400	*	.	.	.	44	1191
1.48700	*	.	.	.	44	1190
1.49800	*	.	.	.	44	1189
1.49800	*	.	.	.	44	1188
1.50300	*	.	.	.	44	1187
1.52000	*	.	.	.	44	1186
1.53600	*	.	.	.	44	1185
1.55200	*	.	.	.	44	1184
1.55200	*	.	.	.	44	1183
1.57200	*	.	.	.	44	1182
1.57200	*	.	.	.	44	1181
1.57700	*	.	.	.	44	1180
1.58500	*	.	.	.	44	1179

phregparms_mianalyze_mi_4099

The LIFETEST Procedure

Stratum 3: Statin intensity at study enrollment = Medium-intensity statin dose

Product-Limit Survival Estimates						
Prim_Outc_Time		Survival	Failure	Survival Standard Error	Number Failed	Number Left
1.59100	*	.	.	.	44	1178
1.59100	*	.	.	.	44	1177
1.59600	*	.	.	.	44	1176
1.60400	*	.	.	.	44	1175
1.61000	*	.	.	.	44	1174
1.61300	*	.	.	.	44	1173
1.61500	*	.	.	.	44	1172
1.61800	*	.	.	.	44	1171
1.62900	*	.	.	.	44	1170
1.62900	*	.	.	.	44	1169
1.62900	*	.	.	.	44	1168
1.63200	*	.	.	.	44	1167
1.64800	*	.	.	.	44	1166
1.64800	*	.	.	.	44	1165
1.65100	*	.	.	.	44	1164
1.65400		0.9762	0.0238	0.00361	45	1163
1.65400	*	.	.	.	45	1162
1.67000	*	.	.	.	45	1161
1.68700	*	.	.	.	45	1160
1.70600	*	.	.	.	45	1159
1.70800	*	.	.	.	45	1158
1.71100	*	.	.	.	45	1157
1.71400	*	.	.	.	45	1156
1.71900	*	.	.	.	45	1155
1.72200	*	.	.	.	45	1154
1.72500	*	.	.	.	45	1153
1.73000	*	.	.	.	45	1152
1.73000	*	.	.	.	45	1151
1.73600	*	.	.	.	45	1150

phregparms_mianalyze_mi_4099

The LIFETEST Procedure

Stratum 3: Statin intensity at study enrollment = Medium-intensity statin dose

Product-Limit Survival Estimates						
Prim_Outc_Time		Survival	Failure	Survival Standard Error	Number Failed	Number Left
1.73600	*	.	.	.	45	1149
1.74900	*	.	.	.	45	1148
1.75800	*	.	.	.	45	1147
1.75800	*	.	.	.	45	1146
1.76300	*	.	.	.	45	1145
1.76300	*	.	.	.	45	1144
1.76300	*	.	.	.	45	1143
1.76300	*	.	.	.	45	1142
1.76600	*	.	.	.	45	1141
1.76600	*	.	.	.	45	1140
1.76600	*	.	.	.	45	1139
1.76900	*	.	.	.	45	1138
1.77700	*	.	.	.	45	1137
1.77700	*	.	.	.	45	1136
1.78000	*	.	.	.	45	1135
1.78200	*	.	.	.	45	1134
1.78200	*	.	.	.	45	1133
1.78200	*	.	.	.	45	1132
1.78200	*	.	.	.	45	1131
1.79100	*	.	.	.	45	1130
1.79100	*	.	.	.	45	1129
1.79300	*	.	.	.	45	1128
1.79900		0.9753	0.0247	0.00371	46	1127
1.80200	*	.	.	.	46	1126
1.80200	*	.	.	.	46	1125
1.80200	*	.	.	.	46	1124
1.80200	*	.	.	.	46	1123
1.80200	*	.	.	.	46	1122
1.80400	*	.	.	.	46	1121

phregparms_mianalyze_mi_4099

The LIFETEST Procedure

Stratum 3: Statin intensity at study enrollment = Medium-intensity statin dose

Product-Limit Survival Estimates						
Prim_Outc_Time		Survival	Failure	Survival Standard Error	Number Failed	Number Left
1.80700	*	.	.	.	46	1120
1.80700	*	.	.	.	46	1119
1.81000	*	.	.	.	46	1118
1.81200	*	.	.	.	46	1117
1.81500	*	.	.	.	46	1116
1.81500	*	.	.	.	46	1115
1.81800	*	.	.	.	46	1114
1.82100	*	.	.	.	46	1113
1.82100	*	.	.	.	46	1112
1.82100	*	.	.	.	46	1111
1.82100	*	.	.	.	46	1110
1.82100	*	.	.	.	46	1109
1.82300	*	.	.	.	46	1108
1.82300	*	.	.	.	46	1107
1.82600	*	.	.	.	46	1106
1.82900	*	.	.	.	46	1105
1.83400	*	.	.	.	46	1104
1.83400	*	.	.	.	46	1103
1.83400	*	.	.	.	46	1102
1.83400	*	.	.	.	46	1101
1.84000	*	.	.	.	46	1100
1.84000	*	.	.	.	46	1099
1.84000	*	.	.	.	46	1098
1.84000	*	.	.	.	46	1097
1.84000	*	.	.	.	46	1096
1.84000	*	.	.	.	46	1095
1.84000	*	.	.	.	46	1094
1.84000	*	.	.	.	46	1093
1.84000	*	.	.	.	46	1092

phregparms_mianalyze_mi_4099

The LIFETEST Procedure

Stratum 3: Statin intensity at study enrollment = Medium-intensity statin dose

Product-Limit Survival Estimates						
Prim_Outc_Time		Survival	Failure	Survival Standard Error	Number Failed	Number Left
1.84000	*	.	.	.	46	1091
1.84000	*	.	.	.	46	1090
1.84300	*	.	.	.	46	1089
1.84500	*	.	.	.	46	1088
1.85400	*	.	.	.	46	1087
1.85600	*	.	.	.	46	1086
1.85600	*	.	.	.	46	1085
1.85900	*	.	.	.	46	1084
1.85900	*	.	.	.	46	1083
1.86400	*	.	.	.	46	1082
1.86700	*	.	.	.	46	1081
1.87000	*	.	.	.	46	1080
1.87300	*	.	.	.	46	1079
1.87300	*	.	.	.	46	1078
1.87800	*	.	.	.	46	1077
1.87800	*	.	.	.	46	1076
1.87800	*	.	.	.	46	1075
1.88100	*	.	.	.	46	1074
1.88100	*	.	.	.	46	1073
1.88100	*	.	.	.	46	1072
1.88400	*	.	.	.	46	1071
1.88400	*	.	.	.	46	1070
1.88600	*	.	.	.	46	1069
1.88600	*	.	.	.	46	1068
1.88600	*	.	.	.	46	1067
1.88900	*	.	.	.	46	1066
1.89200	*	.	.	.	46	1065
1.89700	*	.	.	.	46	1064
1.90000	*	.	.	.	46	1063

phregparms_mianalyze_mi_4099

The LIFETEST Procedure

Stratum 3: Statin intensity at study enrollment = Medium-intensity statin dose

Product-Limit Survival Estimates						
Prim_Outc_Time		Survival	Failure	Survival Standard Error	Number Failed	Number Left
1.90000	*	.	.	.	46	1062
1.90300	*	.	.	.	46	1061
1.91600	*	.	.	.	46	1060
1.91600	*	.	.	.	46	1059
1.91600	*	.	.	.	46	1058
1.91600	*	.	.	.	46	1057
1.91600	*	.	.	.	46	1056
1.91600	*	.	.	.	46	1055
1.91600	*	.	.	.	46	1054
1.91600	*	.	.	.	46	1053
1.91900	*	.	.	.	46	1052
1.92200	*	.	.	.	46	1051
1.93000	*	.	.	.	46	1050
1.93600	*	.	.	.	46	1049
1.93600	*	.	.	.	46	1048
1.93600	*	.	.	.	46	1047
1.93800	*	.	.	.	46	1046
1.94400	*	.	.	.	46	1045
1.94400	*	.	.	.	46	1044
1.94700	*	.	.	.	46	1043
1.94900	*	.	.	.	46	1042
1.95200	*	.	.	.	46	1041
1.95500	*	.	.	.	46	1040
1.95500	*	.	.	.	46	1039
1.95500	*	.	.	.	46	1038
1.95500	*	.	.	.	46	1037
1.95800	*	.	.	.	46	1036
1.96000	*	.	.	.	46	1035
1.96300	*	.	.	.	46	1034

phregparms_mianalyze_mi_4099

The LIFETEST Procedure

Stratum 3: Statin intensity at study enrollment = Medium-intensity statin dose

Product-Limit Survival Estimates						
Prim_Outc_Time		Survival	Failure	Survival Standard Error	Number Failed	Number Left
1.96600	*	.	.	.	46	1033
1.96600	*	.	.	.	46	1032
1.96900	*	.	.	.	46	1031
1.97400	*	.	.	.	46	1030
1.97400	*	.	.	.	46	1029
1.97400	*	.	.	.	46	1028
1.98200	*	.	.	.	46	1027
1.98500	*	.	.	.	46	1026
1.98800		0.9744	0.0256	0.00382	47	1025
1.99300	*	.	.	.	47	1024
1.99300	*	.	.	.	47	1023
1.99300	*	.	.	.	47	1022
1.99300	*	.	.	.	47	1021
1.99300	*	.	.	.	47	1020
1.99300	*	.	.	.	47	1019
1.99600	*	.	.	.	47	1018
1.99600	*	.	.	.	47	1017
1.99900	*	.	.	.	47	1016
2.00100	*	.	.	.	47	1015
2.00100	*	.	.	.	47	1014
2.00400	*	.	.	.	47	1013
2.00400	*	.	.	.	47	1012
2.00700	*	.	.	.	47	1011
2.01500	*	.	.	.	47	1010
2.02100	*	.	.	.	47	1009
2.02600	*	.	.	.	47	1008
2.03100	*	.	.	.	47	1007
2.03700	*	.	.	.	47	1006
2.04000		0.9734	0.0266	0.00394	48	1005

phregparms_mianalyze_mi_4099

The LIFETEST Procedure

Stratum 3: Statin intensity at study enrollment = Medium-intensity statin dose

Product-Limit Survival Estimates						
Prim_Outc_Time		Survival	Failure	Survival Standard Error	Number Failed	Number Left
2.04000	*	.	.	.	48	1004
2.04200	*	.	.	.	48	1003
2.04200	*	.	.	.	48	1002
2.04200	*	.	.	.	48	1001
2.05100	*	.	.	.	48	1000
2.05900	*	.	.	.	48	999
2.06700	*	.	.	.	48	998
2.07000	*	.	.	.	48	997
2.07300	*	.	.	.	48	996
2.07300	*	.	.	.	48	995
2.08100	*	.	.	.	48	994
2.08400	*	.	.	.	48	993
2.08600	*	.	.	.	48	992
2.09200	*	.	.	.	48	991
2.09400	*	.	.	.	48	990
2.11100	*	.	.	.	48	989
2.15200	*	.	.	.	48	988
2.16600	*	.	.	.	48	987
2.16800	*	.	.	.	48	986
2.16800	*	.	.	.	48	985
2.16800	*	.	.	.	48	984
2.16800	*	.	.	.	48	983
2.16800	*	.	.	.	48	982
2.16800	*	.	.	.	48	981
2.16800	*	.	.	.	48	980
2.17100		0.9724	0.0276	0.00406	49	979
2.17100	*	.	.	.	49	978
2.17400		0.9714	0.0286	0.00418	50	977
2.18500	*	.	.	.	50	976

phregparms_mianalyze_mi_4099

The LIFETEST Procedure

Stratum 3: Statin intensity at study enrollment = Medium-intensity statin dose

Product-Limit Survival Estimates						
Prim_Outc_Time		Survival	Failure	Survival Standard Error	Number Failed	Number Left
2.19600	*	.	.	.	50	975
2.19600	*	.	.	.	50	974
2.20700		0.9704	0.0296	0.00429	51	973
2.22900	*	.	.	.	51	972
2.24200	*	.	.	.	51	971
2.24500	*	.	.	.	51	970
2.27000	*	.	.	.	51	969
2.27000	*	.	.	.	51	968
2.27500		0.9694	0.0306	0.00440	52	967
2.28600	*	.	.	.	52	966
2.30000	*	.	.	.	52	965
2.30000	*	.	.	.	52	964
2.30000	*	.	.	.	52	963
2.30000	*	.	.	.	52	962
2.30300	*	.	.	.	52	961
2.30500	*	.	.	.	52	960
2.31600	*	.	.	.	52	959
2.33300	*	.	.	.	52	958
2.33500	*	.	.	.	52	957
2.33800	*	.	.	.	52	956
2.33800	*	.	.	.	52	955
2.34400	*	.	.	.	52	954
2.34400	*	.	.	.	52	953
2.34400	*	.	.	.	52	952
2.34900	*	.	.	.	52	951
2.35200	*	.	.	.	52	950
2.35700	*	.	.	.	52	949
2.35700	*	.	.	.	52	948
2.35700	*	.	.	.	52	947

phregparms_mianalyze_mi_4099

The LIFETEST Procedure

Stratum 3: Statin intensity at study enrollment = Medium-intensity statin dose

Product-Limit Survival Estimates						
Prim_Outc_Time		Survival	Failure	Survival Standard Error	Number Failed	Number Left
2.36000	*	.	.	.	52	946
2.36600	*	.	.	.	52	945
2.37100		0.9684	0.0316	0.00451	53	944
2.37600	*	.	.	.	53	943
2.37900		0.9673	0.0327	0.00462	54	942
2.39600	*	.	.	.	54	941
2.39600	*	.	.	.	54	940
2.39800	*	.	.	.	54	939
2.41500	*	.	.	.	54	938
2.41500	*	.	.	.	54	937
2.41500	*	.	.	.	54	936
2.41500	*	.	.	.	54	935
2.42300	*	.	.	.	54	934
2.43100	*	.	.	.	54	933
2.43400	*	.	.	.	54	932
2.43400	*	.	.	.	54	931
2.43400	*	.	.	.	54	930
2.43700	*	.	.	.	54	929
2.45000	*	.	.	.	54	928
2.45300	*	.	.	.	54	927
2.45300	*	.	.	.	54	926
2.45300	*	.	.	.	54	925
2.45900		0.9663	0.0337	0.00474	55	924
2.47200	*	.	.	.	55	923
2.47500	*	.	.	.	55	922
2.47500	*	.	.	.	55	921
2.47800	*	.	.	.	55	920
2.47800	*	.	.	.	55	919
2.48300	*	.	.	.	55	918

phregparms_mianalyze_mi_4099

The LIFETEST Procedure

Stratum 3: Statin intensity at study enrollment = Medium-intensity statin dose

Product-Limit Survival Estimates						
Prim_Outc_Time		Survival	Failure	Survival Standard Error	Number Failed	Number Left
2.49100	*	.	.	.	55	917
2.49100	*	.	.	.	55	916
2.49700	*	.	.	.	55	915
2.50000	*	.	.	.	55	914
2.50200	*	.	.	.	55	913
2.50200	*	.	.	.	55	912
2.50200	*	.	.	.	55	911
2.50500	*	.	.	.	55	910
2.50500	*	.	.	.	55	909
2.50800	*	.	.	.	55	908
2.51100	*	.	.	.	55	907
2.51100	*	.	.	.	55	906
2.51100	*	.	.	.	55	905
2.51100	*	.	.	.	55	904
2.51100	*	.	.	.	55	903
2.51100	*	.	.	.	55	902
2.51100	*	.	.	.	55	901
2.51100	*	.	.	.	55	900
2.51100	*	.	.	.	55	899
2.51100	*	.	.	.	55	898
2.51100	*	.	.	.	55	897
2.51300	*	.	.	.	55	896
2.51600	*	.	.	.	55	895
2.52400	*	.	.	.	55	894
2.52700	*	.	.	.	55	893
2.53000	*	.	.	.	55	892
2.53000	*	.	.	.	55	891
2.53000	*	.	.	.	55	890
2.53000	*	.	.	.	55	889

phregparms_mianalyze_mi_4099

The LIFETEST Procedure

Stratum 3: Statin intensity at study enrollment = Medium-intensity statin dose

Product-Limit Survival Estimates						
Prim_Outc_Time		Survival	Failure	Survival Standard Error	Number Failed	Number Left
2.53000	*	.	.	.	55	888
2.53000	*	.	.	.	55	887
2.53000	*	.	.	.	55	886
2.53000	*	.	.	.	55	885
2.53000	*	.	.	.	55	884
2.53000	*	.	.	.	55	883
2.53300		0.9652	0.0348	0.00486	56	882
2.54100	*	.	.	.	56	881
2.54100	*	.	.	.	56	880
2.54300	*	.	.	.	56	879
2.54300	*	.	.	.	56	878
2.54900	*	.	.	.	56	877
2.54900	*	.	.	.	56	876
2.54900	*	.	.	.	56	875
2.54900	*	.	.	.	56	874
2.54900	*	.	.	.	56	873
2.54900	*	.	.	.	56	872
2.54900	*	.	.	.	56	871
2.54900	*	.	.	.	56	870
2.54900	*	.	.	.	56	869
2.54900	*	.	.	.	56	868
2.55200	*	.	.	.	56	867
2.55400	*	.	.	.	56	866
2.55400	*	.	.	.	56	865
2.55400	*	.	.	.	56	864
2.55400	*	.	.	.	56	863
2.55400	*	.	.	.	56	862
2.55400	*	.	.	.	56	861
2.55700	*	.	.	.	56	860

phregparms_mianalyze_mi_4099

The LIFETEST Procedure

Stratum 3: Statin intensity at study enrollment = Medium-intensity statin dose

Product-Limit Survival Estimates						
Prim_Outc_Time		Survival	Failure	Survival Standard Error	Number Failed	Number Left
2.55700	*	.	.	.	56	859
2.56000	*	.	.	.	56	858
2.56000	*	.	.	.	56	857
2.56000	*	.	.	.	56	856
2.56500	*	.	.	.	56	855
2.56500	*	.	.	.	56	854
2.56500	*	.	.	.	56	853
2.56800	*	.	.	.	56	852
2.56800	*	.	.	.	56	851
2.56800	*	.	.	.	56	850
2.56800	*	.	.	.	56	849
2.56800	*	.	.	.	56	848
2.56800	*	.	.	.	56	847
2.56800	*	.	.	.	56	846
2.56800	*	.	.	.	56	845
2.56800	*	.	.	.	56	844
2.56800	*	.	.	.	56	843
2.57100	*	.	.	.	56	842
2.57400	*	.	.	.	56	841
2.57600	*	.	.	.	56	840
2.57900	*	.	.	.	56	839
2.58200	*	.	.	.	56	838
2.58200	*	.	.	.	56	837
2.58200	*	.	.	.	56	836
2.58700	*	.	.	.	56	835
2.58700	*	.	.	.	56	834
2.58700	*	.	.	.	56	833
2.58700	*	.	.	.	56	832
2.58700	*	.	.	.	56	831

phregparms_mianalyze_mi_4099

The LIFETEST Procedure

Stratum 3: Statin intensity at study enrollment = Medium-intensity statin dose

Product-Limit Survival Estimates						
Prim_Outc_Time		Survival	Failure	Survival Standard Error	Number Failed	Number Left
2.58700	*	.	.	.	56	830
2.58700	*	.	.	.	56	829
2.58700	*	.	.	.	56	828
2.58700	*	.	.	.	56	827
2.58700	*	.	.	.	56	826
2.58700	*	.	.	.	56	825
2.58700	*	.	.	.	56	824
2.59300	*	.	.	.	56	823
2.59500	*	.	.	.	56	822
2.59500	*	.	.	.	56	821
2.60400	*	.	.	.	56	820
2.60400	*	.	.	.	56	819
2.60600	*	.	.	.	56	818
2.60600	*	.	.	.	56	817
2.60600	*	.	.	.	56	816
2.60600	*	.	.	.	56	815
2.60600	*	.	.	.	56	814
2.60600	*	.	.	.	56	813
2.60600	*	.	.	.	56	812
2.60600	*	.	.	.	56	811
2.60600	*	.	.	.	56	810
2.60600	*	.	.	.	56	809
2.60900	*	.	.	.	56	808
2.61500	*	.	.	.	56	807
2.61500	*	.	.	.	56	806
2.61500	*	.	.	.	56	805
2.62300	*	.	.	.	56	804
2.62600	*	.	.	.	56	803
2.62600	*	.	.	.	56	802

phregparms_mianalyze_mi_4099

The LIFETEST Procedure

Stratum 3: Statin intensity at study enrollment = Medium-intensity statin dose

Product-Limit Survival Estimates						
Prim_Outc_Time		Survival	Failure	Survival Standard Error	Number Failed	Number Left
2.62600	*	.	.	.	56	801
2.62600	*	.	.	.	56	800
2.62600	*	.	.	.	56	799
2.62600	*	.	.	.	56	798
2.62600	*	.	.	.	56	797
2.62600	*	.	.	.	56	796
2.62600	*	.	.	.	56	795
2.62600	*	.	.	.	56	794
2.62600	*	.	.	.	56	793
2.63100	*	.	.	.	56	792
2.63700	*	.	.	.	56	791
2.64500	*	.	.	.	56	790
2.64500	*	.	.	.	56	789
2.64500	*	.	.	.	56	788
2.64500	*	.	.	.	56	787
2.64800	*	.	.	.	56	786
2.64800	*	.	.	.	56	785
2.65000	*	.	.	.	56	784
2.65000	*	.	.	.	56	783
2.65800	*	.	.	.	56	782
2.66100	*	.	.	.	56	781
2.66100	*	.	.	.	56	780
2.66100	*	.	.	.	56	779
2.66400	*	.	.	.	56	778
2.66400	*	.	.	.	56	777
2.66400	*	.	.	.	56	776
2.66400	*	.	.	.	56	775
2.66400	*	.	.	.	56	774
2.66400	*	.	.	.	56	773

phregparms_mianalyze_mi_4099

The LIFETEST Procedure

Stratum 3: Statin intensity at study enrollment = Medium-intensity statin dose

Product-Limit Survival Estimates						
Prim_Outc_Time		Survival	Failure	Survival Standard Error	Number Failed	Number Left
2.66400	*	.	.	.	56	772
2.66400	*	.	.	.	56	771
2.66700	*	.	.	.	56	770
2.67500	*	.	.	.	56	769
2.68300	*	.	.	.	56	768
2.68300	*	.	.	.	56	767
2.68300	*	.	.	.	56	766
2.68300	*	.	.	.	56	765
2.68300	*	.	.	.	56	764
2.68300	*	.	.	.	56	763
2.68300	*	.	.	.	56	762
2.68300	*	.	.	.	56	761
2.68600	*	.	.	.	56	760
2.68600	*	.	.	.	56	759
2.68900	*	.	.	.	56	758
2.68900	*	.	.	.	56	757
2.69700	*	.	.	.	56	756
2.70000	*	.	.	.	56	755
2.70000	*	.	.	.	56	754
2.70200	*	.	.	.	56	753
2.70200	*	.	.	.	56	752
2.70200	*	.	.	.	56	751
2.70200	*	.	.	.	56	750
2.70200	*	.	.	.	56	749
2.70200	*	.	.	.	56	748
2.70500	*	.	.	.	56	747
2.70500	*	.	.	.	56	746
2.71000	*	.	.	.	56	745
2.72100	*	.	.	.	56	744

phregparms_mianalyze_mi_4099

The LIFETEST Procedure

Stratum 3: Statin intensity at study enrollment = Medium-intensity statin dose

Product-Limit Survival Estimates						
Prim_Outc_Time		Survival	Failure	Survival Standard Error	Number Failed	Number Left
2.72100	*	.	.	.	56	743
2.72100	*	.	.	.	56	742
2.72100	*	.	.	.	56	741
2.72100	*	.	.	.	56	740
2.72100	*	.	.	.	56	739
2.72100	*	.	.	.	56	738
2.72700	*	.	.	.	56	737
2.73000	*	.	.	.	56	736
2.73200	*	.	.	.	56	735
2.73800	*	.	.	.	56	734
2.73800	*	.	.	.	56	733
2.74100	*	.	.	.	56	732
2.74100	*	.	.	.	56	731
2.74100	*	.	.	.	56	730
2.74100	*	.	.	.	56	729
2.74100	*	.	.	.	56	728
2.74100	*	.	.	.	56	727
2.74100	*	.	.	.	56	726
2.74100	*	.	.	.	56	725
2.74100	*	.	.	.	56	724
2.74100	*	.	.	.	56	723
2.74100	*	.	.	.	56	722
2.74100	*	.	.	.	56	721
2.74100	*	.	.	.	56	720
2.74100	*	.	.	.	56	719
2.74100	*	.	.	.	56	718
2.74100	*	.	.	.	56	717
2.74100	*	.	.	.	56	716
2.74100	*	.	.	.	56	715

phregparms_mianalyze_mi_4099

The LIFETEST Procedure

Stratum 3: Statin intensity at study enrollment = Medium-intensity statin dose

Product-Limit Survival Estimates						
Prim_Outc_Time		Survival	Failure	Survival Standard Error	Number Failed	Number Left
2.74100	*	.	.	.	56	714
2.74100	*	.	.	.	56	713
2.74900	*	.	.	.	56	712
2.75400	*	.	.	.	56	711
2.75700	*	.	.	.	56	710
2.76000	*	.	.	.	56	709
2.76000	*	.	.	.	56	708
2.76000	*	.	.	.	56	707
2.76000	*	.	.	.	56	706
2.76000	*	.	.	.	56	705
2.76000	*	.	.	.	56	704
2.76000	*	.	.	.	56	703
2.76000	*	.	.	.	56	702
2.76000	*	.	.	.	56	701
2.76000	*	.	.	.	56	700
2.76000	*	.	.	.	56	699
2.76000	*	.	.	.	56	698
2.76000	*	.	.	.	56	697
2.76000	*	.	.	.	56	696
2.76000	*	.	.	.	56	695
2.76000	*	.	.	.	56	694
2.76000	*	.	.	.	56	693
2.76000	*	.	.	.	56	692
2.76000	*	.	.	.	56	691
2.76000	*	.	.	.	56	690
2.76000	*	.	.	.	56	689
2.76000	*	.	.	.	56	688
2.76000	*	.	.	.	56	687
2.76200	*	.	.	.	56	686

phregparms_mianalyze_mi_4099

The LIFETEST Procedure

Stratum 3: Statin intensity at study enrollment = Medium-intensity statin dose

Product-Limit Survival Estimates						
Prim_Outc_Time		Survival	Failure	Survival Standard Error	Number Failed	Number Left
2.76500	*	.	.	.	56	685
2.77100	*	.	.	.	56	684
2.77100	*	.	.	.	56	683
2.77900	*	.	.	.	56	682
2.77900	*	.	.	.	56	681
2.77900	*	.	.	.	56	680
2.77900	*	.	.	.	56	679
2.77900	*	.	.	.	56	678
2.77900	*	.	.	.	56	677
2.77900	*	.	.	.	56	676
2.77900	*	.	.	.	56	675
2.77900	*	.	.	.	56	674
2.77900	*	.	.	.	56	673
2.77900	*	.	.	.	56	672
2.77900	*	.	.	.	56	671
2.78200	*	.	.	.	56	670
2.78200	*	.	.	.	56	669
2.78200	*	.	.	.	56	668
2.78700	*	.	.	.	56	667
2.79500		0.9638	0.0362	0.00506	57	666
2.79500	*	.	.	.	57	665
2.79500	*	.	.	.	57	664
2.79800	*	.	.	.	57	663
2.79800	*	.	.	.	57	662
2.79800	*	.	.	.	57	661
2.79800	*	.	.	.	57	660
2.79800	*	.	.	.	57	659
2.79800	*	.	.	.	57	658
2.79800	*	.	.	.	57	657

phregparms_mianalyze_mi_4099

The LIFETEST Procedure

Stratum 3: Statin intensity at study enrollment = Medium-intensity statin dose

Product-Limit Survival Estimates						
Prim_Outc_Time		Survival	Failure	Survival Standard Error	Number Failed	Number Left
2.79800	*	.	.	.	57	656
2.79800	*	.	.	.	57	655
2.79800	*	.	.	.	57	654
2.79800	*	.	.	.	57	653
2.80100	*	.	.	.	57	652
2.80400	*	.	.	.	57	651
2.80900	*	.	.	.	57	650
2.81500	*	.	.	.	57	649
2.81700	*	.	.	.	57	648
2.81700	*	.	.	.	57	647
2.81700	*	.	.	.	57	646
2.81700	*	.	.	.	57	645
2.81700	*	.	.	.	57	644
2.81700	*	.	.	.	57	643
2.81700	*	.	.	.	57	642
2.81700	*	.	.	.	57	641
2.81700	*	.	.	.	57	640
2.82300	*	.	.	.	57	639
2.82500	*	.	.	.	57	638
2.82500	*	.	.	.	57	637
2.83400	*	.	.	.	57	636
2.83600	*	.	.	.	57	635
2.83600	*	.	.	.	57	634
2.83600	*	.	.	.	57	633
2.83600	*	.	.	.	57	632
2.83600	*	.	.	.	57	631
2.83600	*	.	.	.	57	630
2.83600	*	.	.	.	57	629
2.83600	*	.	.	.	57	628

phregparms_mianalyze_mi_4099

The LIFETEST Procedure

Stratum 3: Statin intensity at study enrollment = Medium-intensity statin dose

Product-Limit Survival Estimates						
Prim_Outc_Time		Survival	Failure	Survival Standard Error	Number Failed	Number Left
2.83600	*	.	.	.	57	627
2.83900	*	.	.	.	57	626
2.84500	*	.	.	.	57	625
2.84700	*	.	.	.	57	624
2.84700	*	.	.	.	57	623
2.85000	*	.	.	.	57	622
2.85300	*	.	.	.	57	621
2.85600	*	.	.	.	57	620
2.85600	*	.	.	.	57	619
2.85600	*	.	.	.	57	618
2.85600	*	.	.	.	57	617
2.85800	*	.	.	.	57	616
2.86400	*	.	.	.	57	615
2.86700	*	.	.	.	57	614
2.87500		0.9622	0.0378	0.00529	58	613
2.87500	*	.	.	.	58	612
2.87500	*	.	.	.	58	611
2.87500	*	.	.	.	58	610
2.87500	*	.	.	.	58	609
2.87500	*	.	.	.	58	608
2.87500	*	.	.	.	58	607
2.87500	*	.	.	.	58	606
2.87500	*	.	.	.	58	605
2.88000	*	.	.	.	58	604
2.89400	*	.	.	.	58	603
2.89400	*	.	.	.	58	602
2.89400	*	.	.	.	58	601
2.89400	*	.	.	.	58	600
2.89400	*	.	.	.	58	599

phregparms_mianalyze_mi_4099

The LIFETEST Procedure

Stratum 3: Statin intensity at study enrollment = Medium-intensity statin dose

Product-Limit Survival Estimates						
Prim_Outc_Time		Survival	Failure	Survival Standard Error	Number Failed	Number Left
2.89400	*	.	.	.	58	598
2.89400	*	.	.	.	58	597
2.89400	*	.	.	.	58	596
2.89900	*	.	.	.	58	595
2.90200	*	.	.	.	58	594
2.90500	*	.	.	.	58	593
2.90800	*	.	.	.	58	592
2.91000	*	.	.	.	58	591
2.91300	*	.	.	.	58	590
2.91300	*	.	.	.	58	589
2.91300	*	.	.	.	58	588
2.91300	*	.	.	.	58	587
2.91300	*	.	.	.	58	586
2.91300	*	.	.	.	58	585
2.91300	*	.	.	.	58	584
2.91300	*	.	.	.	58	583
2.91300	*	.	.	.	58	582
2.91900	*	.	.	.	58	581
2.92100	*	.	.	.	58	580
2.92100	*	.	.	.	58	579
2.92400	*	.	.	.	58	578
2.93200	*	.	.	.	58	577
2.93200	*	.	.	.	58	576
2.93200	*	.	.	.	58	575
2.93200	*	.	.	.	58	574
2.93200	*	.	.	.	58	573
2.93200	*	.	.	.	58	572
2.93200	*	.	.	.	58	571
2.93200	*	.	.	.	58	570

phregparms_mianalyze_mi_4099

The LIFETEST Procedure

Stratum 3: Statin intensity at study enrollment = Medium-intensity statin dose

Product-Limit Survival Estimates						
Prim_Outc_Time		Survival	Failure	Survival Standard Error	Number Failed	Number Left
2.93200	*	.	.	.	58	569
2.93200	*	.	.	.	58	568
2.93500	*	.	.	.	58	567
2.93800	*	.	.	.	58	566
2.95100	*	.	.	.	58	565
2.95100	*	.	.	.	58	564
2.95100	*	.	.	.	58	563
2.95100	*	.	.	.	58	562
2.95100	*	.	.	.	58	561
2.95100	*	.	.	.	58	560
2.95100	*	.	.	.	58	559
2.95700	*	.	.	.	58	558
2.96000	*	.	.	.	58	557
2.96500	*	.	.	.	58	556
2.97100	*	.	.	.	58	555
2.97100	*	.	.	.	58	554
2.97100	*	.	.	.	58	553
2.97100	*	.	.	.	58	552
2.97100	*	.	.	.	58	551
2.97100	*	.	.	.	58	550
2.97100	*	.	.	.	58	549
2.97100	*	.	.	.	58	548
2.97100	*	.	.	.	58	547
2.97100	*	.	.	.	58	546
2.97100	*	.	.	.	58	545
2.97100	*	.	.	.	58	544
2.97100	*	.	.	.	58	543
2.97100	*	.	.	.	58	542
2.97100	*	.	.	.	58	541

phregparms_mianalyze_mi_4099

The LIFETEST Procedure

Stratum 3: Statin intensity at study enrollment = Medium-intensity statin dose

Product-Limit Survival Estimates						
Prim_Outc_Time		Survival	Failure	Survival Standard Error	Number Failed	Number Left
2.97100	*	.	.	.	58	540
2.97300		0.9604	0.0396	0.00557	59	539
2.97300	*	.	.	.	59	538
2.97600	*	.	.	.	59	537
2.97600	*	.	.	.	59	536
2.97900	*	.	.	.	59	535
2.98200	*	.	.	.	59	534
2.98200	*	.	.	.	59	533
2.98400		0.9586	0.0414	0.00584	60	532
2.99000	*	.	.	.	60	531
2.99000	*	.	.	.	60	530
2.99000	*	.	.	.	60	529
2.99000	*	.	.	.	60	528
2.99000	*	.	.	.	60	527
2.99000	*	.	.	.	60	526
2.99000	*	.	.	.	60	525
2.99000	*	.	.	.	60	524
2.99000	*	.	.	.	60	523
2.99000	*	.	.	.	60	522
2.99000	*	.	.	.	60	521
2.99000	*	.	.	.	60	520
2.99000	*	.	.	.	60	519
2.99000	*	.	.	.	60	518
2.99000	*	.	.	.	60	517
2.99000	*	.	.	.	60	516
2.99000	*	.	.	.	60	515
2.99000	*	.	.	.	60	514
2.99000	*	.	.	.	60	513
2.99000	*	.	.	.	60	512

phregparms_mianalyze_mi_4099

The LIFETEST Procedure

Stratum 3: Statin intensity at study enrollment = Medium-intensity statin dose

Product-Limit Survival Estimates						
Prim_Outc_Time		Survival	Failure	Survival Standard Error	Number Failed	Number Left
2.99000	*	.	.	.	60	511
2.99000	*	.	.	.	60	510
2.99000	*	.	.	.	60	509
2.99200	*	.	.	.	60	508
2.99500	*	.	.	.	60	507
3.00100	*	.	.	.	60	506
3.00100	*	.	.	.	60	505
3.00100	*	.	.	.	60	504
3.00100	*	.	.	.	60	503
3.00100	*	.	.	.	60	502
3.00100	*	.	.	.	60	501
3.00100	*	.	.	.	60	500
3.00100	*	.	.	.	60	499
3.00100	*	.	.	.	60	498
3.00100	*	.	.	.	60	497
3.00100	*	.	.	.	60	496
3.00100	*	.	.	.	60	495
3.00100	*	.	.	.	60	494
3.00100	*	.	.	.	60	493
3.00100	*	.	.	.	60	492
3.00100	*	.	.	.	60	491
3.00100	*	.	.	.	60	490
3.00100	*	.	.	.	60	489
3.00100	*	.	.	.	60	488
3.00100	*	.	.	.	60	487
3.00300	*	.	.	.	60	486
3.00600	*	.	.	.	60	485
3.00900	*	.	.	.	60	484
3.00900	*	.	.	.	60	483

phregparms_mianalyze_mi_4099

The LIFETEST Procedure

Stratum 3: Statin intensity at study enrollment = Medium-intensity statin dose

Product-Limit Survival Estimates						
Prim_Outc_Time		Survival	Failure	Survival Standard Error	Number Failed	Number Left
3.00900	*	.	.	.	60	482
3.00900	*	.	.	.	60	481
3.00900	*	.	.	.	60	480
3.00900	*	.	.	.	60	479
3.00900	*	.	.	.	60	478
3.00900	*	.	.	.	60	477
3.00900	*	.	.	.	60	476
3.00900	*	.	.	.	60	475
3.01200	*	.	.	.	60	474
3.01400	*	.	.	.	60	473
3.02300	*	.	.	.	60	472
3.02800	*	.	.	.	60	471
3.02800	*	.	.	.	60	470
3.02800	*	.	.	.	60	469
3.02800	*	.	.	.	60	468
3.02800	*	.	.	.	60	467
3.02800	*	.	.	.	60	466
3.03100	*	.	.	.	60	465
3.03400	*	.	.	.	60	464
3.03600	*	.	.	.	60	463
3.03900		0.9565	0.0435	0.00619	61	462
3.03900	*	.	.	.	61	461
3.04700	*	.	.	.	61	460
3.04700	*	.	.	.	61	459
3.05000	*	.	.	.	61	458
3.05300	*	.	.	.	61	457
3.05300	*	.	.	.	61	456
3.06100	*	.	.	.	61	455
3.06400	*	.	.	.	61	454

phregparms_mianalyze_mi_4099

The LIFETEST Procedure

Stratum 3: Statin intensity at study enrollment = Medium-intensity statin dose

Product-Limit Survival Estimates						
Prim_Outc_Time		Survival	Failure	Survival Standard Error	Number Failed	Number Left
3.06600	*	.	.	.	61	453
3.06600	*	.	.	.	61	452
3.06600	*	.	.	.	61	451
3.06600	*	.	.	.	61	450
3.06600	*	.	.	.	61	449
3.06600	*	.	.	.	61	448
3.06600	*	.	.	.	61	447
3.07200	*	.	.	.	61	446
3.07500	*	.	.	.	61	445
3.07500	*	.	.	.	61	444
3.07500	*	.	.	.	61	443
3.07700	*	.	.	.	61	442
3.08600	*	.	.	.	61	441
3.08600	*	.	.	.	61	440
3.08600	*	.	.	.	61	439
3.08600	*	.	.	.	61	438
3.08600	*	.	.	.	61	437
3.09100	*	.	.	.	61	436
3.09100	*	.	.	.	61	435
3.09100	*	.	.	.	61	434
3.09400	*	.	.	.	61	433
3.09400	*	.	.	.	61	432
3.09700	*	.	.	.	61	431
3.09700	*	.	.	.	61	430
3.09700	*	.	.	.	61	429
3.09700	*	.	.	.	61	428
3.09700	*	.	.	.	61	427
3.09900	*	.	.	.	61	426
3.10500	*	.	.	.	61	425

phregparms_mianalyze_mi_4099

The LIFETEST Procedure

Stratum 3: Statin intensity at study enrollment = Medium-intensity statin dose

Product-Limit Survival Estimates						
Prim_Outc_Time		Survival	Failure	Survival Standard Error	Number Failed	Number Left
3.10500	*	.	.	.	61	424
3.10500	*	.	.	.	61	423
3.10500	*	.	.	.	61	422
3.11000	*	.	.	.	61	421
3.11300	*	.	.	.	61	420
3.12100	*	.	.	.	61	419
3.12100	*	.	.	.	61	418
3.12400	*	.	.	.	61	417
3.12400	*	.	.	.	61	416
3.12700		0.9542	0.0458	0.00659	62	415
3.13500	*	.	.	.	62	414
3.13800	*	.	.	.	62	413
3.14300	*	.	.	.	62	412
3.14300	*	.	.	.	62	411
3.14600	*	.	.	.	62	410
3.14900	*	.	.	.	62	409
3.14900	*	.	.	.	62	408
3.15100	*	.	.	.	62	407
3.15400	*	.	.	.	62	406
3.15700	*	.	.	.	62	405
3.15700	*	.	.	.	62	404
3.15900	*	.	.	.	62	403
3.16200	*	.	.	.	62	402
3.16200	*	.	.	.	62	401
3.16200	*	.	.	.	62	400
3.17900	*	.	.	.	62	399
3.18100	*	.	.	.	62	398
3.18400	*	.	.	.	62	397
3.18400	*	.	.	.	62	396

phregparms_mianalyze_mi_4099

The LIFETEST Procedure

Stratum 3: Statin intensity at study enrollment = Medium-intensity statin dose

Product-Limit Survival Estimates						
Prim_Outc_Time		Survival	Failure	Survival Standard Error	Number Failed	Number Left
3.19800	*	.	.	.	62	395
3.19800	*	.	.	.	62	394
3.20900	*	.	.	.	62	393
3.20900	*	.	.	.	62	392
3.21700	*	.	.	.	62	391
3.21700	*	.	.	.	62	390
3.22000	*	.	.	.	62	389
3.22000	*	.	.	.	62	388
3.22500	*	.	.	.	62	387
3.22800	*	.	.	.	62	386
3.23100	*	.	.	.	62	385
3.23300	*	.	.	.	62	384
3.23900	*	.	.	.	62	383
3.23900	*	.	.	.	62	382
3.25000	*	.	.	.	62	381
3.25000	*	.	.	.	62	380
3.25500	*	.	.	.	62	379
3.25800	*	.	.	.	62	378
3.26600	*	.	.	.	62	377
3.27200	*	.	.	.	62	376
3.27700	*	.	.	.	62	375
3.27700	*	.	.	.	62	374
3.27700	*	.	.	.	62	373
3.30200	*	.	.	.	62	372
3.30500	*	.	.	.	62	371
3.30500	*	.	.	.	62	370
3.32400	*	.	.	.	62	369
3.32900	*	.	.	.	62	368
3.32900	*	.	.	.	62	367

phregparms_mianalyze_mi_4099

The LIFETEST Procedure

Stratum 3: Statin intensity at study enrollment = Medium-intensity statin dose

Product-Limit Survival Estimates						
Prim_Outc_Time		Survival	Failure	Survival Standard Error	Number Failed	Number Left
3.33200	*	.	.	.	62	366
3.33500	*	.	.	.	62	365
3.33500	*	.	.	.	62	364
3.34300	*	.	.	.	62	363
3.35400	*	.	.	.	62	362
3.36200	*	.	.	.	62	361
3.36800		0.9516	0.0484	0.00708	63	360
3.36800	*	.	.	.	63	359
3.37300	*	.	.	.	63	358
3.38700	*	.	.	.	63	357
3.39200	*	.	.	.	63	356
3.39500	*	.	.	.	63	355
3.44400	*	.	.	.	63	354
3.45000	*	.	.	.	63	353
3.45000	*	.	.	.	63	352
3.45000	*	.	.	.	63	351
3.45000	*	.	.	.	63	350
3.45000	*	.	.	.	63	349
3.45000	*	.	.	.	63	348
3.45200	*	.	.	.	63	347
3.45800	*	.	.	.	63	346
3.46300	*	.	.	.	63	345
3.46300	*	.	.	.	63	344
3.46900	*	.	.	.	63	343
3.48500	*	.	.	.	63	342
3.48800	*	.	.	.	63	341
3.50200		0.9488	0.0512	0.00759	64	340
3.50700	*	.	.	.	64	339
3.50700	*	.	.	.	64	338

phregparms_mianalyze_mi_4099

The LIFETEST Procedure

Stratum 3: Statin intensity at study enrollment = Medium-intensity statin dose

Product-Limit Survival Estimates						
Prim_Outc_Time		Survival	Failure	Survival Standard Error	Number Failed	Number Left
3.51300	*	.	.	.	64	337
3.52400	*	.	.	.	64	336
3.52600	*	.	.	.	64	335
3.52600	*	.	.	.	64	334
3.52600	*	.	.	.	64	333
3.52600	*	.	.	.	64	332
3.52600	*	.	.	.	64	331
3.53500	*	.	.	.	64	330
3.54000	*	.	.	.	64	329
3.54600		0.9459	0.0541	0.00809	65	328
3.54600	*	.	.	.	65	327
3.54600	*	.	.	.	65	326
3.55400	*	.	.	.	65	325
3.56500	*	.	.	.	65	324
3.56500	*	.	.	.	65	323
3.56500	*	.	.	.	65	322
3.57000	*	.	.	.	65	321
3.57300	*	.	.	.	65	320
3.57800	*	.	.	.	65	319
3.57800	*	.	.	.	65	318
3.58700	*	.	.	.	65	317
3.58700	*	.	.	.	65	316
3.59200	*	.	.	.	65	315
3.60000	*	.	.	.	65	314
3.60000	*	.	.	.	65	313
3.60000	*	.	.	.	65	312
3.60000	*	.	.	.	65	311
3.60300	*	.	.	.	65	310
3.60300	*	.	.	.	65	309

phregparms_mianalyze_mi_4099

The LIFETEST Procedure

Stratum 3: Statin intensity at study enrollment = Medium-intensity statin dose

Product-Limit Survival Estimates						
Prim_Outc_Time		Survival	Failure	Survival Standard Error	Number Failed	Number Left
3.60300	*	.	.	.	65	308
3.62800	*	.	.	.	65	307
3.64100	*	.	.	.	65	306
3.64100	*	.	.	.	65	305
3.65000	*	.	.	.	65	304
3.65000	*	.	.	.	65	303
3.66100	*	.	.	.	65	302
3.67400	*	.	.	.	65	301
3.68000	*	.	.	.	65	300
3.68000	*	.	.	.	65	299
3.68000	*	.	.	.	65	298
3.68000	*	.	.	.	65	297
3.68000	*	.	.	.	65	296
3.68000	*	.	.	.	65	295
3.68000	*	.	.	.	65	294
3.68200	*	.	.	.	65	293
3.68500	*	.	.	.	65	292
3.68800	*	.	.	.	65	291
3.68800	*	.	.	.	65	290
3.69900	*	.	.	.	65	289
3.69900	*	.	.	.	65	288
3.70200	*	.	.	.	65	287
3.71800	*	.	.	.	65	286
3.71800	*	.	.	.	65	285
3.72100	*	.	.	.	65	284
3.73700		0.9426	0.0574	0.00872	66	283
3.73700	*	.	.	.	66	282
3.73700	*	.	.	.	66	281
3.74000	*	.	.	.	66	280

phregparms_mianalyze_mi_4099

The LIFETEST Procedure

Stratum 3: Statin intensity at study enrollment = Medium-intensity statin dose

Product-Limit Survival Estimates						
Prim_Outc_Time		Survival	Failure	Survival Standard Error	Number Failed	Number Left
3.74000	*	.	.	.	66	279
3.74000	*	.	.	.	66	278
3.75100	*	.	.	.	66	277
3.75600	*	.	.	.	66	276
3.75600	*	.	.	.	66	275
3.75600	*	.	.	.	66	274
3.75600	*	.	.	.	66	273
3.75900	*	.	.	.	66	272
3.76200	*	.	.	.	66	271
3.76500	*	.	.	.	66	270
3.77000	*	.	.	.	66	269
3.77300	*	.	.	.	66	268
3.77500	*	.	.	.	66	267
3.77500	*	.	.	.	66	266
3.77500	*	.	.	.	66	265
3.77800	*	.	.	.	66	264
3.77800	*	.	.	.	66	263
3.78100	*	.	.	.	66	262
3.78900		0.9390	0.0610	0.00940	67	261
3.78900	*	.	.	.	67	260
3.79200	*	.	.	.	67	259
3.79500	*	.	.	.	67	258
3.79500	*	.	.	.	67	257
3.80800	*	.	.	.	67	256
3.81100	*	.	.	.	67	255
3.81100	*	.	.	.	67	254
3.81100	*	.	.	.	67	253
3.81100	*	.	.	.	67	252
3.81400	*	.	.	.	67	251

phregparms_mianalyze_mi_4099

The LIFETEST Procedure

Stratum 3: Statin intensity at study enrollment = Medium-intensity statin dose

Product-Limit Survival Estimates						
Prim_Outc_Time		Survival	Failure	Survival Standard Error	Number Failed	Number Left
3.81400	*	.	.	.	67	250
3.81400	*	.	.	.	67	249
3.81400	*	.	.	.	67	248
3.81700	*	.	.	.	67	247
3.81900	*	.	.	.	67	246
3.81900	*	.	.	.	67	245
3.81900	*	.	.	.	67	244
3.82800	*	.	.	.	67	243
3.83000	*	.	.	.	67	242
3.83300	*	.	.	.	67	241
3.83300	*	.	.	.	67	240
3.83300	*	.	.	.	67	239
3.83300	*	.	.	.	67	238
3.83300	*	.	.	.	67	237
3.83300	*	.	.	.	67	236
3.83300	*	.	.	.	67	235
3.83300	*	.	.	.	67	234
3.83300	*	.	.	.	67	233
3.83300	*	.	.	.	67	232
3.83300	*	.	.	.	67	231
3.83300	*	.	.	.	67	230
3.83600	*	.	.	.	67	229
3.83800	*	.	.	.	67	228
3.83800	*	.	.	.	67	227
3.83800	*	.	.	.	67	226
3.84700	*	.	.	.	67	225
3.84900	*	.	.	.	67	224
3.84900	*	.	.	.	67	223
3.85200	*	.	.	.	67	222

phregparms_mianalyze_mi_4099

The LIFETEST Procedure

Stratum 3: Statin intensity at study enrollment = Medium-intensity statin dose

Product-Limit Survival Estimates						
Prim_Outc_Time		Survival	Failure	Survival Standard Error	Number Failed	Number Left
3.85500	*	.	.	.	67	221
3.85500	*	.	.	.	67	220
3.85500	*	.	.	.	67	219
3.85500	*	.	.	.	67	218
3.85800	*	.	.	.	67	217
3.86000	*	.	.	.	67	216
3.86900	*	.	.	.	67	215
3.87100	*	.	.	.	67	214
3.87400	*	.	.	.	67	213
3.87400	*	.	.	.	67	212
3.87400	*	.	.	.	67	211
3.87400	*	.	.	.	67	210
3.87400	*	.	.	.	67	209
3.87400	*	.	.	.	67	208
3.87400	*	.	.	.	67	207
3.87400	*	.	.	.	67	206
3.88500	*	.	.	.	67	205
3.88800	*	.	.	.	67	204
3.88800	*	.	.	.	67	203
3.88800	*	.	.	.	67	202
3.88800	*	.	.	.	67	201
3.89000	*	.	.	.	67	200
3.89900	*	.	.	.	67	199
3.89900	*	.	.	.	67	198
3.90700	*	.	.	.	67	197
3.90700	*	.	.	.	67	196
3.91000	*	.	.	.	67	195
3.91000	*	.	.	.	67	194
3.91000	*	.	.	.	67	193

phregparms_mianalyze_mi_4099

The LIFETEST Procedure

Stratum 3: Statin intensity at study enrollment = Medium-intensity statin dose

Product-Limit Survival Estimates						
Prim_Outc_Time		Survival	Failure	Survival Standard Error	Number Failed	Number Left
3.91000	*	.	.	.	67	192
3.91000	*	.	.	.	67	191
3.91200	*	.	.	.	67	190
3.91200	*	.	.	.	67	189
3.91500	*	.	.	.	67	188
3.91800	*	.	.	.	67	187
3.92900	*	.	.	.	67	186
3.92900	*	.	.	.	67	185
3.92900	*	.	.	.	67	184
3.93200	*	.	.	.	67	183
3.93200	*	.	.	.	67	182
3.93200	*	.	.	.	67	181
3.93200	*	.	.	.	67	180
3.93200	*	.	.	.	67	179
3.93200	*	.	.	.	67	178
3.93200	*	.	.	.	67	177
3.93700	*	.	.	.	67	176
3.93700	*	.	.	.	67	175
3.94000	*	.	.	.	67	174
3.94800	*	.	.	.	67	173
3.95300	*	.	.	.	67	172
3.98600	*	.	.	.	67	171
3.98600	*	.	.	.	67	170
4.00000	*	.	.	.	67	169
4.00300	*	.	.	.	67	168
4.00300	*	.	.	.	67	167
4.00500	*	.	.	.	67	166
4.00500	*	.	.	.	67	165
4.00800	*	.	.	.	67	164

phregparms_mianalyze_mi_4099

The LIFETEST Procedure

Stratum 3: Statin intensity at study enrollment = Medium-intensity statin dose

Product-Limit Survival Estimates						
Prim_Outc_Time		Survival	Failure	Survival Standard Error	Number Failed	Number Left
4.00800	*	.	.	.	67	163
4.01600	*	.	.	.	67	162
4.01900	*	.	.	.	67	161
4.01900	*	.	.	.	67	160
4.02200	*	.	.	.	67	159
4.02200	*	.	.	.	67	158
4.02200	*	.	.	.	67	157
4.02200	*	.	.	.	67	156
4.02500	*	.	.	.	67	155
4.02700	*	.	.	.	67	154
4.02700	*	.	.	.	67	153
4.03000	*	.	.	.	67	152
4.03000	*	.	.	.	67	151
4.03600	*	.	.	.	67	150
4.03800	*	.	.	.	67	149
4.04400	*	.	.	.	67	148
4.04700	*	.	.	.	67	147
4.04900	*	.	.	.	67	146
4.05500	*	.	.	.	67	145
4.06300	*	.	.	.	67	144
4.06800	*	.	.	.	67	143
4.06800	*	.	.	.	67	142
4.06800	*	.	.	.	67	141
4.07900	*	.	.	.	67	140
4.08200	*	.	.	.	67	139
4.08500	*	.	.	.	67	138
4.08500	*	.	.	.	67	137
4.08500	*	.	.	.	67	136
4.08800	*	.	.	.	67	135

phregparms_mianalyze_mi_4099

The LIFETEST Procedure

Stratum 3: Statin intensity at study enrollment = Medium-intensity statin dose

Product-Limit Survival Estimates						
Prim_Outc_Time		Survival	Failure	Survival Standard Error	Number Failed	Number Left
4.08800	*	.	.	.	67	134
4.09000	*	.	.	.	67	133
4.09300	*	.	.	.	67	132
4.10100	*	.	.	.	67	131
4.10100	*	.	.	.	67	130
4.10100	*	.	.	.	67	129
4.10100	*	.	.	.	67	128
4.10700		0.9317	0.0683	0.0119	68	127
4.10700	*	.	.	.	68	126
4.10700	*	.	.	.	68	125
4.11000	*	.	.	.	68	124
4.11200	*	.	.	.	68	123
4.11500	*	.	.	.	68	122
4.12000	*	.	.	.	68	121
4.12000	*	.	.	.	68	120
4.12000	*	.	.	.	68	119
4.12000	*	.	.	.	68	118
4.12000	*	.	.	.	68	117
4.12000	*	.	.	.	68	116
4.12300	*	.	.	.	68	115
4.12600	*	.	.	.	68	114
4.12600	*	.	.	.	68	113
4.13400	*	.	.	.	68	112
4.13700	*	.	.	.	68	111
4.13700	*	.	.	.	68	110
4.13700	*	.	.	.	68	109
4.13700	*	.	.	.	68	108
4.14000	*	.	.	.	68	107
4.14000	*	.	.	.	68	106

phregparms_mianalyze_mi_4099

The LIFETEST Procedure

Stratum 3: Statin intensity at study enrollment = Medium-intensity statin dose

Product-Limit Survival Estimates						
Prim_Outc_Time		Survival	Failure	Survival Standard Error	Number Failed	Number Left
4.14000	*	.	.	.	68	105
4.14000	*	.	.	.	68	104
4.14000	*	.	.	.	68	103
4.14500	*	.	.	.	68	102
4.14500	*	.	.	.	68	101
4.14500	*	.	.	.	68	100
4.14500	*	.	.	.	68	99
4.14800	*	.	.	.	68	98
4.15300	*	.	.	.	68	97
4.15300	*	.	.	.	68	96
4.15300	*	.	.	.	68	95
4.15300	*	.	.	.	68	94
4.15300	*	.	.	.	68	93
4.15300	*	.	.	.	68	92
4.15300	*	.	.	.	68	91
4.15900	*	.	.	.	68	90
4.15900	*	.	.	.	68	89
4.15900	*	.	.	.	68	88
4.15900	*	.	.	.	68	87
4.15900	*	.	.	.	68	86
4.15900	*	.	.	.	68	85
4.15900	*	.	.	.	68	84
4.15900	*	.	.	.	68	83
4.15900	*	.	.	.	68	82
4.16200	*	.	.	.	68	81
4.16400	*	.	.	.	68	80
4.16400	*	.	.	.	68	79
4.16400	*	.	.	.	68	78
4.17200	*	.	.	.	68	77

phregparms_mianalyze_mi_4099

The LIFETEST Procedure

Stratum 3: Statin intensity at study enrollment = Medium-intensity statin dose

Product-Limit Survival Estimates						
Prim_Outc_Time		Survival	Failure	Survival Standard Error	Number Failed	Number Left
4.17500	*	.	.	.	68	76
4.17500	*	.	.	.	68	75
4.17800	*	.	.	.	68	74
4.17800	*	.	.	.	68	73
4.18300	*	.	.	.	68	72
4.18300	*	.	.	.	68	71
4.19200	*	.	.	.	68	70
4.19400	*	.	.	.	68	69
4.19400	*	.	.	.	68	68
4.19400	*	.	.	.	68	67
4.19400	*	.	.	.	68	66
4.19400	*	.	.	.	68	65
4.21900	*	.	.	.	68	64
4.22200	*	.	.	.	68	63
4.22200	*	.	.	.	68	62
4.22200	*	.	.	.	68	61
4.22500	*	.	.	.	68	60
4.22700	*	.	.	.	68	59
4.23300	*	.	.	.	68	58
4.23500	*	.	.	.	68	57
4.23500	*	.	.	.	68	56
4.24600	*	.	.	.	68	55
4.24600	*	.	.	.	68	54
4.24600	*	.	.	.	68	53
4.25200	*	.	.	.	68	52
4.25500	*	.	.	.	68	51
4.25700	*	.	.	.	68	50
4.26000	*	.	.	.	68	49
4.26300	*	.	.	.	68	48

phregparms_mianalyze_mi_4099

The LIFETEST Procedure

Stratum 3: Statin intensity at study enrollment = Medium-intensity statin dose

Product-Limit Survival Estimates						
Prim_Outc_Time		Survival	Failure	Survival Standard Error	Number Failed	Number Left
4.26300	*	.	.	.	68	47
4.26300	*	.	.	.	68	46
4.26300	*	.	.	.	68	45
4.26600	*	.	.	.	68	44
4.26800	*	.	.	.	68	43
4.26800	*	.	.	.	68	42
4.26800	*	.	.	.	68	41
4.26800	*	.	.	.	68	40
4.26800	*	.	.	.	68	39
4.26800	*	.	.	.	68	38
4.27400	*	.	.	.	68	37
4.27700	*	.	.	.	68	36
4.27700	*	.	.	.	68	35
4.29000	*	.	.	.	68	34
4.29600	*	.	.	.	68	33
4.29600	*	.	.	.	68	32
4.29600	*	.	.	.	68	31
4.30100	*	.	.	.	68	30
4.30100	*	.	.	.	68	29
4.30900	*	.	.	.	68	28
4.30900	*	.	.	.	68	27
4.31200	*	.	.	.	68	26
4.31500	*	.	.	.	68	25
4.31500	*	.	.	.	68	24
4.31500	*	.	.	.	68	23
4.31800	*	.	.	.	68	22
4.32000	*	.	.	.	68	21
4.32000	*	.	.	.	68	20
4.32000	*	.	.	.	68	19

phregparms_mianalyze_mi_4099

The LIFETEST Procedure

Stratum 3: Statin intensity at study enrollment = Medium-intensity statin dose

Product-Limit Survival Estimates						
Prim_Outc_Time		Survival	Failure	Survival Standard Error	Number Failed	Number Left
4.35000	*	.	.	.	68	18
4.35000	*	.	.	.	68	17
4.35000	*	.	.	.	68	16
4.35600	*	.	.	.	68	15
4.35600	*	.	.	.	68	14
4.35600	*	.	.	.	68	13
4.36400	*	.	.	.	68	12
4.36400	*	.	.	.	68	11
4.36400	*	.	.	.	68	10
4.36400	*	.	.	.	68	9
4.36700	*	.	.	.	68	8
4.37500	*	.	.	.	68	7
4.38300	*	.	.	.	68	6
4.38300	*	.	.	.	68	5
4.49600	*	.	.	.	68	4
4.52600	*	.	.	.	68	3
4.70900	*	.	.	.	68	2
4.77500	*	.	.	.	68	1
4.77800	*	.	.	.	68	0

Note: The marked survival times are censored observations.

phregparms_mianalyze_mi_4099

The LIFETEST Procedure

Stratum 3: Statin intensity at study enrollment = Medium-intensity statin dose

Summary Statistics for Time Variable Prim_Outc_Time

Quartile Estimates				
	Point	95% Confidence Interval		
Percent	Estimate	Transform	[Lower	Upper)
75	.	LOGLOG	.	.
50	.	LOGLOG	.	.
25	.	LOGLOG	.	.

Mean	Standard Error
3.98488	0.01484

Note: The mean survival time and its standard error were underestimated because the largest observation was censored and the estimation was restricted to the largest event time.

phregparms_mianalyze_mi_4099

The LIFETEST Procedure

Stratum 4: Statin intensity at study enrollment = No use statin dose

Product-Limit Survival Estimates						
Prim_Outc_Time		Survival	Failure	Survival Standard Error	Number Failed	Number Left
0.00000		1.0000	0	0	0	1166
0.00300	*	.	.	.	0	1165
0.01400	*	.	.	.	0	1164
0.01400	*	.	.	.	0	1163
0.01900	*	.	.	.	0	1162
0.01900	*	.	.	.	0	1161
0.04400	*	.	.	.	0	1160
0.05700	*	.	.	.	0	1159
0.05700	*	.	.	.	0	1158
0.06000		0.9991	0.000864	0.000863	1	1157
0.07400	*	.	.	.	1	1156
0.07700	*	.	.	.	1	1155
0.07900	*	.	.	.	1	1154
0.08500	*	.	.	.	1	1153
0.08500	*	.	.	.	1	1152
0.08500	*	.	.	.	1	1151
0.09000	*	.	.	.	1	1150
0.09000	*	.	.	.	1	1149
0.10100	*	.	.	.	1	1148
0.10400	*	.	.	.	1	1147
0.11200	*	.	.	.	1	1146
0.12900	*	.	.	.	1	1145
0.15300	*	.	.	.	1	1144
0.16200	*	.	.	.	1	1143
0.17200	*	.	.	.	1	1142
0.18100	*	.	.	.	1	1141
0.19200	*	.	.	.	1	1140
0.19700	*	.	.	.	1	1139
0.20500	*	.	.	.	1	1138

phregparms_mianalyze_mi_4099

The LIFETEST Procedure

Stratum 4: Statin intensity at study enrollment = No use statin dose

Product-Limit Survival Estimates						
Prim_Outc_Time		Survival	Failure	Survival Standard Error	Number Failed	Number Left
0.20800	*	.	.	.	1	1137
0.20800	*	.	.	.	1	1136
0.21100	*	.	.	.	1	1135
0.21900	*	.	.	.	1	1134
0.23000	*	.	.	.	1	1133
0.23000	*	.	.	.	1	1132
0.23000	*	.	.	.	1	1131
0.23000	*	.	.	.	1	1130
0.23300		0.9983	0.00175	0.00123	2	1129
0.23300	*	.	.	.	2	1128
0.23800	*	.	.	.	2	1127
0.24600	*	.	.	.	2	1126
0.24900	*	.	.	.	2	1125
0.24900	*	.	.	.	2	1124
0.24900	*	.	.	.	2	1123
0.25200	*	.	.	.	2	1122
0.25200	*	.	.	.	2	1121
0.25200	*	.	.	.	2	1120
0.25200	*	.	.	.	2	1119
0.25200	*	.	.	.	2	1118
0.25500	*	.	.	.	2	1117
0.26800		0.9974	0.00264	0.00152	3	1116
0.27100	*	.	.	.	3	1115
0.27700	*	.	.	.	3	1114
0.27700	*	.	.	.	3	1113
0.28200	*	.	.	.	3	1112
0.28200	*	.	.	.	3	1111
0.28500	*	.	.	.	3	1110
0.28700	*	.	.	.	3	1109

phregparms_mianalyze_mi_4099

The LIFETEST Procedure

Stratum 4: Statin intensity at study enrollment = No use statin dose

Product-Limit Survival Estimates						
Prim_Outc_Time		Survival	Failure	Survival Standard Error	Number Failed	Number Left
0.28700	*	.	.	.	3	1108
0.28700	*	.	.	.	3	1107
0.28700	*	.	.	.	3	1106
0.29300	*	.	.	.	3	1105
0.29600	*	.	.	.	3	1104
0.29800	*	.	.	.	3	1103
0.29800	*	.	.	.	3	1102
0.30700		0.9965	0.00355	0.00177	4	1101
0.30700	*	.	.	.	4	1100
0.31200	*	.	.	.	4	1099
0.31200	*	.	.	.	4	1098
0.31800	*	.	.	.	4	1097
0.32300	*	.	.	.	4	1096
0.33700	*	.	.	.	4	1095
0.33900		0.9955	0.00446	0.00199	5	1094
0.37500	*	.	.	.	5	1093
0.38600		0.9946	0.00537	0.00219	6	1092
0.40200	*	.	.	.	6	1091
0.44100	*	.	.	.	6	1090
0.44100	*	.	.	.	6	1089
0.44100	*	.	.	.	6	1088
0.45700	*	.	.	.	6	1087
0.46000	*	.	.	.	6	1086
0.46000	*	.	.	.	6	1085
0.46000	*	.	.	.	6	1084
0.46000	*	.	.	.	6	1083
0.46000	*	.	.	.	6	1082
0.46000	*	.	.	.	6	1081
0.46000	*	.	.	.	6	1080

phregparms_mianalyze_mi_4099

The LIFETEST Procedure

Stratum 4: Statin intensity at study enrollment = No use statin dose

Product-Limit Survival Estimates						
Prim_Outc_Time		Survival	Failure	Survival Standard Error	Number Failed	Number Left
0.46000	*	.	.	.	6	1079
0.46000	*	.	.	.	6	1078
0.46000	*	.	.	.	6	1077
0.46000	*	.	.	.	6	1076
0.46000	*	.	.	.	6	1075
0.46000	*	.	.	.	6	1074
0.46000	*	.	.	.	6	1073
0.46000	*	.	.	.	6	1072
0.46000	*	.	.	.	6	1071
0.46500	*	.	.	.	6	1070
0.46500	*	.	.	.	6	1069
0.47100		0.9937	0.00630	0.00237	7	1068
0.47400		0.9928	0.00723	0.00255	8	1067
0.47400	*	.	.	.	8	1066
0.47900	*	.	.	.	8	1065
0.47900	*	.	.	.	8	1064
0.47900	*	.	.	.	8	1063
0.47900	*	.	.	.	8	1062
0.49600	*	.	.	.	8	1061
0.51200	*	.	.	.	8	1060
0.51500	*	.	.	.	8	1059
0.51500	*	.	.	.	8	1058
0.51700	*	.	.	.	8	1057
0.51700	*	.	.	.	8	1056
0.51700	*	.	.	.	8	1055
0.52000		0.9918	0.00817	0.00271	9	1054
0.52300	*	.	.	.	9	1053
0.52800	*	.	.	.	9	1052
0.53700	*	.	.	.	9	1051

phregparms_mianalyze_mi_4099

The LIFETEST Procedure

Stratum 4: Statin intensity at study enrollment = No use statin dose

Product-Limit Survival Estimates						
Prim_Outc_Time		Survival	Failure	Survival Standard Error	Number Failed	Number Left
0.53700	*	.	.	.	9	1050
0.53700	*	.	.	.	9	1049
0.53700	*	.	.	.	9	1048
0.53700	*	.	.	.	9	1047
0.54200	*	.	.	.	9	1046
0.54200	*	.	.	.	9	1045
0.54500	*	.	.	.	9	1044
0.57500	*	.	.	.	9	1043
0.57800	*	.	.	.	9	1042
0.59400	*	.	.	.	9	1041
0.61300	*	.	.	.	9	1040
0.61300	*	.	.	.	9	1039
0.61300	*	.	.	.	9	1038
0.61300	*	.	.	.	9	1037
0.63000	*	.	.	.	9	1036
0.63800	*	.	.	.	9	1035
0.64100	*	.	.	.	9	1034
0.65200		0.9909	0.00913	0.00287	10	1033
0.65700	*	.	.	.	10	1032
0.67100	*	.	.	.	10	1031
0.68200		0.9899	0.0101	0.00303	11	1030
0.68400	*	.	.	.	11	1029
0.69000	*	.	.	.	11	1028
0.69000	*	.	.	.	11	1027
0.69000	*	.	.	.	11	1026
0.69000	*	.	.	.	11	1025
0.69000	*	.	.	.	11	1024
0.69000	*	.	.	.	11	1023
0.69000	*	.	.	.	11	1022

phregparms_mianalyze_mi_4099

The LIFETEST Procedure

Stratum 4: Statin intensity at study enrollment = No use statin dose

Product-Limit Survival Estimates						
Prim_Outc_Time		Survival	Failure	Survival Standard Error	Number Failed	Number Left
0.69000	*	.	.	.	11	1021
0.69000	*	.	.	.	11	1020
0.69000	*	.	.	.	11	1019
0.69000	*	.	.	.	11	1018
0.69000	*	.	.	.	11	1017
0.69000	*	.	.	.	11	1016
0.69000	*	.	.	.	11	1015
0.69000	*	.	.	.	11	1014
0.70900	*	.	.	.	11	1013
0.70900	*	.	.	.	11	1012
0.72300	*	.	.	.	11	1011
0.72800	*	.	.	.	11	1010
0.74500	*	.	.	.	11	1009
0.74700	*	.	.	.	11	1008
0.74700	*	.	.	.	11	1007
0.75300	*	.	.	.	11	1006
0.75800		0.9889	0.0111	0.00318	12	1005
0.76700	*	.	.	.	12	1004
0.76700	*	.	.	.	12	1003
0.78600	*	.	.	.	12	1002
0.80200		0.9879	0.0121	0.00333	13	1001
0.80500	*	.	.	.	13	1000
0.80500	*	.	.	.	13	999
0.82100	*	.	.	.	13	998
0.82400	*	.	.	.	13	997
0.82400	*	.	.	.	13	996
0.83500	*	.	.	.	13	995
0.84100	*	.	.	.	13	994
0.84300	*	.	.	.	13	993

phregparms_mianalyze_mi_4099

The LIFETEST Procedure

Stratum 4: Statin intensity at study enrollment = No use statin dose

Product-Limit Survival Estimates						
Prim_Outc_Time		Survival	Failure	Survival Standard Error	Number Failed	Number Left
0.84300	*	.	.	.	13	992
0.84300	*	.	.	.	13	991
0.84300	*	.	.	.	13	990
0.84900	*	.	.	.	13	989
0.87100		0.9869	0.0131	0.00347	14	988
0.87600		0.9859	0.0141	0.00361	15	987
0.88200	*	.	.	.	15	986
0.89300	*	.	.	.	15	985
0.89800	*	.	.	.	15	984
0.90100	*	.	.	.	15	983
0.90100	*	.	.	.	15	982
0.90100	*	.	.	.	15	981
0.90100	*	.	.	.	15	980
0.90100	*	.	.	.	15	979
0.90600	*	.	.	.	15	978
0.91200	*	.	.	.	15	977
0.91400	*	.	.	.	15	976
0.92000	*	.	.	.	15	975
0.92000	*	.	.	.	15	974
0.92000	*	.	.	.	15	973
0.92000	*	.	.	.	15	972
0.92000	*	.	.	.	15	971
0.92000	*	.	.	.	15	970
0.92000	*	.	.	.	15	969
0.92000	*	.	.	.	15	968
0.92000	*	.	.	.	15	967
0.92000	*	.	.	.	15	966
0.92000	*	.	.	.	15	965
0.92000	*	.	.	.	15	964

phregparms_mianalyze_mi_4099

The LIFETEST Procedure

Stratum 4: Statin intensity at study enrollment = No use statin dose

Product-Limit Survival Estimates						
Prim_Outc_Time		Survival	Failure	Survival Standard Error	Number Failed	Number Left
0.92000	*	.	.	.	15	963
0.92000	*	.	.	.	15	962
0.92000	*	.	.	.	15	961
0.92000	*	.	.	.	15	960
0.92000	*	.	.	.	15	959
0.92000	*	.	.	.	15	958
0.92000	*	.	.	.	15	957
0.92000	*	.	.	.	15	956
0.92000	*	.	.	.	15	955
0.92000	*	.	.	.	15	954
0.92000	*	.	.	.	15	953
0.92000	*	.	.	.	15	952
0.92000	*	.	.	.	15	951
0.92000	*	.	.	.	15	950
0.92000	*	.	.	.	15	949
0.92000	*	.	.	.	15	948
0.92000	*	.	.	.	15	947
0.92000	*	.	.	.	15	946
0.92000	*	.	.	.	15	945
0.92000	*	.	.	.	15	944
0.92000	*	.	.	.	15	943
0.92000	*	.	.	.	15	942
0.92000	*	.	.	.	15	941
0.92000	*	.	.	.	15	940
0.92000	*	.	.	.	15	939
0.92000	*	.	.	.	15	938
0.92000	*	.	.	.	15	937
0.92000	*	.	.	.	15	936
0.92000	*	.	.	.	15	935

phregparms_mianalyze_mi_4099

The LIFETEST Procedure

Stratum 4: Statin intensity at study enrollment = No use statin dose

Product-Limit Survival Estimates						
Prim_Outc_Time		Survival	Failure	Survival Standard Error	Number Failed	Number Left
0.92000	*	.	.	.	15	934
0.92000	*	.	.	.	15	933
0.92000	*	.	.	.	15	932
0.92000	*	.	.	.	15	931
0.92000	*	.	.	.	15	930
0.92300	*	.	.	.	15	929
0.92500	*	.	.	.	15	928
0.92500	*	.	.	.	15	927
0.93600	*	.	.	.	15	926
0.93600	*	.	.	.	15	925
0.93600	*	.	.	.	15	924
0.93900	*	.	.	.	15	923
0.93900	*	.	.	.	15	922
0.93900	*	.	.	.	15	921
0.93900	*	.	.	.	15	920
0.93900	*	.	.	.	15	919
0.93900	*	.	.	.	15	918
0.93900	*	.	.	.	15	917
0.93900	*	.	.	.	15	916
0.93900	*	.	.	.	15	915
0.93900	*	.	.	.	15	914
0.93900	*	.	.	.	15	913
0.93900	*	.	.	.	15	912
0.94200	*	.	.	.	15	911
0.94500	*	.	.	.	15	910
0.95300	*	.	.	.	15	909
0.95600	*	.	.	.	15	908
0.95800	*	.	.	.	15	907
0.95800	*	.	.	.	15	906

phregparms_mianalyze_mi_4099

The LIFETEST Procedure

Stratum 4: Statin intensity at study enrollment = No use statin dose

Product-Limit Survival Estimates						
Prim_Outc_Time		Survival	Failure	Survival Standard Error	Number Failed	Number Left
0.95800	*	.	.	.	15	905
0.95800	*	.	.	.	15	904
0.95800	*	.	.	.	15	903
0.95800	*	.	.	.	15	902
0.96100	*	.	.	.	15	901
0.96100	*	.	.	.	15	900
0.96100	*	.	.	.	15	899
0.96400		0.9848	0.0152	0.00377	16	898
0.97700	*	.	.	.	16	897
0.97700	*	.	.	.	16	896
0.97700	*	.	.	.	16	895
0.97700	*	.	.	.	16	894
0.99700	*	.	.	.	16	893
0.99700	*	.	.	.	16	892
0.99700	*	.	.	.	16	891
0.99700	*	.	.	.	16	890
0.99700	*	.	.	.	16	889
0.99700	*	.	.	.	16	888
0.99700	*	.	.	.	16	887
0.99700	*	.	.	.	16	886
0.99700	*	.	.	.	16	885
0.99700	*	.	.	.	16	884
0.99700	*	.	.	.	16	883
0.99900	*	.	.	.	16	882
0.99900	*	.	.	.	16	881
1.00500		0.9837	0.0163	0.00393	17	880
1.01600	*	.	.	.	17	879
1.01600	*	.	.	.	17	878
1.01600	*	.	.	.	17	877

phregparms_mianalyze_mi_4099

The LIFETEST Procedure

Stratum 4: Statin intensity at study enrollment = No use statin dose

Product-Limit Survival Estimates						
Prim_Outc_Time		Survival	Failure	Survival Standard Error	Number Failed	Number Left
1.01600	*	.	.	.	17	876
1.01800	*	.	.	.	17	875
1.02100		0.9826	0.0174	0.00408	18	874
1.02100	*	.	.	.	18	873
1.03500	*	.	.	.	18	872
1.03500	*	.	.	.	18	871
1.04900	*	.	.	.	18	870
1.04900	*	.	.	.	18	869
1.04900	*	.	.	.	18	868
1.05100	*	.	.	.	18	867
1.05400	*	.	.	.	18	866
1.05400	*	.	.	.	18	865
1.05400	*	.	.	.	18	864
1.05400	*	.	.	.	18	863
1.05400	*	.	.	.	18	862
1.06200	*	.	.	.	18	861
1.06200	*	.	.	.	18	860
1.06800	*	.	.	.	18	859
1.06800	*	.	.	.	18	858
1.06800	*	.	.	.	18	857
1.07300	*	.	.	.	18	856
1.07300	*	.	.	.	18	855
1.07300	*	.	.	.	18	854
1.07300	*	.	.	.	18	853
1.07300	*	.	.	.	18	852
1.07300	*	.	.	.	18	851
1.07300	*	.	.	.	18	850
1.07300	*	.	.	.	18	849
1.07600	*	.	.	.	18	848

phregparms_mianalyze_mi_4099

The LIFETEST Procedure

Stratum 4: Statin intensity at study enrollment = No use statin dose

Product-Limit Survival Estimates						
Prim_Outc_Time		Survival	Failure	Survival Standard Error	Number Failed	Number Left
1.09200	*	.	.	.	18	847
1.09200	*	.	.	.	18	846
1.09200	*	.	.	.	18	845
1.09200	*	.	.	.	18	844
1.09200	*	.	.	.	18	843
1.09200	*	.	.	.	18	842
1.09500	*	.	.	.	18	841
1.10100	*	.	.	.	18	840
1.11200	*	.	.	.	18	839
1.11400	*	.	.	.	18	838
1.12000	*	.	.	.	18	837
1.12800	*	.	.	.	18	836
1.13100	*	.	.	.	18	835
1.13100	*	.	.	.	18	834
1.13100	*	.	.	.	18	833
1.13100	*	.	.	.	18	832
1.13600	*	.	.	.	18	831
1.14200	*	.	.	.	18	830
1.14400	*	.	.	.	18	829
1.14400	*	.	.	.	18	828
1.14700	*	.	.	.	18	827
1.14700	*	.	.	.	18	826
1.15000	*	.	.	.	18	825
1.15000	*	.	.	.	18	824
1.15000	*	.	.	.	18	823
1.15000	*	.	.	.	18	822
1.15000	*	.	.	.	18	821
1.15000	*	.	.	.	18	820
1.15000	*	.	.	.	18	819

phregparms_mianalyze_mi_4099

The LIFETEST Procedure

Stratum 4: Statin intensity at study enrollment = No use statin dose

Product-Limit Survival Estimates						
Prim_Outc_Time		Survival	Failure	Survival Standard Error	Number Failed	Number Left
1.15000	*	.	.	.	18	818
1.15000	*	.	.	.	18	817
1.15000	*	.	.	.	18	816
1.15000	*	.	.	.	18	815
1.15000	*	.	.	.	18	814
1.15000	*	.	.	.	18	813
1.15000	*	.	.	.	18	812
1.15000	*	.	.	.	18	811
1.15000	*	.	.	.	18	810
1.15000	*	.	.	.	18	809
1.15000	*	.	.	.	18	808
1.15000	*	.	.	.	18	807
1.15000	*	.	.	.	18	806
1.15000	*	.	.	.	18	805
1.15000	*	.	.	.	18	804
1.15000	*	.	.	.	18	803
1.15000	*	.	.	.	18	802
1.15000	*	.	.	.	18	801
1.15000	*	.	.	.	18	800
1.15000	*	.	.	.	18	799
1.15000	*	.	.	.	18	798
1.15000	*	.	.	.	18	797
1.15000	*	.	.	.	18	796
1.15000	*	.	.	.	18	795
1.15000	*	.	.	.	18	794
1.15000	*	.	.	.	18	793
1.15000	*	.	.	.	18	792
1.15000	*	.	.	.	18	791
1.15000	*	.	.	.	18	790

phregparms_mianalyze_mi_4099

The LIFETEST Procedure

Stratum 4: Statin intensity at study enrollment = No use statin dose

Product-Limit Survival Estimates						
Prim_Outc_Time		Survival	Failure	Survival Standard Error	Number Failed	Number Left
1.15000	*	.	.	.	18	789
1.15000	*	.	.	.	18	788
1.15000	*	.	.	.	18	787
1.15000	*	.	.	.	18	786
1.15000	*	.	.	.	18	785
1.15000	*	.	.	.	18	784
1.15000	*	.	.	.	18	783
1.15000	*	.	.	.	18	782
1.15000	*	.	.	.	18	781
1.15000	*	.	.	.	18	780
1.15000	*	.	.	.	18	779
1.15300	*	.	.	.	18	778
1.15500	*	.	.	.	18	777
1.16400	*	.	.	.	18	776
1.16400	*	.	.	.	18	775
1.16600	*	.	.	.	18	774
1.16900	*	.	.	.	18	773
1.16900	*	.	.	.	18	772
1.16900	*	.	.	.	18	771
1.16900	*	.	.	.	18	770
1.16900	*	.	.	.	18	769
1.17500	*	.	.	.	18	768
1.18000	*	.	.	.	18	767
1.18500	*	.	.	.	18	766
1.18500	*	.	.	.	18	765
1.18800	*	.	.	.	18	764
1.18800	*	.	.	.	18	763
1.18800	*	.	.	.	18	762
1.18800	*	.	.	.	18	761

phregparms_mianalyze_mi_4099

The LIFETEST Procedure

Stratum 4: Statin intensity at study enrollment = No use statin dose

Product-Limit Survival Estimates						
Prim_Outc_Time		Survival	Failure	Survival Standard Error	Number Failed	Number Left
1.18800	*	.	.	.	18	760
1.18800	*	.	.	.	18	759
1.18800	*	.	.	.	18	758
1.18800	*	.	.	.	18	757
1.18800	*	.	.	.	18	756
1.18800	*	.	.	.	18	755
1.18800	*	.	.	.	18	754
1.18800	*	.	.	.	18	753
1.18800	*	.	.	.	18	752
1.18800	*	.	.	.	18	751
1.18800	*	.	.	.	18	750
1.19100	*	.	.	.	18	749
1.19100	*	.	.	.	18	748
1.19600	*	.	.	.	18	747
1.19900	*	.	.	.	18	746
1.20200	*	.	.	.	18	745
1.20200	*	.	.	.	18	744
1.20200	*	.	.	.	18	743
1.20700	*	.	.	.	18	742
1.20700	*	.	.	.	18	741
1.20700	*	.	.	.	18	740
1.21000	*	.	.	.	18	739
1.21600	*	.	.	.	18	738
1.21800	*	.	.	.	18	737
1.22100	*	.	.	.	18	736
1.22400	*	.	.	.	18	735
1.22700	*	.	.	.	18	734
1.22700	*	.	.	.	18	733
1.22700	*	.	.	.	18	732

phregparms_mianalyze_mi_4099

The LIFETEST Procedure

Stratum 4: Statin intensity at study enrollment = No use statin dose

Product-Limit Survival Estimates						
Prim_Outc_Time		Survival	Failure	Survival Standard Error	Number Failed	Number Left
1.22700	*	.	.	.	18	731
1.22700	*	.	.	.	18	730
1.22700	*	.	.	.	18	729
1.22700	*	.	.	.	18	728
1.22900	*	.	.	.	18	727
1.22900	*	.	.	.	18	726
1.23200	*	.	.	.	18	725
1.24000	*	.	.	.	18	724
1.24600	*	.	.	.	18	723
1.24600	*	.	.	.	18	722
1.24600	*	.	.	.	18	721
1.24600	*	.	.	.	18	720
1.24800	*	.	.	.	18	719
1.25100	*	.	.	.	18	718
1.25100	*	.	.	.	18	717
1.25700	*	.	.	.	18	716
1.26500	*	.	.	.	18	715
1.26500	*	.	.	.	18	714
1.26500	*	.	.	.	18	713
1.26500	*	.	.	.	18	712
1.27600	*	.	.	.	18	711
1.28400	*	.	.	.	18	710
1.28700	*	.	.	.	18	709
1.29500	*	.	.	.	18	708
1.29800	*	.	.	.	18	707
1.30000	*	.	.	.	18	706
1.30300	*	.	.	.	18	705
1.30300	*	.	.	.	18	704
1.30300	*	.	.	.	18	703

phregparms_mianalyze_mi_4099

The LIFETEST Procedure

Stratum 4: Statin intensity at study enrollment = No use statin dose

Product-Limit Survival Estimates						
Prim_Outc_Time		Survival	Failure	Survival Standard Error	Number Failed	Number Left
1.30900	*	.	.	.	18	702
1.32000	*	.	.	.	18	701
1.32200	*	.	.	.	18	700
1.32200	*	.	.	.	18	699
1.33600	*	.	.	.	18	698
1.34700		0.9812	0.0188	0.00431	19	697
1.34700	*	.	.	.	19	696
1.34700	*	.	.	.	19	695
1.36100		0.9798	0.0202	0.00453	20	694
1.36100	*	.	.	.	20	693
1.36100	*	.	.	.	20	692
1.36600	*	.	.	.	20	691
1.38000	*	.	.	.	20	690
1.38000	*	.	.	.	20	689
1.38000	*	.	.	.	20	688
1.38000	*	.	.	.	20	687
1.38000	*	.	.	.	20	686
1.38000	*	.	.	.	20	685
1.38000	*	.	.	.	20	684
1.38500	*	.	.	.	20	683
1.39400	*	.	.	.	20	682
1.39900	*	.	.	.	20	681
1.39900	*	.	.	.	20	680
1.40500	*	.	.	.	20	679
1.41000	*	.	.	.	20	678
1.41300	*	.	.	.	20	677
1.41300	*	.	.	.	20	676
1.41800	*	.	.	.	20	675
1.41800	*	.	.	.	20	674

phregparms_mianalyze_mi_4099

The LIFETEST Procedure

Stratum 4: Statin intensity at study enrollment = No use statin dose

Product-Limit Survival Estimates						
Prim_Outc_Time		Survival	Failure	Survival Standard Error	Number Failed	Number Left
1.42100	*	.	.	.	20	673
1.43200	*	.	.	.	20	672
1.43700	*	.	.	.	20	671
1.44300		0.9783	0.0217	0.00475	21	670
1.45700		0.9769	0.0231	0.00496	22	669
1.45700	*	.	.	.	22	668
1.47600		0.9754	0.0246	0.00517	23	667
1.47600	*	.	.	.	23	666
1.49500	*	.	.	.	23	665
1.51100		0.9739	0.0261	0.00536	24	664
1.53300	*	.	.	.	24	663
1.53900	*	.	.	.	24	662
1.54400	*	.	.	.	24	661
1.54700	*	.	.	.	24	660
1.55000	*	.	.	.	24	659
1.55200	*	.	.	.	24	658
1.56100	*	.	.	.	24	657
1.56600	*	.	.	.	24	656
1.57200	*	.	.	.	24	655
1.58800	*	.	.	.	24	654
1.59100	*	.	.	.	24	653
1.60400	*	.	.	.	24	652
1.61000	*	.	.	.	24	651
1.61000	*	.	.	.	24	650
1.61000	*	.	.	.	24	649
1.61000	*	.	.	.	24	648
1.61000	*	.	.	.	24	647
1.61000	*	.	.	.	24	646
1.61000	*	.	.	.	24	645

phregparms_mianalyze_mi_4099

The LIFETEST Procedure

Stratum 4: Statin intensity at study enrollment = No use statin dose

Product-Limit Survival Estimates						
Prim_Outc_Time		Survival	Failure	Survival Standard Error	Number Failed	Number Left
1.61000	*	.	.	.	24	644
1.61000	*	.	.	.	24	643
1.61000	*	.	.	.	24	642
1.61500	*	.	.	.	24	641
1.61500	*	.	.	.	24	640
1.61500	*	.	.	.	24	639
1.61500	*	.	.	.	24	638
1.62100	*	.	.	.	24	637
1.62100	*	.	.	.	24	636
1.62100	*	.	.	.	24	635
1.62600	*	.	.	.	24	634
1.62600	*	.	.	.	24	633
1.63200	*	.	.	.	24	632
1.63200	*	.	.	.	24	631
1.63400	*	.	.	.	24	630
1.64800	*	.	.	.	24	629
1.64800	*	.	.	.	24	628
1.65600	*	.	.	.	24	627
1.66200	*	.	.	.	24	626
1.67000	*	.	.	.	24	625
1.67000	*	.	.	.	24	624
1.67000	*	.	.	.	24	623
1.67600	*	.	.	.	24	622
1.67800	*	.	.	.	24	621
1.68700	*	.	.	.	24	620
1.68700	*	.	.	.	24	619
1.69200	*	.	.	.	24	618
1.70000		0.9724	0.0276	0.00558	25	617
1.71100	*	.	.	.	25	616

phregparms_mianalyze_mi_4099

The LIFETEST Procedure

Stratum 4: Statin intensity at study enrollment = No use statin dose

Product-Limit Survival Estimates						
Prim_Outc_Time		Survival	Failure	Survival Standard Error	Number Failed	Number Left
1.71400	*	.	.	.	25	615
1.71400	*	.	.	.	25	614
1.71400	*	.	.	.	25	613
1.71900	*	.	.	.	25	612
1.71900	*	.	.	.	25	611
1.72500	*	.	.	.	25	610
1.72500	*	.	.	.	25	609
1.72500	*	.	.	.	25	608
1.72500	*	.	.	.	25	607
1.73300	*	.	.	.	25	606
1.73900	*	.	.	.	25	605
1.74700	*	.	.	.	25	604
1.74700	*	.	.	.	25	603
1.75200	*	.	.	.	25	602
1.76000	*	.	.	.	25	601
1.76000	*	.	.	.	25	600
1.76300		0.9707	0.0293	0.00580	26	599
1.76300	*	.	.	.	26	598
1.76300	*	.	.	.	26	597
1.76300	*	.	.	.	26	596
1.76300	*	.	.	.	26	595
1.76600	*	.	.	.	26	594
1.76600	*	.	.	.	26	593
1.76900	*	.	.	.	26	592
1.77100	*	.	.	.	26	591
1.78000	*	.	.	.	26	590
1.78200	*	.	.	.	26	589
1.78200	*	.	.	.	26	588
1.78200	*	.	.	.	26	587

phregparms_mianalyze_mi_4099

The LIFETEST Procedure

Stratum 4: Statin intensity at study enrollment = No use statin dose

Product-Limit Survival Estimates						
Prim_Outc_Time		Survival	Failure	Survival Standard Error	Number Failed	Number Left
1.80200	*	.	.	.	26	586
1.80700	*	.	.	.	26	585
1.81200	*	.	.	.	26	584
1.81500	*	.	.	.	26	583
1.82100	*	.	.	.	26	582
1.82300	*	.	.	.	26	581
1.82300	*	.	.	.	26	580
1.82300	*	.	.	.	26	579
1.82900	*	.	.	.	26	578
1.82900	*	.	.	.	26	577
1.83200	*	.	.	.	26	576
1.83200	*	.	.	.	26	575
1.83200	*	.	.	.	26	574
1.84000	*	.	.	.	26	573
1.84000	*	.	.	.	26	572
1.84000	*	.	.	.	26	571
1.84000	*	.	.	.	26	570
1.84000	*	.	.	.	26	569
1.84300	*	.	.	.	26	568
1.84500		0.9690	0.0310	0.00604	27	567
1.84500	*	.	.	.	27	566
1.84500	*	.	.	.	27	565
1.85100	*	.	.	.	27	564
1.85100	*	.	.	.	27	563
1.85400	*	.	.	.	27	562
1.85900	*	.	.	.	27	561
1.85900	*	.	.	.	27	560
1.86400	*	.	.	.	27	559
1.86400	*	.	.	.	27	558

phregparms_mianalyze_mi_4099

The LIFETEST Procedure

Stratum 4: Statin intensity at study enrollment = No use statin dose

Product-Limit Survival Estimates						
Prim_Outc_Time		Survival	Failure	Survival Standard Error	Number Failed	Number Left
1.86400	*	.	.	.	27	557
1.86700	*	.	.	.	27	556
1.86700	*	.	.	.	27	555
1.86700	*	.	.	.	27	554
1.87000	*	.	.	.	27	553
1.87000	*	.	.	.	27	552
1.87000	*	.	.	.	27	551
1.87000	*	.	.	.	27	550
1.87300	*	.	.	.	27	549
1.87500	*	.	.	.	27	548
1.87500	*	.	.	.	27	547
1.87800	*	.	.	.	27	546
1.88400	*	.	.	.	27	545
1.88400	*	.	.	.	27	544
1.88400	*	.	.	.	27	543
1.88600	*	.	.	.	27	542
1.89200	*	.	.	.	27	541
1.90000	*	.	.	.	27	540
1.90000	*	.	.	.	27	539
1.90300	*	.	.	.	27	538
1.90800	*	.	.	.	27	537
1.91100	*	.	.	.	27	536
1.91600	*	.	.	.	27	535
1.91900	*	.	.	.	27	534
1.92200	*	.	.	.	27	533
1.92200	*	.	.	.	27	532
1.93300	*	.	.	.	27	531
1.93800	*	.	.	.	27	530
1.94400	*	.	.	.	27	529

phregparms_mianalyze_mi_4099

The LIFETEST Procedure

Stratum 4: Statin intensity at study enrollment = No use statin dose

Product-Limit Survival Estimates						
Prim_Outc_Time		Survival	Failure	Survival Standard Error	Number Failed	Number Left
1.94900	*	.	.	.	27	528
1.95500	*	.	.	.	27	527
1.96300	*	.	.	.	27	526
1.96300	*	.	.	.	27	525
1.96600	*	.	.	.	27	524
1.96600	*	.	.	.	27	523
1.97100	*	.	.	.	27	522
1.97700	*	.	.	.	27	521
1.97700	*	.	.	.	27	520
1.97900	*	.	.	.	27	519
1.98500	*	.	.	.	27	518
1.98800	*	.	.	.	27	517
1.99300	*	.	.	.	27	516
2.00100	*	.	.	.	27	515
2.00100	*	.	.	.	27	514
2.00400	*	.	.	.	27	513
2.00700		0.9671	0.0329	0.00632	28	512
2.01200	*	.	.	.	28	511
2.01200	*	.	.	.	28	510
2.01500	*	.	.	.	28	509
2.02600	*	.	.	.	28	508
2.03100	*	.	.	.	28	507
2.04000	*	.	.	.	28	506
2.04000	*	.	.	.	28	505
2.05100	*	.	.	.	28	504
2.05600	*	.	.	.	28	503
2.06200	*	.	.	.	28	502
2.06200	*	.	.	.	28	501
2.06700	*	.	.	.	28	500

phregparms_mianalyze_mi_4099

The LIFETEST Procedure

Stratum 4: Statin intensity at study enrollment = No use statin dose

Product-Limit Survival Estimates						
Prim_Outc_Time		Survival	Failure	Survival Standard Error	Number Failed	Number Left
2.07000	*	.	.	.	28	499
2.07000	*	.	.	.	28	498
2.08900	*	.	.	.	28	497
2.08900	*	.	.	.	28	496
2.10800	*	.	.	.	28	495
2.12700	*	.	.	.	28	494
2.14100	*	.	.	.	28	493
2.16600	*	.	.	.	28	492
2.17100	*	.	.	.	28	491
2.20700	*	.	.	.	28	490
2.22900	*	.	.	.	28	489
2.23700	*	.	.	.	28	488
2.24200	*	.	.	.	28	487
2.24200	*	.	.	.	28	486
2.25600	*	.	.	.	28	485
2.27500	*	.	.	.	28	484
2.30000	*	.	.	.	28	483
2.31900		0.9651	0.0349	0.00661	29	482
2.32700	*	.	.	.	29	481
2.34600	*	.	.	.	29	480
2.35700	*	.	.	.	29	479
2.35700	*	.	.	.	29	478
2.35700	*	.	.	.	29	477
2.35700	*	.	.	.	29	476
2.37100	*	.	.	.	29	475
2.37100	*	.	.	.	29	474
2.37100	*	.	.	.	29	473
2.37900	*	.	.	.	29	472
2.40400	*	.	.	.	29	471

phregparms_mianalyze_mi_4099

The LIFETEST Procedure

Stratum 4: Statin intensity at study enrollment = No use statin dose

Product-Limit Survival Estimates						
Prim_Outc_Time		Survival	Failure	Survival Standard Error	Number Failed	Number Left
2.40400	*	.	.	.	29	470
2.41500	*	.	.	.	29	469
2.41500	*	.	.	.	29	468
2.43400	*	.	.	.	29	467
2.43400	*	.	.	.	29	466
2.45600	*	.	.	.	29	465
2.48000	*	.	.	.	29	464
2.48600	*	.	.	.	29	463
2.49100	*	.	.	.	29	462
2.49700		0.9630	0.0370	0.00692	30	461
2.49700	*	.	.	.	30	460
2.50500	*	.	.	.	30	459
2.51100	*	.	.	.	30	458
2.51100	*	.	.	.	30	457
2.51100	*	.	.	.	30	456
2.51300	*	.	.	.	30	455
2.51600	*	.	.	.	30	454
2.51600	*	.	.	.	30	453
2.51600	*	.	.	.	30	452
2.53000	*	.	.	.	30	451
2.53000	*	.	.	.	30	450
2.53000	*	.	.	.	30	449
2.53000	*	.	.	.	30	448
2.53000	*	.	.	.	30	447
2.53000	*	.	.	.	30	446
2.53000	*	.	.	.	30	445
2.53000	*	.	.	.	30	444
2.53500	*	.	.	.	30	443
2.53800	*	.	.	.	30	442

phregparms_mianalyze_mi_4099

The LIFETEST Procedure

Stratum 4: Statin intensity at study enrollment = No use statin dose

Product-Limit Survival Estimates						
Prim_Outc_Time		Survival	Failure	Survival Standard Error	Number Failed	Number Left
2.54100	*	.	.	.	30	441
2.54100	*	.	.	.	30	440
2.54300	*	.	.	.	30	439
2.54600	*	.	.	.	30	438
2.54900	*	.	.	.	30	437
2.54900	*	.	.	.	30	436
2.54900	*	.	.	.	30	435
2.54900	*	.	.	.	30	434
2.55400	*	.	.	.	30	433
2.55400	*	.	.	.	30	432
2.56000	*	.	.	.	30	431
2.56000	*	.	.	.	30	430
2.56300	*	.	.	.	30	429
2.56500	*	.	.	.	30	428
2.56500	*	.	.	.	30	427
2.56800	*	.	.	.	30	426
2.56800	*	.	.	.	30	425
2.56800	*	.	.	.	30	424
2.56800	*	.	.	.	30	423
2.57100	*	.	.	.	30	422
2.58200	*	.	.	.	30	421
2.58200	*	.	.	.	30	420
2.58700	*	.	.	.	30	419
2.58700	*	.	.	.	30	418
2.58700	*	.	.	.	30	417
2.58700	*	.	.	.	30	416
2.58700	*	.	.	.	30	415
2.60100	*	.	.	.	30	414
2.60100	*	.	.	.	30	413

phregparms_mianalyze_mi_4099

The LIFETEST Procedure

Stratum 4: Statin intensity at study enrollment = No use statin dose

Product-Limit Survival Estimates						
Prim_Outc_Time		Survival	Failure	Survival Standard Error	Number Failed	Number Left
2.60600	*	.	.	.	30	412
2.60600	*	.	.	.	30	411
2.60600	*	.	.	.	30	410
2.60600	*	.	.	.	30	409
2.61700	*	.	.	.	30	408
2.62600	*	.	.	.	30	407
2.62600	*	.	.	.	30	406
2.62600	*	.	.	.	30	405
2.62600	*	.	.	.	30	404
2.62600	*	.	.	.	30	403
2.64200	*	.	.	.	30	402
2.64500	*	.	.	.	30	401
2.64500	*	.	.	.	30	400
2.64500	*	.	.	.	30	399
2.65600	*	.	.	.	30	398
2.65800	*	.	.	.	30	397
2.66400	*	.	.	.	30	396
2.68300	*	.	.	.	30	395
2.68300	*	.	.	.	30	394
2.68300	*	.	.	.	30	393
2.68300	*	.	.	.	30	392
2.68900	*	.	.	.	30	391
2.69100	*	.	.	.	30	390
2.69700	*	.	.	.	30	389
2.70000	*	.	.	.	30	388
2.70000	*	.	.	.	30	387
2.70000	*	.	.	.	30	386
2.70200	*	.	.	.	30	385
2.70200	*	.	.	.	30	384

phregparms_mianalyze_mi_4099

The LIFETEST Procedure

Stratum 4: Statin intensity at study enrollment = No use statin dose

Product-Limit Survival Estimates						
Prim_Outc_Time		Survival	Failure	Survival Standard Error	Number Failed	Number Left
2.70500	*	.	.	.	30	383
2.70800	*	.	.	.	30	382
2.71000	*	.	.	.	30	381
2.71600	*	.	.	.	30	380
2.71900	*	.	.	.	30	379
2.72400	*	.	.	.	30	378
2.73000	*	.	.	.	30	377
2.73800	*	.	.	.	30	376
2.74100	*	.	.	.	30	375
2.74100	*	.	.	.	30	374
2.74100	*	.	.	.	30	373
2.74100	*	.	.	.	30	372
2.74300	*	.	.	.	30	371
2.74600	*	.	.	.	30	370
2.75200	*	.	.	.	30	369
2.75400	*	.	.	.	30	368
2.76000	*	.	.	.	30	367
2.76000	*	.	.	.	30	366
2.76000	*	.	.	.	30	365
2.76000	*	.	.	.	30	364
2.76000	*	.	.	.	30	363
2.76000	*	.	.	.	30	362
2.76000	*	.	.	.	30	361
2.76200	*	.	.	.	30	360
2.76800	*	.	.	.	30	359
2.77300	*	.	.	.	30	358
2.77300	*	.	.	.	30	357
2.77900	*	.	.	.	30	356
2.77900	*	.	.	.	30	355

phregparms_mianalyze_mi_4099

The LIFETEST Procedure

Stratum 4: Statin intensity at study enrollment = No use statin dose

Product-Limit Survival Estimates						
Prim_Outc_Time		Survival	Failure	Survival Standard Error	Number Failed	Number Left
2.77900	*	.	.	.	30	354
2.77900	*	.	.	.	30	353
2.77900	*	.	.	.	30	352
2.77900	*	.	.	.	30	351
2.77900	*	.	.	.	30	350
2.77900	*	.	.	.	30	349
2.77900	*	.	.	.	30	348
2.78400	*	.	.	.	30	347
2.79000	*	.	.	.	30	346
2.79800	*	.	.	.	30	345
2.79800	*	.	.	.	30	344
2.79800	*	.	.	.	30	343
2.79800	*	.	.	.	30	342
2.80400	*	.	.	.	30	341
2.81200	*	.	.	.	30	340
2.81200	*	.	.	.	30	339
2.81500	*	.	.	.	30	338
2.81500	*	.	.	.	30	337
2.81700	*	.	.	.	30	336
2.81700	*	.	.	.	30	335
2.81700	*	.	.	.	30	334
2.82000	*	.	.	.	30	333
2.83600	*	.	.	.	30	332
2.83600	*	.	.	.	30	331
2.83600	*	.	.	.	30	330
2.83900	*	.	.	.	30	329
2.83900	*	.	.	.	30	328
2.85600	*	.	.	.	30	327
2.85600	*	.	.	.	30	326

phregparms_mianalyze_mi_4099

The LIFETEST Procedure

Stratum 4: Statin intensity at study enrollment = No use statin dose

Product-Limit Survival Estimates						
Prim_Outc_Time		Survival	Failure	Survival Standard Error	Number Failed	Number Left
2.85600	*	.	.	.	30	325
2.85600	*	.	.	.	30	324
2.86100	*	.	.	.	30	323
2.86100	*	.	.	.	30	322
2.86700	*	.	.	.	30	321
2.87500	*	.	.	.	30	320
2.88000	*	.	.	.	30	319
2.89100	*	.	.	.	30	318
2.91300	*	.	.	.	30	317
2.91300	*	.	.	.	30	316
2.91300	*	.	.	.	30	315
2.91300	*	.	.	.	30	314
2.92700	*	.	.	.	30	313
2.93000	*	.	.	.	30	312
2.93200	*	.	.	.	30	311
2.93200	*	.	.	.	30	310
2.93200	*	.	.	.	30	309
2.93500	*	.	.	.	30	308
2.94300	*	.	.	.	30	307
2.94900	*	.	.	.	30	306
2.96000	*	.	.	.	30	305
2.96200	*	.	.	.	30	304
2.96500		0.9599	0.0401	0.00759	31	303
2.97100	*	.	.	.	31	302
2.97100	*	.	.	.	31	301
2.97100	*	.	.	.	31	300
2.97100	*	.	.	.	31	299
2.97300	*	.	.	.	31	298
2.97300	*	.	.	.	31	297

phregparms_mianalyze_mi_4099

The LIFETEST Procedure

Stratum 4: Statin intensity at study enrollment = No use statin dose

Product-Limit Survival Estimates						
Prim_Outc_Time		Survival	Failure	Survival Standard Error	Number Failed	Number Left
2.98400	*	.	.	.	31	296
2.98400	*	.	.	.	31	295
2.98400	*	.	.	.	31	294
2.98700	*	.	.	.	31	293
2.99000	*	.	.	.	31	292
2.99000	*	.	.	.	31	291
2.99000	*	.	.	.	31	290
2.99000	*	.	.	.	31	289
3.00100	*	.	.	.	31	288
3.00100	*	.	.	.	31	287
3.00100	*	.	.	.	31	286
3.00100	*	.	.	.	31	285
3.00100	*	.	.	.	31	284
3.00100	*	.	.	.	31	283
3.00100	*	.	.	.	31	282
3.00100	*	.	.	.	31	281
3.00100	*	.	.	.	31	280
3.00100	*	.	.	.	31	279
3.00100	*	.	.	.	31	278
3.00100	*	.	.	.	31	277
3.00100	*	.	.	.	31	276
3.00100	*	.	.	.	31	275
3.00100	*	.	.	.	31	274
3.00100	*	.	.	.	31	273
3.00100	*	.	.	.	31	272
3.00100	*	.	.	.	31	271
3.00100	*	.	.	.	31	270
3.00100	*	.	.	.	31	269
3.00100	*	.	.	.	31	268

phregparms_mianalyze_mi_4099

The LIFETEST Procedure

Stratum 4: Statin intensity at study enrollment = No use statin dose

Product-Limit Survival Estimates						
Prim_Outc_Time		Survival	Failure	Survival Standard Error	Number Failed	Number Left
3.00100	*	.	.	.	31	267
3.00100	*	.	.	.	31	266
3.00100	*	.	.	.	31	265
3.00100	*	.	.	.	31	264
3.00100	*	.	.	.	31	263
3.00900	*	.	.	.	31	262
3.00900	*	.	.	.	31	261
3.00900	*	.	.	.	31	260
3.00900	*	.	.	.	31	259
3.02800	*	.	.	.	31	258
3.04200	*	.	.	.	31	257
3.04400	*	.	.	.	31	256
3.04700	*	.	.	.	31	255
3.05300	*	.	.	.	31	254
3.06100	*	.	.	.	31	253
3.08000	*	.	.	.	31	252
3.08600	*	.	.	.	31	251
3.09900	*	.	.	.	31	250
3.09900	*	.	.	.	31	249
3.10500	*	.	.	.	31	248
3.12100		0.9560	0.0440	0.00849	32	247
3.15700	*	.	.	.	32	246
3.16200	*	.	.	.	32	245
3.17000	*	.	.	.	32	244
3.17600	*	.	.	.	32	243
3.18100	*	.	.	.	32	242
3.18100	*	.	.	.	32	241
3.18400	*	.	.	.	32	240
3.19500	*	.	.	.	32	239

phregparms_mianalyze_mi_4099

The LIFETEST Procedure

Stratum 4: Statin intensity at study enrollment = No use statin dose

Product-Limit Survival Estimates						
Prim_Outc_Time		Survival	Failure	Survival Standard Error	Number Failed	Number Left
3.20100	*	.	.	.	32	238
3.21400	*	.	.	.	32	237
3.21400	*	.	.	.	32	236
3.22000	*	.	.	.	32	235
3.23300	*	.	.	.	32	234
3.23900	*	.	.	.	32	233
3.25800	*	.	.	.	32	232
3.26900	*	.	.	.	32	231
3.27200	*	.	.	.	32	230
3.29400	*	.	.	.	32	229
3.29600	*	.	.	.	32	228
3.29900		0.9518	0.0482	0.00943	33	227
3.30700	*	.	.	.	33	226
3.30700	*	.	.	.	33	225
3.32100	*	.	.	.	33	224
3.33200	*	.	.	.	33	223
3.33200	*	.	.	.	33	222
3.34800	*	.	.	.	33	221
3.36800	*	.	.	.	33	220
3.37300	*	.	.	.	33	219
3.37300	*	.	.	.	33	218
3.37900	*	.	.	.	33	217
3.38900	*	.	.	.	33	216
3.41100	*	.	.	.	33	215
3.44400	*	.	.	.	33	214
3.44400	*	.	.	.	33	213
3.44400	*	.	.	.	33	212
3.44400	*	.	.	.	33	211
3.44400	*	.	.	.	33	210

phregparms_mianalyze_mi_4099

The LIFETEST Procedure

Stratum 4: Statin intensity at study enrollment = No use statin dose

Product-Limit Survival Estimates						
Prim_Outc_Time		Survival	Failure	Survival Standard Error	Number Failed	Number Left
3.44400	*	.	.	.	33	209
3.44700	*	.	.	.	33	208
3.45000	*	.	.	.	33	207
3.45000	*	.	.	.	33	206
3.45000	*	.	.	.	33	205
3.46300	*	.	.	.	33	204
3.50700	*	.	.	.	33	203
3.50700	*	.	.	.	33	202
3.51800	*	.	.	.	33	201
3.52600	*	.	.	.	33	200
3.52600	*	.	.	.	33	199
3.53500	*	.	.	.	33	198
3.53500	*	.	.	.	33	197
3.54300	*	.	.	.	33	196
3.54300	*	.	.	.	33	195
3.54300	*	.	.	.	33	194
3.54300	*	.	.	.	33	193
3.54300	*	.	.	.	33	192
3.57600	*	.	.	.	33	191
3.57600	*	.	.	.	33	190
3.57600	*	.	.	.	33	189
3.57800	*	.	.	.	33	188
3.57800	*	.	.	.	33	187
3.57800	*	.	.	.	33	186
3.57800	*	.	.	.	33	185
3.60000	*	.	.	.	33	184
3.60000	*	.	.	.	33	183
3.60000	*	.	.	.	33	182
3.66100	*	.	.	.	33	181

phregparms_mianalyze_mi_4099

The LIFETEST Procedure

Stratum 4: Statin intensity at study enrollment = No use statin dose

Product-Limit Survival Estimates						
Prim_Outc_Time		Survival	Failure	Survival Standard Error	Number Failed	Number Left
3.68000	*	.	.	.	33	180
3.68000	*	.	.	.	33	179
3.68800	*	.	.	.	33	178
3.69900	*	.	.	.	33	177
3.70200	*	.	.	.	33	176
3.71300	*	.	.	.	33	175
3.71300	*	.	.	.	33	174
3.71800	*	.	.	.	33	173
3.71800	*	.	.	.	33	172
3.71800	*	.	.	.	33	171
3.73700	*	.	.	.	33	170
3.73700	*	.	.	.	33	169
3.75100	*	.	.	.	33	168
3.75100	*	.	.	.	33	167
3.75600	*	.	.	.	33	166
3.77000	*	.	.	.	33	165
3.77500	*	.	.	.	33	164
3.78400	*	.	.	.	33	163
3.78900	*	.	.	.	33	162
3.78900	*	.	.	.	33	161
3.79500	*	.	.	.	33	160
3.79500	*	.	.	.	33	159
3.79700	*	.	.	.	33	158
3.80600	*	.	.	.	33	157
3.81100	*	.	.	.	33	156
3.81100	*	.	.	.	33	155
3.81400	*	.	.	.	33	154
3.81700	*	.	.	.	33	153
3.81700	*	.	.	.	33	152

phregparms_mianalyze_mi_4099

The LIFETEST Procedure

Stratum 4: Statin intensity at study enrollment = No use statin dose

Product-Limit Survival Estimates						
Prim_Outc_Time		Survival	Failure	Survival Standard Error	Number Failed	Number Left
3.81700	*	.	.	.	33	151
3.81900	*	.	.	.	33	150
3.82500	*	.	.	.	33	149
3.82800	*	.	.	.	33	148
3.83000	*	.	.	.	33	147
3.83300	*	.	.	.	33	146
3.83300	*	.	.	.	33	145
3.83300	*	.	.	.	33	144
3.83300	*	.	.	.	33	143
3.83300	*	.	.	.	33	142
3.83300	*	.	.	.	33	141
3.83300	*	.	.	.	33	140
3.83300	*	.	.	.	33	139
3.84100	*	.	.	.	33	138
3.84400	*	.	.	.	33	137
3.84400	*	.	.	.	33	136
3.84700	*	.	.	.	33	135
3.84700	*	.	.	.	33	134
3.84700	*	.	.	.	33	133
3.84700	*	.	.	.	33	132
3.84700	*	.	.	.	33	131
3.84900	*	.	.	.	33	130
3.84900	*	.	.	.	33	129
3.84900	*	.	.	.	33	128
3.84900	*	.	.	.	33	127
3.85200	*	.	.	.	33	126
3.85200	*	.	.	.	33	125
3.85200	*	.	.	.	33	124
3.85200	*	.	.	.	33	123

phregparms_mianalyze_mi_4099

The LIFETEST Procedure

Stratum 4: Statin intensity at study enrollment = No use statin dose

Product-Limit Survival Estimates						
Prim_Outc_Time		Survival	Failure	Survival Standard Error	Number Failed	Number Left
3.85200	*	.	.	.	33	122
3.85500	*	.	.	.	33	121
3.85500	*	.	.	.	33	120
3.85500	*	.	.	.	33	119
3.85500	*	.	.	.	33	118
3.85500	*	.	.	.	33	117
3.85500	*	.	.	.	33	116
3.86300	*	.	.	.	33	115
3.87100	*	.	.	.	33	114
3.87400	*	.	.	.	33	113
3.87400	*	.	.	.	33	112
3.87400	*	.	.	.	33	111
3.87400	*	.	.	.	33	110
3.87400	*	.	.	.	33	109
3.87400	*	.	.	.	33	108
3.87400	*	.	.	.	33	107
3.87400	*	.	.	.	33	106
3.87400	*	.	.	.	33	105
3.87400	*	.	.	.	33	104
3.88800	*	.	.	.	33	103
3.88800	*	.	.	.	33	102
3.89000	*	.	.	.	33	101
3.89900	*	.	.	.	33	100
3.89900	*	.	.	.	33	99
3.89900	*	.	.	.	33	98
3.90100	*	.	.	.	33	97
3.91000	*	.	.	.	33	96
3.91200	*	.	.	.	33	95
3.91800	*	.	.	.	33	94

phregparms_mianalyze_mi_4099

The LIFETEST Procedure

Stratum 4: Statin intensity at study enrollment = No use statin dose

Product-Limit Survival Estimates						
Prim_Outc_Time		Survival	Failure	Survival Standard Error	Number Failed	Number Left
3.91800	*	.	.	.	33	93
3.91800	*	.	.	.	33	92
3.93200	*	.	.	.	33	91
3.93200	*	.	.	.	33	90
3.93200	*	.	.	.	33	89
3.93200	*	.	.	.	33	88
3.93400	*	.	.	.	33	87
3.93700		0.9409	0.0591	0.0143	34	86
3.93700	*	.	.	.	34	85
3.93700	*	.	.	.	34	84
3.94300	*	.	.	.	34	83
3.94800	*	.	.	.	34	82
3.96700	*	.	.	.	34	81
3.97800	*	.	.	.	34	80
3.97800	*	.	.	.	34	79
3.98600	*	.	.	.	34	78
3.98600	*	.	.	.	34	77
3.98600	*	.	.	.	34	76
4.00000	*	.	.	.	34	75
4.00000	*	.	.	.	34	74
4.00300	*	.	.	.	34	73
4.00500	*	.	.	.	34	72
4.00500	*	.	.	.	34	71
4.01900	*	.	.	.	34	70
4.01900	*	.	.	.	34	69
4.02200	*	.	.	.	34	68
4.02200	*	.	.	.	34	67
4.02500	*	.	.	.	34	66
4.02700	*	.	.	.	34	65

phregparms_mianalyze_mi_4099

The LIFETEST Procedure

Stratum 4: Statin intensity at study enrollment = No use statin dose

Product-Limit Survival Estimates						
Prim_Outc_Time		Survival	Failure	Survival Standard Error	Number Failed	Number Left
4.02700	*	.	.	.	34	64
4.02700	*	.	.	.	34	63
4.02700	*	.	.	.	34	62
4.02700	*	.	.	.	34	61
4.03000	*	.	.	.	34	60
4.04100	*	.	.	.	34	59
4.04900	*	.	.	.	34	58
4.04900	*	.	.	.	34	57
4.06000	*	.	.	.	34	56
4.07100	*	.	.	.	34	55
4.07400	*	.	.	.	34	54
4.08500	*	.	.	.	34	53
4.08500	*	.	.	.	34	52
4.08500	*	.	.	.	34	51
4.08500	*	.	.	.	34	50
4.08500	*	.	.	.	34	49
4.08800	*	.	.	.	34	48
4.08800	*	.	.	.	34	47
4.09300	*	.	.	.	34	46
4.10100	*	.	.	.	34	45
4.10100	*	.	.	.	34	44
4.10700	*	.	.	.	34	43
4.10700	*	.	.	.	34	42
4.12000	*	.	.	.	34	41
4.13400	*	.	.	.	34	40
4.13700	*	.	.	.	34	39
4.13700	*	.	.	.	34	38
4.13700	*	.	.	.	34	37
4.13700	*	.	.	.	34	36

phregparms_mianalyze_mi_4099

The LIFETEST Procedure

Stratum 4: Statin intensity at study enrollment = No use statin dose

Product-Limit Survival Estimates						
Prim_Outc_Time		Survival	Failure	Survival Standard Error	Number Failed	Number Left
4.13700	*	.	.	.	34	35
4.13700	*	.	.	.	34	34
4.14000	*	.	.	.	34	33
4.14000	*	.	.	.	34	32
4.14000	*	.	.	.	34	31
4.14000	*	.	.	.	34	30
4.14000	*	.	.	.	34	29
4.14500	*	.	.	.	34	28
4.15300	*	.	.	.	34	27
4.15300	*	.	.	.	34	26
4.15300	*	.	.	.	34	25
4.15300	*	.	.	.	34	24
4.15600	*	.	.	.	34	23
4.15900	*	.	.	.	34	22
4.15900	*	.	.	.	34	21
4.15900	*	.	.	.	34	20
4.15900	*	.	.	.	34	19
4.15900	*	.	.	.	34	18
4.17200	*	.	.	.	34	17
4.17800	*	.	.	.	34	16
4.17800	*	.	.	.	34	15
4.18100	*	.	.	.	34	14
4.18300	*	.	.	.	34	13
4.18600	*	.	.	.	34	12
4.19200	*	.	.	.	34	11
4.21600	*	.	.	.	34	10
4.22200	*	.	.	.	34	9
4.22200	*	.	.	.	34	8
4.22700	*	.	.	.	34	7

phregparms_mianalyze_mi_4099

The LIFETEST Procedure

Stratum 4: Statin intensity at study enrollment = No use statin dose

Product-Limit Survival Estimates						
Prim_Outc_Time		Survival	Failure	Survival Standard Error	Number Failed	Number Left
4.23000	*	.	.	.	34	6
4.23000	*	.	.	.	34	5
4.25200	*	.	.	.	34	4
4.26800	*	.	.	.	34	3
4.37000	*	.	.	.	34	2
4.38300	*	.	.	.	34	1
4.75600	*	.	.	.	34	0

Note: The marked survival times are censored observations.

phregparms_mianalyze_mi_4099

The LIFETEST Procedure

Stratum 4: Statin intensity at study enrollment = No use statin dose

Summary Statistics for Time Variable Prim_Outc_Time

Quartile Estimates				
Percent	Point Estimate	95% Confidence Interval		
		Transform	[Lower	Upper)
75	.	LOGLOG	.	.
50	.	LOGLOG	.	.
25	.	LOGLOG	.	.

Mean	Standard Error
3.82807	0.01894

Note: The mean survival time and its standard error were underestimated because the largest observation was censored and the estimation was restricted to the largest event time.

Summary of the Number of Censored and Uncensored Values					
Stratum	statin_intensity_HML	Total	Failed	Censored	Percent Censored
1	High-intensity statin dose	183	2	181	98.91
2	Low-intensity statin dose	412	5	407	98.79
3	Medium-intensity statin dose	2338	68	2270	97.09
4	No use statin dose	1166	34	1132	97.08
Total		4099	109	3990	97.34

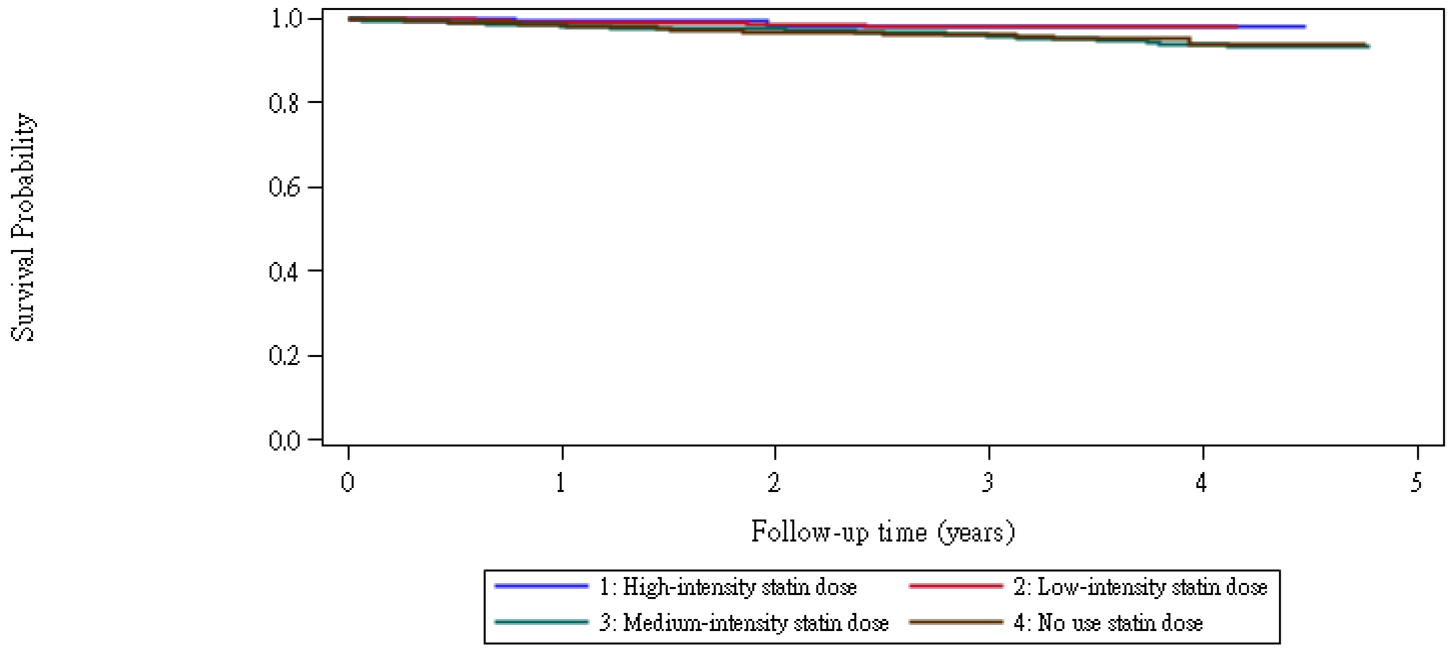

1: High-intensity statin dose	183	115	86	51	16	0
2: Low-intensity statin dose	412	270	160	42	7	0
3: Medium-intensity statin dose	2338	1491	1016	507	170	0
4: No use statin dose	1166	881	516	289	76	0

phregparms_mianalyze_mi_4099

The LIFETEST Procedure

Testing Homogeneity of Survival Curves for Prim_Outc_Time over Strata

Rank Statistics	
statin_intensity_HML	Log-Rank
High-intensity statin dose	-3.0492
Low-intensity statin dose	-4.8792
Medium-intensity statin dose	6.4077
No use statin dose	1.5207

Covariance Matrix for the Log-Rank Statistics				
statin_intensity_HML	High-intensity statin dose	Low-intensity statin dose	Medium-intensity statin dose	No use statin dose
High-intensity statin dose	4.8132	-0.4499	-2.8587	-1.5046
Low-intensity statin dose	-0.4499	8.9369	-5.5634	-2.9236
Medium-intensity statin dose	-2.8587	-5.5634	26.7574	-18.3353
No use statin dose	-1.5046	-2.9236	-18.3353	22.7635

Test of Equality over Strata			
Test	Chi-Square	DF	Pr > Chi-Square
Log-Rank	4.9990	3	0.1719

Adjustment for Multiple Comparisons for the Logrank Test				
Strata Comparison			p-Values	
statin_intensity_HML	statin_intensity_HML	Chi-Square	Raw	Sidak
High-intensity statin dose	Low-intensity statin dose	0.2286	0.6326	0.9975
High-intensity statin dose	Medium-intensity statin dose	2.3984	0.1215	0.5402
High-intensity statin dose	No use statin dose	0.6828	0.4086	0.9572
Low-intensity statin dose	Medium-intensity statin dose	2.7208	0.0990	0.4652
Low-intensity statin dose	No use statin dose	1.0908	0.2963	0.8786
Medium-intensity statin dose	No use statin dose	0.2771	0.5986	0.9958
